# Phosphetes via Transition Metal Free Ring Closure – Taking the Proper Turn at a Thermodynamic Crossing

**DOI:** 10.1002/chem.202101298

**Published:** 2021-06-02

**Authors:** Fabian Roesler, Máté Kovács, Clemens Bruhn, Zsolt Kelemen, Rudolf Pietschnig

**Affiliations:** ^1^ Institute for Chemistry and CINSaT University of Kassel Heinrich Plett-Straße 40 34132 Kassel Germany; ^2^ Department of Inorganic and Analytical Chemistry Budapest University of Technology and Economics Szt. Gellért tér 4 H-1111 Budapest Hungary

**Keywords:** cyclization, density functional calculations, luminescence, phosphorus heterocycles, silanes

## Abstract

A transition metal free route to phosphetes featuring an exocyclic alkene unit is presented. In this approach phosphanides are added to a variety of diynes generating phosphaallylic intermediates which depending on the reaction conditions transform either to phosphetes or the corresponding phospholes. Investigation of the reaction mechanism by combined quantum chemical and experimental means identifies phosphole formation as thermodynamically controlled reaction path, whereas kinetic control furnishes the corresponding phosphetes. Structural and luminescence properties of the rare class of phosphetes are explored, as well as for selected key intermediates.

## Graphical abstract

**New route**: Investigation of the phosphanide addition to alkynes revealed that the ring size can be controlled during the cyclization step leading either to phospholes or phosphetes starting from the same reactants.

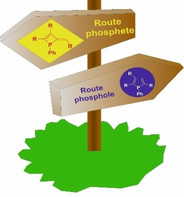

## Introduction

**Scheme 1:**
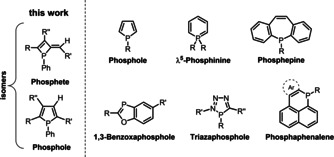
General sketch of fundamental fluorescent π‐conjugated phosphorus heterocycles.

Phosphorus heterocycles emerged as useful class of π‐conjugated luminophores for light emitting or light harvesting applications.^[1]^ Among the organophosphorus materials, phospholes are probably the most prominent examples for such purposes,^[2]^ besides λ^5^‐phosphinines^[3]^ and more sophisticated molecular scaffolds^[4]^ or systems with additional heteroatoms^[5]^ (Scheme 1). Phosphetes with an exocyclic methylene unit are in fact constitutional isomers of phospholes, and first examples containing the four‐membered heterocyclic motif have been obtained via rearrangement in the coordination sphere of transition metal fragments.^[6]^ Their potential revolving around their optoelectronic properties has been discovered only recently.[^6h^, ^7^] A peculiar feature of such strained heterocycles is their structural flexibility in terms of ring opening and closure which led to the description of phosphetes as masked 1‐phosphabutadienes.^[6b]^ Somewhat related is the addition of neutral phosphane units to an adjacent alkyne leading to phosphanylidene bridged stilbenes^[4e]^ or cyclic P‐ylidic mesoionic carbenes,^[8]^ which attracted attention as ligand system towards transition metal centers.

In recent studies we explored the preparation of β‐silyl substituted phospholes which besides their relevance for phosphole containing organic‐inorganic hybrid materials are also valuable precursors to β‐H substituted phospholes. The general synthetic access takes advantage of phosphanide addition to diynes,^[9]^ which is closely related to an approach originally developed by Märkl.^[10]^ Despite the high reactivity of phosphanides, this synthetic strategy is surprisingly tolerant to substituents carrying functional groups including organofluorine substituents.^[9a]^

Here we report our investigation shedding light on the mechanism of the phosphole formation via phosphanide to diyne addition. With experimental and computational measures, we explore a synthetic pathway giving selectively access to either the ring contracted four‐membered ring heterocyclic phosphetes or to phospholes, their five‐membered ring heterocyclic isomers, depending on the reaction conditions, with identification of key intermediates with spectroscopic and structural means. With a series of phosphetes in hand, their luminescence properties are explored for the silylated and desilylated congeners. An additional attractive feature of the desilylated phosphetes is their chiral nature, even for uniform substituents, for which the respective phosphole remains achiral.

## Results and Discussion

**Scheme 2:**
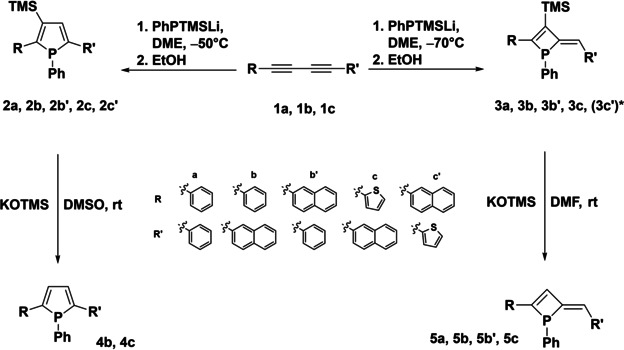
Synthesis of β‐silyl phospholes **2 a**, **2 b**, **2 b’**, **2 c, 2 c’** and the corresponding phosphetes **3 a**, **3 b**, **3 b’**, **3 c** and their desilylation to β‐H phosphetes **5 a**, **5 b**, **5 b’**, **5 c** and β‐H phospholes **4 b**, **4 c** (*: **3 c’** has not been isolated).

To investigate the mechanism of the phosphole formation via phosphanide addition to diynes, we varied the reaction parameters with careful in situ monitoring by ^31^P NMR. In doing so we realized the formation of a by‐product in considerable amounts at lower reaction temperatures (−70 °C). Upon isolation, we identified this by‐product as phosphete **3 a**, a constitutional isomer of phosphole **2 a**. Further investigations with different asymmetric starting diynes (**1 b** and **1 c**) showed, that the corresponding phosphetes (**3 b**, **3 c**) can be obtained in similar fashion as well, while at higher reaction temperature the corresponding phospholes (**2 b**, **2 c**) are the preferred products (Scheme 2). Starting from symmetric diyne **1 a** selectively one phosphole (**2 a**) or phosphete (**3 a**) is obtained depending on the reaction temperature. Using unsymmetrically substituted diynes **1 b** and **1 c**, however, yields two isomers for each ring‐size where R and R’ have switched positions in **2**, **3** (**b**, **b’**, **c**, **c’**) as outlined in Scheme 2. Based on NMR integration the ratio of these isomers is quite similar for phenyl‐naphthyl‐substituted **2 b**, **b’** (78 : 22 [%]) and thienyl‐naphthyl‐substituted **2 c, c’** (81 : 19 [%]) phospholes. By contrast, the corresponding phosphetes feature an almost 1 : 1 ratio for phenyl‐naphthyl‐substituted **3 b**, **3 b’** (54 : 46 [%]) while thienyl‐naphthyl‐substituted **3 c** is strongly favoured over **3 c’**, which can only be detected as a trace (ca. 1 %) at the limit of detection and isolation. It needs to be pointed out however, that for all isolated phosphetes shown in Scheme 2 only the *Z* isomers with respect to the exocyclic alkene unit are obtained exclusively. Moreover, the *Z*‐configuration shows a high thermal stability and no changes in *E*/*Z* configuration were observed upon heating to 200 °C.

**Figure 1:**
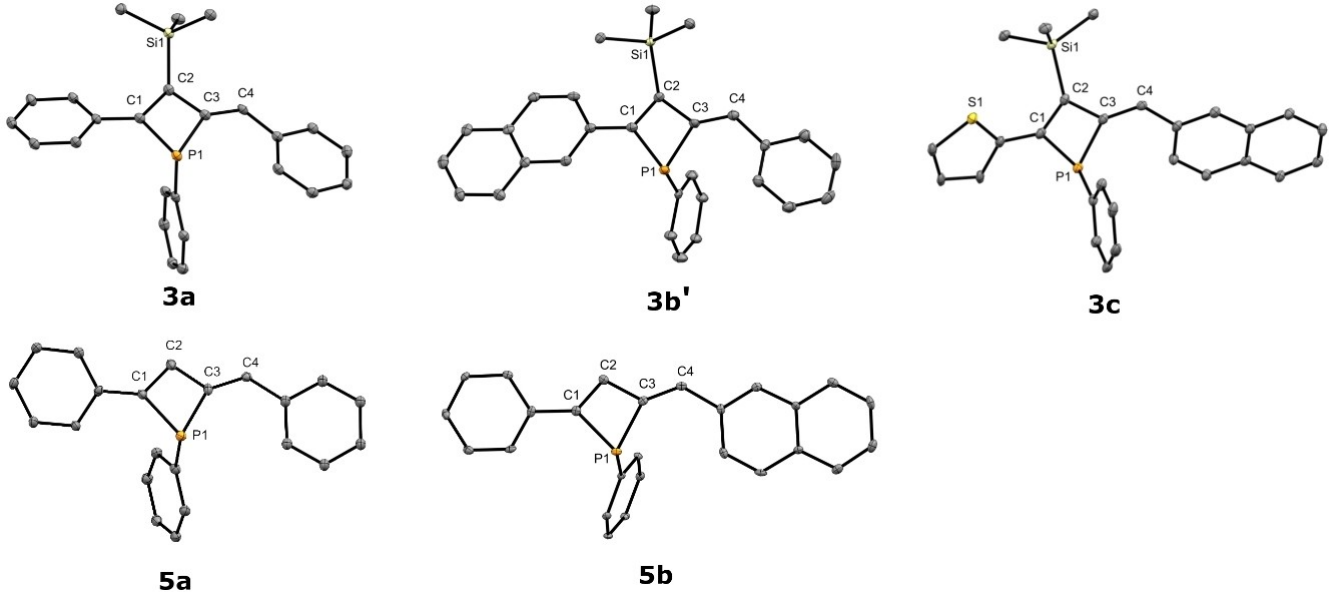
Molecular structure of β‐silyl phosphetes (top; **3 a** (left), **3 b’** (middle) and **3 c** (right)) and β‐H phosphetes (bottom; **5 a** (left) and **5 b** (right)) in the solid state. Thermal ellipsoids drawn at 30 % probability level. For clarity, hydrogen atoms have been omitted as well as disordered positions. Important bond lengths and angles of **3 a**: P(1)−C(1) 1.846 (4) Å, P(1)−C(3) 1.854 (4) Å, (P(1)−C(20) 1.829 (5) Å; C(1)−P(1)−C(3) 72.3 (2)°, C(1)−P(1)−C(20) 105.9 (2)°, C(3)−P(1)−C(20) 104.7 (2)°; **3 b’**: P(1)−C(1) 1.836 (3) Å, P(1)−C(3) 1.845 (3) Å, P(1)−C(21) 1.861 (18) Å, P(1)−C(21 A) 1.796 (19) Å; C(1)−P(1)−C(3) 71.6 (1)°, C(1)−P(1)−C(21) 106.1 (5)°, C(3)−P(1)−C(21) 106.2 (5)°; **3 c**: P(1)−C(1) 1.835 (6) Å, (P(1)−C(3) 1.853 (6) Å, P(1)−C(18) 1.844 (6) Å; 72.0 (3)° C(1)−P(1)−C(3), 104.0 (3)° C(3)−P(1)−C(18), 103.7 (3)° C(1)−P(1)−C(18); **5 a**: P(1)−C(1) 1.841 (11) Å, P(1)−C(3) 1.849 (15) Å, P(1)−C(17) 1.847 (8) Å; C(1)−P(1)−C(3) 72.4 (6)°, C(1)−P(1)−C(17) 106.4 (5), C(3)−P(1)−C(17) 103.7 (6); **5 b**: P(1)−C(1) 1.871 (4) Å, P(1)−C(3) 1.862 (4) Å, P(1)−C(21) 1.836 (3) Å; C(1)−P(1)−C(3) 71.88 (17), C(1)−P(1)−C(21) 103.95 (17), C(3)−P(1)−C(21) 105.36 (18).

Phosphetes **3** all feature ^31^P NMR resonances in a similar region (32.1 (**3 a**), 32.1 (**3 b**), 31.5 (**3 b’**), 37.4 (**3 c**), 35.2 (**3 c’**) ppm) consistently at lower field (Δδ: ca. 10 ppm) compared with the corresponding phospholes **2** (22.3 (**2 a**), 22.6 (**2 b**), 22.4 (**2 b’**), 24.7 (**2 c**), 24.5 (**2 c’**) ppm). In analogy to the known desilylation of silyl phospholes^[9a]^ giving access to the corresponding β‐H phospholes (**4 b**, **4 c**), we explored desilylation of phosphetes **3 a**, **3 b**, **3 b’** and **3 c** as well (Scheme 2, bottom). As intended, the trimethylsilyl (TMS) group can be removed by reaction with KOTMS in DMF yielding β‐H phosphetes **5 a**, **5 b**, **5 b’** and **5 c** in excellent yields of ca. 90 %. In the ^31^P NMR spectra, the β‐H phosphetes resonate at 18.3 (**5 a**), 18.5 (**5 b**, **5 b’**) and 25.1 (**5 c**) ppm which is significantly shielded with respect to their β‐silyl phosphete counterparts. The ^31^P NMR resonances of isomeric β‐H phosphetes **5 b** and **5 b’** are isochronic but both isomers can be conveniently distinguished in the ^1^H NMR spectra based on the signals for the vinyl‐H indicating a ratio of 60 : 40 [%] which is comparable to the ratio of the isomers in the starting β‐silyl phosphetes **3 b**, **3 b’** (54 : 46 [%]). Again, the *Z*‐configuration at the exocyclic alkene unit remains unchanged upon desilylation. Constitution and structural situation of β‐silyl phosphetes **3 a**, **3 b’** and **3 c** as well as β‐H phosphetes **5 a** and **5 b** are further illustrated by single‐crystal X‐ray diffraction (Figure 1).

The substituents at phosphorus adopt a pyramidal geometry in all phosphetes (Σ_P_=282.9 (3)° (**3 a**), 283.9 (7)° (**3 b’**), 279.8 (5)° (**3 c**), 281.9 (7)° (**5 a**) and 281.2 (3)° (**5 b**)) with marginally smaller angular sum at phosphorus in the β‐H phosphetes. The endocyclic C−P−C angle within the central phosphete rings is close to 72° in all cases (72.3 (2)° (**3 a**), 71.6 (1)° (**3 b’**), 72.0 (3)° (**3 c**), 72.4 (6)° (**5 a**) and 71.88 (17)° (**5 b**)) which is significantly more acute than in previously reported phosphetes complexes by Mathey et al. (ca. 87°)^[6b]^ but similar to zirconaphosphetes without P‐coordination by Majoral.^[6e]^ The phosphete ring is slightly folded with a larger deviation of the phosphorus atom from the C1−C2−C3 plane for the β‐silyl phosphetes (**3 a**: 0.351(11), **3 b′**: 0.274(6), **3 c**: 0.235(14) [Å]) as compared with their corresponding β‐H counterparts (**5 a**: 0.22(4), **5 b**: 0.197(11) [Å]). The P1−C1 bond lengths in the here reported phosphete structures (1.835 (6) ‐1.871 (4) Å) are at or in‐between those found for zirconaphosphetes by Majoral (1.844)^[6e]^ and the ones for phosphete complexes by Mathey et al. (1.93 Å)^[6b]^

Regarding conjugation and luminescence properties the torsion of the adjacent rings is of special interest.^[9a]^ For β‐silyl phosphetes the aromatic rings attached to C1 of the phosphete ring are twisted by 27.5 (2)° (**3 a**), 24.8 (1)° (**3 b’**) and 22.2 (6)° (**3 c**) respectively, while the torsion angles between phosphete ring and the substituents at the exocyclic alkene (P1−C3−C4−C5) are 4.4 (6)° (**3 a**), 8.1 (4)° (**3 b’**) and 10.8 (2)° (**3 c**). In desilylated **5 a** and **5 b** however, the phosphete and the adjacent phenyl ring feature a near coplanar arrangement (5.6 (7)° (**5 a**) and 8.67(14)° (**5 b**)) which is also true for the torsion of the aryl rings at C4 (P1−C3−C4−C5: 2.0 (2)° (**5 a**) and 1.9 (6)° (**5 b**)). This coplanarity in β‐H phosphetes enables improved π‐delocalization and may be attributed to the absence of the sterically more demanding TMS group, as in related P‐heterocycles.^[9a]^

### Mechanism of formation

In light of the subtle balance between the competitive formation of either phosphetes or phospholes, we set out to investigate the mechanism of this reaction with theoretical means and DFT calculations were performed at ωB97X‐D/6‐311+G** level of theory. Since the DME used as solvent is prone to Li^+^ complexation, solvent separated ion pairs can be anticipated. Therefore, the “naked” anionic systems were selected for the investigation of the reaction mechanism, however several steps were checked in the presence of Li^+^ and Li(DME)_n_^+^ as well (more details in the Supporting Information, Table S1‐S3).

**Scheme 3:**
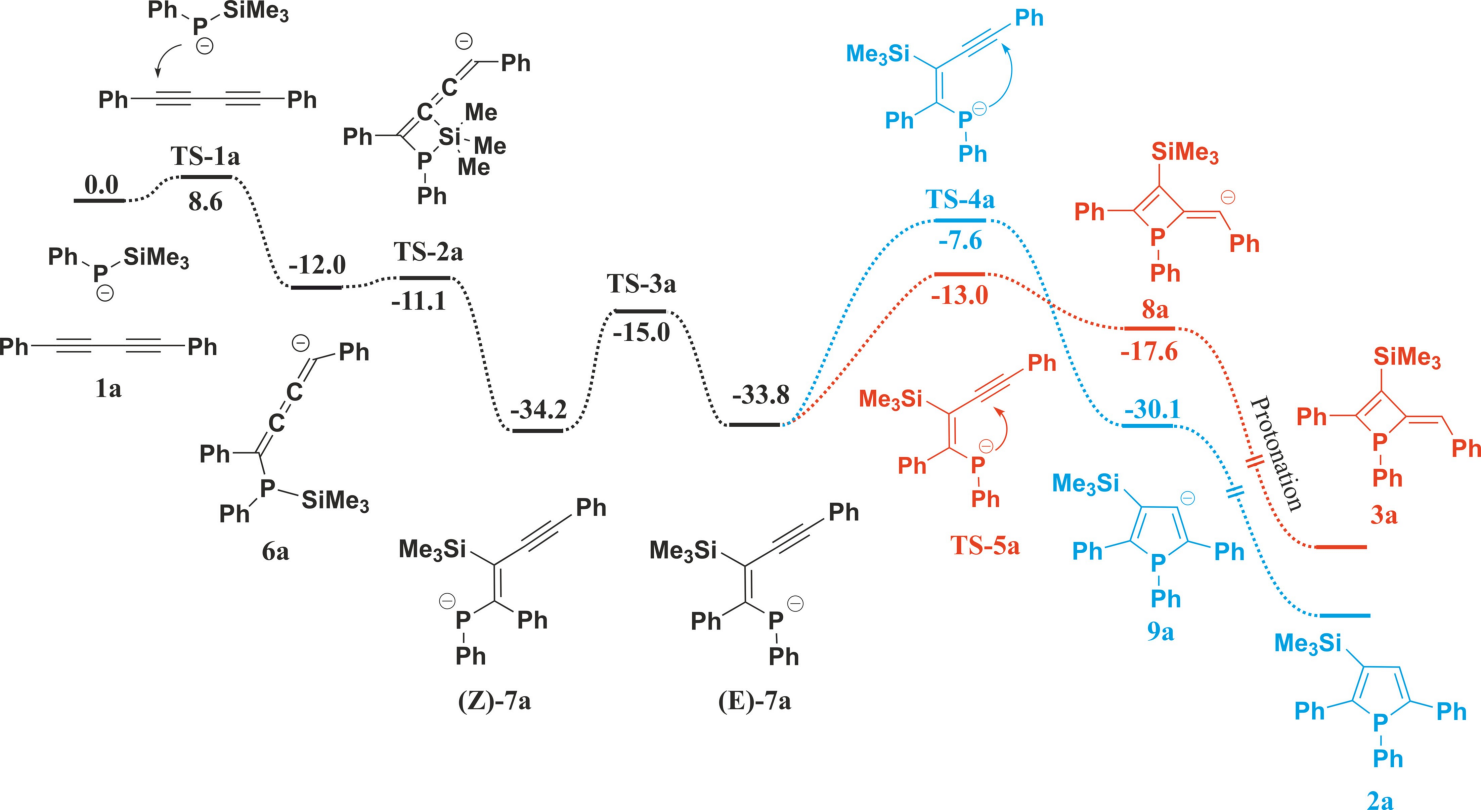
Calculated reaction mechanism of the formation of phosphole **2 a** (blue) and phosphete **3 a** (red) at ωB97X‐D/6‐311+G** level of theory (relative energies in kcal/mol).

According to our findings (Scheme 3 and Figure S1 depict the mechanism starting from **1 a**, tabulated data for the systems **b**, **b’**, **c**, and **c’** were in Table S1 in the Supporting Information), the initial step is the nucleophilic attack of the phosphanide anion (PhPTMSLi) to one of the triple bonds of **1 a‐c**, requiring a low energy barrier (**TS‐1 a**–**c’** ▵E^#^=4.7–8.6 kcal/mol). The resulting vinyl anions (**6 a**–**c’**) subsequently stabilizes via silyl migration, during which a weaker P−Si is cleaved in favour of a more stable C−Si bond, moreover the negative charge gets delocalized in the 1‐phosphaallylic π‐system, which gave exceptional stability to this intermediate. 1‐Phosphaallylic anion **(*Z*)‐7 a**–**c’** is accessible via a very low energy barrier (**TS‐2 a**–**c’**, ▵E^#^=0.0–1.1 kcal/mol) from phosphanylcumulene anion **6 a**–**c’** proceeding through a transition state in which the silicon is pentacoordinate (SN2 type reaction, Figure S2). The forming 1‐phosphaallylic anions (**(Z)‐7 a**–**c’** have Z‐configuration) isomerize to the corresponding *E‐*isomers (**(*E*)‐7 a**–**c’**), which are somewhat less stable (by 0.2–1.0 kcal/mol),^[11]^ but suitable to undergo ring closure via nucleophilic attack on the adjacent alkyne unit furnishing either a 4‐ or 5‐membered ring (**8 a**–**c’** or **9 a**–**c’**) depending on the site of attack at the alkyne (**TS‐4 a**–**c’**, **TS‐5 a**–**c’**). Based on the experimentally observed temperature dependence, phosphetes can be considered as the kinetic products, while the formation of phospholes occurs under thermodynamic control. Indeed, phosphole intermediates (**9 a**–**c’**) are more stable by 10.3‐12.8 kcal/mol, than their phosphete isomers and the barrier for ring closure is lower by 1.4–6.9 kcal/mol in favour of phosphetes (Scheme 3).

Upon protonation, the energy difference between corresponding phosphetes and phospholes increases further 7.2–8.6 kcal/mol. It should be highlighted that phosphaallylic anions (**7 a**–**c’**) exhibit higher stability (see Scheme 3 and Table S1) than the corresponding anionic ring closed systems, therefore the question arises what is the thermodynamic driving force of the formation of the phosphole and phosphete products. As both products do not undergo spontaneous ring opening, protonation during the quenching step obviously traps the respective anionic species. The order of stability of the anionic species is **(*E*)‐7 a**–**c’**>**9 a**–**c’**>**8 a**–**c’** (see Table S1), whereas for the protonated systems the stability order changes with **2 a**–**c’** and **3 a**–**c’** exhibiting higher stability than vinylphosphanes **(*E*)‐10 a**–**c’** by 38.9–40.9 kcal/mol and 18.9–21.6 kcal/mol (Table S4), respectively. Obviously, the protonation step (quenching the reaction mixture) plays a prominent role in the selectivity of the reaction.

Owing to the limited acidity of the alcohol usually applied for quenching purposes the thermodynamically most stable protonated species **2** or **3** are formed but no protonated intermediates. We wondered what the outcome of the reaction might be in the presence of a more acidic quenching agent, allowing more rapid protonation. Quenching the reaction mixture with saturated NH_4_Cl‐solution or diluted aq. HCl indeed yielded the corresponding alkynyl compounds **10 a**, **10 b**, **10 c**, while aq. ammonia or ethanol did not. Alkynyl vinylphosphanes **(*E*)‐10 a**, **(*E*)‐10 b**, **(*E*)‐10b’** and **(*E*)‐10 c** are initially formed as single isomers which subsequently undergo *E*/*Z* isomerization which is partially slow on the NMR time scale. For **10 a** the primary (*E*)‐isomer shows a signal at −37.7 ppm in the ^31^P NMR with a ^1^ *J*_H‐P_‐coupling of 221.3 Hz whereas after four weeks a second more deshielded species (*δ*(^31^P): −30.5 ppm, ^1^ *J*_H‐P_=229.8 Hz) is present (ratio *E*/*Z*=3 : 1). **(*E*)‐10 b**, **(*E*)‐10b’** and **(*E*)‐10 c** show similar isomerization, selected NMR data are summarized in Table 1. The primary isomers featuring the higher field resonances the values of the ^3^ *J*_Si‐P_ coupling constants are significantly larger which supports their tentative assignment to the *E*‐isomer.^[12]^ In full agreement with the presence of both *E* and *Z* isomers, the energy differences between them are tiny (ΔE=0.2–1.0 kcal/mol).

**Table 1:** Summarized ^31^P and ^29^Si NMR data of compounds **10** in CD_2_Cl_2_. For compound **10 b** the configuration could not be determined.

Compound	^31^P{^1^H}NMR [ppm]	^1^*J*(^1^H‐^31^P) [Hz]	^29^Si{^1^H}NMR [ppm]	^3^*J*(^29^Si‐^31^P) [Hz]
**(*E*)‐10 a (*Z*)‐10 a**	−37.7 −30.5	221.3 229.8	−4.3 −3.5	7.5 3.4
**10 b**	−39.3 −39.8 −32.8 −32.1	221 221 229 229.8	– – – ‐3.4	– – – 3.4
**(*E*)‐10 c (*Z*)‐10 c**	−39.7 −28.4	221.3 229.7	−3.7 −2.9	10.9 2.5

**Scheme 4:**
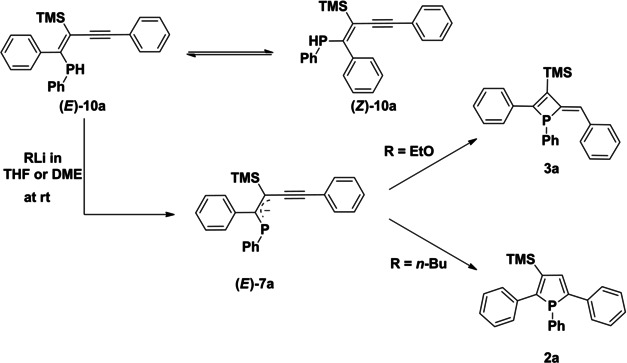
Reaction of phosphane **10 a** with EtOLi and *n*BuLi in THF and DME.

We wondered whether the corresponding phosphete or phosphole is available from these isolated protonated intermediates. Therefore, we investigated the deprotonation of alkynyl vinylphosphanes **10 a** with EtOLi in DME or THF which indeed resulted in the formation of phosphete **3 a** (Scheme 4). During the reaction, 1‐phosphaallylic anion **(*E*)‐7 a** is formed as an intermediate and its ^31^P NMR chemical shift at 12.4 ppm is comparable to literature known phosphaallylic anions by Niecke et al.^[13]^ Similarly, alkynyl vinylphosphanes **10 b**, **10 b’** and **10 c** upon deprotonation convert via phosphaallylic anions into the corresponding phosphetes **3 b**, **3 b’** and **3 c** at room temperature as well. The transition states of the ring closures were calculated in the presence of one EtOH molecule (see Figure S3). In agreement with the experiments the barrier of the four membered ring formation is somewhat lower in energy (by 1.9 kcal/mol), than the corresponding transition state of the formation of phosphole ring. By contrast, replacing EtOLi with *n*BuLi (and subsequent quenching with EtOH at r.t.) leads to formation of the corresponding β‐silyl phosphole. These findings corroborate the reaction mechanism outlined above and the prominent role of the quenching conditions.

**Scheme 5:**
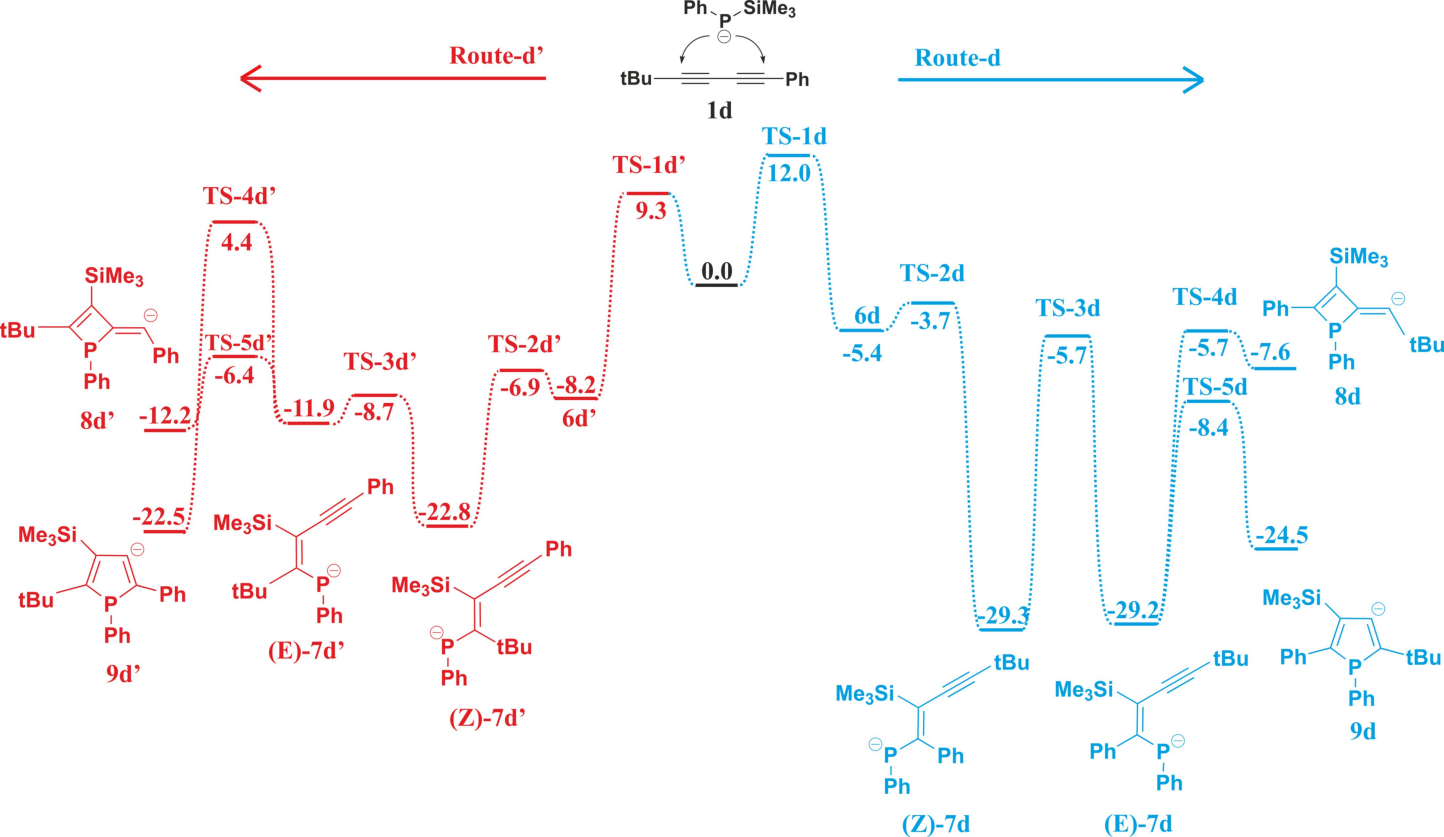
Reaction paths starting from **1 d**. For better clarity only the most important intermediates were shown (relative energies calculated at ωB97X‐D/6‐311+G** level of theory in kcal/mol unit).

**Scheme 6:**
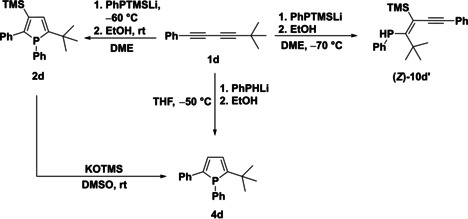
Synthesis with *t*‐butyl substituted diyne **1 d**.

Apart from the importance of the protonation step in locking the underlying species from further transformation, we wondered how the substitution pattern may affect or even tune the charge distribution and stability in the underlying intermediates. It can be anticipated that changing the R’ aryl substituent at one alkyne unit to an alkyl group shifts the reaction towards the phosphole product, as in deprotonated phosphete (**8 d**, Scheme 5) the alkyl substituents cannot stabilize the adjacent negative charge (thermodynamic destabilization of the product) and, moreover, the alkyl substituted alkyne unit in **7 d** is less prone to nucleophilic attack by the phosphanide *viz*. 1‐phosphaallylic anion. To test this hypothesis *t*‐butyl substituted diyne **1 d** was selected, which exclusively yields phosphole **2 d** without any phosphete formation (Scheme 6) in full agreement with our DFT calculations favouring the phosphole both kinetically (ΔE_(TS‐4d‐TS‐5d)_=2.7 kcal/mol) and thermodynamically (ΔE_(8d‐9d)_=16.9 kcal/mol) (see Table S5 and S6 in Supporting Information).

In addition, a second phosphole isomer (nucleophilic attack of the phosphanide anion to the other triple bond of **1 d**) could not be detected, however intermediate **10d’** has been identified and isolated, when the reaction was carried out at low temperature. Surprisingly, according to the ^3^ *J*_Si‐P_ coupling constant the observed isomer has *Z* configuration, which is in marked contrast to the configuration of the previously obtained (*E*)‐alkynyl‐vinylphosphanes. Investigating this reaction pathway by DFT calculations established that the corresponding *E* isomer **(*E*)‐10d’** is destabilized by steric repulsion between the bulky trimethylsilyl‐ and *t‐*butyl groups. Isodesmic reaction (Scheme S1 in the Supporting Information) verified that the origin of the destabilization is steric and amounts to around 12.1 kcal/mol. The isolation of this intermediate is a strong confirmation of the proposed reaction mechanism and underlines the importance of the isomerization of the phosphaallylic anion (**7 a**–**c’**).

**Figure 2:**
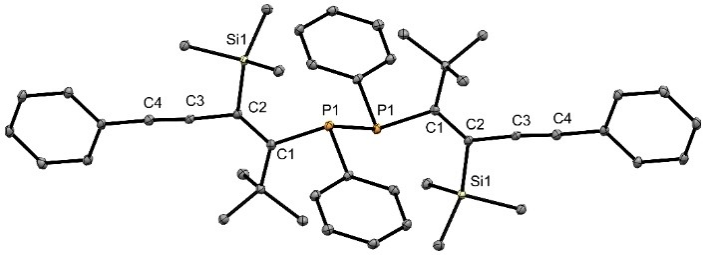
Molecular structure of **11** in the solid state. Thermal ellipsoids drawn at 30 % probability level. For clarity, hydrogen atoms have been omitted. Important bond lengths and angles of **11**: P(1)−P(1) 2.255(2) Å, P(1)−C(1) 1.853(3) Å, P(1)−C(15) 1.843(4) Å, C(1)−C(2) 1.364(5) Å, P(1)−C(1) 1.853(3) Å, C(2)−Si(1) 1.930(2) Å, C(2)−C(3) 1.434(5) Å, C(3)−C(4) 1.205(5) Å, P(1)−C(1) 1.853(3) Å, C(4)−C(5) 1.428(5) Å; P(1)−P(1)−C(1) 106.1 (1)°, P(1)−C(1)−C(2) 110.6 (2)°, C(1)−P(1)−C(15) 103.0 (2)°, C(1)−C(2)−C(3) 122.2 (2)°, C(2)−C(3)−C(4) 175.2 (3)°.

Alkyl‐substituted intermediate **(*Z*)‐10d’** is sensitive towards oxidation and partially oxidized during isolation via column chromatography, resulting in the corresponding diphosphane (**11**). The molecular structure of the dimer (**11**) was established by single crystal X‐ray diffraction shown in Figure 2.

The ^31^P NMR spectra of **11** in solution shows a signal at 4.4 ppm, which are in a range comparable to compounds known in the literature.^[14]^ Further spectroscopic characterization of diphosphane **11** was not pursued as it exceeds the scope of our investigation and furthermore was obtained as a minor by‐product only (<1 %).

**Figure 3:**
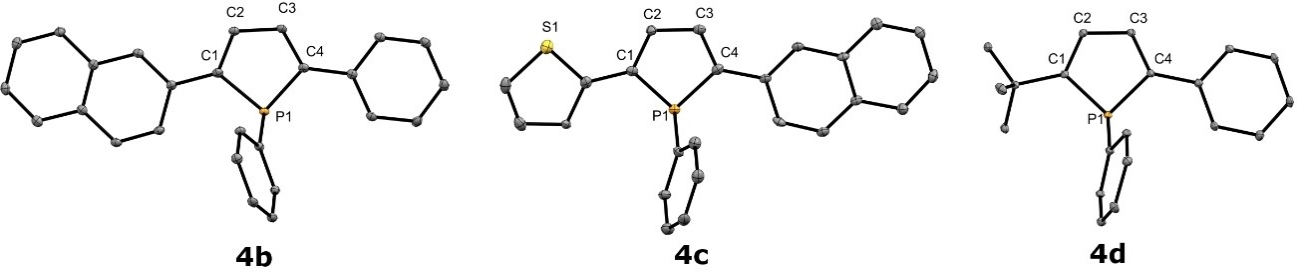
Molecular structure of **4 b**, **4 c** and **4 d** in the solid state. Thermal ellipsoids drawn at 30 % probability level. For clarity, hydrogen atoms and disordered P‐substituted phenyl ring (**4 d**) have been omitted. Important bond lengths and angles of **4 b**: P(1)−C(1) 1.826 (3) Å, P(1)−C(4) 1.828 (12) Å, P(1)−C(21) 1.836 (3) Å; C(1)−P(1)−C(4) 91.61 (15)°, C(4)−P(1)−C(21) 105.22 (14), C(1)−P(1)−C(21) 104.09 (15); **4 c**: (C1)−(P1) 1.817(7) Å, (P1)−(C4) 1.809(7) Å, (P1)−(C19) 1.826(7) Å; (C4)−(P1)−(C19) 105.9(3)°, (C1)−(P1)−(C19) 104.7(3)°, (C1)−(P1)−(C4) 91.1(3)°; **4 d**: P(1)−C(1) 1.814 (4) Å, P(1)−C(4) 1.814 (4) Å, P(1)−C(15) 1.840 (4) Å; C(1)−P(1)−C(4) 91.49 (18), C(4)−P(1)−C(15) 105.39 (17), C(1)−P(1)−C(15) 107.38 (18).

As there are not many examples of β‐H phospholes in the literature,[^9a^, ^10^, ^15^] the molecular structures of phospholes **4 b**, **4 c** and **4 d** were determined in the solid state (Figure 3). The attached aromatic rings in **4 b**, **4 c** and **4 d** feature a coplanar arrangement 4.20(11)°, 3.56(9)°, 0.2(0.2)°, 5.6(2)° and 5.2(1)°. The phosphorus atom shows a pyramidal environment in all cases Σ_P_=300.9(3)°, 301.7(5)° and 302.3(3)°. These data as well as the bond distances are in a typical range for this type of compounds. However, owing to the larger ring size in phospholes, the angular sums at phosphorus adopt larger values than in the isomeric phosphetes (see above).

### Luminescence properties

**Figure 4:**
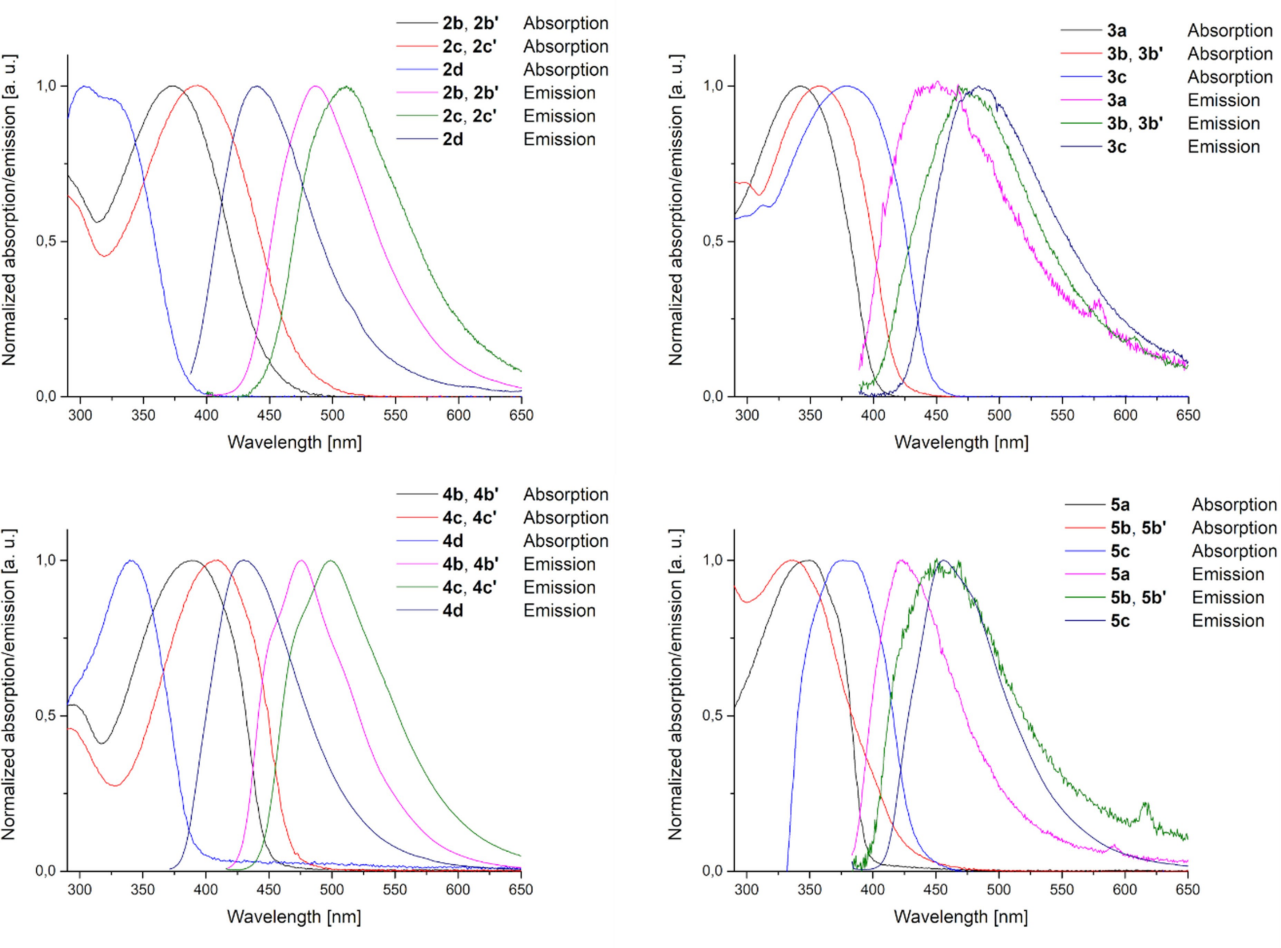
Measured absorption and emission spectra of all synthesized phosphetes and phospholes in CH_2_Cl_2_ solution (5×10^−5^ M). The spectra show the normalized absorption and emission, arbitrarily setting the maximum intensity as equal for both.

While phospholes are prominent luminescent compounds,[^9a^, ^9b^, ^16^] much less information is available for phosphetes in this respect and owing to their air sensitivity only photophysical properties of phosphete derivatives such as their oxides or gold complexes have been investigated so far.^[6h]^ Therefore, we were curious to explore the luminescence properties of the phosphetes prepared by us. The absorption and emission spectra of all synthesised phosphetes and phospholes are shown in Figure 4.

**Figure 5:**
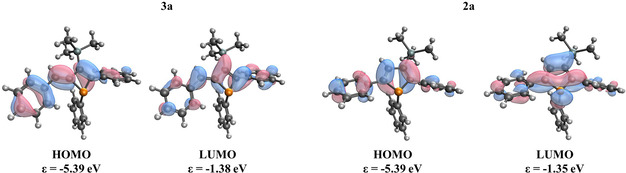
The calculated Kohn‐Sham frontier orbitals of **3 a** and **2 a** at B3LYP/6‐31G*//ωB97X‐D/6‐311+G** level of theory.

A comparison of the optical properties of phosphetes and the corresponding, isomeric phospholes shows an absorption maximum between 342–378 and 362^[9a]^–393 nm. The absorption maxima show a hypsochromic shift (between 15–28 nm) due to the smaller ring system. The relatively small differences between the absorption maxima of the phosphetes and phosphole are in agreement with their very similar energy level of the frontier orbitals (Figure 5). The strong absorption can be attributed to HOMO‐LUMO transition according to TD‐DFT calculations (Table S7 in Supporting Information).

Interestingly, the silyl phosphetes show almost no emission (*ϕ*=0.6–1.1 %) whereas the β‐silyl phospholes show an emission maximum with a quantum yield of *ϕ*=3.5–18.7 %. The desilylated phospholes have an even higher quantum yield (*ϕ*=15.4–56.9%). In contrast, the quantum yield of the β‐H phosphetes is unchanged. The only exception is **5 c**. This has a quantum yield of *ϕ*=12.6 % at 456 nm. The emission maximum of the isomeric phosphole is at 499 nm with *ϕ*=39.1 %. Further details are shown in Table S7 in the Supporting Information. Phosphetes and phospholes have comparable absorption coefficients, with somewhat blue shifted absorption maxima for the phosphetes. Overall, the photophysical properties of phosphetes are comparable with those of the corresponding phospholes, with the advantage of largely unexplored possibilities for photoresponsive materials, devices, and applications.

## Conclusion

In summary, we have shown that phosphetes are accessible under transition‐metal free condition*s* **via** phosphanide addition to a variety of diynes, selectively featuring *Z*‐configuration at the exocyclic alkene unit.

The combination of experiment and theory during the investigation of the reaction mechanism provided a plausible mechanistic picture and revealed a delicate balance between phosphete and phosphole formation as competing processes. Alkynyl substituted phosphaallylic systems have been identified as key intermediates. The availability of both isomeric heterocyclic systems, phosphetes and phospholes, at a time allows direct comparison of their photophysical properties as a function of ring size. In addition, the here presented phosphetes are P‐stereogenic compounds even for the cases where the corresponding symmetrically substituted phospholes are not. The new facile and variable access to this emerging compound class expands their potential for future applications owing to their chemical and photophysical properties in analogy to their phosphole counterparts.

## Experimental

Full details of experimental procedures, synthetic protocols and characterisation, NMR date, crystallographic data and computational details can be found in the Supporting Information.

Deposition Number(s) 2067623 (for **3 a**)„ 2067624 (for **5 b**), 2067625 (for **5 a**), 2067626 (for **4 d**), 2067627 (for **1 c**), 2067628 (for **3 c**), 2067629 (for **3 b’**), 2067630 (for **4 b**), 2067631 (for **4 c**), 2067632 (for **1 d**), 2067633 (for **1 l**), and 2075536 (for **1 b**) contain(s) the supplementary crystallographic data for this paper. These data are provided free of charge by the joint Cambridge Crystallographic Data Centre and Fachinformationszentrum Karlsruhe Access Structures service www.ccdc.cam.ac.uk/structures.

## Conflict of interest

The authors declare no conflict of interest.

## Supporting information

As a service to our authors and readers, this journal provides supporting information supplied by the authors. Such materials are peer reviewed and may be re‐organized for online delivery, but are not copy‐edited or typeset. Technical support issues arising from supporting information (other than missing files) should be addressed to the authors.

Supplementary
