# Supplementary material for: Phosphetes via Transition Metal Free Ring Closure – Taking the Proper Turn at a Thermodynamic Crossing
Source: Chemistry. 2021 Jun 2;27(38):9782–90. doi: 10.1002/chem.202101298 (PMC8361766; doi:10.1002/chem.202101298)
Supplement: Supplementary file 1 — Supplementary [file CHEM-27-9782-s001.pdf]

Chemistry–A European Journal

Supporting Information

Phosphetes via Transition Metal Free Ring Closure – Taking the Proper Turn at a Thermodynamic Crossing

Fabian Roesler, Máté Kovács, Clemens Bruhn, Zsolt Kelemen,* and Rudolf Pietschnig*

Content:

Table S1

Table S2

Table S3

Fig. S1

Fig. S2

Fig. S3

Table S4

Table S5

Table S6

Scheme S1

Table S7

a) Experimental procedures

b) Synthetic protocols and characterization

c) NMR data

d) Crystallographic data

e) Computational details

Table S1 Relative energies (in kcal/mol and calculated at ωB97X-D/6-311+G** level of theory) of the investigated intermediates and transition states.

substitution pattern	**1a-c + PhSiMe$_3$P$^-$**	**TS-1a-c'**	**6a-c'**	**TS-2a-c'**	**(Z)-7a-c'**	**TS-3 a-c'**	**(E)-7a-c'**	**TS-5a-c'**	**8a-c'**	**TS-4a-c'**	**9a-c'**
a	0.0	8.6	-12.0	-11.1	-34.2	-15.0	-33.8	-13.0	-17.6	-7.6	-30.1
b	0.0	7.9	-13.9	-12,3	-35.2	-17.2	-34.9	-16.0	-21.3	-9.0	-32.5
b'	0.0	5.0	-13.7	-13.3	-36.8	17,7	-35.8	-14.8	-19.2	-10.0	-32.0
c	0.0	8.0	-14.4	-14.4	-35.8	-18.7	-35.6	-16.3	-22.7	-14.9	-34.1
c'	0.0	4.7	-15.4	-13,6	-36.7	-18,4	-35.9	-15.1	-21.7	-10.6	-32.0

Table S2 Relative energies of the anionic systems in the presence of Li^+ cation at ωB97X-D/6-311+G** level of theory and in kcal/mol unit.

substitution pattern	**R**	**R'**	**(Z)-7a-c' + Li$^+$**	**(E)-7a-c' + Li$^+$**	**8a-c' + Li$^+$**	**9a-c' + Li$^+$**
a	phenyl	phenyl	6.1	0.0	13.8	-4.7
b	phenyl	naphtyl	-6.9	0.0	11.0	-6.5
b'	naphtyl	phenyl	-7.6	0.0	14.0	-4.5
c	thienyl	naphtyl	-5.4	0.0	10.2	-7.4
c'	naphtyl	thienyl	-12.8	0.0	12.0	-7.6

Table S3 Relative energies of the anionic systems in the presence of $Li(DME)^+$ cation at ωB97X-D/6-311+G** level of theory and in kcal/mol unit.

**(E)-7a + Li(DME)$^+$**	**8a + Li(DME)$^+$**	**9a + Li(DME)$^+$**
0.0	10.9	-7.2

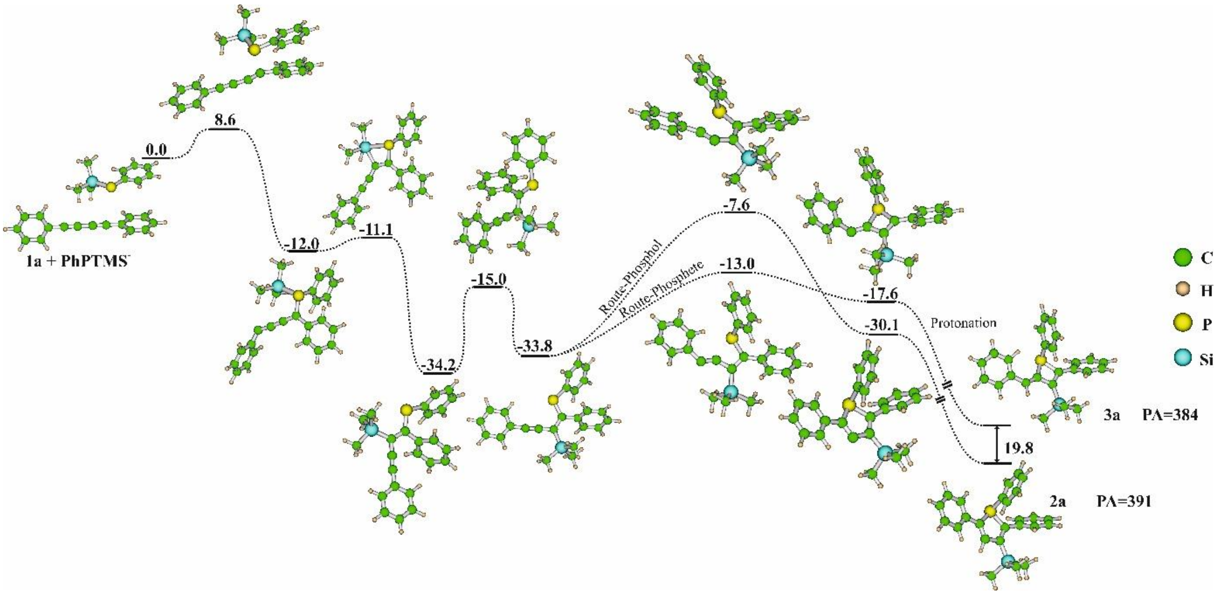

Fig. S1 Reaction energy profile of the reaction of **1d** with PhSiMe3P$^-$ anion at ωB97X-D/6-311+G** level of theory and in kcal/mol unit. Intermediates and transition states were represented by the optimized geometries of the systems (ball-and-sticks model).

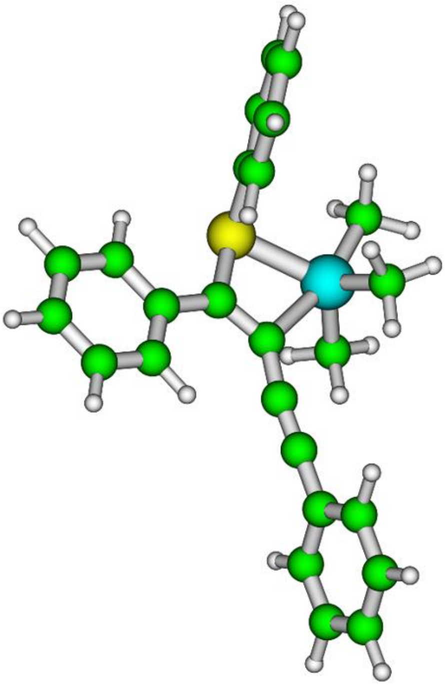

Fig. S2 Optimized transition state (at ωB97X-D/6-311+G** level of theory) of the silyl-migration (white: H, green: C, blue: Si, yellow: P).

Table S4 Relative energies of **(Z)-10a-c**’, **(E)-10a-c**’, **3a-c**’ and **2a-c**’ (at ωB97X-D/6-311+G** level of theory and in kcal/mol unit)

substitution pattern	**R1**	**R2**	**(Z)-10a-c’**	**(E)-10a-c’**	**3a-c’**	**2a-c’**
a	phenyl	phenyl	0.0	-2.3	-22.0	-41.8
b	phenyl	naphtyl	0.0	-2.0	-22.6	-41.8
b’	naphtyl	phenyl	0.0	-2.5	-21.3	-41.4
c	thienyl	naphtyl	0.0	-0.9	-22.5	-41.7
c’	naphtyl	thienyl	0.0	-2.5	-22.4	-41.4

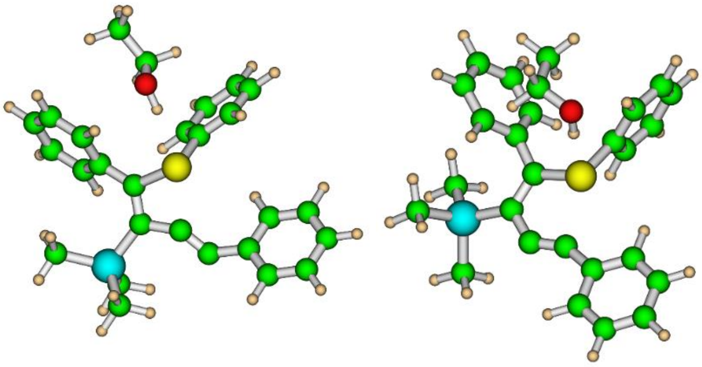

Fig. S3 Optimized transition states of the ring‘ closures (left: phosphete, right: phosphole) in the presence of one EtOH molecule (at ωB97X-D/6-311+G** level of theory, white: H, green: C, blue: Si, yellow:P)

Table S5 Relative energies of intermediates and transitions states of the reaction of **1d** with $SiMe_3PhP^-$ anion at ωB97X-D/6-311+G** level of theory and in kcal/mol unit.

substitution pattern	1d-d‘ + PhSiMe3P-	TS-1d-d‘	6 d-d‘	TS-2 d-d‘	(Z)-7 d-d‘	TS-3 d-d‘	(E)-7 d-d‘	TS-5 d-d‘	8d-d‘	TS-4 d-d‘	9d-d‘
d	0.0	12.0	-5.4	-3,7	-29.3	-5,7	-29.2	-5.7	-7.6	-8.4	-24.5
d’	0.0	7.2	-10.3	-9.1	-24.9	-10,9*	-14.0	-8.6	-14.4	2.3	-24.7

*All attempts to localise TS-3d’ fail in our hand, therefore relaxed potential energy surface scan was performed, in which the dihedral angle of (P-C-C)-Si was substantially changed. The estimated energy of the corresponding TS was obtained from the investigation of the energy values of these relaxed systems.

Table S6 Relative energies of **(Z)-10a-d'**, **(E)-10a-d'**, **3a-d'** and **2a-d'** (at ωB97X-D/6-311+G** level of theory and in kcal/mol unit).

substitution pattern	**R1**	**R2**	**(Z)-10a-d'**	**(E)-10a-d'**	**3a-d'**	**2a-d'**
d	phenyl	terc-butyl	0.0	-1.6	-21.2	-40.5
d'	terc-butyl	phenyl	0.0	4.3	-25.6	-41.1

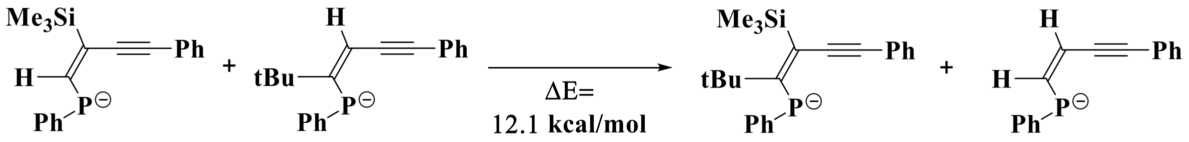

Scheme S1 Isodesmic reaction to estimate the steric destabilization of **E-7d** (ωB97X-D/6-311+G**).

Table S7 Photophysical properties of the investigated compounds (table contains both experimentally and theoretically obtained values).

Compound	Absorption Exp.[nm]	Absorption Calc.[nm]	Emission Exp.[nm]	Emission Calc. [nm]	ε_{max}*10^4	φ [-]	Oscillator strength	Transitions	Contributions [%]
**3a**	343	337	440	405	2.21	0.006	0.5461	HOMO → LUMO	90
								HOMO-1 → LUMO	6
**3b**	358	352	468	436	1.97	0.011	0.3693	HOMO → LUMO	95
								HOMO-2 → LUMO	3
**3c**	378	380	483	450	4.05	0.011	0.7782	HOMO → LUMO	97
**2b**	374	367	486	513	0.43	0.187	0.4621	HOMO → LUMO	96
**2c**	393	382	510	539	3.29	0.035	0.4183	HOMO → LUMO	98
**4b**	389	379	476	506	0.96	0.569	0.6511	HOMO → LUMO	98
**4c**	407	396	499	531	3.29	0.391	0.6375	HOMO → LUMO	99
**5a**	351	350	421	390	3.24	0.008	0.7662	HOMO → LUMO	92
								HOMO-1 → LUMO	5
**5b**	365	370	449	424	1.32	0.011	0.6042	HOMO → LUMO	96
**5c**	379	381	456	435	1.7	0.126	1.069	HOMO → LUMO	98
**2d**	307	317	440	460	0.6	0.046	0.137	HOMO → LUMO	97
**4d**	340	328	429	457	0.57	0.154	0.2962	HOMO → LUMO	97

a) Experimental procedures

All reactions were carried out by means of standard Schlenk or glovebox techniques under inert gas atmosphere (argon). Solvents were dried over Na/K alloy before use and were freshly distilled under inert gas.

Phenylphosphane ($PhPH_2$) has been prepared following literature procedure[1]. Diyne **1a** was synthesised like described in the literature.[2] The procedures for the asymmetrical diynes **1b** and **1d** have been published, too.[3] But the compounds weren't fully characterized so far.

Deuterated solvents for NMR-spectroscopy were dried and stored over molecular sieves. All chemicals were purchased from Sigma-Aldrich, ABCR or TCI and used without further purification.

For purification via column chromatography a puriFlash XS 520 plus (*Interchim*) was used. The used cartridges were filled with spheric silica gel (particle size: 15 µm or 25µm).

^{1}H-, ^{13}C-, ^{31}P-, and ^{29}Si-NMR-data were recorded on Varian VNMRS-500 MHz or MR-400 MHz spectrometers at 25°C. Chemical shifts were referenced to residual protic impurities in the solvent (^{1}H) or the deuterated solvent (^{13}C) and reported relative to external $SiMe_4$ (^{1}H, ^{13}C, ^{29}Si), H_3PO_4 (^{31}P).

APCI mass determinations were performed on a Finnigan LCQ Deca (*ThermoQuest*). Mass calibration was carried out immediately before sample measurement on sodium formate clusters or by the ESI-Tune Mix standard (*Agilent*).

Elemental analyses were performed with a HEKAtech Euro EA CHNS elemental analyser. Samples were prepared in a Sn cup and analysed with added V_2O_5.

Absorption spectra were recorded using a Shimadzu UV-1900 spectrometer in solution. Emission spectra as well as luminescence quantum yields (absolute method) were measured with the Hamamatsu C11347 system in solution. For the refinement of the data OriginPro was used.

Crystallographic measurements were carried out on a *Stoe* IPDS2 diffractometer with a STOE image plate detector and a Mo-Kα (λ = 0.71073 Å) monochromator or a *Stoe* StadiVari diffractometer with a Pilatus 200K image plate detector and Cu-Kα (λ = 1.54186 Å) radiation. Direct methods were used to

solve the measurements and refined by "least-square" cycles (SHELXL-2017).[4] All non-hydrogen atoms were anisotropically refined without restriction. The evaluation of the data sets, as well as the graphical preparation of the structures was carried out using Olex2[5] and Mercury.[6] Details of the structure determinations and refinement are summarized in Table S8–S11. The CCDC depositions 2067623–2067633 contain the supplementary crystallographic data for this paper, which can be obtained free of charge via emailing data_request@ccdc.cam.ac.uk, or by contacting The Cambridge Crystallographic Data Centre at 12 Union Road, Cambridge CB2 1EZ, UK; fax: +44 1223 336033. The molecular structure of phosphete **3c** shows a disorder within the thienyl ring (ratio: 1:1). The phenyl ring at the phosphorus atom in **3b'** disordered as well (ratio: 11:9). In phosphete **5a** the whole molecule, except the phenyl substituent at the phosphorus atom, is disordered (ratio: 1:1). All disorders were refined anisotropically. The molecular structures of diynes **1b**–**1d** are shown in Fig. S72–S74.

b) Synthetic protocols and characterization

Synthesis of diynes **1b, 1c** and **1d**

Synthesis of diyne **1b**

CuCl (38 mg, 0.38 mmol, 0.02 eq.) is dissolved in aqueous, 30% *n*BuNH$_2$ solution (47 mL) at 0 °C. The blue solution became colorless by adding a spatula tip NH$_2$OH·HCl. Then 2-ethinyltnaphthaline (3.24 g, 21.3 mmol, 1.1 eq.) was added to the reaction mixture in 7 mL THF. After 5 min bromethinylbenzene (3.50 g, 19.4 mmol, 1.0 eq.) was added to the reaction mixture in 7 mL THF. The remaining NH$_2$OH·HCl (total 888 mg, 12.8 mmol, 0.66 eq.) was then added over a period of 45 min. After 70 min, the reaction was stopped by addition of saturated NH$_4$Cl-Lsg. The crude product was obtained as brown oil after extraction with dichloromethane (3 x 30 mL) followed by drying over MgSO$_4$. After purification by column chromatography (*n*hexane), the product (R$_F$ = 0.3) was obtained as colouless solid.

Yield: 23% (1.12 g).

^{1}H-NMR (399.87 MHz, $CDCl_3$) $\delta = 8.08$ (br. s, 1 H, CH_{Ar}), 7.84–7.79 (m, 3 H, CH_{Ar}), 7.57–7.50 (m, 5 H, 5 x CH_{Ar}), 7.41–7.33 (m, 3 H, 3 x CH_{Ar}).

^{13}C{^{1}H}-NMR (100.56 MHz, $CDCl_3$) $\delta = 133.3$ (s, C_{Ar}), 133.2 (s, C_{Ar}), 133.0 (s, C_{Ar}), 132.7 (s, C_{Ar}), 129.4 (s, C_{Ar}), 128.6 (s, C_{Ar}), 128.6 (s, C_{Ar}), 128.3 (s, C_{Ar}), 128.0 (s, C_{Ar}), 128.0 (s, C_{Ar}), 127.4 (s, C_{Ar}), 127.0 (s, C_{Ar}), 122.0 (s, C_{Ar}), 119.2 (s, C_{Ar}), 82.2 (s, C_{Alkyne}), 81.9 (s, C_{Alkyne}), 74.4 (s, C_{Alkyne}), 74.2 (s, C_{Alkyne}).

Elemental Analysis (%): Calculated: C 95.21, H 4.79 found: C 95.09, H 4.94.

MS (APCI-HR) m/z: 253.10118. [**1b**+H]$^+$ calculated: 253.10118.

Synthesis of diyne **1c**

CuCl (33 mg, 0.33 mmol, 0.02 eq.) is dissolved in aqueous, 30% *n*$BuNH_2$ solution (42 mL) at 0 °C. The blue solution became colorless by adding a spatula tip $NH_2OH \cdot HCl$. Then 2-ethynylthiophene (1.94 g, 17.9 mmol, 1.1 eq.) was added to the reaction mixture in 6 mL THF. After 5 min 2-(bromoethinyl)naphtaline (3.77 g, 16.3 mmol, 1.0 eq.) was added to the reaction mixture in 6 mL THF. The remaining $NH_2OH \cdot HCl$ (total 747 mg, 10.8 mmol, 0.66 eq.) was then added over a period of 45 min. After 70 min, the reaction was stopped by addition of saturated NH_4Cl-Lsg. The crude product was obtained as brown oil after extraction with dichloromethane (3 x 30 mL) followed by drying over $MgSO_4$. After purification by column chromatography with *n*pentane/DCM 10:1, the product ($R_F = 0.3$) was obtained as light yellow solid.

Yield: 81% (3.41 g).

^{1}H-NMR (399.87 MHz, $CDCl_3$) $\delta = 8.07$ (br. s, 1 H, CH_{Ar}), 7.84–7.79 (m, 3 H, 3 x CH_{Ar}), 7.55–7.50 (m, 3 H, 3 x CH_{Ar}), 7.39–7.32 (m, 2 H, 2 x CH_{Ar}), 7.03–7.01 (m, 1 H, CH_{Ar}).

^{13}C{^{1}H}-NMR (100.56 MHz, $CDCl_3$) δ = 134.5 (s, C_{Ar}), 133.3 (s, C_{Ar}), 133.2 (s, C_{Ar}), 132.9 (s, C_{Ar}), 128.9 (s, C_{Ar}), 128.5 (s, C_{Ar}), 128.3 (s, C_{Ar}), 128.0 (s, C_{Ar}), 128.0 (s, C_{Ar}), 127.4 (s, C_{Ar}), 127.3 (s, C_{Ar}), 126.9 (s, C_{Ar}), 122.2 (s, C_{Ar}), 119.0 (s, C_{Ar}), 84.3 (s, C_{Alkyne}), 78.3 (s, C_{Alkyne}), 74.9 (s, C_{Alkyne}), 74.2 (s, C_{Alkyne}).

Elemental Analysis (%): Calculated: C 83.69, H 3.90, found: C 83.95, H 3.83.

MS (APCI-HR) m/z: 259.05760. [**1c**+H]$^+$ calculated: 259.05760.

Synthesis of diyne **1d**

CuCl (96 mg, 0.97 mmol, 0.02 eq.) is dissolved in aqueous, 30% *n*$BuNH_2$ solution (133 mL) at 0 °C. The blue solution became colorless by adding a spatula tip $NH_2OH{\cdot}HCl$. Then 3,3-dimethylbut-1-yne (4.00 g, 48.7 mmol, 1.0 eq.) was added to the reaction mixture in 6 mL THF. After 5 min bromethinylbenzene (9.70 g, 53.6 mmol, 1.1 eq.) was added to the reaction mixture in 6 mL THF. The remaining $NH_2OH{\cdot}HCl$ (total 2.23 g, 32.0 mmol, 0.66 eq.) was then added over a period of 45 min. After 18 h, the reaction was stopped by addition of saturated NH_4Cl-Lsg. The crude product was obtained as brown oil after extraction with dichloromethane (3 x 50 mL) followed by drying over $MgSO_4$. After purification by column chromatography (*n*hexane), the product (R_F = 0.3) was obtained as colouless solid.

Yield: 41% (3.60 g).

^{1}H-NMR (399.87 MHz, $CDCl_3$) δ = 7.46–7.46 (m, 2 H, 2 x CH_{Ar}), 7.36–7.27 (m, 3 H, 3 x CH_{Ar}), 1.29 (s, 9 H, 3 x CH_3).

^{13}C{^{1}H}-NMR (100.56 MHz, $CDCl_3$) δ = 132.6 (s, C_{Ar}), 128.9 (s, C_{Ar}), 128.5 (s, C_{Ar}), 122.3 (s, C_{Ar}), 92.4 (s, C_{Alkyne}), 76.1 (s, C_{Alkyne}), 74.3 (s, C_{Alkyne}), 63.9 (s, C_{Alkyne}), 30.7 (s, CCH_3), 28.5 (s, CCH$_3$).

Elemental Analysis (%): Calculated: C 92.26, H 7.74, found: C 92.52, H 8.03.

MS (APCI-HR) m/z: 365.22638. [2 **1d**+H]$^+$ calculated: 365.22638.

Synthesis of the phosphetes **3a, 3b, 3b', 3c**

Synthesis of phosphete **3a**

A Schlenk tube was charged with DME (20 mL) and cooled to –60 °C. Then, phenylphosphane (0.11 g, 1 mmol, 1.0 eq.) was added. After that, *n*BuLi (2.5 M, 0.4 mL, 1 mmol, 1 eq.) was used for lithiation. The orange solution was stirred for 40 min and quenched with trimethylchlorosilane (0.13 mL, 1 mmol, 1eq.). The colourless solution was lithiated with *n*BuLi (2.5 M, 0.4 mL, 1 mmol, 1 eq.) again. After further 75 min, the reaction mixture was cooled to –80 °C and 1,4-diphenylbuta-1,3-diyne (0.20 g, 1 mmol, 1.0 eq.), dissolved in DME (10 mL), was added dropwise to the orange solution. The colour changed to deep blue and after 55 min the reaction was terminated by adding ethanol. Afterwards, the solvent was removed, and the crude product was purified by silica gel column chromatography with *n*pentane/dichloromethane 10:1 (R_F = 0.2). The product was obtained as colourless solid.

Yield: 0.09 g (21%).

^{1}H-NMR (399.87 MHz, CD_2Cl_2) δ = 7.65–7.61 (m, 2 H, 2 x CH_{Ar}), 7.42–7.22 (m, 12 H, 12f x CH_{Ar}), 7.17–7.13 (m, 1 H, CH_{Ar}),6.88 (d, $^3J(^1H-^{31}P)$ = 20.4 Hz, 1 H, PhC*H*), 0.37 (s, 9 H, 3 x SiCH_3).

^{13}C{^{1}H}-NMR (100.56 MHz, CD_2Cl_2) δ = 167.3 (s, C_{Ar}), 159.2 (d, $J(^{13}C-^{31}P)$ = 9.2 Hz, C_{Ar}), 143.0 (d, $J(^{13}C-^{31}P)$ = 4.1 Hz, C_{Ar}), 138.0 (s, C_{Ar}), 137.0 (d, $J(^{13}C-^{31}P)$ = 12.4 Hz, C_{Ar}), 136.0 (d, $J(^{13}C-^{31}P)$ = 33.3 Hz, C_{Ar}), 133.4 (d, $J(^{13}C-^{31}P)$ = 18.2 Hz, C_{Ar}), 130.5 (d, $J(^{13}C-^{31}P)$ = 1.3 Hz, C_{Ar}), 129.0 (d, $J(^{13}C-^{31}P)$ = 6.7 Hz, C_{Ar}), 128.9 (s, C_{Ar}), 128.8 (d, $J(^{13}C-^{31}P)$ = 0.6 Hz, C_{Ar}), 128.7 (s, C_{Ar}), 128.2 (d, $J(^{13}C-^{31}P)$ = 5.5 Hz, C_{Ar}), 127.9 (d, $J(^{13}C-^{31}P)$ = 6.1 Hz, C_{Ar}), 127.7 (s, C_{Ar}), 123.8 (d, $J(^{13}C-^{31}P)$ = 5.2 Hz, C_{Ar}), 0.7 (s, SiCH$_3$).

^{29}Si{^{1}H}-NMR (99.28 MHz, CD_2Cl_2) δ = –13.2 (d, $^3J(^{29}Si-^{31}P)$ = 5.5 Hz).

^{31}P-NMR (202.30 MHz, CD_2Cl_2) δ = 32.1 (dt, $^3J(^{31}P-^1H)$ = 20.6 Hz, $^3J(^{31}P-^1H)$ = 7.6 Hz).

Elemental Analysis (%): calculated for $C_{25}H_{25}PSi$: C 78.09, H 6.55, found: C 78.02, H 6.61.

MS (APCI-HR) m/z: found: 385.15359. [**3a**+H]$^+$, calculated: 385.15359.

Synthesis of phosphete **3b** and **3b’**

A Schlenk tube was charged with DME (20 mL) and cooled to –50 °C. Then, phenylphosphane (0.11 g, 1 mmol, 1.0 eq.) was added. After that, *n*BuLi (2.5 M, 0.4 mL, 1 mmol, 1 eq.) was used for lithiation. The orange solution was stirred for 45 min and quenched with trimethylchlorosilane (0.13 mL, 1 mmol, 1eq.). The colourless solution was lithiated with *n*BuLi (2.5 M, 0.4 mL, 1 mmol, 1 eq.) again. After further 45 min, the reaction mixture was cooled to –80 °C and 2-(phenylbuta-1,3-diyn-1-yl)naphtaline (0.25 g, 1 mmol, 1 eq.), dissolved in DME (10 mL), was added dropwise to the orange solution. The colour changed to black blue and after 60 min the reaction was terminated by adding ethanol resulting in a reddish solution. Afterwards, the solvent was removed and the crude product was purified by silica gel column chromatography with *n*pentane/dichloromethane 5:1 (R_F = 0.5). The product was obtained as slightly yellow solid.

Yield: 24% (0.11 g)

^{1}H-NMR (399.87 MHz, CD_2Cl_2) δ = 7.88–7.17 (m, 17 H, 17 x CH_{Ar}), 7.06 (d, $^{3}J(^{1}H\text{-}^{31}P)$ = 20.2 Hz 0.55 H, TMSCC*H*), 6.94 (d, $^{3}J(^{1}H\text{-}^{31}P)$ = 10.2 Hz 0.45 H, TMSCC*H*), 0.42 (s, 9 H, 3 x Si(CH_3)).

^{13}C{^{1}H}-NMR (100.56 MHz, CD_2Cl_2) δ = 167.4 (s, C_{Ar}), 167.3 (s, C_{Ar}), 159.5 (s, C_{Ar}), 159.4 (s, C_{Ar}), 159.3 (s, C_{Ar}), 142.2 (d, $J(^{13}C\text{-}^{31}P)$ = 3.8 Hz, C_{Ar}), 141.6 (d, $J(^{13}C\text{-}^{31}P)$ = 3.8 Hz, C_{Ar}), 138.1 (s, C_{Ar}), 137.0 (d, $J(^{13}C\text{-}^{31}P)$ = 12.4 Hz, C_{Ar}), 136.2 (d, $J(^{13}C\text{-}^{31}P)$ = 3.8 Hz, C_{Ar}), 135.8 (d, $J(^{13}C\text{-}^{31}P)$ = 3.8 Hz, C_{Ar}), 135.8 (s, C_{Ar}), 134.6 (d, $J(^{13}C\text{-}^{31}P)$ = 12.4 Hz, C_{Ar}), 134.1 (s, C_{Ar}), 133.7 (s, C_{Ar}), 133.6 (s, C_{Ar}), 133.5 (d, $J(^{13}C\text{-}^{31}P)$ = 7.2 Hz, C_{Ar}), 133.3 (s, C_{Ar}), 133.2 (s, C_{Ar}), 130.5 (d, $J(^{13}C\text{-}^{31}P)$ = 1.3 Hz, C_{Ar}), 130.4 (d, $J(^{13}C\text{-}^{31}P)$ = 1.3 Hz, C_{Ar}), 129.1 (d, $J(^{13}C\text{-}^{31}P)$ = 3.3 Hz, C_{Ar}), 129.0 (d, $J(^{13}C\text{-}^{31}P)$ = 3.1 Hz, C_{Ar}), 128.9 (s, C_{Ar}), 128.9 (s, C_{Ar}), 128.8 (s, C_{Ar}), 128.7 (s, C_{Ar}), 128.4 (d, $J(^{13}C\text{-}^{31}P)$ = 4.3 Hz, C_{Ar}), 128.3 (s, C_{Ar}), 128.2 (s, C_{Ar}), 128.1 (d, $J(^{13}C\text{-}^{31}P)$ = 9.1 Hz, C_{Ar}), 128.0 (d, $J(^{13}C\text{-}^{31}P)$ = 5.9 Hz, C_{Ar}), 127.7 (s, C_{Ar}), 127.3 (s, C_{Ar}), 127.3 (d, $J(^{13}C\text{-}^{31}P)$ = 3.1 Hz, C_{Ar}), 127.0 (d, $J(^{13}C\text{-}^{31}P)$ = 4.1 Hz, C_{Ar}), 126.7 (s, C_{Ar}), 126.4 (s, C_{Ar}), 126.2 (d, $J(^{13}C\text{-}^{31}P)$ = 5.6 Hz, C_{Ar}), 125.6 (d, $J(^{13}C\text{-}^{31}P)$ = 8.5 Hz, C_{Ar}),

123.9 (d, $J(^{13}C\text{-}^{31}P)$ = 5.3 Hz, C_{Ar}), 123.8 (d, $J(^{13}C\text{-}^{31}P)$ = 5.2 Hz, C_{Ar}), 0.8 (s, $Si(CH_3)_3$), 0.7 (d, $J(^{13}C\text{-}^{31}P)$ = 0.7 Hz, $Si(CH_3)_3$).

^{29}Si{^{1}H}-NMR (99.28 MHz, CD_2Cl_2) δ = –13.2 (d, $^{3}J(^{29}Si\text{-}^{31}P)$ = 5.4 Hz).

^{31}P-NMR (202.30 MHz, CD_2Cl_2) δ = 32.1 (s), 31.5 (s).

Elemental Analysis (%): calculated for $C_{29}H_{27}PSi$+3/4H_2O: C 77.73, H 6.41, found: C 77.52, H 6.63.

MS (APCI-HR) m/z: found: 435.16924 [**3b**/**3b'**+H]$^+$, calculated: 435.16924.

Synthesis of phosphete **3c**

A Schlenk tube was charged with DME (20 mL) and cooled to –60 °C. Then, phenylphosphane (0.11 g, 1 mmol, 1.0 eq.) was added. After that, *n*BuLi (2.5 M, 0.4 mL, 1 mmol, 1 eq.) was used for lithiation. The orange solution was stirred for 40 min and quenched with trimethylchlorosilane (0.13 mL, 1 mmol, 1eq.). The colourless solution was litfhiated with *n*BuLi (2.5 M, 0.4 mL, 1 mmol, 1 eq.) again. After further 75 min, the reaction mixture was cooled to –80 °C and 2-(naphtalin-2-ylbuta-1,3-diyn-1-yl)thiophene (0.26 g, 1 mmol, 1.0 eq.), dissolved in DME (10 mL), was added dropwise to the orange solution. The colour changed to black blue and after 55 min the reaction was terminated by adding ethanol. Afterwards, the solvent was removed and the crude product was purified by silica gel column chromatography with *n*pentane/dichloromethane 10:1 (R_F = 0.2). The product was obtained as slightly yellow solid.

Yield: 41% (0.20 g)

^{1}H-NMR (399.87 MHz, CD_2Cl_2) δ = 7.81–7.74 (m, 5 H, 5 x CH_{Ar}), 7.723–7.60 (m, 2 H, 2 x CH_{Ar}), 7.49–7.31 (m, 6 H, 6 x CH_{Ar}), 7.15–7.10 (m, 2 H, 2 x CH_{Ar}), 7.03–7.01 (m, 1 H, *H*C-Naph), 0.56 (s, 9 H, 3 x $SiCH_3$).

^{13}C{^{1}H}-NMR (100.56 MHz, CD_2Cl_2) δ = 158.1 (s, C_{Ar}), 158.0 (s, C_{Ar}), 143.0 (d, $J(^{13}C\text{-}^{31}P)$ = 4.8 Hz, C_{Ar}), 140.1 (d, $J(^{13}C\text{-}^{31}P)$ = 15.2 Hz, C_{Ar}), 136.1 (s, C_{Ar}), 136.0 (s, C_{Ar}), 135.8 (s, C_{Ar}), 134.3 (s, C_{Ar}), 133.8 (d, $J(^{13}C\text{-}^{31}P)$ = 18.7 Hz, C_{Ar}), 133.4 (s, C_{Ar}), 131.0 (d, $J(^{13}C\text{-}^{31}P)$ = 1.0 Hz, C_{Ar}), 129.4 (d,

$J(^{13}C-^{31}P)$ = 7.0 Hz, C_{Ar}), 128.7 (s, C_{Ar}), 128.6 (s, C_{Ar}), 128.6 (s, C_{Ar}), 128.6 (s, C_{Ar}), 128.3 (s, C_{Ar}), 127.5 (d, $J(^{13}C-^{31}P)$ = 4.0 Hz, C_{Ar}), 127.0 (s, C_{Ar}), 126.6 (s, C_{Ar}), 125.8 (d, $J(^{13}C-^{31}P)$ = 8.5 Hz, C_{Ar}), 124.4 (d, $J(^{13}C-^{31}P)$ = 5.3 Hz, C_{Ar}), 0.8 (s, SiCH_3).

^{29}Si{^{1}H}-NMR (99.28 MHz, CD_2Cl_2) δ = –13.3 (d, $^3J(^{29}Si-^{31}P)$ = 5.3 Hz).

^{31}P-NMR (202.30 MHz, CD_2Cl_2) δ = 37.4 (s).

Elemental Analysis (%): calculated for $C_{27}H_{25}PSSi$: C 73.60, H 5.72, found: C 73.82, H 5.88.

MS (APCI-HR) m/z: found: 441.12566 [**3c**+H]$^+$, calculated: 441.12566.

Synthesis of the β-silyl phospholes **2b, 2b’, 2c, 2c’**

Synthesis of phosphole **2b** and **2b’**

A Schlenk tube was charged with DME (20 mL) and cooled to –50 °C. Then, phenylphosphane (0.11 g, 1 mmol, 1.0 eq.) was added. After that, *n*BuLi (2.5 M, 0.4 mL, 1 mmol, 1.0 eq.) was used for lithiation. The orange solution was stirred for 45 min and quenched with trimethylchlorosilane (0.13 mL, 1 mmol, 1eq.). The colourless solution was lithiated with *n*BuLi (2.5 M, 0.4 mL, 1.1 mmol, 1 eq.) again. After further 45 min, the reaction mixture was cooled to –80 °C and 2-(phenylbuta-1,3-diyn-1-yl)naphtaline (0.25 g, 1 mmol, 1 eq.), dissolved in DME (10 mL), was added dropwise to the orange solution. The colour changed to deep blue and after 60 min the reaction was terminated by adding ethanol resulting in a reddish solution. Afterwards, the solvent was removed and the crude product was purified by silica gel column chromatography with *n*pentane/dichloromethane 5:1 (R_F = 0.6). The product was obtained as yellow wax.

Yield: 24% (0.11 g).

Isomer 1

^{1}H-NMR (399.87 MHz, CD_2Cl_2) δ = 7.99 (br. s, 1 H, CH_{Ar}), 7.82–7.75 (m, 4 H, 4 x CH_{Ar}), 7.61 (m, 1 H, TMSCC*H*), 7.51–7.37 (m, 3 H, 3 x CH_{Ar}), 7.31–7.14 (m, 9 H, 9 x CH_{Ar}), 0.18 (s, 9 H, 3 x Si(CH_3)).

^{13}C{^{1}H}-NMR (100.56 MHz, CD_2Cl_2) δ = 164.2 (d, $J(^{13}C\text{-}^{31}P)$ = 5.1 Hz, C_{Ar}), 150.7 (d, $J(^{13}C\text{-}^{31}P)$ = 2.7 Hz, C_{Ar}), 148.4 (d, $J(^{13}C\text{-}^{31}P)$ = 5.2 Hz, C_{Ar}), 139.5 (d, $J(^{13}C\text{-}^{31}P)$ = 19.6 Hz, C_{Ar}), 138.9 (d, $J(^{13}C\text{-}^{31}P)$ = 13.4 Hz, C_{Ar}), 134.8 (d, $J(^{13}C\text{-}^{31}P)$ = 17.1 Hz, C_{Ar}), 134.2 (d, $J(^{13}C\text{-}^{31}P)$ = 18.7 Hz, C_{Ar}), 133.2 (s, C_{Ar}), 130.0 (d, $J(^{13}C\text{-}^{31}P)$ = 1.9 Hz, C_{Ar}), 129.7 (d, $J(^{13}C\text{-}^{31}P)$ = 7.6 Hz, C_{Ar}), 129.2 (d, $J(^{13}C\text{-}^{31}P)$ = 8.4 Hz, C_{Ar}), 129.3 (s, C_{Ar}), 128.8 (s, C_{Ar}), 128.5 (s, C_{Ar}), 128.4 (s, C_{Ar}), 128.1 (s, C_{Ar}), 127.7 (d, $J(^{13}C\text{-}^{31}P)$ = 1.4 Hz, C_{Ar}), 126.9 (s, C_{Ar}), 126.2 (s, C_{Ar}), 125.6 (d, $J(^{13}C\text{-}^{31}P)$ = 10.2 Hz, C_{Ar}), 125.4 (d, $J(^{13}C\text{-}^{31}P)$ = 8.1 Hz, C_{Ar}), 0.6 (d, $J(^{13}C\text{-}^{31}P)$ = 0.9 Hz, $Si(CH_3)_3$).

^{29}Si{^{1}H}-NMR (99.28 MHz, CD_2Cl_2) δ = –7.6 (d, $^{3}J(^{29}Si\text{-}^{31}P)$ = 3.9 Hz).

^{31}P-NMR (202.30 MHz, CD_2Cl_2) δ = 22.6 (s).

Isomer 2

^{1}H-NMR (399.87 MHz, CD_2Cl_2) δ = 0.17 (s, 9 H, 3 x $Si(CH_3)$).

^{13}C{^{1}H}-NMR (100.56 MHz, CD_2Cl_2) δ = 164.0 (d, $J(^{13}C\text{-}^{31}P)$ = 5.0 Hz, C_{Ar}), 150.0 (d, $J(^{13}C\text{-}^{31}P)$ = 2.4 Hz, C_{Ar}), 148.6 (d, $J(^{13}C\text{-}^{31}P)$ = 5.4 Hz, C_{Ar}), 139.0 (s, C_{Ar}), 138.6 (d, $J(^{13}C\text{-}^{31}P)$ = 13.1 Hz, C_{Ar}), 137.2 (s, C_{Ar}), 137.0 (s, C_{Ar}), 134.2 (d, $J(^{13}C\text{-}^{31}P)$ = 20.1 Hz, C_{Ar}), 131.4 (d, $J(^{13}C\text{-}^{31}P)$ = 10.1 Hz, C_{Ar}), 129.0 (s, C_{Ar}), 128.9 (d, $J(^{13}C\text{-}^{31}P)$ = 7.6 Hz, C_{Ar}), 128.5 (s, C_{Ar}), 128.4 (s, C_{Ar}), 128.4 (s, C_{Ar}), 128.2 (s, C_{Ar}), 128.0 (d, $J(^{13}C\text{-}^{31}P)$ = 8.2 Hz, C_{Ar}), 127.9 (d, $J(^{13}C\text{-}^{31}P)$ = 5.8 Hz, C_{Ar}), 127.8 (s, C_{Ar}), 127.6 (s, C_{Ar}), 127.0 (d, $J(^{13}C\text{-}^{31}P)$ = 9.1 Hz, C_{Ar}), 126.8 (s, C_{Ar}),

0.7 (d, $J(^{13}C\text{-}^{31}P)$ = 1.3 Hz, $Si(CH_3)_3$).

^{29}Si{^{1}H}-NMR (99.28 MHz, CD_2Cl_2) δ = –7.6 (d, $^{3}J(^{29}Si\text{-}^{31}P)$ = 3.9 Hz).

^{31}P-NMR (202.30 MHz, CD_2Cl_2) δ = 22.4 (s).

Elemental Analysis (%): calculated for $C_{29}H_{27}PSi$: C 80.15, H 6.26, found C 80.33, H 6.58

MS (APCI-HR) m/z: found: 435.16924 [**2b**/ **2b'**+H]$^{+}$, calculated: 435.16924.

Synthesis of phosphole **2c** and **2c'**

A Schlenk tube was charged with DME (20 mL) and cooled to –60 °C. Then, phenylphosphane (0.11 g, 1 mmol, 1.0 eq.) was added. After that, *n*BuLi (2.5 M, 0.4 mL, 1 mmol, 1.0 eq.) was used for lithiation. The orange solution was stirred for 30 min and quenched with trimethylchlorosilane (0.13 mL, 1 mmol, 1eq.). The colourless solution was lithiated with *n*BuLi (2.5 M, 0.4 mL, 1.1 mmol, 1 eq.) again. After further 60 min, the reaction mixture was cooled to –80 °C and 1,4-2-(naphtalin-2-ylbuta-1,3-diyn-1-yl)thiophene (0.20 g, 1 mmol, 1.0 eq.), dissolved in DME (10 mL), was added dropwise to the orange solution. The colour changed to deep blue and after 30 min the reaction was terminated by adding ethanol. Afterwards, the solvent was removed, and the crude product was purified by silica gel column chromatography with cyclohexane/ethyl acetate 20:1 (R_F = 0.3). The product was obtained as yellow wax.

Yield: 39% (0.17 g).

^{1}H-NMR (399.87 MHz, CD_2Cl_2) δ = 8.02 (br. s, 1 H, CH_{Ar}), 7.83–7.80 (m, 4 H, 4 x CH_{Ar}), 7.63 (d, $^{3}J(^{1}H\text{-}^{31}P)$ = 10.6 Hz, 1 H, TMSCC*H*), 7.52–7.35 (m, 4 H, 4 x CH_{Ar}), 7.30–7.18 (m, 4 H, 4 x CH_{Ar}), 7.09–6.96 (m, 2 H, 2 x CH_{Ar}), 0.34 (s, 9 H, 3 x Si(CH_3)).

^{13}C{^{1}H}-NMR (100.56 MHz, CD_2Cl_2) δ = 163.0 (s, C_{Ar}), 155.2 (d, $J(^{13}C\text{-}^{31}P)$ = 3.7 Hz, C_{Ar}), 150.9 (d, $J(^{13}C\text{-}^{31}P)$ = 2.4 Hz, C_{Ar}), 149.7 (d, $J(^{13}C\text{-}^{31}P)$ = 2.4 Hz, C_{Ar}), 141.0 (d, $J(^{13}C\text{-}^{31}P)$ = 23.8 Hz, C_{Ar}), 139.3 (d, $J(^{13}C\text{-}^{31}P)$ = 11.8 Hz, C_{Ar}), 134.4 (s, C_{Ar}), 134.2 (d, $J(^{13}C\text{-}^{31}P)$ = 0.9 Hz, C_{Ar}), 133.2 (d, $J(^{13}C\text{-}^{31}P)$ = 0.6 Hz, C_{Ar}), 131.6 (d, $J(^{13}C\text{-}^{31}P)$ = 9.8 Hz, C_{Ar}), 130.2 (d, $J(^{13}C\text{-}^{31}P)$ = 1.6 Hz, C_{Ar}), 129.3 (s, C_{Ar}), 129.2 (s, C_{Ar}), 128.8 (d, $J(^{13}C\text{-}^{31}P)$ = 1.0 Hz, C_{Ar}), 128.5 (s, C_{Ar}), 128.1 (s, C_{Ar}), 128.0 (s, C_{Ar}), 127.0 (d, $J(^{13}C\text{-}^{31}P)$ = 6.7 Hz, C_{Ar}), 126.9 (s, C_{Ar}), 126.3 s, C_{Ar}), 125.7 (d, $J(^{13}C\text{-}^{31}P)$ = 10.4 Hz, C_{Ar}), 125.3 (d, $J(^{13}C\text{-}^{31}P)$ = 8.2 Hz, C_{Ar}), 0.6 (d, $J(^{13}C\text{-}^{31}P)$ = 1.4 Hz, Si(CH_3)$_3$).

^{29}Si{^{1}H}-NMR (99.28 MHz, CD_2Cl_2) δ = –7.6 (d, $^{3}J(^{29}Si\text{-}^{31}P)$ = 3.2 Hz).

^{31}P-NMR (202.30 MHz, CD_2Cl_2) δ = 24.7 (s).

Isomer 2

^{1}H-NMR (399.87 MHz, CD_2Cl_2) δ = 0.21 (s, 9 H, 3 x Si(CH_3)).

^{13}C{^{1}H}-NMR (100.56 MHz, CD_2Cl_2) δ = 162.9 (s, C_{Ar}), 149.0 (d, $J(^{13}C\text{-}^{31}P)$ = 4.3 Hz, C_{Ar}), 144.4 (d, $J(^{13}C\text{-}^{31}P)$ = 1.7 Hz, C_{Ar}), 141.0 (d, $J(^{13}C\text{-}^{31}P)$ = 21.2 Hz, C_{Ar}), 138.2 (d, $J(^{13}C\text{-}^{31}P)$ = 12.8 Hz, C_{Ar}), 136.8 (d, $J(^{13}C\text{-}^{31}P)$ = 19.9 Hz, C_{Ar}), 134.7 (s, C_{Ar}), 134.5 (d, $J(^{13}C\text{-}^{31}P)$ = 2.8 Hz, C_{Ar}), 134.3 (d, $J(^{13}C\text{-}^{31}P)$ = 2.6 Hz, C_{Ar}), 133.6 (s, C_{Ar}), 133.1 (d, $J(^{13}C\text{-}^{31}P)$ = 0.6 Hz, C_{Ar}), 131.4 (d, $J(^{13}C\text{-}^{31}P)$ = 10.4 Hz, C_{Ar}), 130.3 (d, $J(^{13}C\text{-}^{31}P)$ = 2.0 Hz, C_{Ar}), 128.4 (s, C_{Ar}), 128.3 (d, $J(^{13}C\text{-}^{31}P)$ = 8.0 Hz, C_{Ar}), 128.2 (s, C_{Ar}), 128.1 (s, C_{Ar}), 127.9 (s, C_{Ar}), 126.9 (s, C_{Ar}), 126.4 (d, $J(^{13}C\text{-}^{31}P)$ = 1.9 Hz, C_{Ar}), 124.8 (d, $J(^{13}C\text{-}^{31}P)$ = 6.7 Hz, C_{Ar}), 124.8 (d, $J(^{13}C\text{-}^{31}P)$ = 1.4 Hz, C_{Ar}), 0.7 (d, $J(^{13}C\text{-}^{31}P)$ = 0.9 Hz, Si(CH_3)$_3$).

^{31}P-NMR (202.30 MHz, CD_2Cl_2) δ = 24.5 (s).

MS (APCI-HR) m/z: found: 441.12566 [**2c**/**2c'**+H]$^+$, calculated: 441.12566.

Elemental Analysis (%): calculated for $C_{27}H_{25}PSSi$: C 73.60, H 5.72, found: C 73.66, H 5.72.

Synthesis of the phospholes **4b, 4c**

Synthesis of phosphole **4b**

Method A

A Schlenk tube was charged with THF (20 mL) and cooled to –50 °C. Then, phenylphosphane (0.11 g, 1.0 mmol, 1 eq.) was added. After that, *n*BuLi (2.5 M, 0.5 mL, 1.0 mmol, 1. eq.) was used for lithiation. The orange solution was stirred for 90 min. Then, 2-(phenylbuta-1,3-diyn-1-yl)naphtaline (0.25 g, 1.0 mmol, 1 eq.), dissolved in THF (10 mL), was added dropwise to the orange solution. The colour changed to deep black blue and after 60 min the reaction was terminated by adding ethanol resulting in a reddish solution. Afterwards, the solvent was removed and the crude product was purified by silica gel column chromatography with *n*hexane/dichloromethane 10:1 (R_F = 0.1). The product was obtained as yellow solid.

Yield: 35% (0.12 g).

Method B

Phosphole **2b/2b'** (43 mg, 1 eq. 0.19 mmol) was dissolved in DMSO (0.6 mL) and KOTMS (3 mg, 0.02 mmol, 0.2 eq.) was added. The colour of the reaction mixture changed to brown. After 2 d at rt the reaction was terminated by addition of water and the crude product was purified by column chromatography as mentioned in method A.

Yield: 22% (8 mg).

^{1}H-NMR (399.87 MHz, CD_2Cl_2) δ = 8.00 (br. s, 1 H, CH_{Ar}), 7.80–7.72 (m, 4 H, 4 x CH_{Ar}), 7.61–7.59 (m, 2 H, 2 x PCC*H*), 7.49–7.37 (m, 6 H, 6 x CH_{Ar}), 7.33–7.29 (m, 2 H, 2 x CH_{Ar}), 7.23–7.18 (m, 3 H, 3 x CH_{Ar}).

^{13}C{^{1}H}-NMR (100.56 MHz, CD_2Cl_2) δ = 152.4 (d, J(^{13}C-^{31}P) = 16.1 Hz, C_{Ar}), 136.9 (d, J(^{13}C-^{31}P) = 16.9 Hz, C_{Ar}), 134.4 (d, J(^{13}C-^{31}P) = 19.6 Hz, C_{Ar}), 134.4 (s, C_{Ar}), 134.3 (s, C_{Ar}), 134.2 (s, C_{Ar}), 133.3 (d, J(^{13}C-^{31}P) = 8.7 Hz, C_{Ar}), 132.9 (d, J(^{13}C-^{31}P) = 8.9 Hz, C_{Ar}), 131.5 (d, J(^{13}C-^{31}P) = 8.3 Hz, C_{Ar}), 130.4 (s, C_{Ar}), 130.4 (s, C_{Ar}), 129.4 (d, J(^{13}C-^{31}P) = 8.6 Hz, C_{Ar}), 129.3 (s, C_{Ar}), 128.8 (s, C_{Ar}), 128.5 (s, C_{Ar}), 128.1 (s, C_{Ar}), 127.9 (s, C_{Ar}), 126.9 (d, J(^{13}C-^{31}P) = 3.0 Hz, C_{Ar}), 126.9 (s, C_{Ar}), 126.4 (s, C_{Ar}), 125.6 (d, J(^{13}C-^{31}P) = 11.3 Hz, C_{Ar}), 125.1 (d, J(^{13}C-^{31}P) = 8.5 Hz, C_{Ar}).

^{31}P-NMR (202.30 MHz, CD_2Cl_2) δ = 2.2 (s).

Elemental Analysis (%): calculated for $C_{26}H_{19}P$: C 86.17, H 5.28, found: C 86.45, H 5.36.

MS (APCI-HR) m/z: found: 365.18589 [**4b** +H]$^{+}$, calculated: 365.18589.

Synthesis of phosphole **4c**

Method A

A Schlenk tube was charged with THF (20 mL) and cooled to –60 °C. Then, phenylphosphane (0.12 g, 1.1 mmol, 1 eq.) was added. After that, *n*BuLi (2.5 M, 0.5 mL, 1.2 mmol, 1.05 eq.) was used for lithiation. The orange solution was stirred for 90 min. Then, 2-(naphtalin-2-ylbuta-1,3-diyn-1-yl)thiophene (0.28 g, 1.1 mmol, 1 eq), dissolved in THF (10 mL), was added dropwise to the orange solution at –30 °C. The colour changed to deep black blue and after 40 min the reaction was terminated by adding ethanol resulting in a reddish solution. Afterwards, the solvent was removed and the crude

product was purified by silica gel column chromatography with *n*pentane/dichloromethane 10:1 (R_F = 0.2). The product was obtained as yellow solid.

Yield: 34% (0.14 g).

Method B

Phosphole **2c/2c'** (200 mg, 1 eq. 0.23 mmol) was dissolved in DMSO (2.5 mL) and KOTMS (6 mg, 0.05 mmol, 0.2 eq.) was added. The colour of the reaction mixture changed to deep red. After 16 h min at 60 °C the reaction was terminated by addition of water and the crude product was purified by column chromatography as mentioned in method A.

Yield: 20% (16 mg).

^{1}H-NMR (399.87 MHz, CD_2Cl_2) δ = 7.95 (br. s, 1 H, CH_{Ar}), 7.80–7.16 (m, 14 H, 14 x CH_{Ar}), 7.09–7.08 (m,1 H, CH_{Ar}), 6.94–6.92 (m, 1 H, CH_{Ar}).

^{13}C{^{1}H}-NMR (100.56 MHz, CD_2Cl_2) δ = 151.1 (s, C_{Ar}), 145.6 (d, J(^{13}C-^{31}P) = 2.4 Hz, C_{Ar}), 140.9 (d, J(^{13}C-^{31}P) = 21.6 Hz, C_{Ar}), 134.5 (d, J(^{13}C-^{31}P) = 20.2 Hz, C_{Ar}), 134.2 (s, C_{Ar}), 134.1 (s, C_{Ar}), 133.4 (d, J(^{13}C-^{31}P) = 8.1 Hz, C_{Ar}), 133.3 (s, C_{Ar}), 132.4 (d, J(^{13}C-^{31}P) = 8.2 Hz, C_{Ar}), 131.6 (d, J(^{13}C-^{31}P) = 9.1 Hz, C_{Ar}), 130.6 (d, J(^{13}C-^{31}P) = 1.8 Hz, C_{Ar}), 129.5 (d, J(^{13}C-^{31}P) = 8.9 Hz, C_{Ar}), 128.8 (s, C_{Ar}), 128.5 (d, J(^{13}C-^{31}P) = 5.9 Hz, C_{Ar}), 128.1 (s, C_{Ar}), 126.9 (s, C_{Ar}), 126.4 (s, C_{Ar}), 125.5 (d, J(^{13}C-^{31}P) = 11.4 Hz, C_{Ar}), 125.2 (d, J(^{13}C-^{31}P) = 1.4 Hz, C_{Ar}), 125.0 (d, J(^{13}C-^{31}P) = 2.3 Hz, C_{Ar}), 124.9 (s, C_{Ar}).

^{31}P-NMR (202.30 MHz, CD_2Cl_2) δ = 4.8 (s).

Elemental Analysis (%): calculated for $C_{24}H_{17}PS$: C 78.24, H 4.65, found: C 78.40, H 4.46.

MS (APCI-HR) m/z: found: 369.08614 [**4c** +H]$^+$, calculated: 369.08613.

Synthesis of the phosphetes **5a**, **5b**, **5b'**, **5c**

Synthesis of phosphete **5a**

Phosphete **3a** (58 mg, 1 eq. 0.15 mmol) was dissolved in DMF (0.6 mL) and KOTMS (2 mg, 0.02 mmol, 0.1 eq.) was added. The colour of the reaction mixture changed to deep green. After 5 min at rt the reaction was terminated by addition of water and the crude product was purified by column chromatography with *n*pentane/DCM 10:1 ($R_F = 0.1$). The product was obtained as colourless, crystalline solid.

Yield: 94% (44 mg).

^{1}H-NMR (399.87 MHz, THF-d_8) $\delta = 7.72–7.68$ (m, 2 H, 2 x CH_{Ar}), 7.43–7.07 (m, 14 H, 14 x CH_{Ar}), 6.75 (d, $^3J(^1H-^{31}P) = 19.5$ Hz, 1 H, PhC*H*).

^{13}C{^{1}H}-NMR (100.56 MHz, THF-d_8) $\delta = 155.0$ (s, C_{Ar}), 139.2 (d, $J(^{13}C-^{31}P) = 2.9$ Hz, C_{Ar}), 138.3 (s, C_{Ar}), 136.2 (d, $J(^{13}C-^{31}P) = 30.9$ Hz, C_{Ar}), 135.3 (d, $J(^{13}C-^{31}P) = 10.7$ Hz, C_{Ar}), 133.9 (d, $J(^{13}C-^{31}P) = 19.5$ Hz, C_{Ar}), 132.4 (d, $J(^{13}C-^{31}P) = 11.1$ Hz, C_{Ar}), 131.0 (s, C_{Ar}), 129.6 (s, C_{Ar}), 129.5 (s, C_{Ar}), 129.5 (s, C_{Ar}), 129.3 (s, C_{Ar}), 128.2 (d, $J(^{13}C-^{31}P) = 2.9$ Hz, C_{Ar}), 128.2 (s, C_{Ar}), 126.7 (d, $J(^{13}C-^{31}P) = 5.7$ Hz, C_{Ar}), 124.9 (d, $J(^{13}C-^{31}P) = 4.3$ Hz, C_{Ar}).

^{31}P-NMR (202.30 MHz, THF-d_8) $\delta = 20.6$ (s).

Elemental Analysis (%): calculated for $C_{22}H_{17}P$: C 84.60, H 5.49., found: C 84.66, H 5.60.

MS (APCI-HR) m/z: found: 313.11406 [**5a**+H]$^+$, calculated: 313.11406.

Synthesis of phosphete **5b** and **5b'**

Phosphete **2b/2b'** (43 mg, 1 eq. 0.1 mmol) was dissolved in DMF (0.6 mL) and KOTMS (1 mg, 0.01 mmol, 0.1 eq.) was added. The colour of the reaction mixture changed to orange. After 2 d at rt the reaction was terminated by addition of water and the crude product was purified by column chromatography with *n*pentane/DCM 10:1 ($R_F = 0.1$). The product was obtained as colourless solid.

Yield: 86% (31 mg).

^{1}H-NMR (399.87 MHz, CD_2Cl_2) $\delta = 7.92–7.16$ (m, 18 H, 18 x CH_{Ar}), 6.92 (d, $^3J(^1H-^{31}P) = 19.4$ Hz 0.6 H, TMSCC*H*), 6.79 (d, $^3J(^1H-^{31}P) = 19.5$ Hz 0.4 H, TMSCC*H*).

Due to overlapping issues in ^{13}C-NMR a peak list is provided.

$^{13}C\{^1H\}$-NMR (100.56 MHz, CD_2Cl_2) δ = 154.4, 154.1, 139.4, 139.4, 139.1, 139.0, 138.9, 138.9, 138.6, 138.5, 137.6, 135.6, 135.4, 135.3, 134.6, 134.5, 134.1, 133.9, 133.9, 133.7, 133.6, 133.5, 133.4, 133.3, 133.3, 130.8, 130.7, 129.3, 129.3, 129.2, 129.2, 129.2, 129.1, 129.0, 129.0, 128.9, 128.8, 128.8, 128.5, 128.4, 128.2, 128.2, 128.1, 128.0, 127.8, 127.7, 127.3, 127.3, 127.2, 127.1, 126.8, 126.5, 126.4, 126.3, 126.1, 126.1, 125.1, 125.1, 124.5, 124.5, 124.4, 124.4, 123.4, 123.3.

^{31}P-NMR (202.30 MHz, CD_2Cl_2) δ = 18.5 (s), 18.5 (s).

Elemental Analysis (%): calculated for $C_{26}H_{19}P$: C 86.17, H 5.28, found: C 86.34, H 5.43.

MS (APCI-HR) m/z: found: 363.12971 [**5b**/**5b’**+H]$^+$, calculated: 363.12971.

Synthesis of phosphete **5c**

Phosphete **2c** (20 mg, 1 eq. 0.05 mmol) was dissolved in DMF (0.6 mL) and KOTMS (1 mg, 0.01 mmol, 0.1 eq.) was added. The colour of the reaction mixture changed to orange. After 2 h at rt the reaction was terminated by addition of water and the crude product was purified by column chromatography with *n*pentane/DCM 10:1 (R_F = 0.1). The product was obtained as yellow solid.

Yield: 90% (15 mg).

^1H-NMR (399.87 MHz, CD_2Cl_2) δ = 7.85–7.34 (m, 13 H, 13 x CH_{Ar}), 7.16 (d, $^3J(^1H\text{-}^{31}P)$ = 12.7 Hz, 1 H, PCC*H*), 7.02–6.97 (m, 2 H, 2 x CH_{Ar}), 6.90 (d, $^3J(^1H\text{-}^{31}P)$ = 12.7 Hz, 1 H, PCC*H*).

$^{13}C\{^1H\}$-NMR (100.56 MHz, CD_2Cl_2) δ = 147.4 (d, $J(^{13}C\text{-}^{31}P)$ = 3.1 Hz, C_{Ar}), 138.9 (s, C_{Ar}), 138.8 (s, C_{Ar}), 137.0 (d, $J(^{13}C\text{-}^{31}P)$ = 11.1 Hz, C_{Ar}), 135.5 (s, C_{Ar}), 135.3 (s, C_{Ar}), 135.2 (s, C_{Ar}), 134.1 (s, C_{Ar}), 133.5 (d, $J(^{13}C\text{-}^{31}P)$ = 19.5 Hz, C_{Ar}), 133.3 (s, C_{Ar}), 130.9 (s, C_{Ar}), 129.3 (d, $J(^{13}C\text{-}^{31}P)$ = 7.1 Hz, C_{Ar}), 128.5 (d, $J(^{13}C\text{-}^{31}P)$ = 2.0 Hz, C_{Ar}), 128.4 (s, C_{Ar}), 128.2 (s, C_{Ar}), 128.1 (d, $J(^{13}C\text{-}^{31}P)$ = 6.7 Hz, C_{Ar}), 127.5 (d, $J(^{13}C\text{-}^{31}P)$ = 3.8 Hz, C_{Ar}), 127.2 (d, $J(^{13}C\text{-}^{31}P)$ = 3.4 Hz, C_{Ar}), 126.9 (s, C_{Ar}), 126.5 (s, C_{Ar}), 125.0 (d, $J(^{13}C\text{-}^{31}P)$ = 7.9 Hz, C_{Ar}), 124.4 (d, $J(^{13}C\text{-}^{31}P)$ = 4.1 Hz, C_{Ar}).

^{31}P-NMR (202.30 MHz, CD_2Cl_2) δ = 25.1 (s).

Elemental Analysis (%): calculated for $C_{24}H_{17}PS$+1.5 H_2O: C 72.89, H 5.10, found: C 72.82, H 4.83.

MS (APCI-HR) m/z: found: 369.08614 [**5c**+H]$^+$, calculated: 369.08613.

Synthesis of the phosphanes **10a**, **10b**, **10c**

Synthesis of phosphane **10a**

A Schlenk tube was charged with DME (20 mL) and cooled to –60 °C. Then, phenylphosphane (0.12 g, 1.1 mmol, 1 eq.) was added. After that, *n*BuLi (2.5 M, 0.44 mL, 1.1 mmol, 1 eq.) was used for lithiation. The orange solution was stirred for 30 min and quenched with trimethylchlorosilane (0.14 mL, 1.1 mmol, 1eq.). The colourless solution was lithiated with *n*BuLi (2.5 M, 0.44 mL, 1.1 mmol, 1 eq.) again. After further 30 min, the reaction mixture was cooled to –80 °C and 1,4-diphenylbuta-1,3-diyne (0.22 g, 1.1 mmol, 1.0 eq.), dissolved in DME (10 mL), was added dropwise to the orange solution. The colour changed to deep blue and after 2 h the reaction was cooled to –90 °C and precooled ethanol was added. After that, saturated NH_4Cl solution was added and the colour of the solution turned bright yellow. Then, the raw product was extracted with DCM three times (3 x 30 mL). The combined organic layers were dried with $MgSO_4$ and the solvent was removed. The crude product was purified by silica gel column chromatography with *n*pentane/dichloromethane 10:1 (R_F = 0.4). The product was bright yellow oil.

Yield: 33% (0.14 g).

^{1}H-NMR (399.87 MHz, CD_2Cl_2) δ = 7.34–7.10 (m, 15 H, 15 x CH_{Ar}), 5.21 (d, $^1J(^1H-^{31}P)$ = 221.3 Hz, 1 H, *H*-P), 0.53 (s, 9 H, 3 x SiCH_3).

^{13}C{^{1}H}-NMR (100.56 MHz, CD_2Cl_2) δ = 159.5 (d, $J(^{13}C-^{31}P)$ = 18.0 Hz, C_{Ar}), 145.0 (s, C_{Ar}), 133.8 (d, $J(^{13}C-^{31}P)$ = 15.7 Hz, C_{Ar}), 131.8 (s, C_{Ar}), 128.9 (d, $J(^{13}C-^{31}P)$ = 5.7 Hz, C_{Ar}), 128.8 (s, C_{Ar}), 128.6 (d, $J(^{13}C-^{31}P)$ = 7.7 Hz, C_{Ar}), 128.5 (d, $J(^{13}C-^{31}P)$ = 1.9 Hz, C_{Ar}), 128.0 (s, C_{Ar}), 127.4 (s, C_{Ar}), 124.5 (s, C_{Ar}),

129.4 (d, $J(^{13}C-^{31}P) = 7.0$ Hz, C_{Ar}), 128.7 (s, C_{Ar}), 124.5 (s, C_{Ar}), 128.6 (s, C_{Ar}), 99.9 (d, $J(^{13}C-^{31}P) = 3.7$ Hz, CC_{Alkyne}), 93.2 (d, $J(^{13}C-^{31}P) = 22.1$ Hz, CC_{Alkyne}), 1.6 (s, SiCH_3).

^{29}Si{^{1}H}-NMR (99.28 MHz, CD_2Cl_2) $\delta = -4.3$ (d, $^3J(^{29}Si-^{31}P) = 7.5$ Hz).

^{31}P-NMR (202.30 MHz, CD_2Cl_2) $\delta = -37.7$ (d, $^1J(^1H-^{31}P) = 222$ Hz).

Elemental Analysis (%): calculated for $C_{25}H_{25}PSi$: C 78.09, H 6.55, found: C 78.16, H 6.63.

MS (APCI-HR) m/z: found: 385.15359. [**10a**+H]$^+$, calculated: 385.15359.

Synthesis of phosphane **10b**

A Schlenk tube was charged with DME (20 mL) and cooled to –50 °C. Then, phenylphosphane (0.11 g, 1.0 mmol, 1 eq.) was added. After that, *n*BuLi (2.5 M, 0.4 mL, 1.0 mmol, 1 eq.) was used for lithiation. The orange solution was stirred for 45 min and quenched with trimethylchlorosilane (0.13 mL, 1.0 mmol, 1 eq.). The colourless solution was lithiated with *n*BuLi (2.5 M, 0.4 mL, 1.0 mmol, 1 eq.) again. After further 45 min, the reaction mixture was cooled to –90 °C and 2-(phenylbuta-1,3-diyn-1-yl)naphtaline (0.25 g, 1 mmol, 1 eq.), dissolved in DME (10 mL), was added dropwise to the orange solution. The colour changed to deep blue and after 2 h ethanol was added. After that, saturated NH_4Cl solution was added, and the colour of the solution turned orange. Then, the raw product was extracted with DCM three times (3 x 30 mL). The combined organic layers were dried with $MgSO_4$ and the solvent was removed. The crude product was purified by silica gel column chromatography with *n*pentane/dichloromethane 4:1 ($R_F = 0.4$). The product was yellow oil.

Yield: 9% (32 mg).

^{1}H-NMR (399.87 MHz, CD_2Cl_2) $\delta = 7.95$ (s, 1 H, CH_{Ar}), 7.85–7.81 (m, 3 H, 3 x CH_{Ar}), 7.53–7.45 (m, 5 H, 5 x C_{Ar}), 7.27–7.21 (m, 6 H, 6 x CH_{Ar}), 7.04–7.03 (m, 2 H, 2 x CH_{Ar}), 5.72 (d, $^1J(^1H-^{31}P) = 229.8$ Hz, 1 H, *H*-P), 0.00 (s, 9 H, 3 x SiCH_3).

^{13}C{^{1}H}-NMR (100.56 MHz, CD_2Cl_2) $\delta = 161.6$ (d, $J(^{13}C-^{31}P) = 25.0$ Hz, C_{Ar}), 143.3 (d, $J(^{13}C-^{31}P) = 8.9$ Hz, C_{Ar}), 135.3 (d, $J(^{13}C-^{31}P) = 16.7$ Hz, C_{Ar}), 133.7 (s, C_{Ar}), 133.5 (d,

$J(^{13}C\text{-}^{31}P)$ = 11.5 Hz, C_{Ar}), 133.3 (s, C_{Ar}), 133.0 (d, $J(^{13}C\text{-}^{31}P)$ = 10.7 Hz, C_{Ar}), 131.3 (s, C_{Ar}), 129.0 (s, C_{Ar}), 128.9 (d, $J(^{13}C\text{-}^{31}P)$ = 2.3 Hz, C_{Ar}), 128.8 (d, $J(^{13}C\text{-}^{31}P)$ = 1.7 Hz, C_{Ar}), 128.7 (s, C_{Ar}), 128.6 (s, C_{Ar}), 128.3 (s, C_{Ar}), 128.3 (s, C_{Ar}), 128.3 (s, C_{Ar}), 127.8 (s, C_{Ar}), 127.2 (s, C_{Ar}), 127.2 (s, C_{Ar}), 121.9 (s, C_{Ar}), 102.1 (d, $J(^{13}C\text{-}^{31}P)$ = 1.7 Hz, CC_{Alkyne}), 93.1 (d, $J(^{13}C\text{-}^{31}P)$ = 9.2 Hz, CC_{Alkyne}), 0.3 (d, $J(^{13}C\text{-}^{31}P)$ = 1.2 Hz, $SiCH_3$).

^{29}Si{^{1}H}-NMR (99.28 MHz, CD_2Cl_2) δ = –3.4 (d, $^3J(^{29}Si\text{-}^{31}P)$ = 3.4 Hz).

^{31}P-NMR (202.30 MHz, CD_2Cl_2) δ = –32.1 (d, $^1J(^1H\text{-}^{31}P)$ = 230 Hz).

Elemental Analysis (%): calculated for $C_{29}H_{27}$Psi+1.5 H_2O: C 75.46, H 6.55, found: C 75.31, H 6.59.

MS (APCI-HR) m/z: found: 435.16924 [**10b**+H]$^+$, calculated: 435.16924.

Synthesis of phosphane **10c**

A Schlenk tube was charged with DME (20 mL) and cooled to –60 °C. Then, phenylphosphane (0.11 g, 1.0 mmol, 1 eq.) was added. After that, *n*BuLi (2.5 M, 0.4 mL, 1.0 mmol, 1 eq.) was used for lithiation. The orange solution was stirred for 45 min and quenched with trimethylchlorosilane (0.13 mL, 1.0 mmol, 1 eq.). The colourless solution was lithiated with *n*BuLi (2.5 M, 0.4 mL, 1.0 mmol, 1 eq.) again. After further 30 min, the reaction mixture was cooled to –80 °C and 2-(naphtalin-2-ylbuta-1,3-diyn-1-yl)thiophene (0.26 g, 1.0 mmol, 1.0 eq.), dissolved in DME (10 mL), was added dropwise to the orange solution. The colour changed to deep blue and after 2 h ethanol was added. After that, saturated NH_4Cl solution was added and the colour of the solution turned yellow. Then, the raw product was extracted with DCM three times (3 x 30 mL). The combined organic layers were dried with $MgSO_4$ and the solvent was removed. The crude product was purified by silica gel column chromatography with *n*pentane/dichloromethane 10:1 (R_F = 0.4). The product was bright yellow oil.

Yield: 45% (0.20 g).

Isomer 1

^{1}H-NMR (399.87 MHz, CD_2Cl_2) δ = 8.04 (br. s, 1 H, C*H*$_{Ar}$), 7.89–7.82 (m, 3 H, 3 x C*H*$_{Ar}$), 7.63–7.48 (m, 9 H, 9 x C$_{Ar}$), 7.04–6.81 (m, 2 H, 2 x C*H*$_{Ar}$), 5.95 (d, $^{1}J(^{1}H\text{-}^{31}P)$ = 229.7 Hz, 1 H, *H*-P), 0.22 (s, 9 H, 3 x SiC*H*$_3$).

^{13}C{^{1}H}-NMR (100.56 MHz, CD_2Cl_2) δ = 153.7 (d, $J(^{13}C\text{-}^{31}P)$ = 24.1 Hz, *C*$_{Ar}$), 144.8 (d, $J(^{13}C\text{-}^{31}P)$ = 12.0 Hz, *C*$_{Ar}$), 135.3 (d, $J(^{13}C\text{-}^{31}P)$ = 16.7 Hz, *C*$_{Ar}$), 133.7 (s, *C*$_{Ar}$), 133.4 (s, *C*$_{Ar}$), 132.3 (d, $J(^{13}C\text{-}^{31}P)$ = 14.9 Hz, *C*$_{Ar}$), 131.8 (s, *C*$_{Ar}$), 131.5 (s, *C*$_{Ar}$), 129.3 (s, *C*$_{Ar}$), 129.2 (d, $J(^{13}C\text{-}^{31}P)$ = 5.1 Hz, *C*$_{Ar}$), 128.9 (d, $J(^{13}C\text{-}^{31}P)$ = 6.6 Hz, *C*$_{Ar}$), 128.7 (s, *C*$_{Ar}$), 128.6 (s, *C*$_{Ar}$), 128.5 (d, $J(^{13}C\text{-}^{31}P)$ = 10.3 Hz, *C*$_{Ar}$), 128.4 (d, $J(^{13}C\text{-}^{31}P)$ = 3.0 Hz, *C*$_{Ar}$), 128.3 (d, $J(^{13}C\text{-}^{31}P)$ = 1.7 Hz, *C*$_{Ar}$), 127.3 (s, *C*$_{Ar}$), 127.2 (d, $J(^{13}C\text{-}^{31}P)$ = 1.4 Hz, *C*$_{Ar}$), 126.8 (s, *C*$_{Ar}$), 122.0 (s, *C*$_{Ar}$), 103.5 (d, $J(^{13}C\text{-}^{31}P)$ = 1.9 Hz, *C*C$_{Alkyne}$), 93.3 (d, $J(^{13}C\text{-}^{31}P)$ = 7.8 Hz, *C*C$_{Alkyne}$), 0.4 (s, Si*C*H$_3$).

^{29}Si{^{1}H}-NMR (99.28 MHz, CD_2Cl_2) δ = –2.9 (d, $^{3}J(^{29}Si\text{-}^{31}P)$ = 2.5 Hz).

^{31}P-NMR (202.30 MHz, CD_2Cl_2) δ = –28.4 (d, $^{1}J(^{1}H\text{-}^{31}P)$ = 230 Hz).

Isomer 2

^{1}H-NMR (399.87 MHz, CD_2Cl_2) δ = 8.07 (s, br. 1 H, 1 x C*H*$_{Ar}$), 7.91–7.81 (m, 3 H, 3 x C*H*$_{Ar}$), 7.64–7.30 (m, 9 H, 9 x C*H*$_{Ar}$), 7.04–6.81 (m, 2 H, 2 x C*H*$_{Ar}$), 5.57 (d, $^{1}J(^{1}H\text{-}^{31}P)$ = 221.3 Hz, 1 H, *H*-P), 0.62 (s, 9 H, 3 x SiC*H*$_3$).

^{13}C{^{1}H}-NMR (100.56 MHz, CD_2Cl_2) δ = 147.7 (d, $J(^{13}C\text{-}^{31}P)$ = 18.0 Hz, *C*$_{Ar}$), 146.4 (s, *C*$_{Ar}$), 139.3 (d, $J(^{13}C\text{-}^{31}P)$ = 12.1 Hz, *C*$_{Ar}$), 137.1 (s, *C*$_{Ar}$), 136.6 (s, *C*$_{Ar}$), 136.4 (d, $J(^{13}C\text{-}^{31}P)$ = 11.0 Hz, *C*$_{Ar}$), 135.3 (s, *C*$_{Ar}$), 134.3 (d, $J(^{13}C\text{-}^{31}P)$ = 18.7 Hz, *C*$_{Ar}$), 133.7 (d, $J(^{13}C\text{-}^{31}P)$ = 12.1 Hz, *C*$_{Ar}$), 133.5 (s, *C*$_{Ar}$), 129.8 (d, $J(^{13}C\text{-}^{31}P)$ = 1.5 Hz, *C*$_{Ar}$), 128.7 (s, *C*$_{Ar}$), 128.6 (s, *C*$_{Ar}$), 127.5 (s, *C*$_{Ar}$), 127.5 (s, *C*$_{Ar}$), 127.3 (s, *C*$_{Ar}$), 127.0 (s, *C*$_{Ar}$), 126.8 (s, *C*$_{Ar}$), 126.6 (s, *C*$_{Ar}$), 121.8 (s, *C*$_{Ar}$), 105.0 (d, $J(^{13}C\text{-}^{31}P)$ = 3.3 Hz, *C*C$_{Alkyne}$), 94.9 (d, $J(^{13}C\text{-}^{31}P)$ = 26.6 Hz, *C*C$_{Alkyne}$), 2.2 (d, $J(^{13}C\text{-}^{31}P)$ = 9.1 Hz, Si*C*H$_3$).

^{29}Si{^{1}H}-NMR (99.28 MHz, CD_2Cl_2) δ = –3.7 (d, $^{3}J(^{29}Si\text{-}^{31}P)$ = 10.9 Hz).

^{31}P-NMR (202.30 MHz, CD_2Cl_2) δ = –39.7 (d, $^{1}J(^{1}H\text{-}^{31}P)$ = 221 Hz).

Elemental Analysis (%): calculated for $C_{27}H_{25}PSSi$: C 73.60, H 5.72, found: C 73.24, H 5.78.

MS (APCI-HR) m/z: found: 441.12566 [**10c**+H]$^+$, calculated: 441.12566.

Synthesis of phosphole **2d**

A Schlenk tube was charged with DME (20 mL) and cooled to –60 °C. Then, phenylphosphane (0.11 g, 1 mmol, 1.0 eq.) was added. After that, *n*BuLi (2.5 M, 0.42 mL, 1.05 mmol, 1.05 eq.) was used for lithiation. The orange solution was stirred for 60 min and quenched with trimethylchlorosilane (0.13 mL, 1 mmol, 1eq.). The colourless solution was lithiated with *n*BuLi (2.5 M, 0.42 mL, 1.05 mmol, 1.05 eq.) again. After further 30 min, the reaction mixture was cooled to –80 °C and 2,2,7,7-tetramethylocta-2,5-diyne (0.18 g, 1.0 mmol, 1 eq.), dissolved in DME (10 mL), was added dropwise to the orange solution at –35 °C. The colour changed to deep blue and after 3 h stirring at rt the reaction was terminated by adding ethanol. Afterwards, the solvent was removed, and the crude product was purified by silica gel column chromatography with *n*pentane/DCM 10:1 (R_F = 0.4). The product was obtained as colourless solid.

Yield: 20% (0.07 g).

^{1}H-NMR (399.87 MHz, CD_2Cl_2) δ = 7.29–7.17 (m, 11 H, 11 x CH_{Ar}), 6.93 (d, $^3J(^1H-^{31}P)$ = 12.7 Hz, 1 H, Phosphol-*H*), 1.19 (s, 9 H, 3 x CH_3), 0.11 (s, 9 H, 3 x Si(CH_3).

^{13}C{^{1}H}-NMR (100.56 MHz, CD_2Cl_2) δ = 163.9 (d, $J(^{13}C-^{31}P)$ = 6.3 Hz, C_{Ar}), 162.5 (d, $J(^{13}C-^{31}P)$ = 3.6 Hz, C_{Ar}), 147.1 (d, $J(^{13}C-^{31}P)$ = 7.1 Hz, C_{Ar}), 139.8 (d, $J(^{13}C-^{31}P)$ = 19.3 Hz, C_{Ar}), 136.8 (d, $J(^{13}C-^{31}P)$ = 12.8 Hz, C_{Ar}), 135.0 (d, $J(^{13}C-^{31}P)$ = 19.9 Hz, C_{Ar}), 132.1 (d, $J(^{13}C-^{31}P)$ = 10.3 Hz, C_{Ar}), 130.0 (d, $J(^{13}C-^{31}P)$ = 1.9 Hz, C_{Ar}), 129.7 (d, $J(^{13}C-^{31}P)$ = 7.1 Hz, C_{Ar}), 128.9 (d, $J(^{13}C-^{31}P)$ = 8.6 Hz, C_{Ar}), 128.1 (s, C_{Ar}), 127.2 (d, $J(^{13}C-^{31}P)$ = 1.3 Hz, C_{Ar}), 36.0 (d, $J(^{13}C-^{31}P)$ = 14.2 Hz, *C*CH_3), 32.9 (d, $J(^{13}C-^{31}P)$ = 5.7 Hz, CCH_3) 0.7 (d, $J(^{13}C-^{31}P)$ = 1.4 Hz, Si(CH_3)$_3$).

^{29}Si{^{1}H}-NMR (99.28 MHz, CD_2Cl_2) δ = –8.4.

^{31}P-NMR (202.30 MHz, CD_2Cl_2) δ = 21.8 (s).

Elemental Analysis (%): calculated for $C_{23}H_{29}PSi$: C 75.78, H 8.02, found: C 75.56, H 8.24.

MS (APCI-HR) m/z: found: 365.18589 [**2d**+H]$^+$, calculated: 365.18589.

Synthesis of phosphole **4d**

Method A

A Schlenk tube was charged with THF (20 mL) and cooled to –80 °C. Then, phenylphosphane (0.12 g, 1.1 mmol, 1.0 eq.) was added. After that, *n*BuLi (2.5 M, 0.5 mL, 1.2 mmol, 1.1 eq.) was used for lithiation. The orange solution was stirred for 30 min (5,5-dimethylhexa-1,3-diyn-1-yl)benzene (0.20 g, 1.1 mmol, 1.0 eq.), dissolved in THF (10 mL), was added dropwise to the orange solution. The colour changed to black blue and after 20 min the reaction was terminated by adding ethanol. Afterwards, the solvent was removed and the crude product was purified by silica gel column chromatography with *n*pentane/dichloromethane 10:1 (R_F = 0.5). The product was obtained as colourless solid. Yield: 0.08 g (29%).

Method B

Phosphole **2d** (73 mg, 1 eq. 0.20 mmol) was dissolved in DMSO (12 mL) and KOTMS (5 mg, 0.04 mmol, 0.2 eq.) was added. The colour of the reaction mixture changed to deep blue. After 2 h min at rt °C the reaction was terminated by addition of water and the crude product was purified by column chromatography as mentioned in method A.

Yield: 99% (57 mg).

^{1}H-NMR (399.87 MHz, CD_2Cl_2) δ = 7.45–7.43 (m, 4 H, 4 x CH_{Ar}), 7.31–7.19 (m, 7 H, 7 x CH_{Ar}), 6.82 (dd, $^3J(^1H\text{-}^{31}P)$ = 12.4 Hz, $^3J(^1H\text{-}^1H)$ = 3.2 Hz, 1 H, *t*BuCC*H*), 1.15 (s, 9 H, 3 x CH_3).

^{13}C{^{1}H}-NMR (100.56 MHz, CD_2Cl_2) δ = 165.6 (d, $J(^{13}C\text{-}^{31}P)$ = 9.1 Hz, C_{Ar}), 150.7 (s, C_{Ar}), 137.0 (d, $J(^{13}C\text{-}^{31}P)$ = 16.7 Hz, C_{Ar}), 135.0 (d, $J(^{13}C\text{-}^{31}P)$ = 20.5 Hz, C_{Ar}), 132.2 (d, $J(^{13}C\text{-}^{31}P)$ = 8.8 Hz, C_{Ar}), 131.7 (d, $J(^{13}C\text{-}^{31}P)$ = 3.8 Hz, C_{Ar}), 131.6 (s, C_{Ar}), 130.2 (d, $J(^{13}C\text{-}^{31}P)$ = 1.9 Hz, C_{Ar}), 129.0 (s, C_{Ar}), 129.0 (s, C_{Ar}), 127.3 (s, C_{Ar}), 126.6 (d, $J(^{13}C\text{-}^{31}P)$ = 9.2 Hz, C_{Ar}), 36.1 (d, $J(^{13}C\text{-}^{31}P)$ = 14.1 Hz, *C*CH_3), 32.6 (d, $J(^{13}C\text{-}^{31}P)$ = 6.0 Hz, CCH_3).

^{31}P-NMR (202.30 MHz, CD_2Cl_2) δ = 0.8 (s).

Elemental Analysis (%): calculated for $C_{20}H_{21}P$: C 82.17, H 7.24, found: C 81.84, H 7.39.

MS (APCI-HR) m/z: found: 293.14536 [**4d**+H]$^+$, calculated: 293.14536.

Synthesis of phosphane **10d'**

A Schlenk tube was charged with DME (20 mL) and cooled to –60 °C. Then, phenylphosphane (0.11 g, 1 mmol, 1.0 eq.) was added. After that, *n*BuLi (2.5 M, 0.4 mL, 1 mmol, 1 eq.) was used for lithiation. The orange solution was stirred for 40 min and quenched with trimethylchlorosilane (0.13 mL, 1 mmol, 1eq.). The colourless solution was lithiated with *n*BuLi (2.5 M, 0.4 mL, 1 mmol, 1 eq.) again. After further 75 min, the reaction mixture was cooled to –80 °C and (5,5-dimethylhexa-1,3-diyn-1-yl)benzene (0.18 g, 1 mmol, 1.0 eq.), dissolved in DME (10 mL), was added dropwise to the orange solution. The colour changed to deep blue-black and after 3 hours the reaction was terminated by adding ethanol. Afterwards, the solvent was removed and the crude product was purified by silica gel column chromatography with *n*pentane/dichloromethane 10:1 (R_F = 0.3). The product was obtained as light-yellow wax.

Yield: 0.08 g (23%).

^{1}H-NMR (399.87 MHz, CD_2Cl_2) δ = 7.54–7.26 (m, 10 H, 10 x CH_{Ar}), 5.32 (d, $^{1}J(^{1}H\text{-}^{31}P)$ = 223.9 Hz, 1 H, P*H*), 1.39 (s, 9 H, 3 x CCH_3), 0.41 (d, *J* = 1.7 Hz, 9 H, 3 x SiCH_3).

^{13}C{^{1}H}-NMR (100.56 MHz, CD_2Cl_2) δ = 168.7 (d, $J(^{13}C\text{-}^{31}P)$ = 21.0 Hz, C_{Ar}), 136.6 (d, $J(^{13}C\text{-}^{31}P)$ = 14.1 Hz, C_{Ar}), 132.5 (d, $J(^{13}C\text{-}^{31}P)$ = 15.1 Hz, C_{Ar}), 131.9 (s, C_{Ar}), 131.3 (s, C_{Ar}), 129.0 (s, C_{Ar}), 129.0 (s, C_{Ar}), 128.7 (s, C_{Ar}), 128.2 (s, C_{Ar}), 125.0 (s, C_{Ar}), 105.6 (d, $J(^{13}C\text{-}^{31}P)$ = 3.6 Hz, C_{Alkyne}), 93.9 (d, $J(^{13}C\text{-}^{31}P)$ = 28.1 Hz, C_{Alkyne}), 30.8 (d, $J(^{13}C\text{-}^{31}P)$ = 2.6 Hz, *C*CH$_3$), 30.0 (d, $J(^{13}C\text{-}^{31}P)$ = 4.9 Hz, C*C*H$_3$), 2.6 (s, Si*C*H$_3$).

^{31}P-NMR (202.30 MHz, CD_2Cl_2) δ = –48.9 (d, $^{1}J(^{1}H\text{-}^{31}P)$ = 224 Hz).

Elemental Analysis (%): calculated for $C_{23}H_{29}PS$+O: C 72.59, H 7.68; found: C: 72.63, H 7.84.

MS (APCI) m/z: found: 381.16. [**10d’**O+H]$^+$, calculated: 381.18.

c) Spectral data

NMR spectra for compound **1c**

Fig. 4: ^{1}H NMR ($CDCl_3$, 400 MHz)

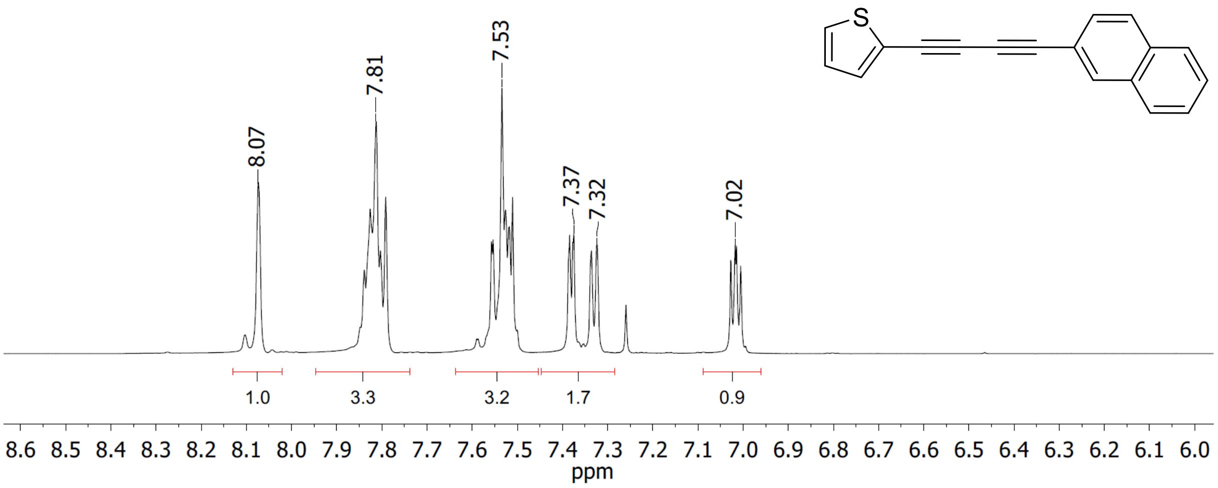

Fig. S5: ^{13}C NMR ($CDCl_3$, 101 MHz)

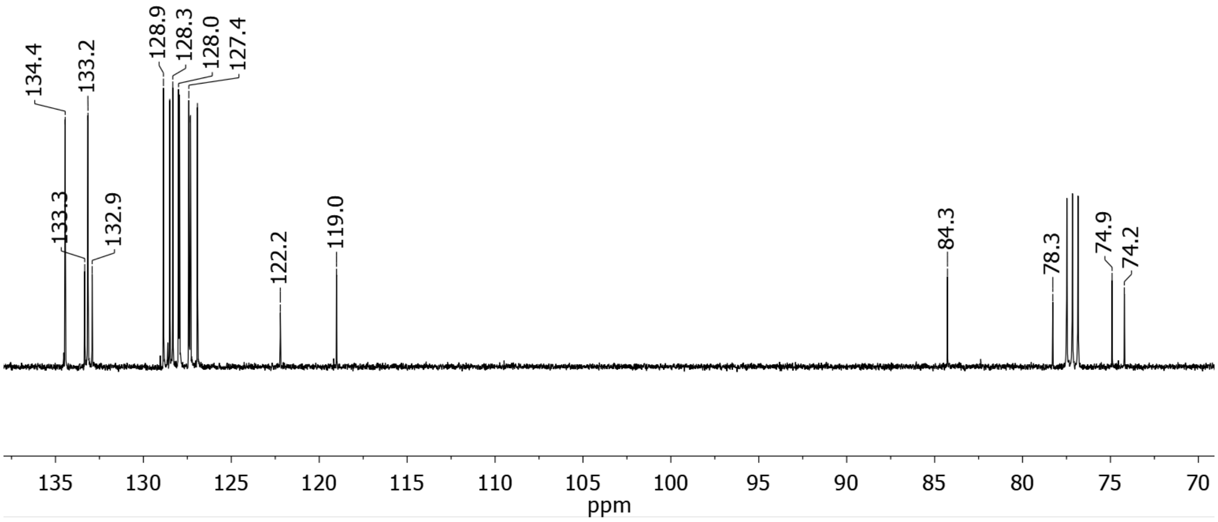

NMR spectra for compound **3a**

Fig. S6: ^{1}H NMR (CD_2Cl_2, 400 MHz)

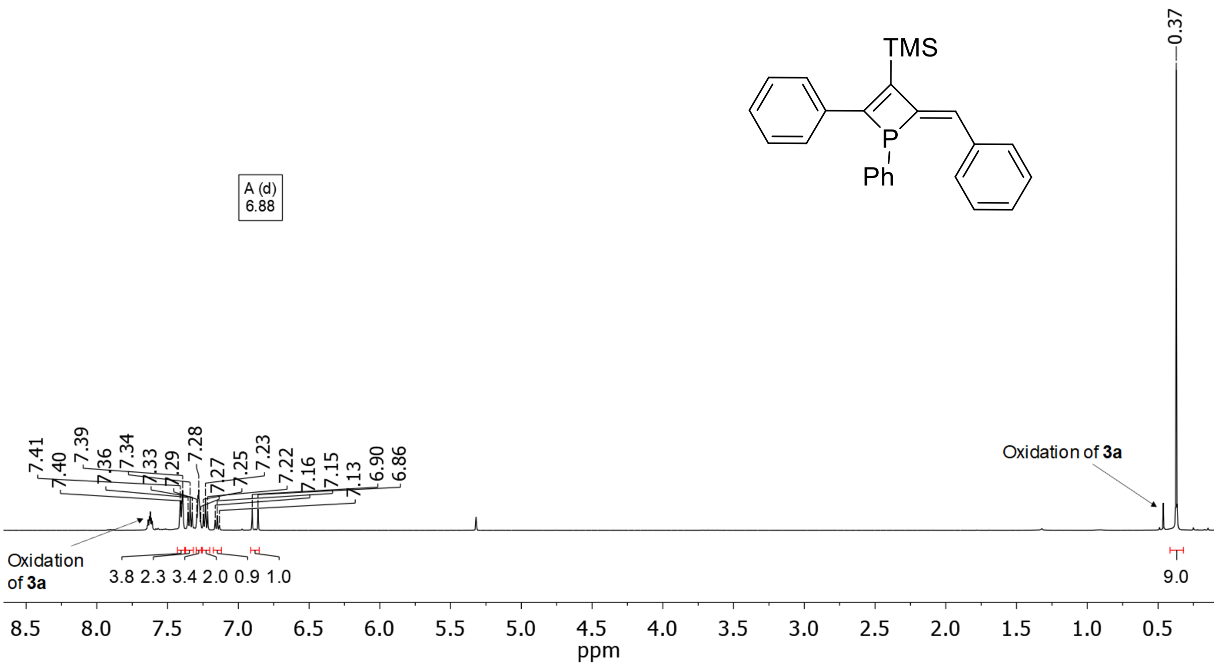

Fig. S7: ^{13}C NMR (CD_2Cl_2, 101 MHz)

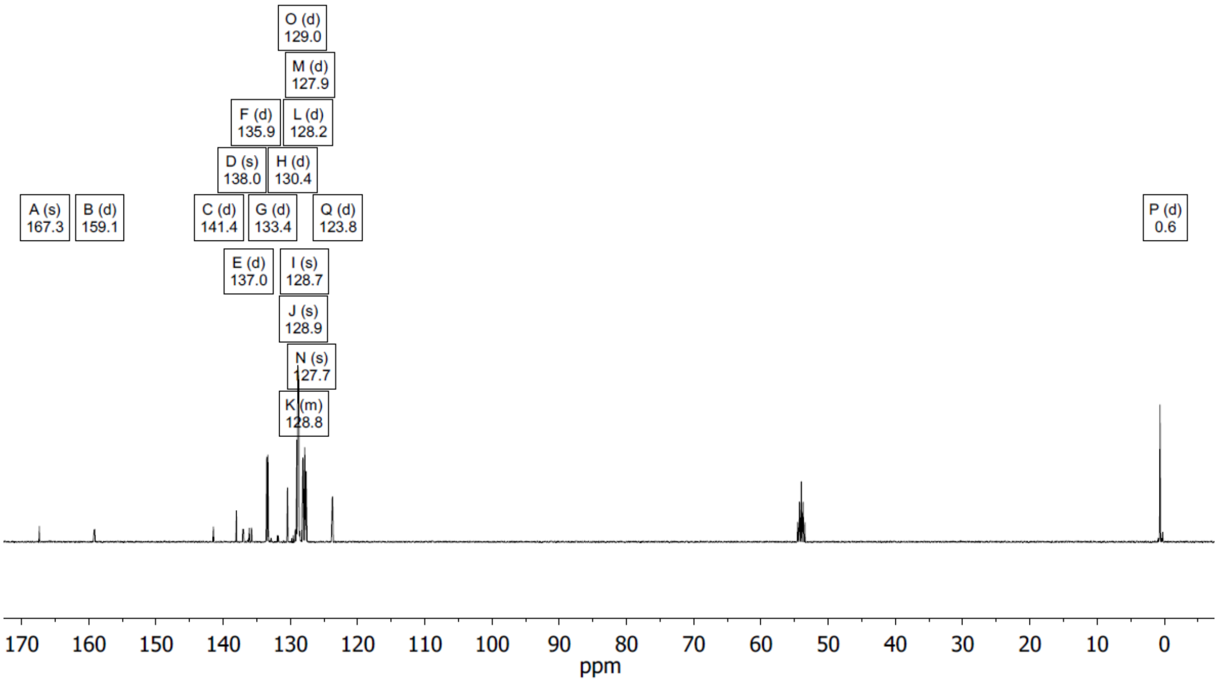

Fig. S8: $^{29}Si\{^1H\}$ NMR (CD_2Cl_2, 99 MHz)

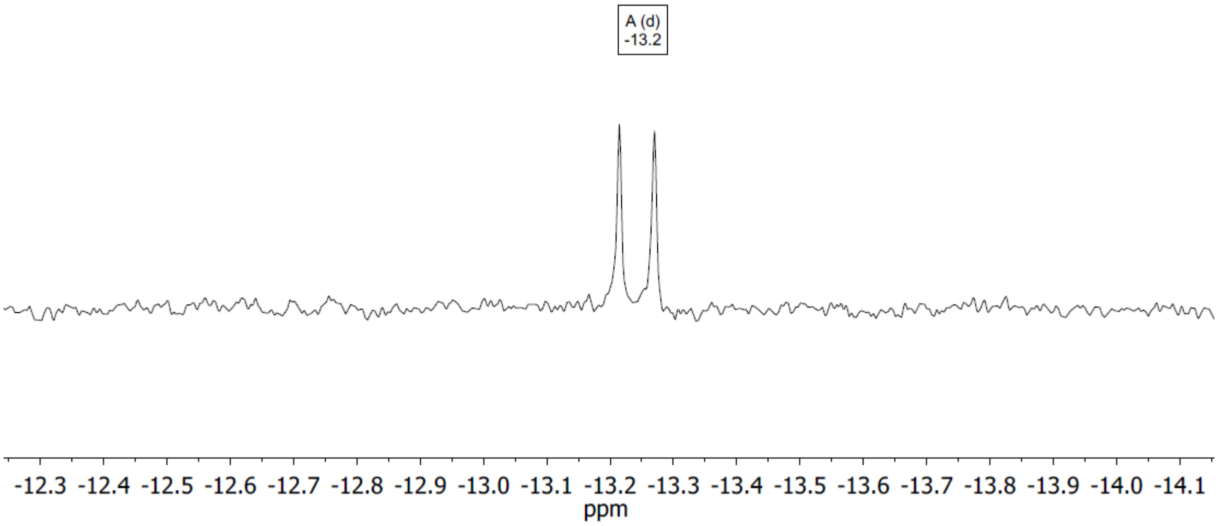

Fig. S9: $^{31}P\{^1H\}$ NMR (CD_2Cl_2, 202 MHz)

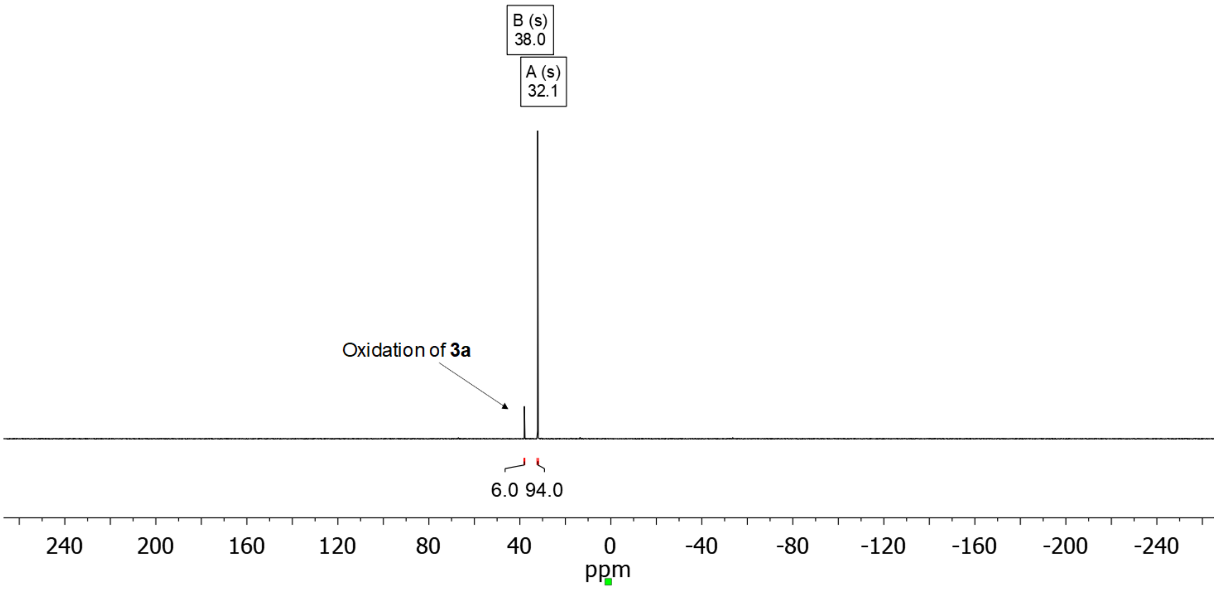

NMR spectra for compound **3b** and **3b'**

Fig. S10: ^{1}H NMR (CD_2Cl_2, 400 MHz)

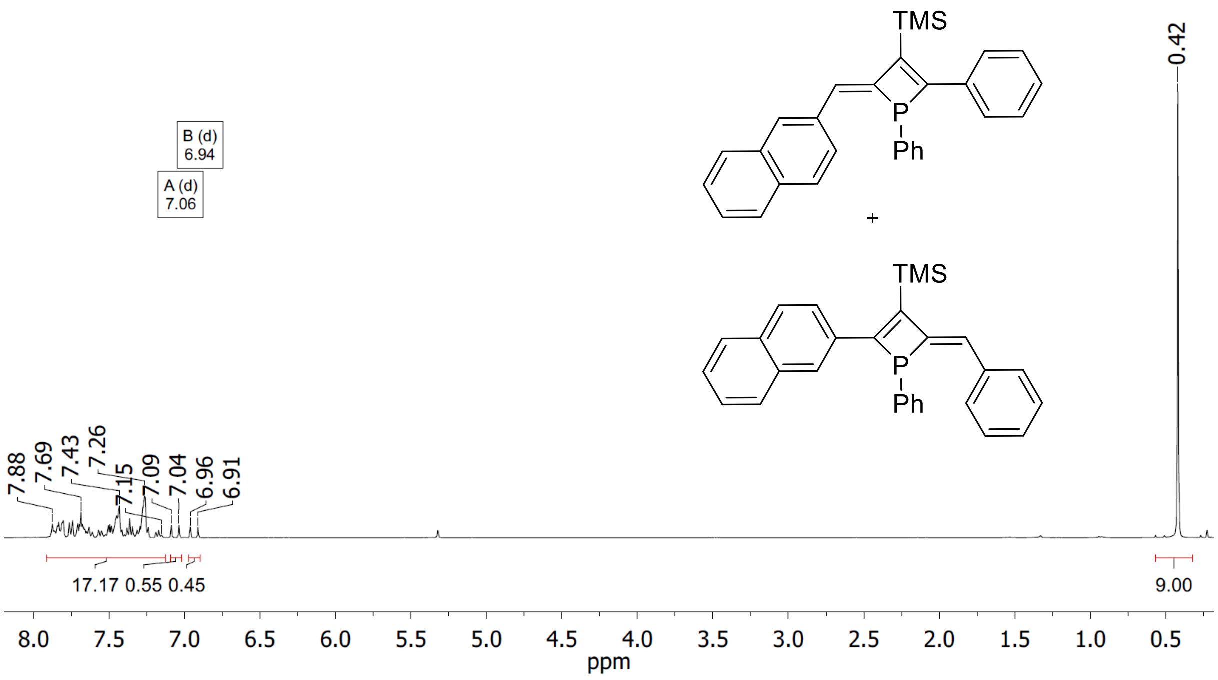

Fig. S11: ^{13}C NMR (CD_2Cl_2, 101 MHz) – Overview spectrum

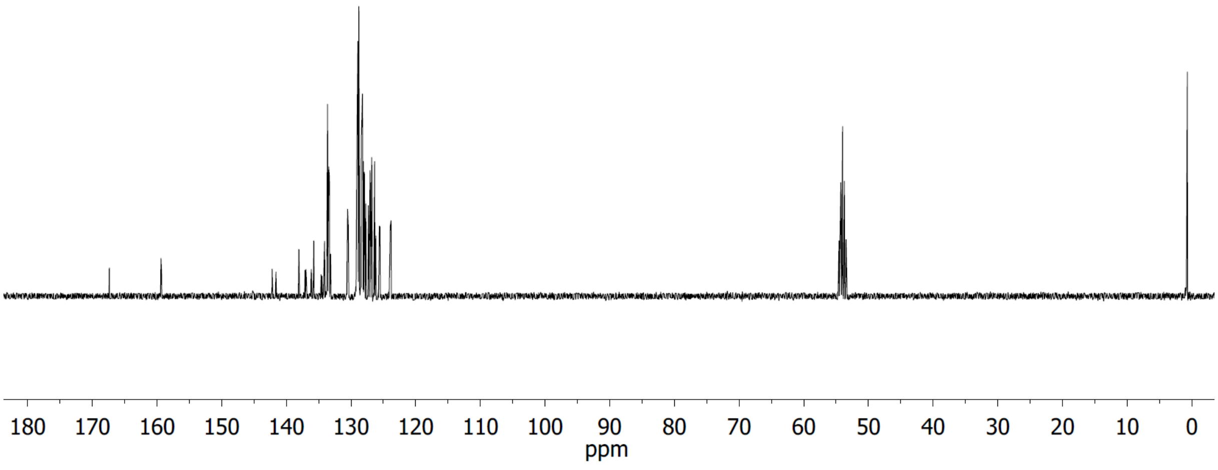

Fig. S12: ^{13}C NMR (CD_2Cl_2, 101 MHz) – 143–123 ppm

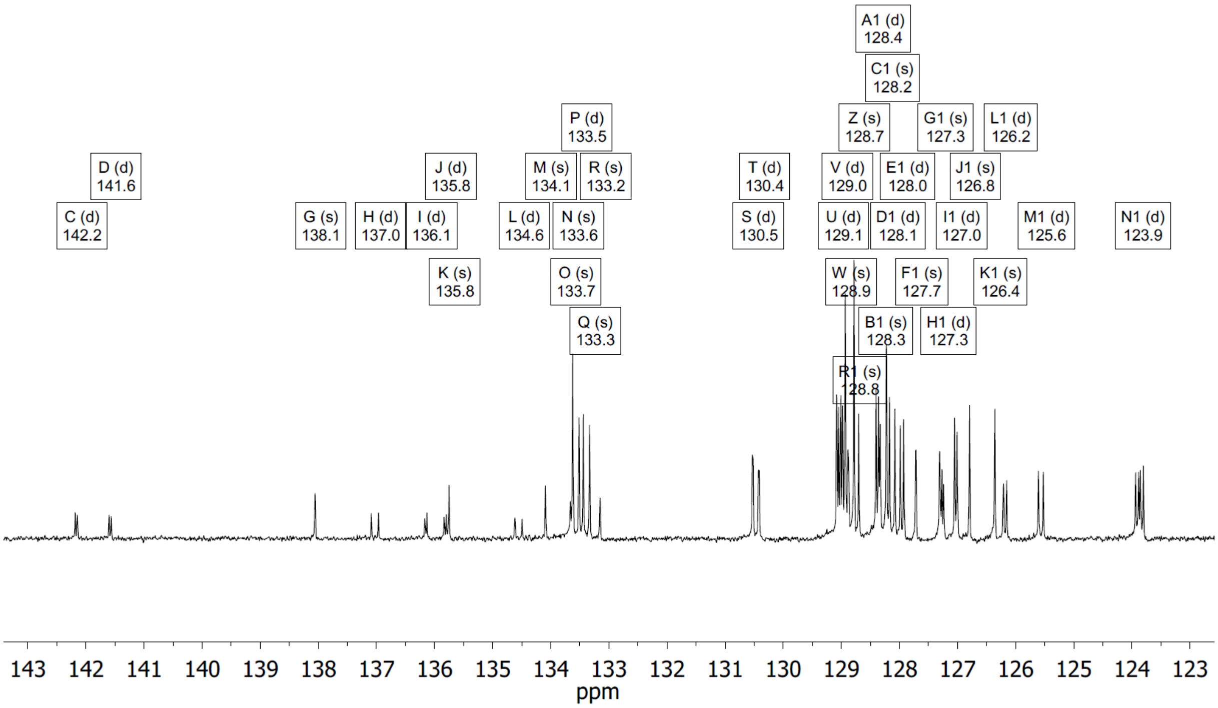

Fig. S13: ^{29}Si{^{1}H} NMR (CD_2Cl_2, 99 MHz)

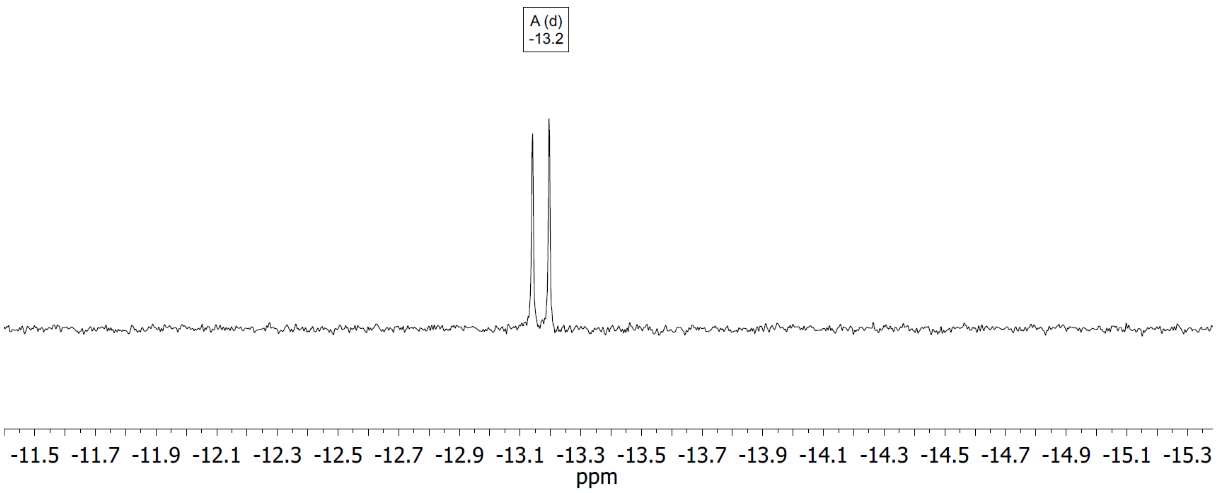

Fig. S14: $^{31}P\{^{1}H\}$ NMR (CD_2Cl_2, 202 MHz)

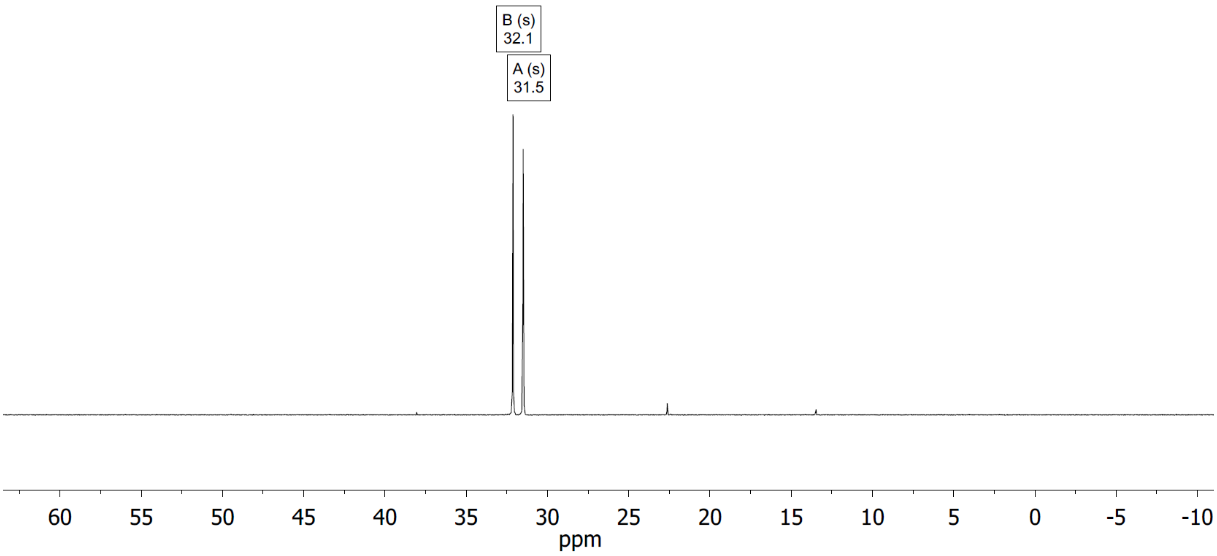

NMR spectra for compound **3c**

Fig. S15: ^{1}H NMR (CD_2Cl_2, 400 MHz)

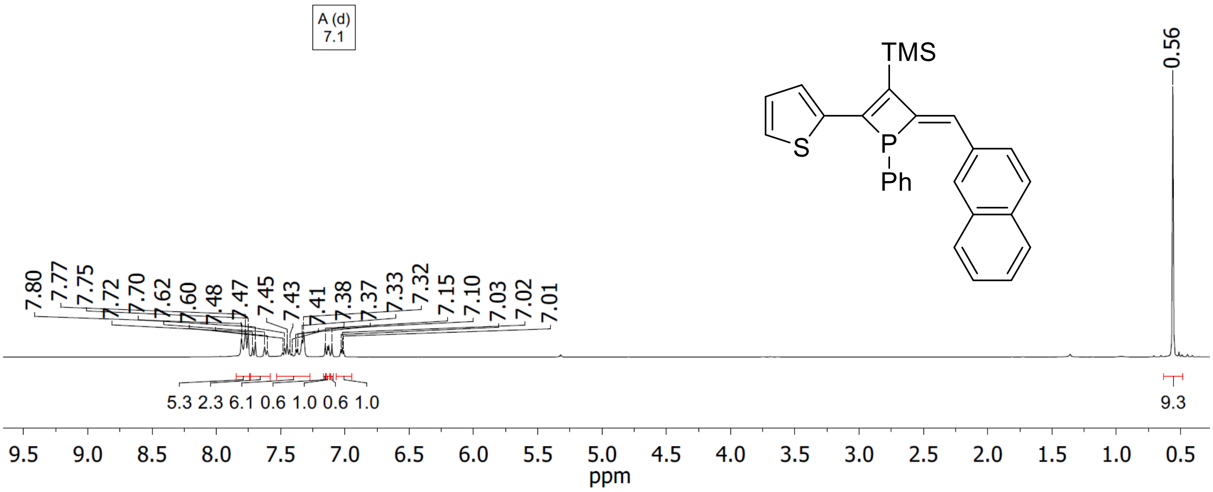

Fig. S16: ^{13}C NMR (CD_2Cl_2, 101 MHz)

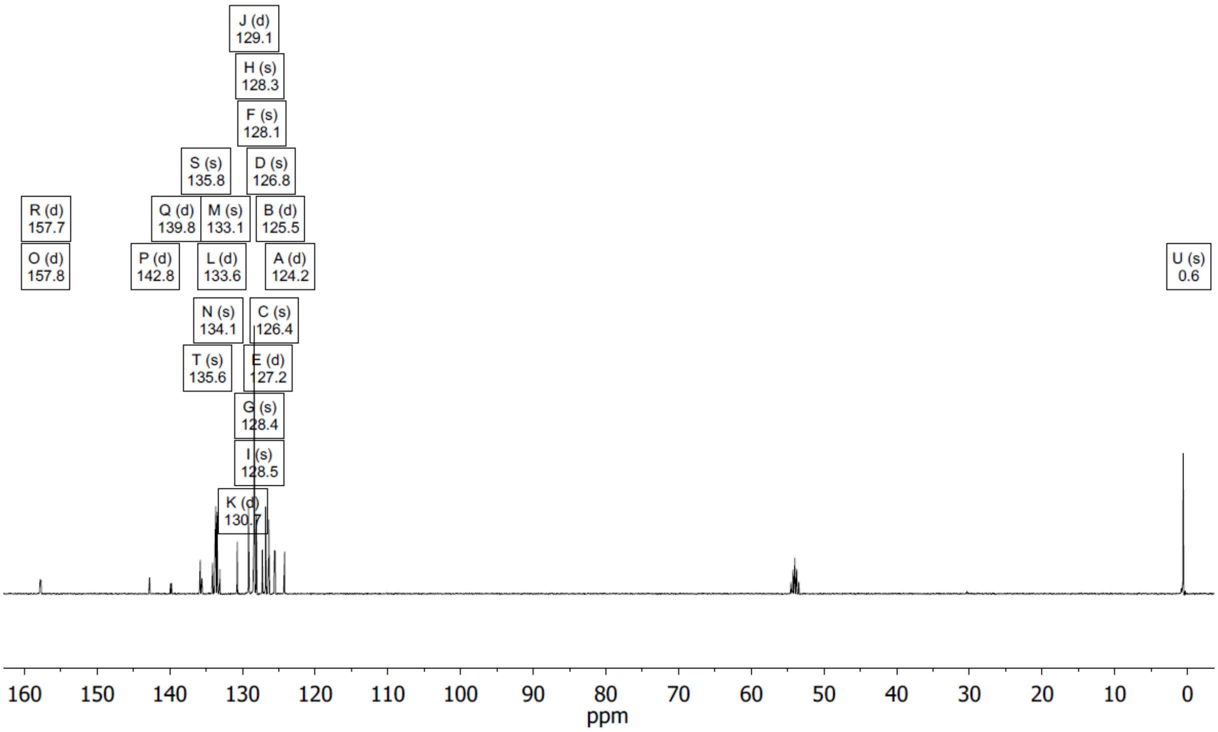

Fig. S17: ^{29}Si{^{1}H} NMR (CD_2Cl_2, 99 MHz)

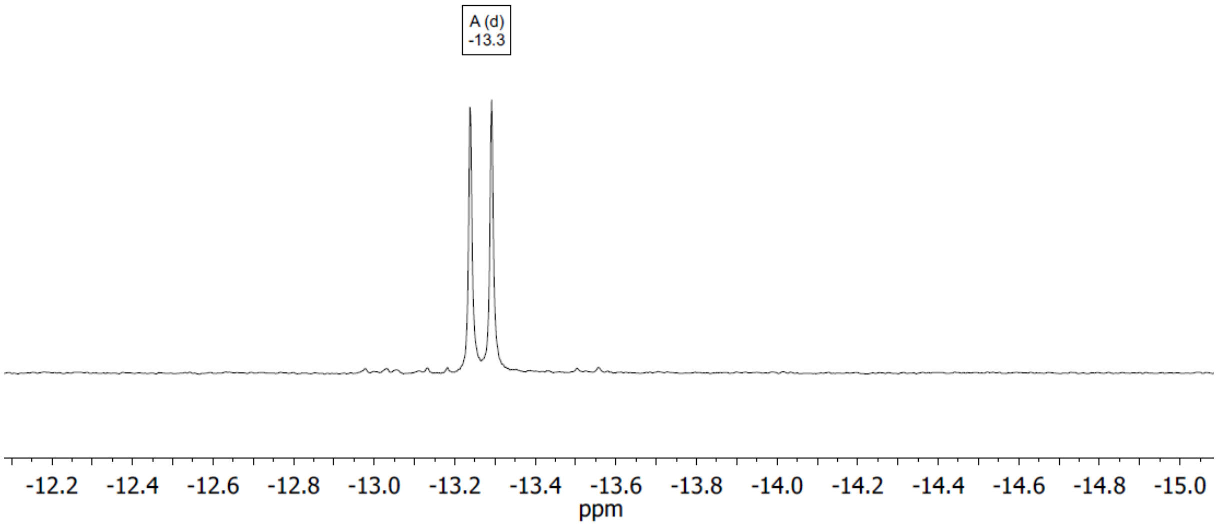

Fig. S18: $^{31}P\{^1H\}$ NMR (CD_2Cl_2, 202 MHz)

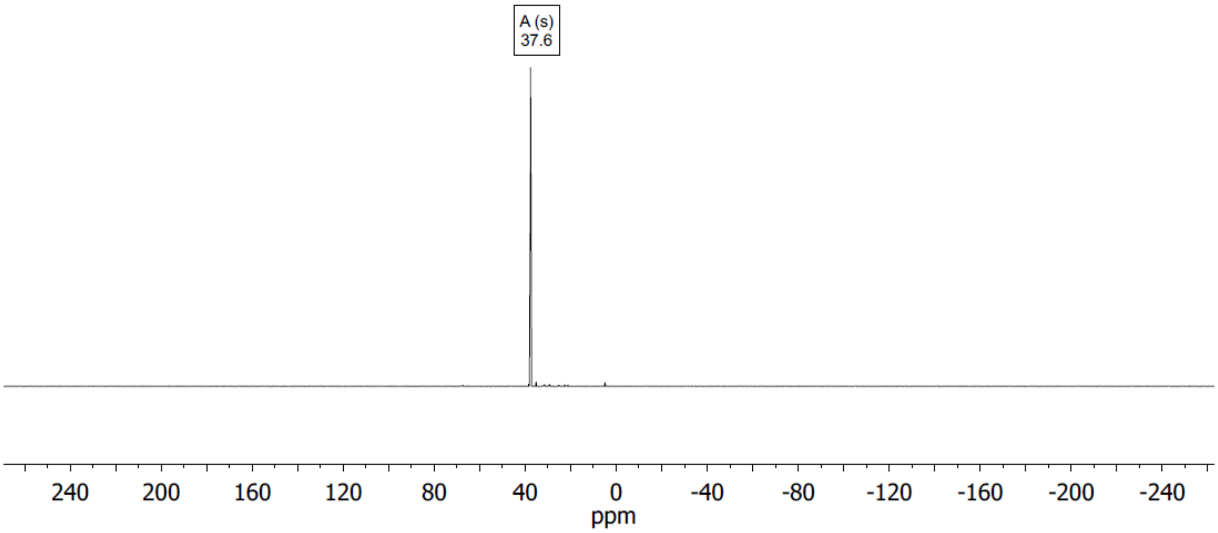

NMR spectra for compound **2b** and **2b’**

Fig. S19: ^{1}H NMR (CD_2Cl_2, 400 MHz)

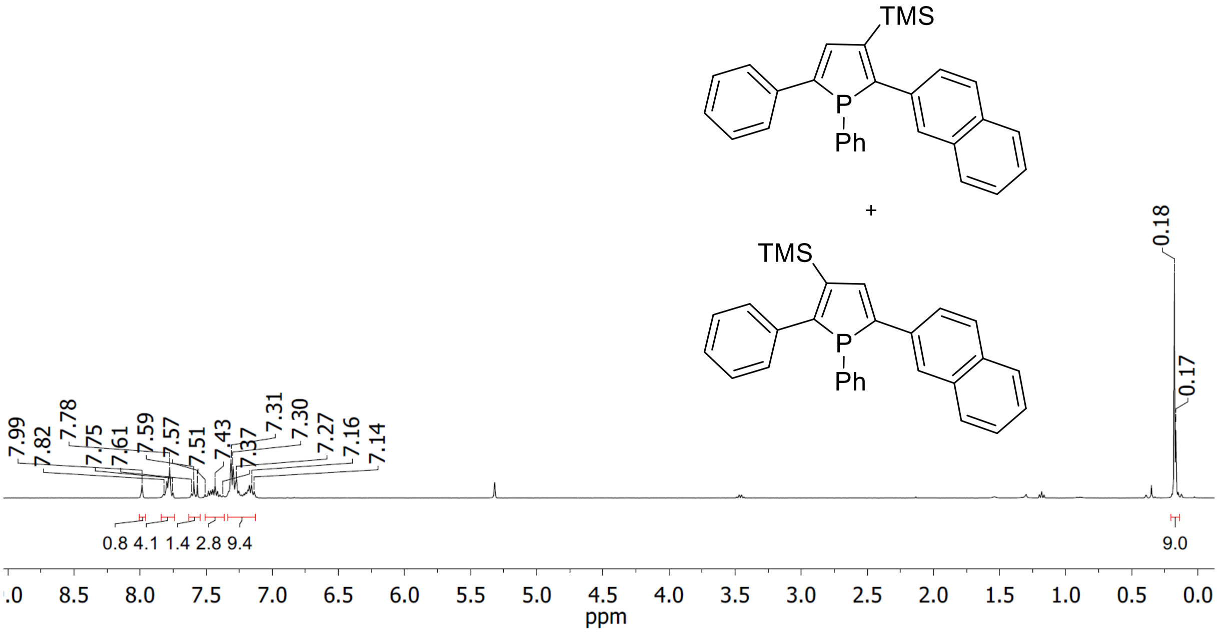

Fig. S20: ^{13}C NMR (CD_2Cl_2, 101 MHz) – Overview spectrum

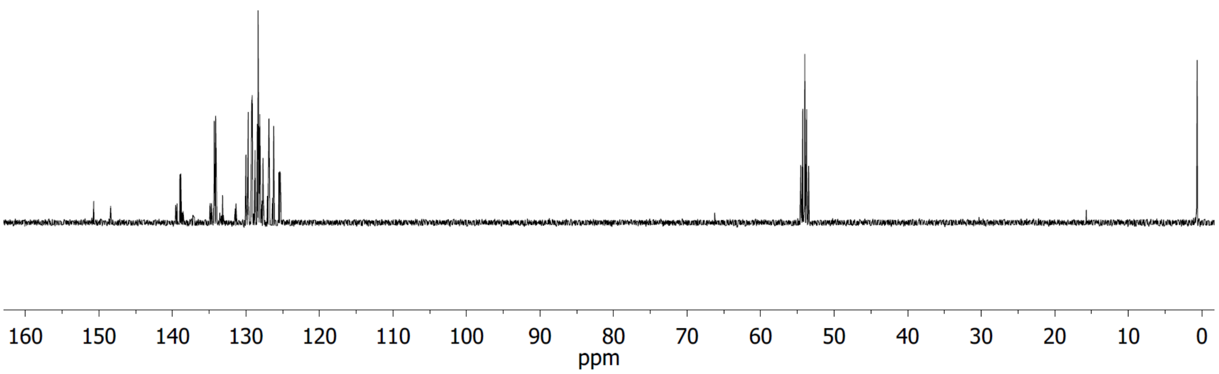

Fig. S21: ^{13}C NMR (CD_2Cl_2, 101 MHz) – 141 – 121ppm

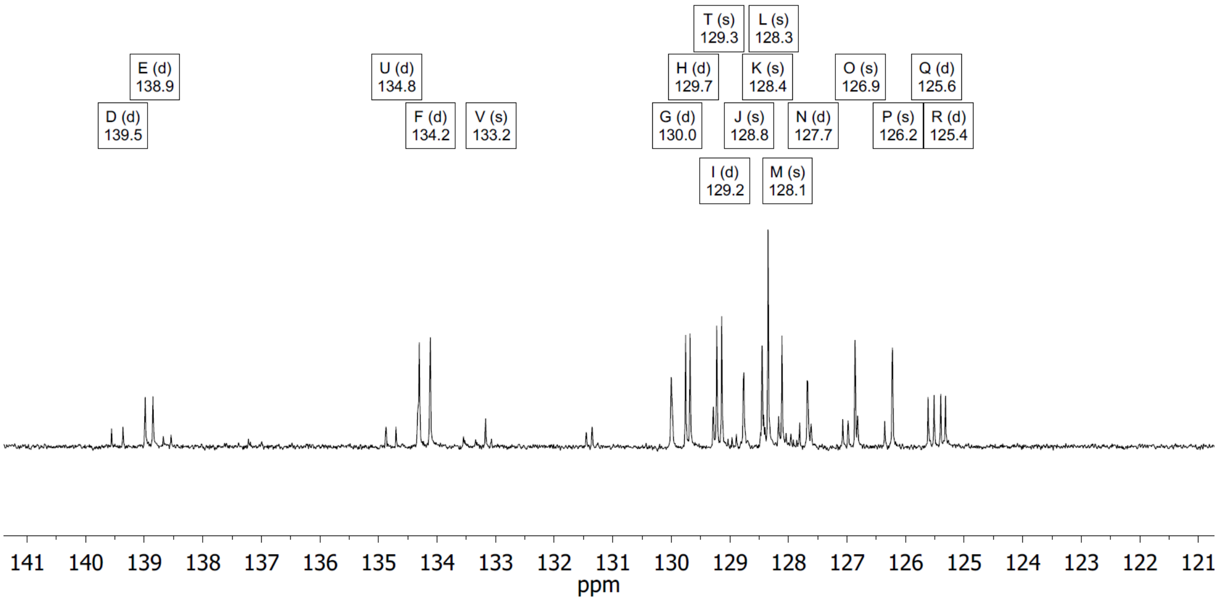

Fig. S22: $^{29}Si\{^1H\}$ NMR (CD_2Cl_2, 99 MHz)

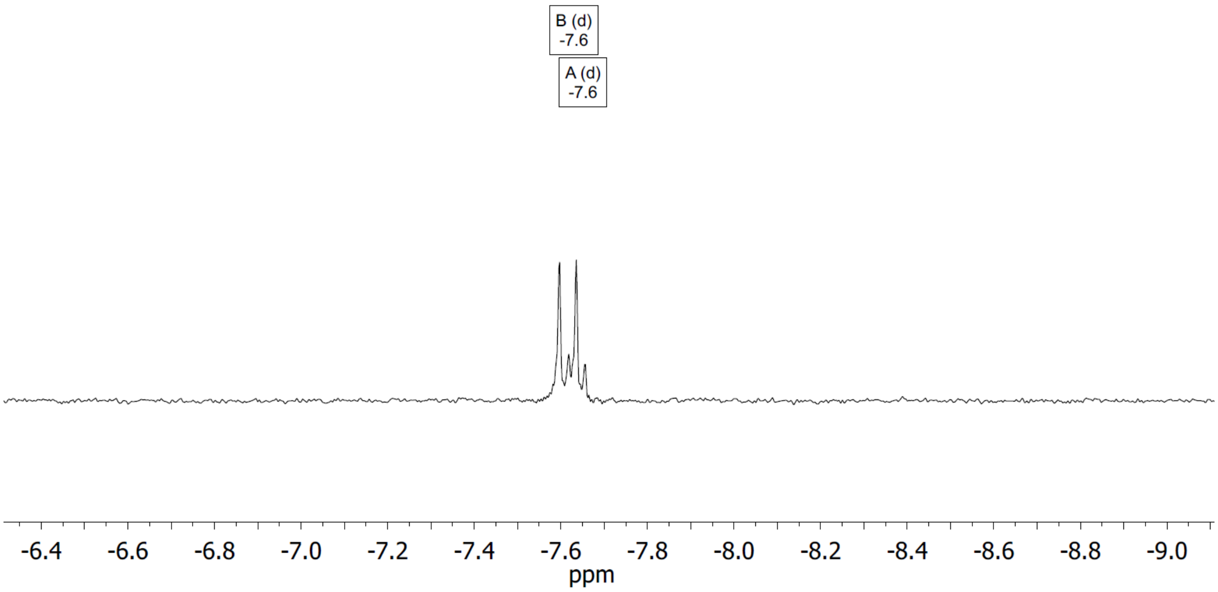

Fig. S23: $^{31}P\{^{1}H\}$ NMR (CD_2Cl_2, 202 MHz)

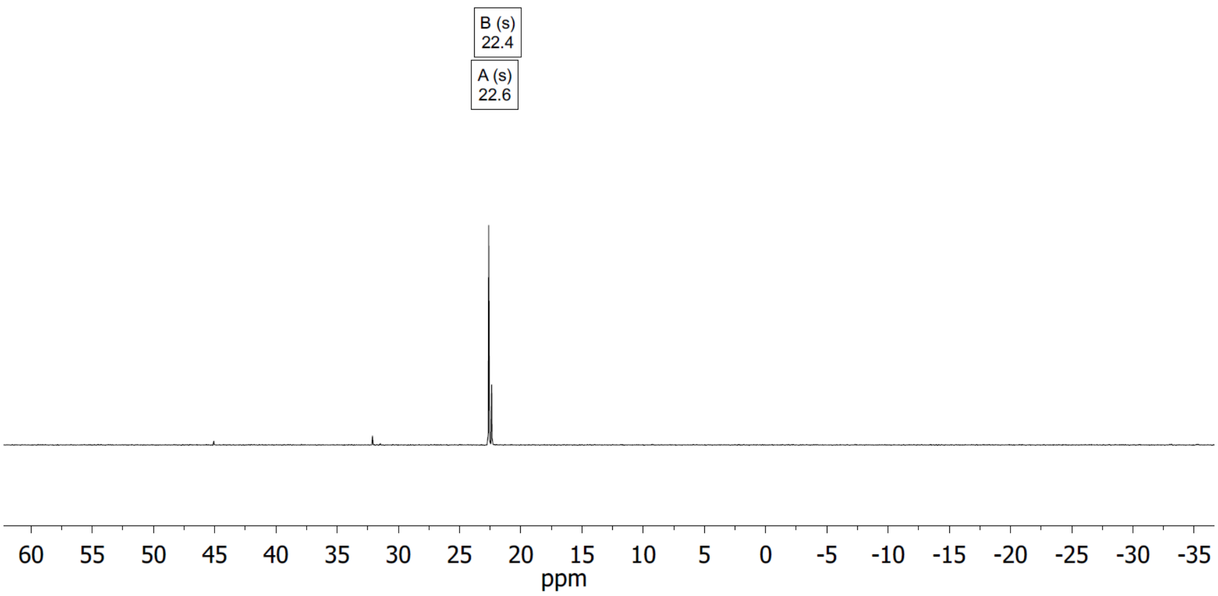

NMR spectra for compound **2c** and **2c'**

Fig. S24: ^{1}H NMR (CD_2Cl_2, 400 MHz)

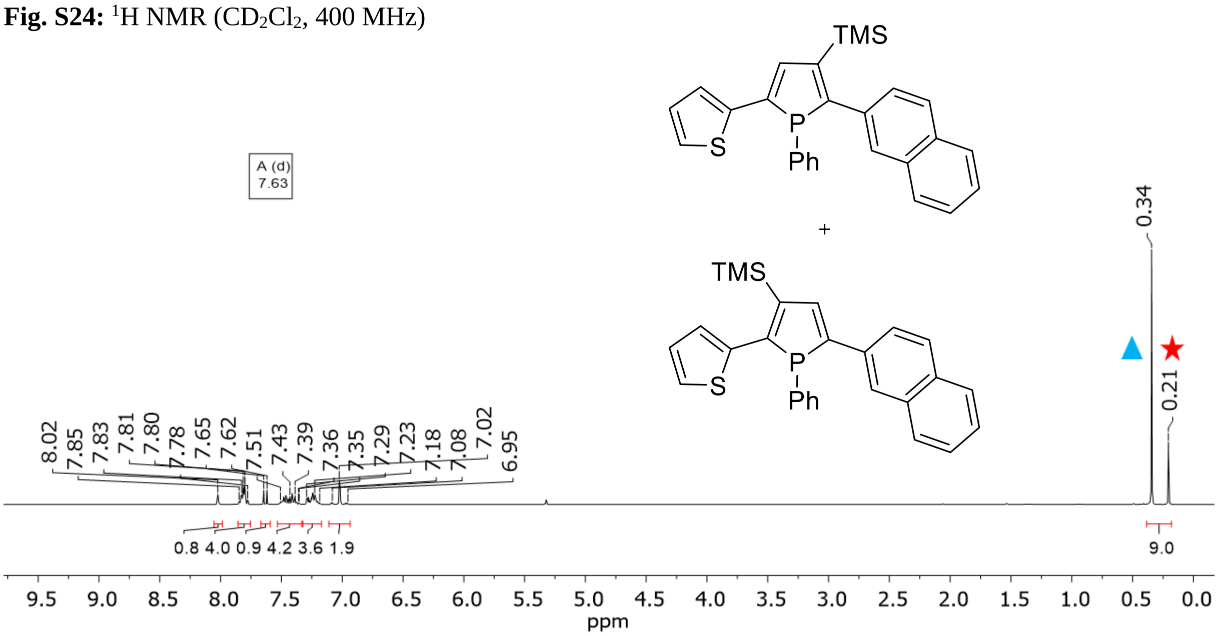

Fig. S25: ^{13}C NMR (CD_2Cl_2, 101 MHz) – Overview spectrum

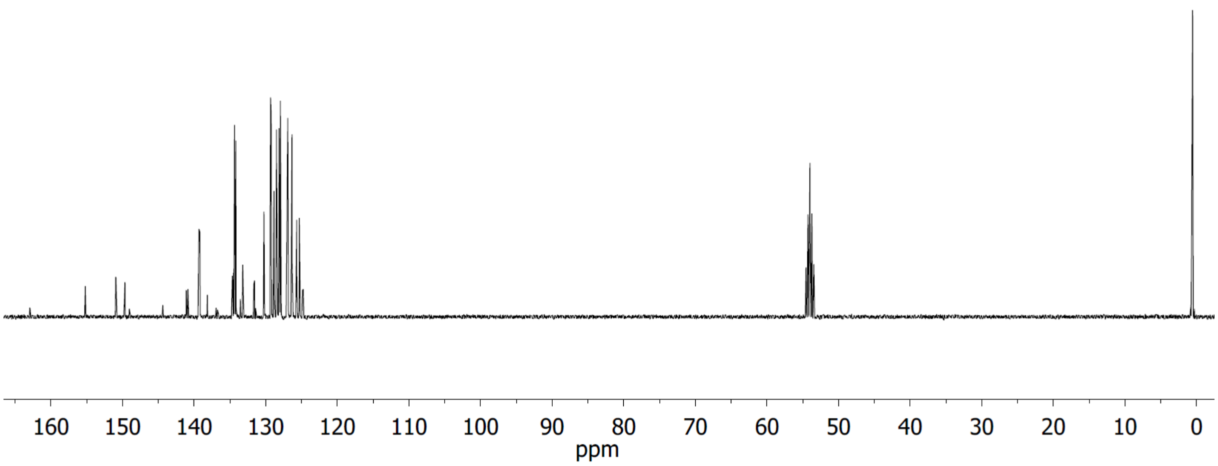

Fig. S26: ^{13}C NMR (CD_2Cl_2, 101 MHz) – 164–137 ppm

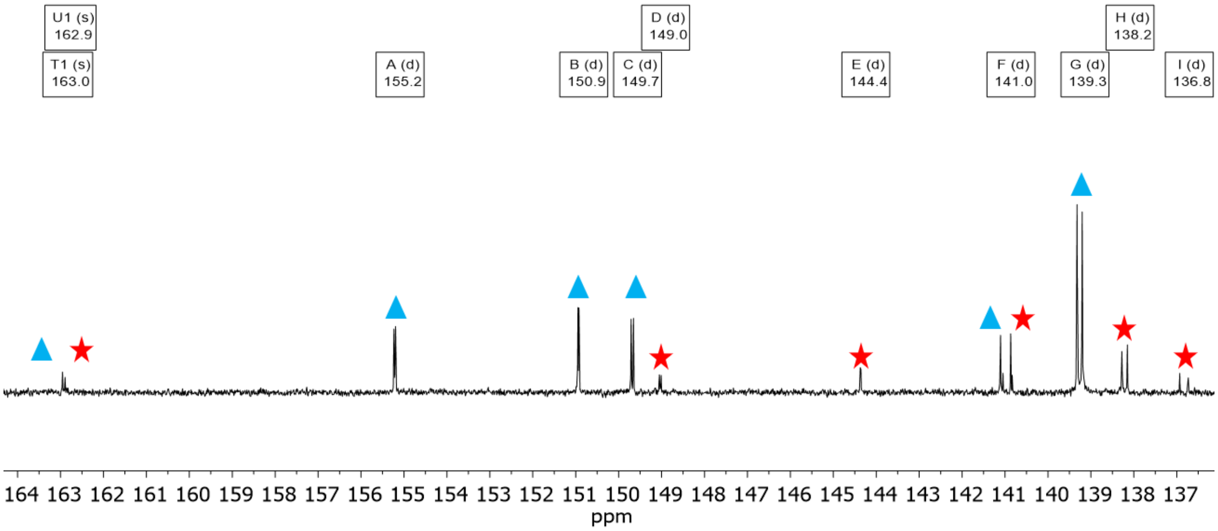

Fig. S27: ^{13}C NMR (CD_2Cl_2, 101 MHz) – 135–125 ppm

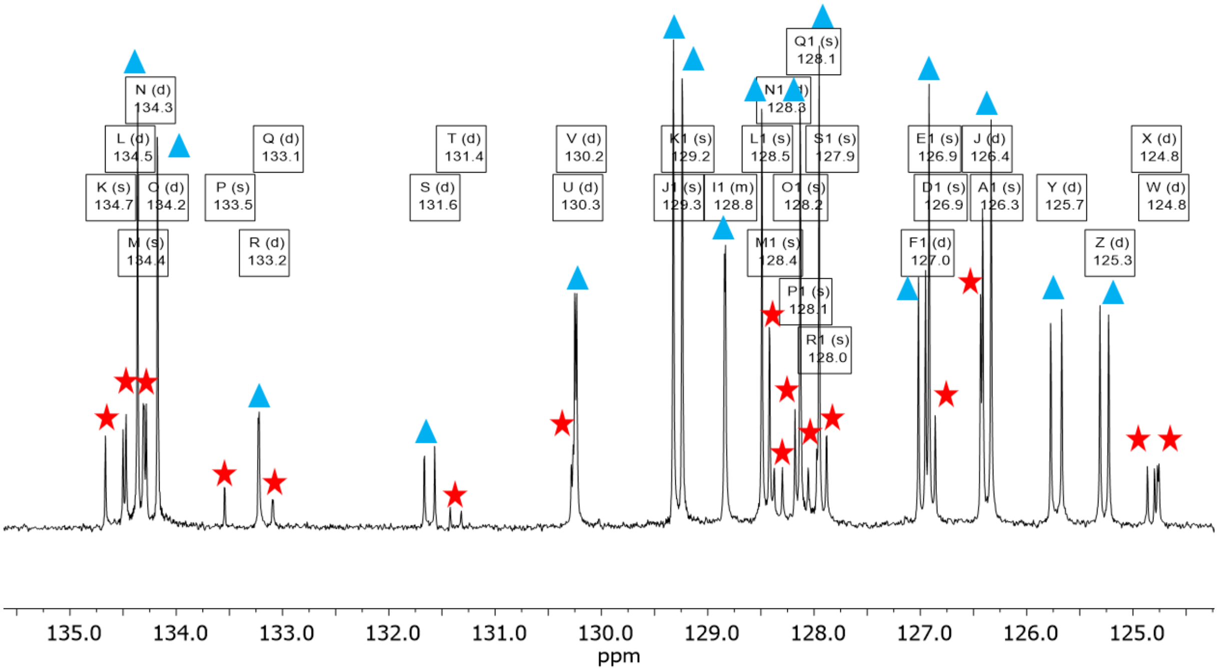

Fig. S28: ^{13}C NMR (CD_2Cl_2, 101 MHz)

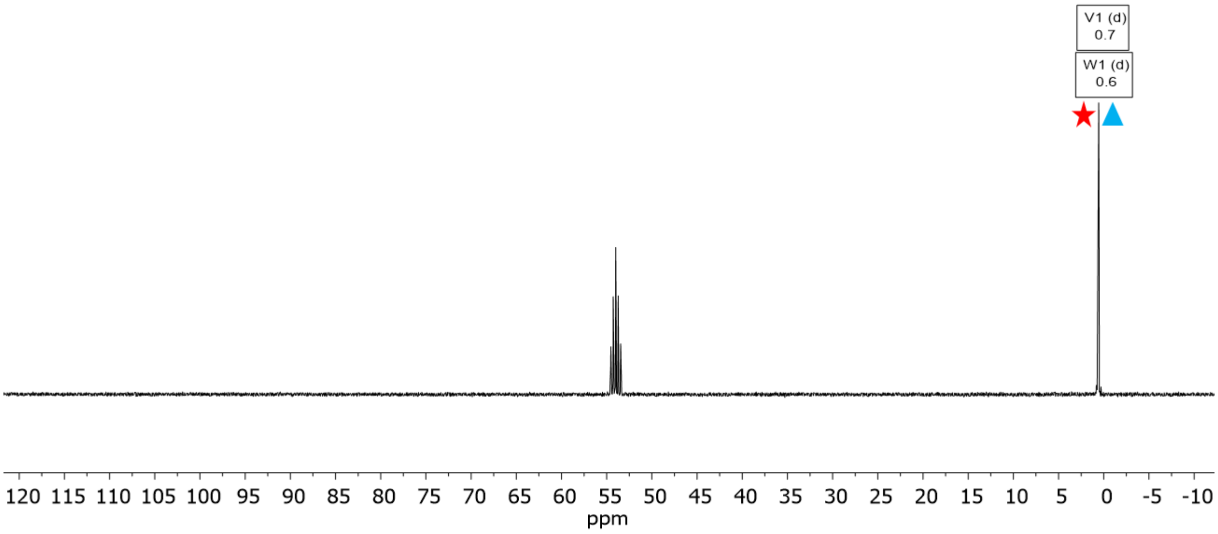

Fig. S29: ^{29}Si{^{1}H} NMR (CD_2Cl_2, 99 MHz)

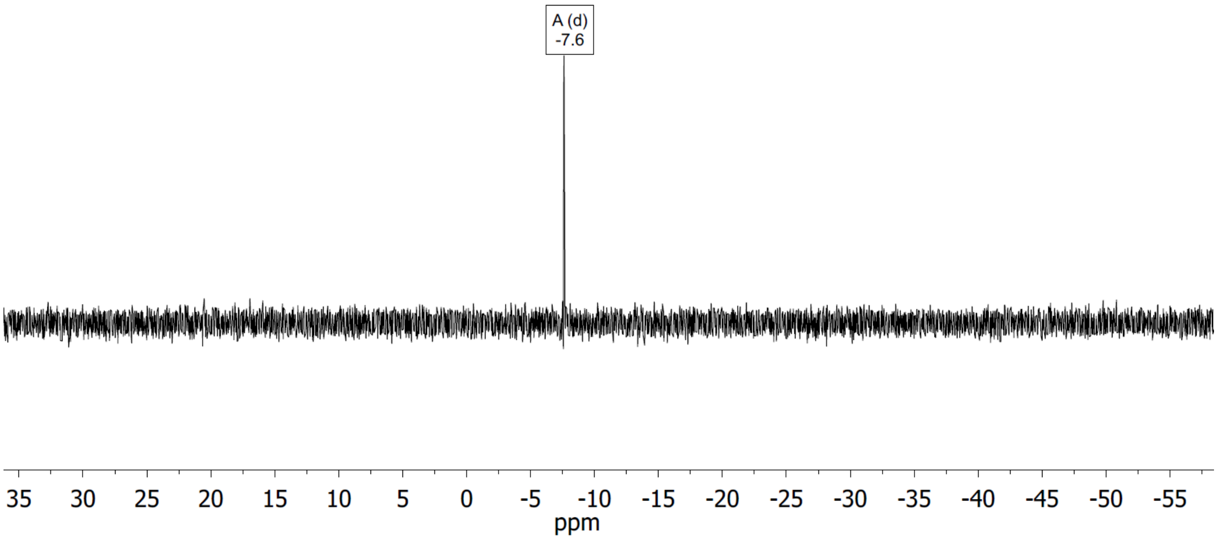

Fig. S30: $^{31}P\{^{1}H\}$NMR (CD_2Cl_2, 202 MHz)

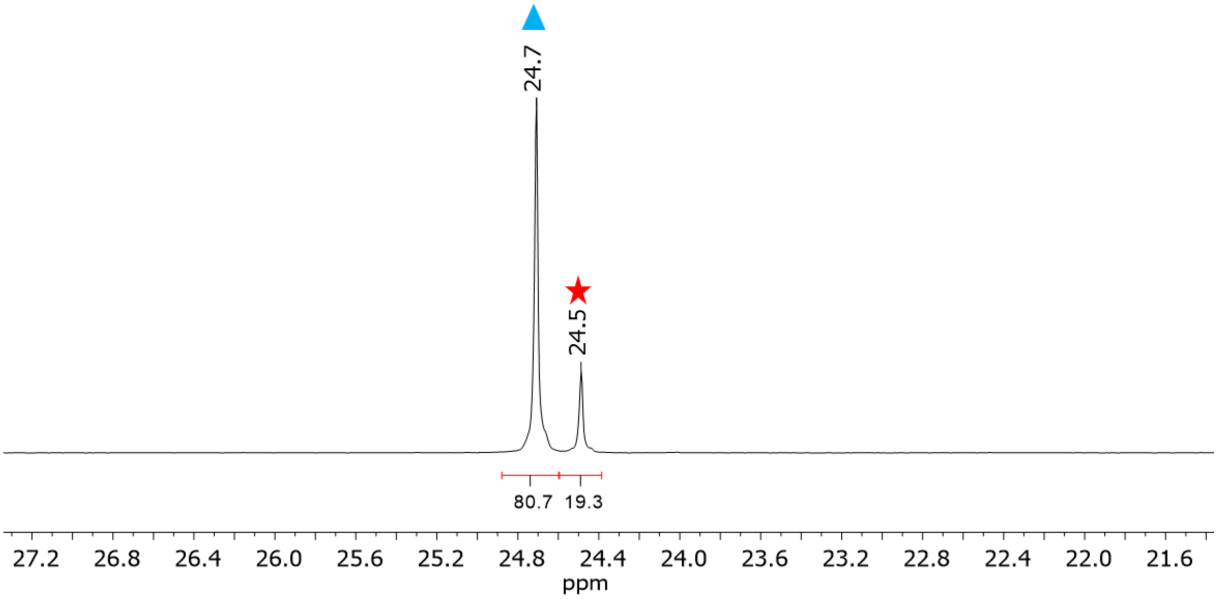

NMR spectra for compound **4b**

Fig. S31: ^{1}H NMR (CD_2Cl_2, 400 MHz)

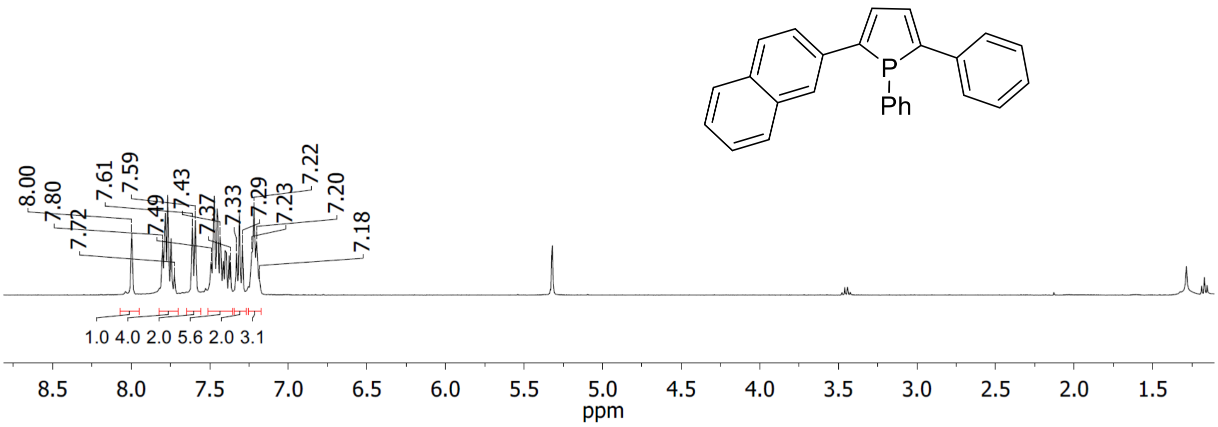

Fig. S32: ^{13}C NMR (CD_2Cl_2, 101 MHz)

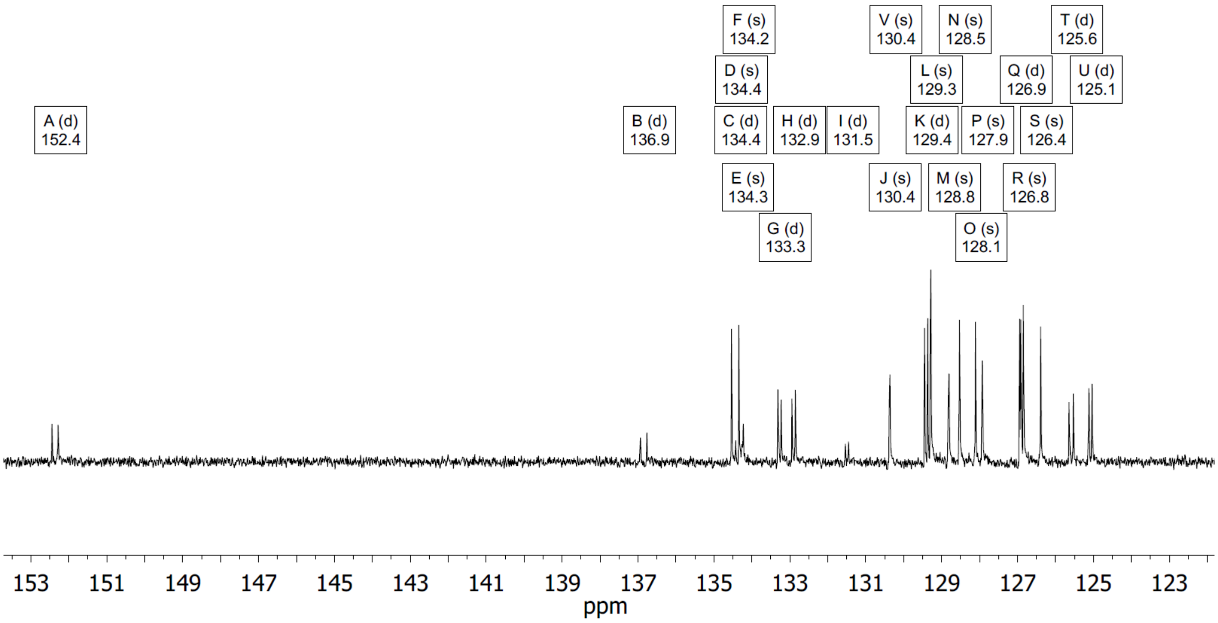

Fig. S33: $^{31}P\{^{1}H\}$ NMR (CD_2Cl_2, 202 MHz)

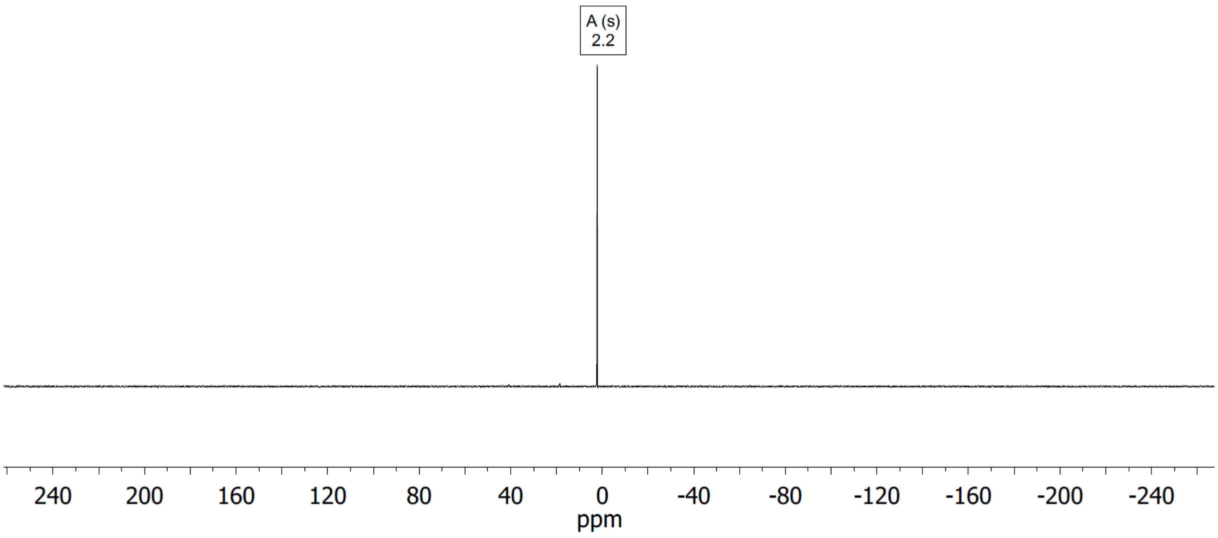

NMR spectra for compound **4c**

Fig. S34: ^{1}H NMR (CD_2Cl_2, 400 MHz)

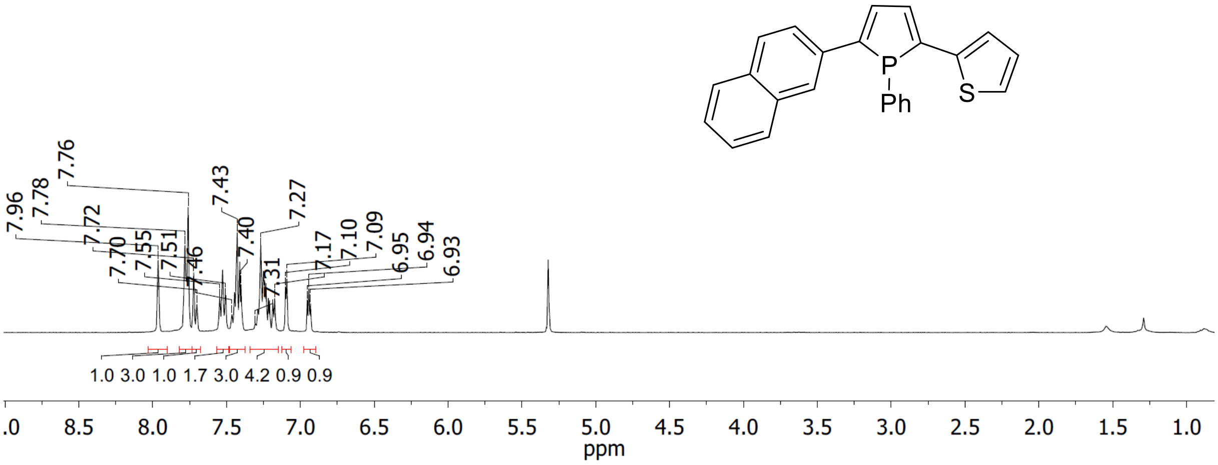

Fig. S35: ^{13}C NMR (CD_2Cl_2, 101 MHz)

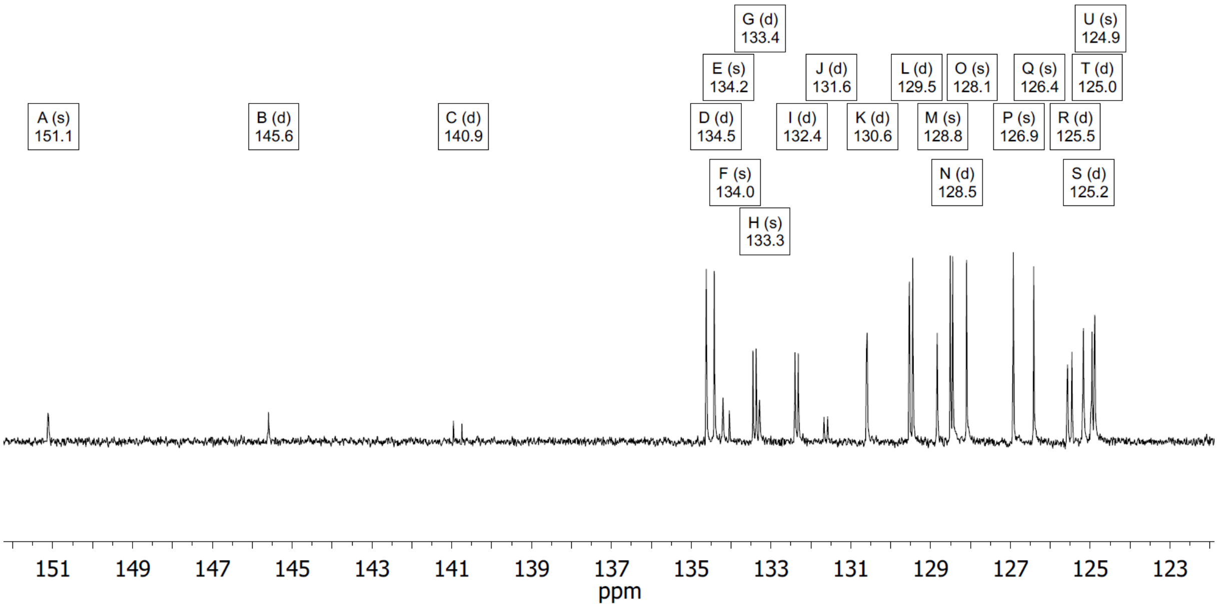

Fig. S36: $^{31}P\{^{1}H\}$ NMR (CD_2Cl_2, 202 MHz)

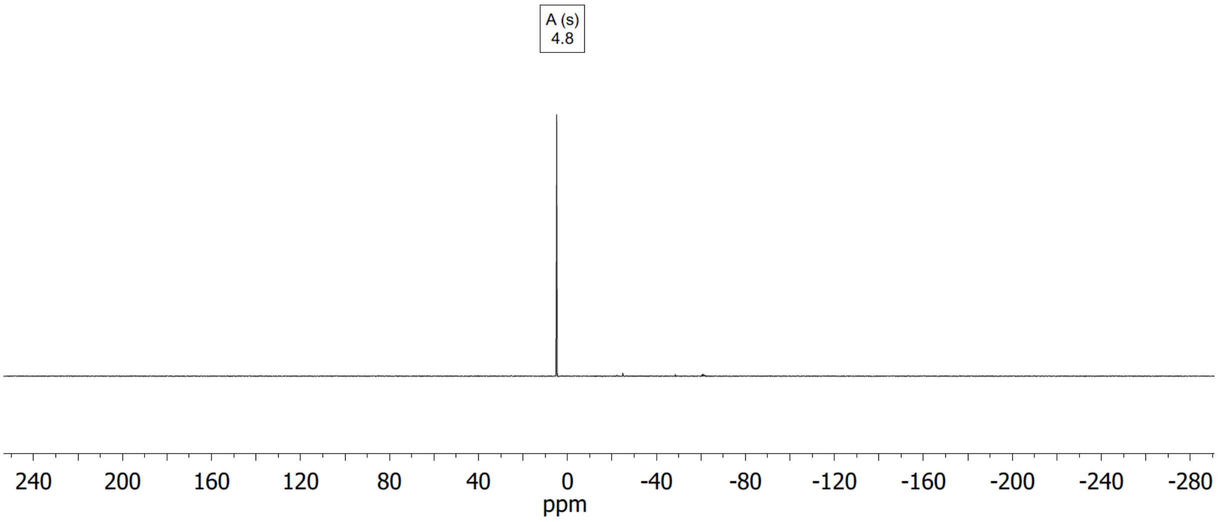

NMR spectra for compound **5a**

Fig. S37: ^{1}H NMR (THF-d_8, 400 MHz)

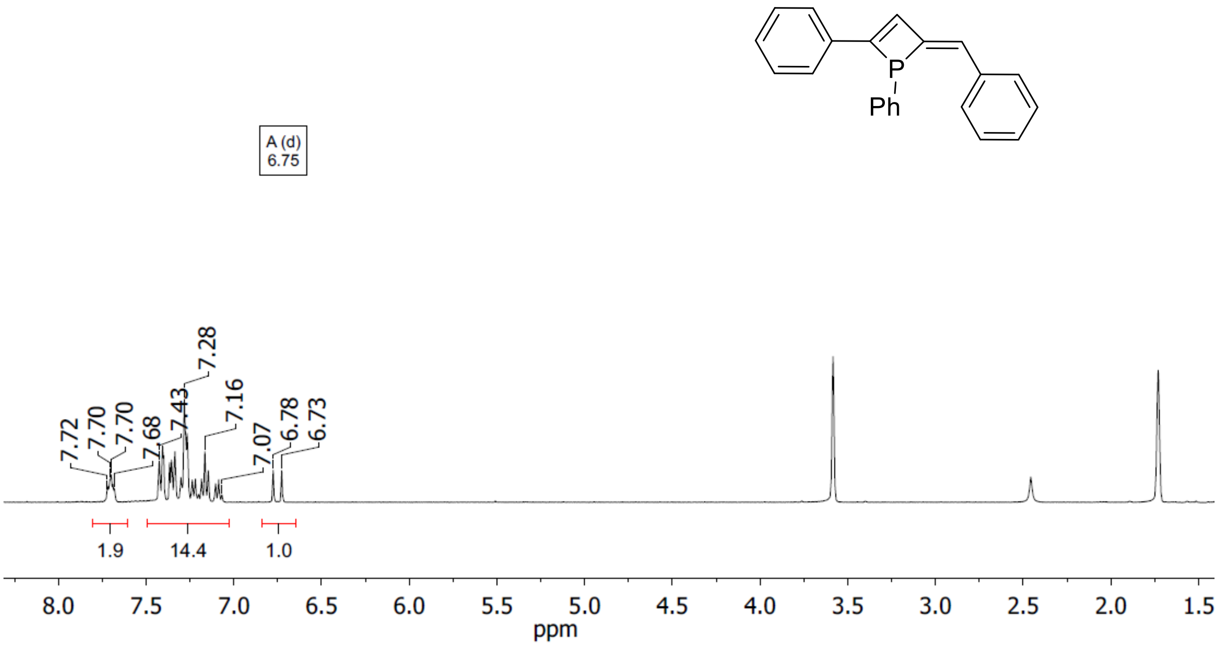

Fig. S38: ^{13}C NMR (THF-d_8, 101 MHz)

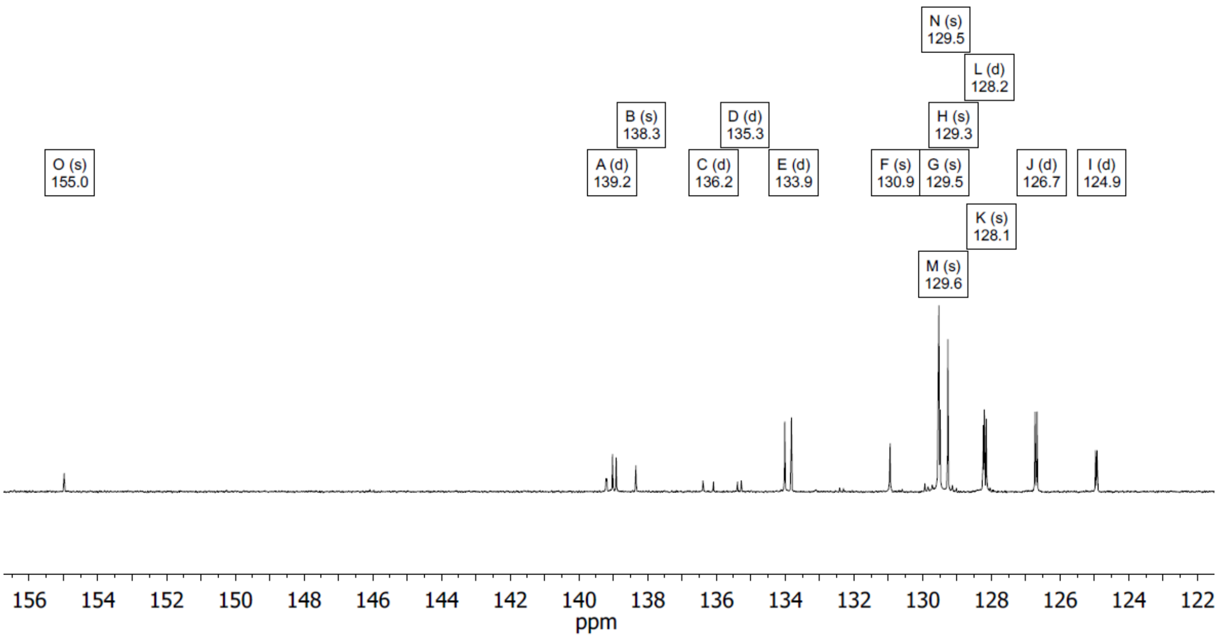

Fig. S39: $^{31}P\{^1H\}$ NMR (THF-d_8, 202 MHz)

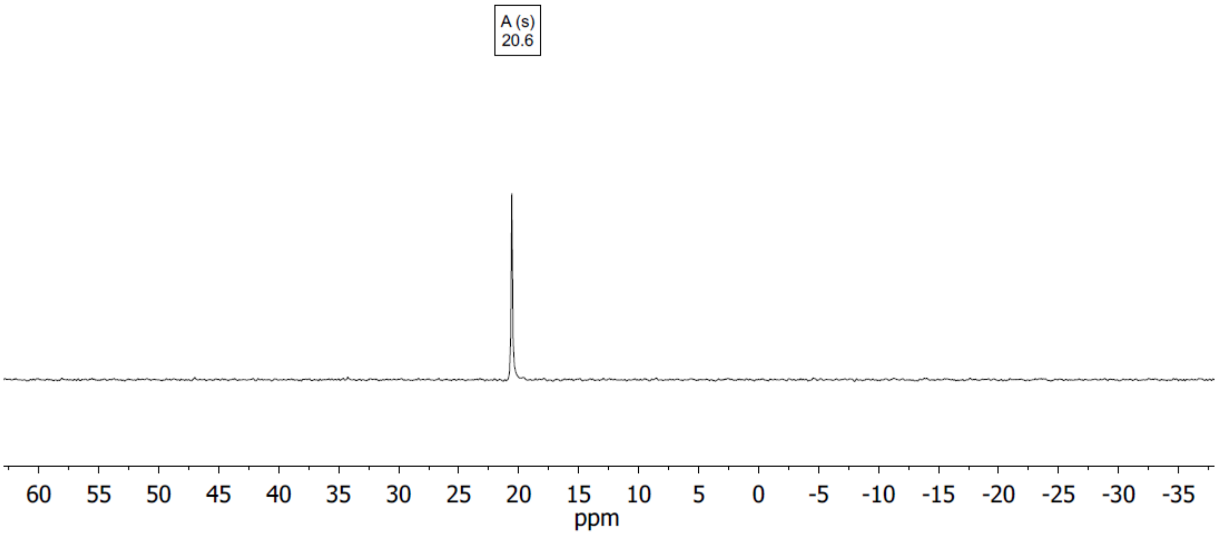

NMR spectra for compound **5b** and **5b'**

Fig. S40: ^1H NMR (CD_2Cl_2, 400 MHz)

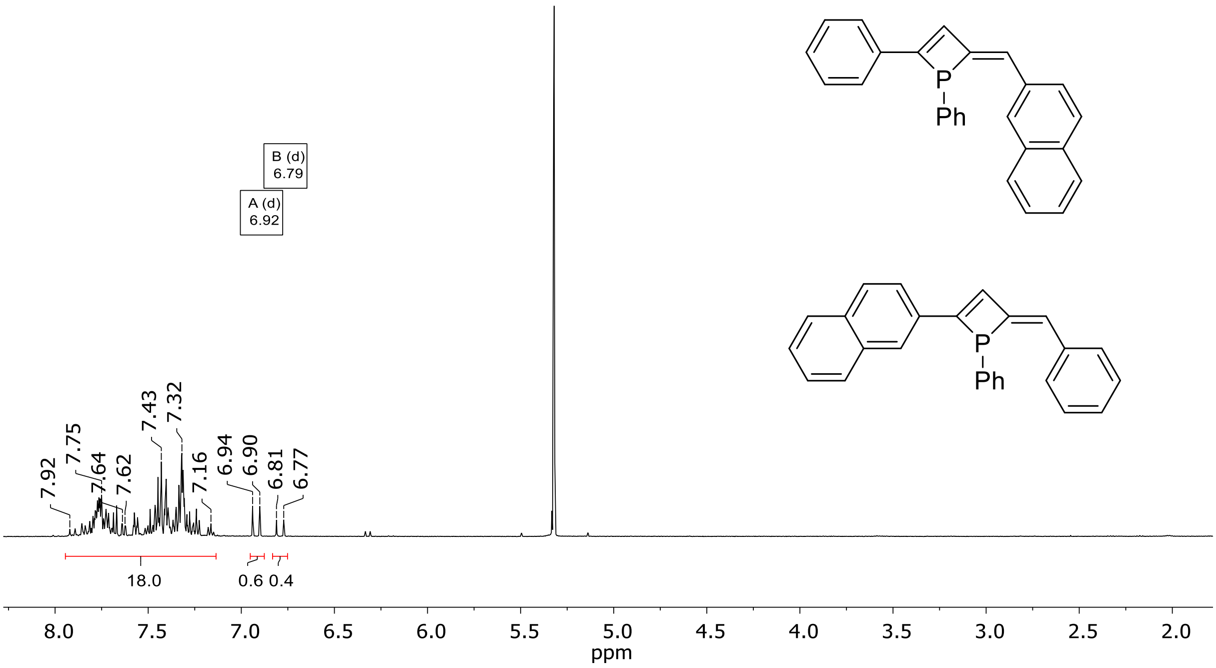

Fig. S41: ^{13}C NMR (CD_2Cl_2, 101 MHz)

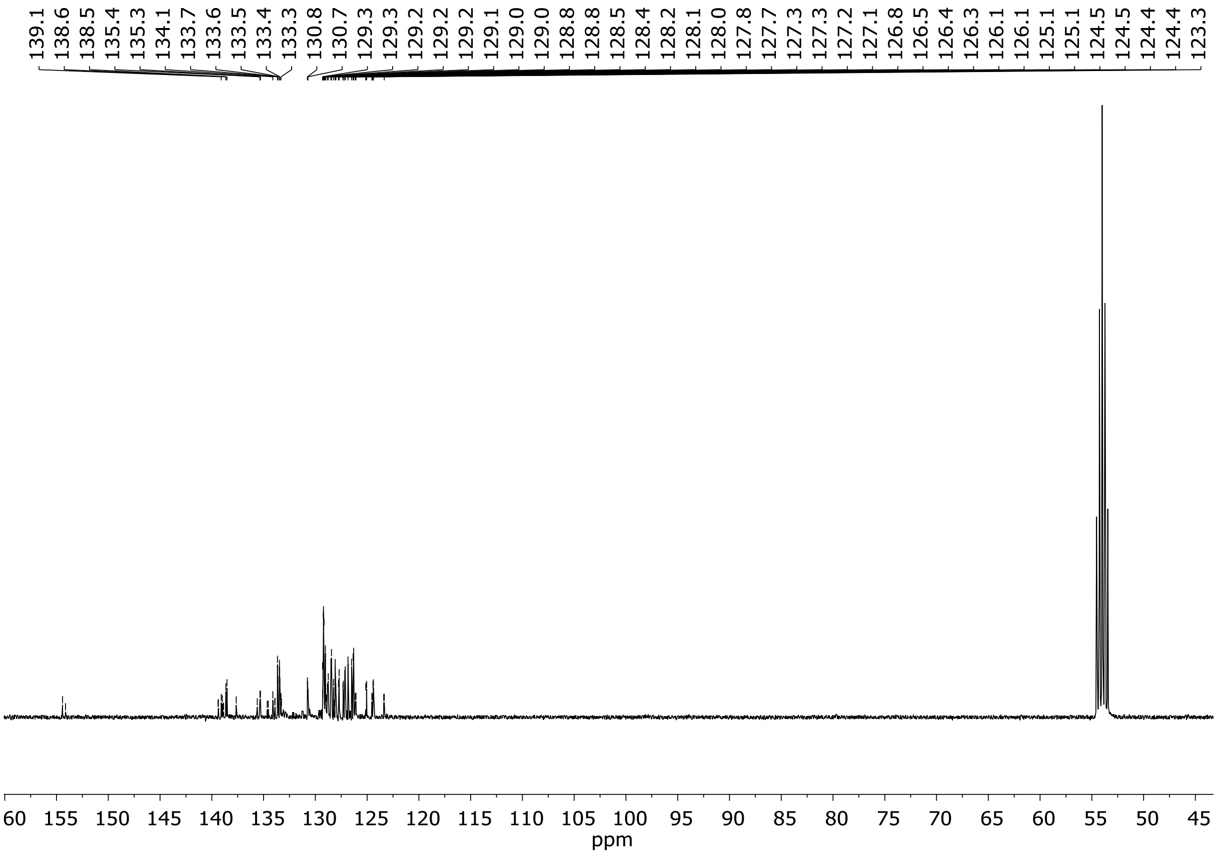

Fig. S42: ^{31}P{^{1}H} NMR (CD_2Cl_2, 202 MHz)

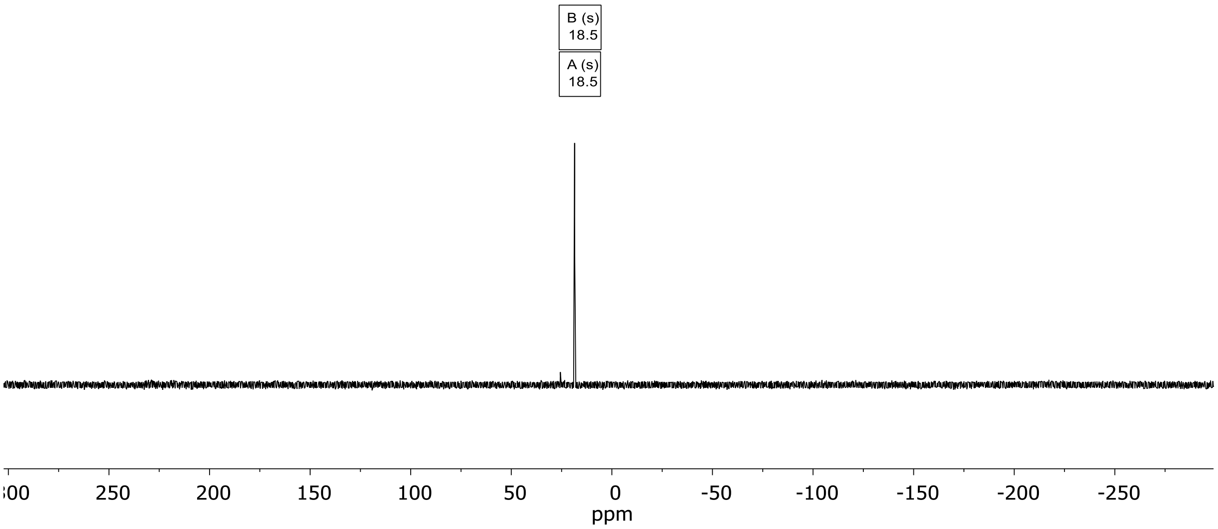

NMR spectra for compound **5c**

Fig. S42: ^{1}H NMR (CD_2Cl_2, 400 MHz)

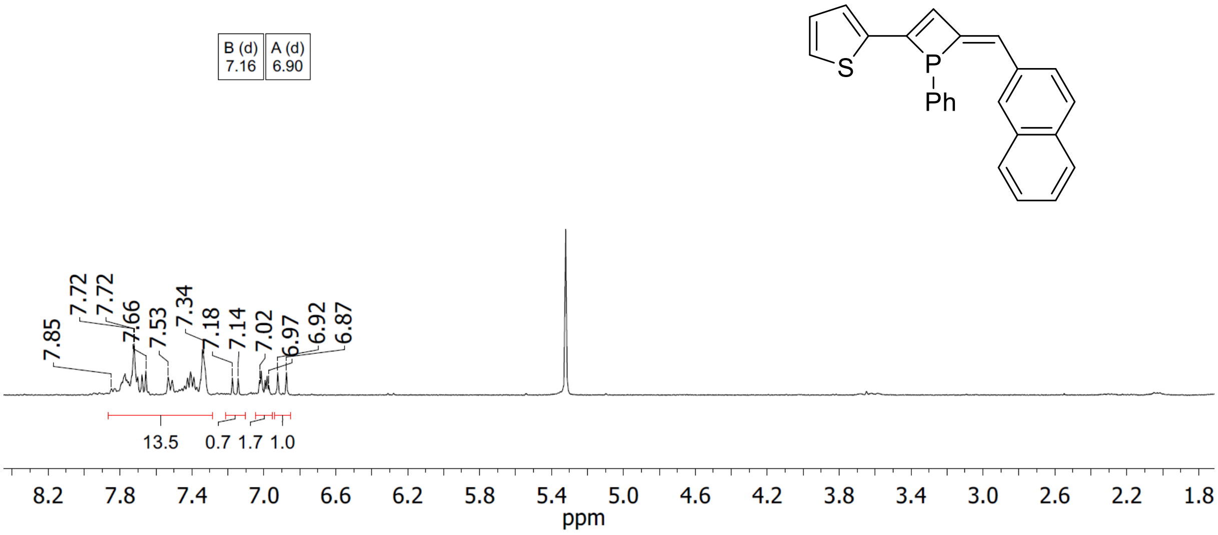

Fig. S43: ^{13}C NMR (CD_2Cl_2, 101 MHz)

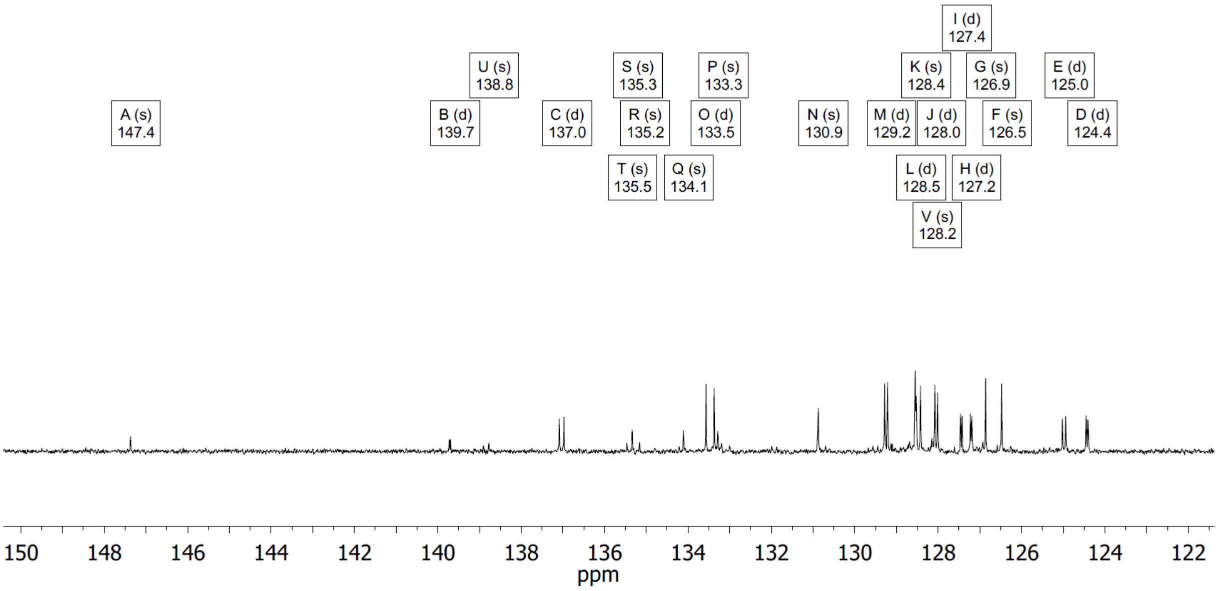

Fig. S44: $^{31}P\{^1H\}$ NMR (CD_2Cl_2, 202 MHz)

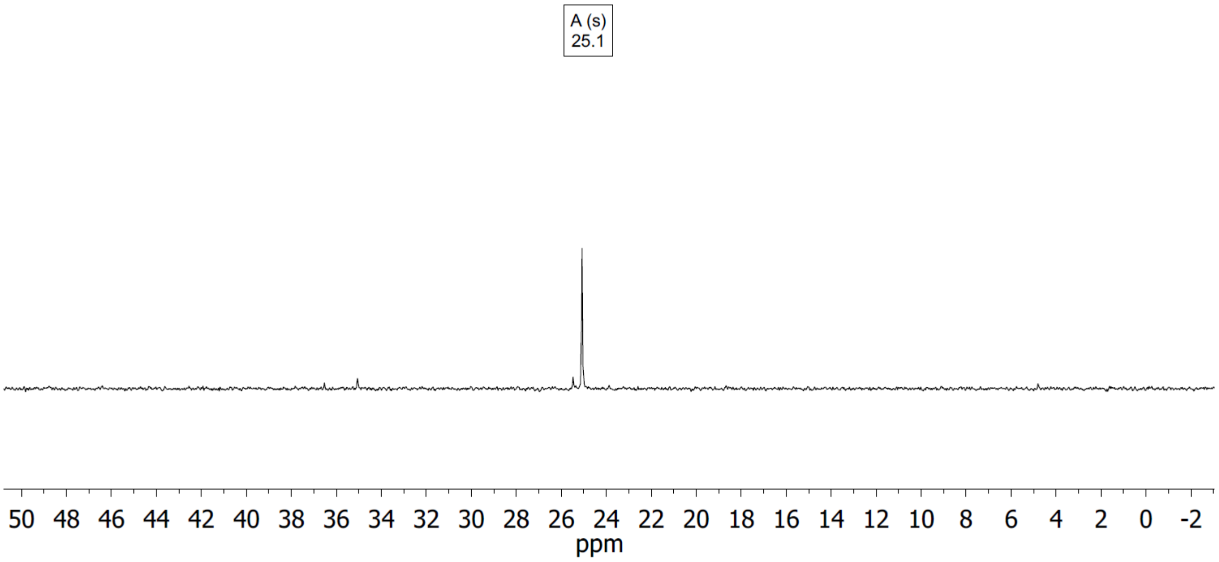

NMR spectra for compound **(E)-10a**

Fig. S45: ^{1}H NMR (CD_2Cl_2, 400 MHz)

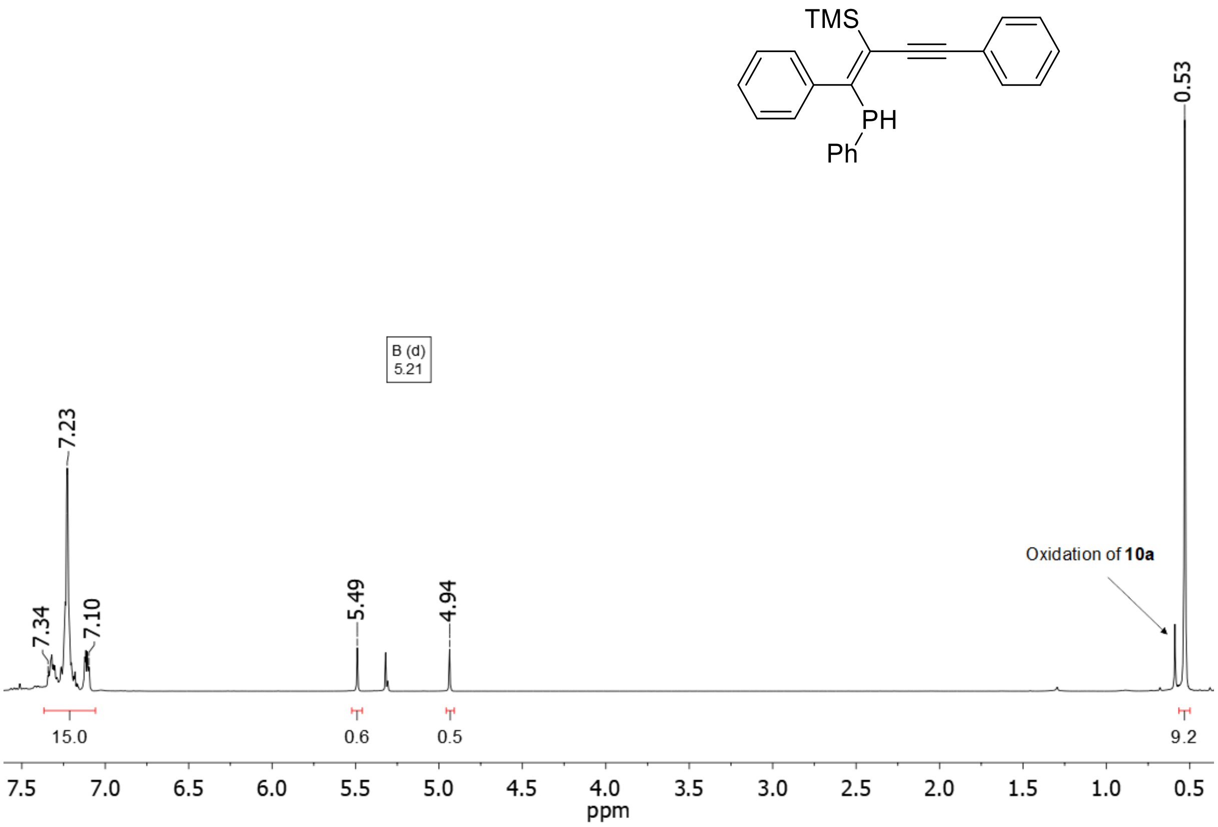

Fig. S46: ^{13}C NMR (CD_2Cl_2, 101 MHz)

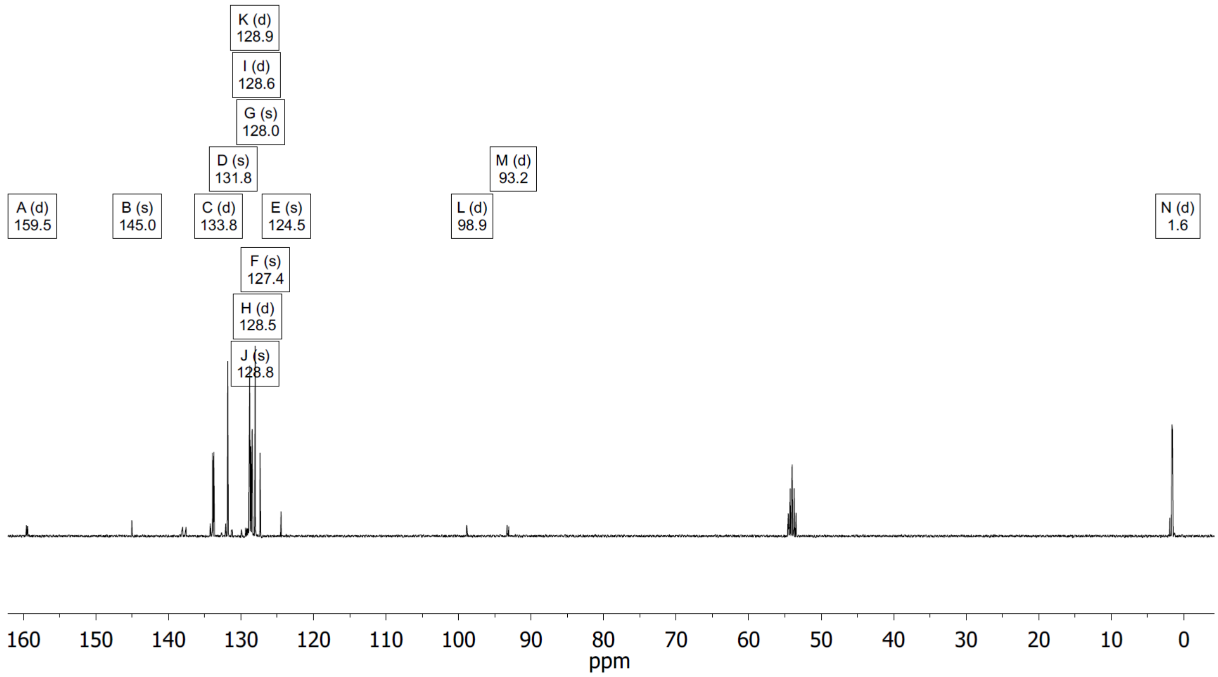

Fig. S47: $^{29}Si\{^1H\}$ NMR (CD_2Cl_2, 99 MHz)

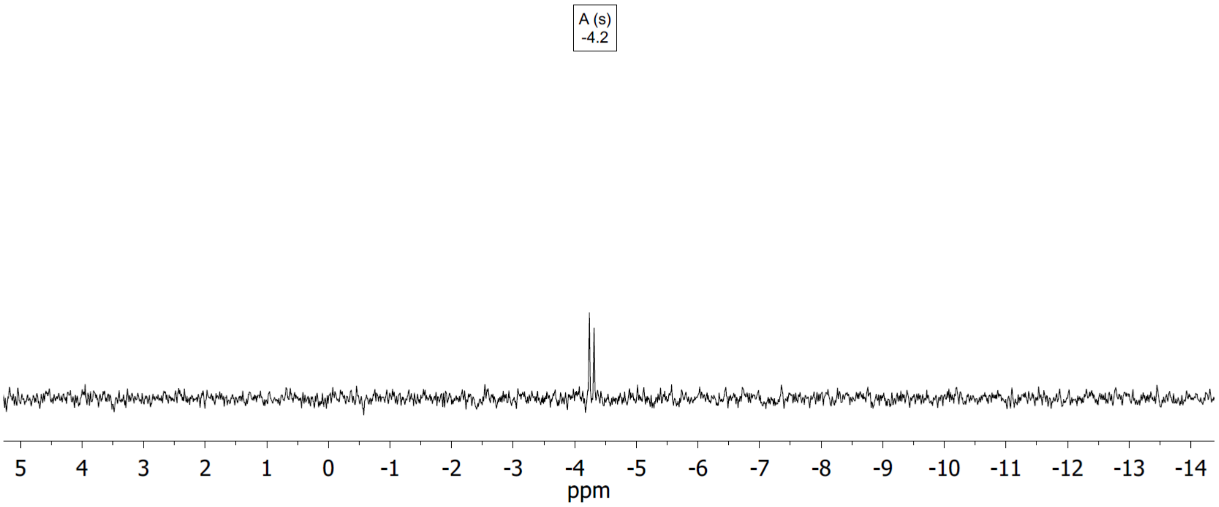

Fig. S48: $^{31}P\{^1H\}$ NMR (CD_2Cl_2, 202 MHz)

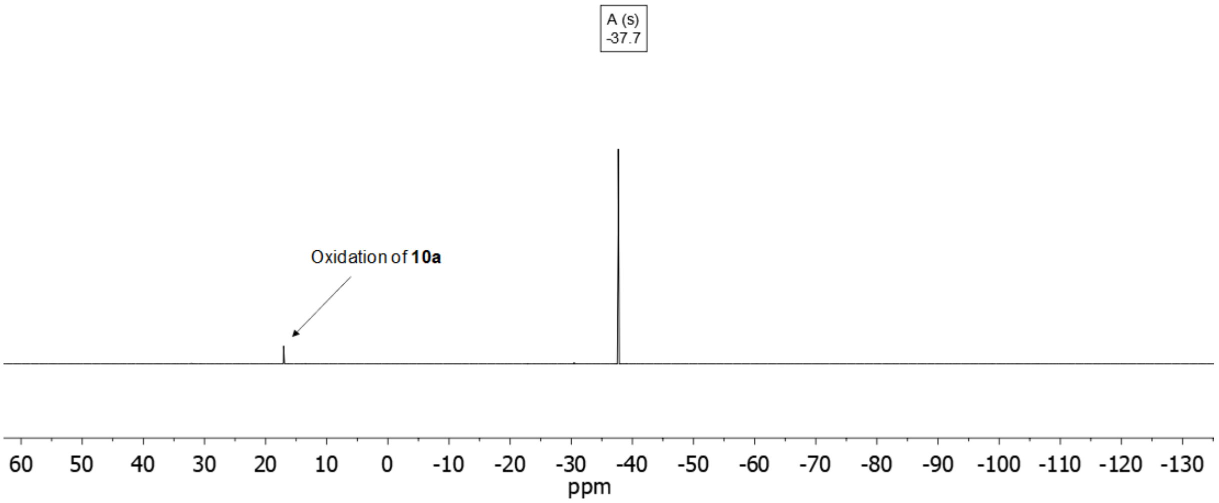

NMR spectra for compound **10b** and **10b’**

Fig. S49: ^{1}H NMR (CD_2Cl_2, 400 MHz)

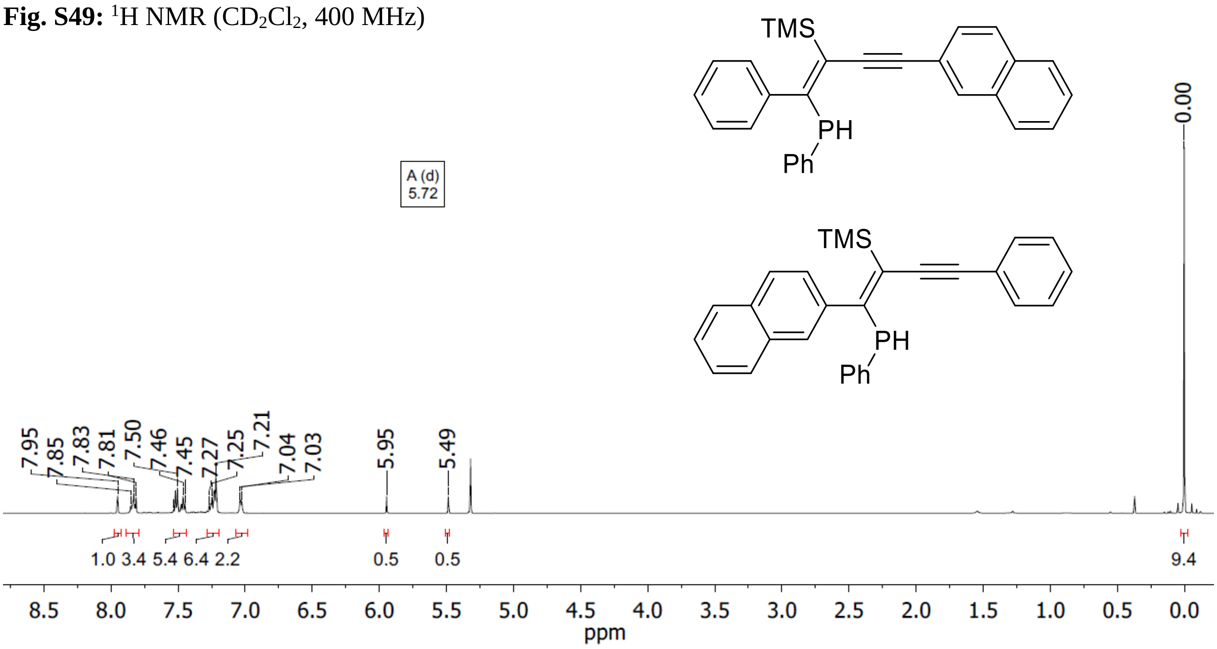

Fig. S50: ^{13}C NMR (CD_2Cl_2, 101 MHz) – 160–0 ppm – Overview spectrum

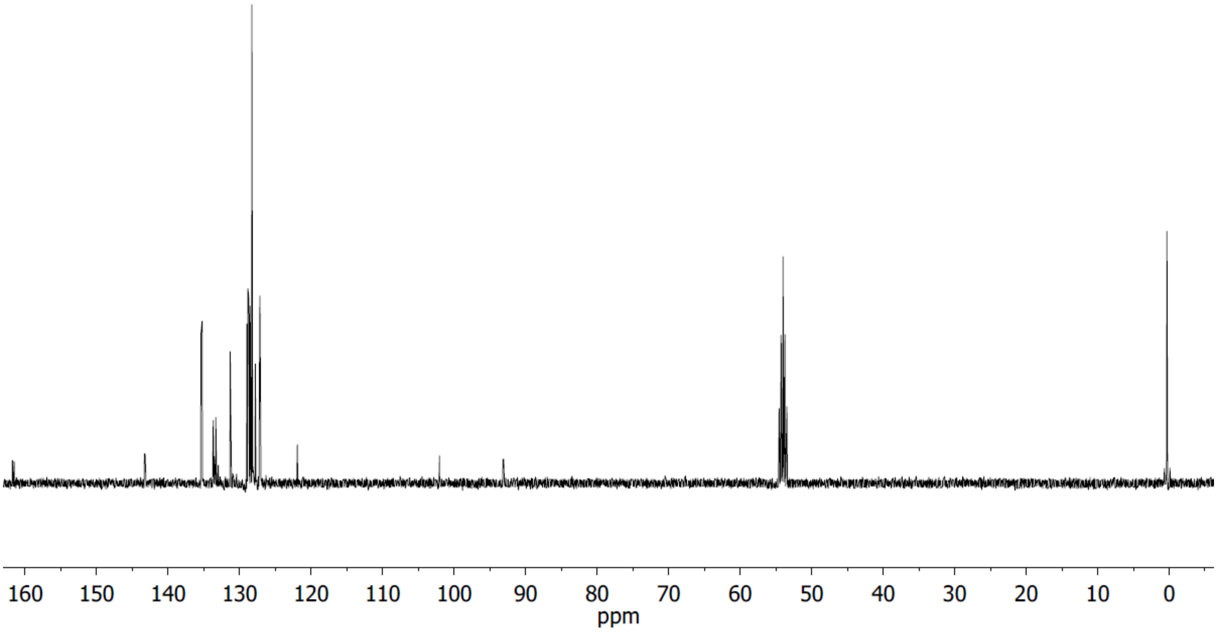

Fig. S51: ^{13}C NMR (CD_2Cl_2, 101 MHz) – 165–0 ppm

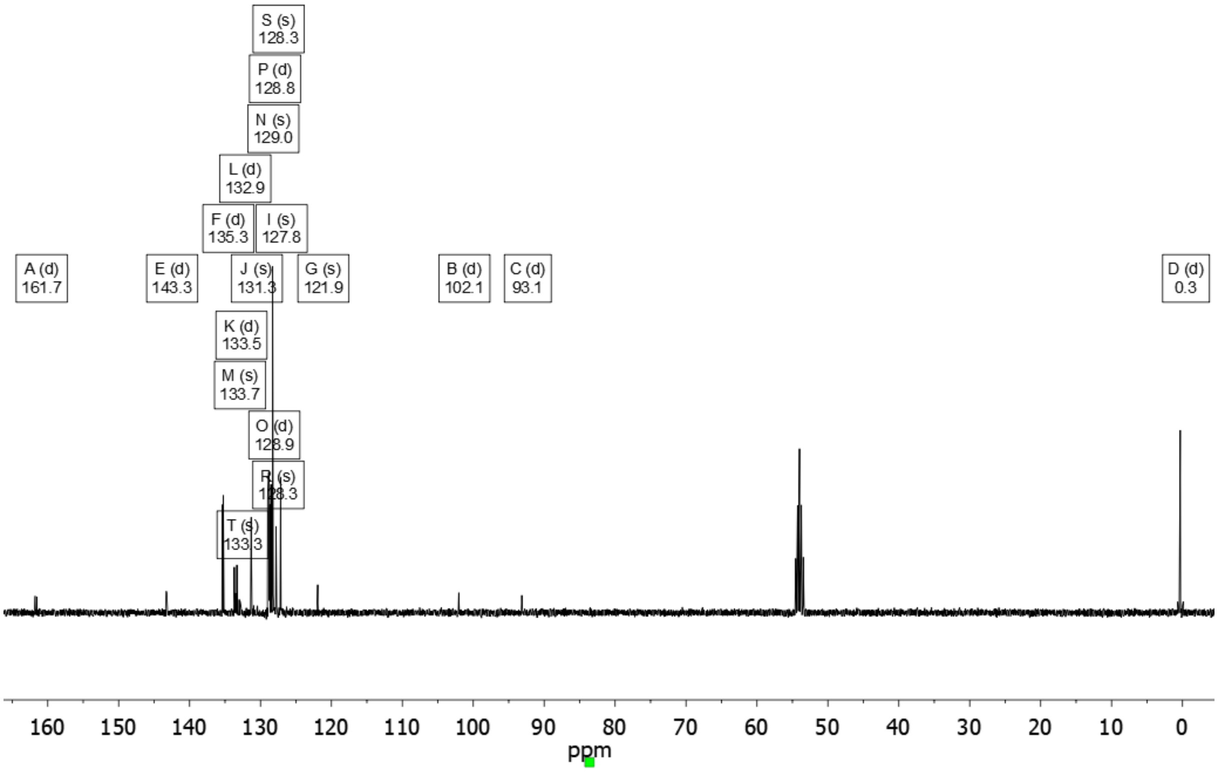

Fig. S52: ^{13}C NMR (CD_2Cl_2, 101 MHz) – 136–126 ppm

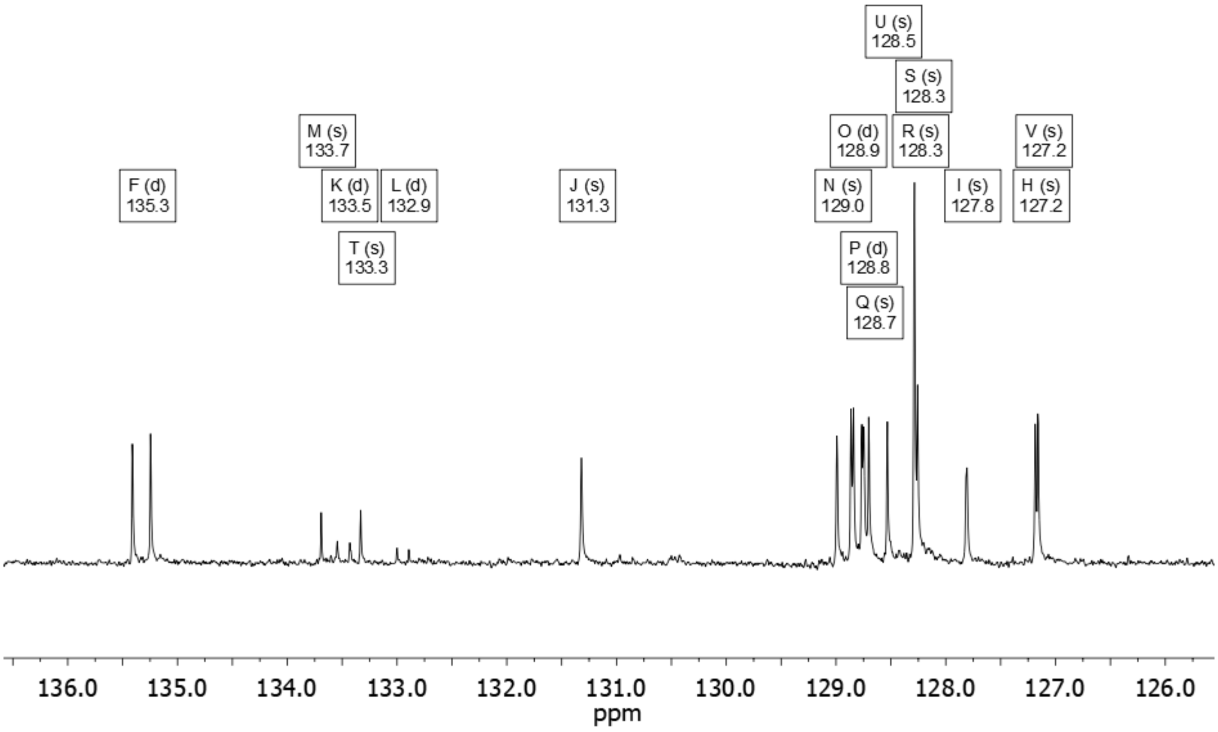

Fig. S53: $^{29}Si\{^1H\}$ NMR (CD_2Cl_2, 99 MHz)

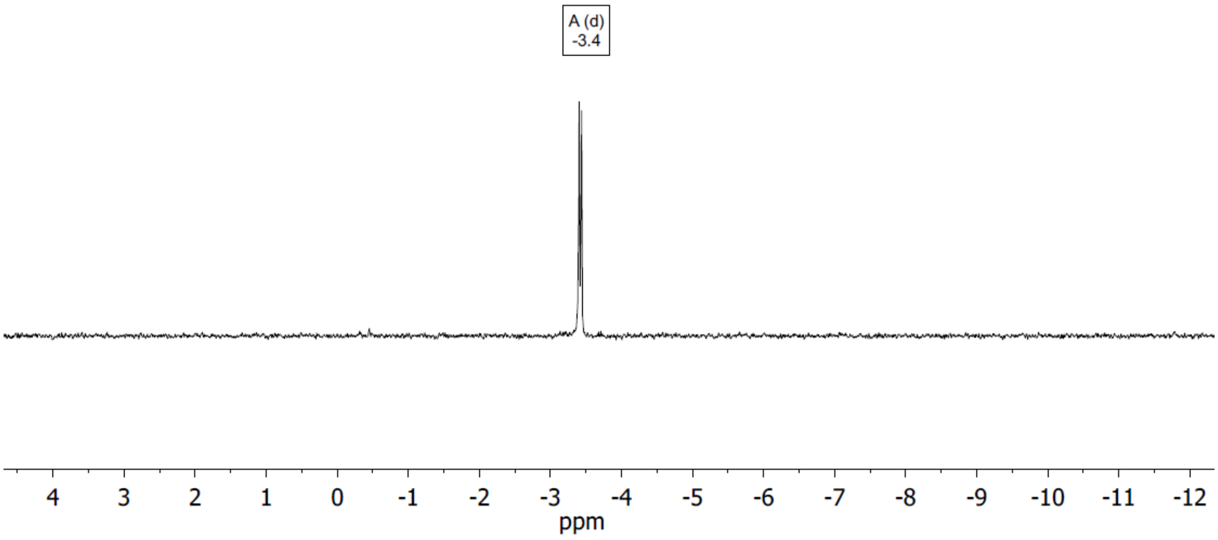

Fig. S54: $^{31}P\{^1H\}$ NMR (CD_2Cl_2, 202 MHz)

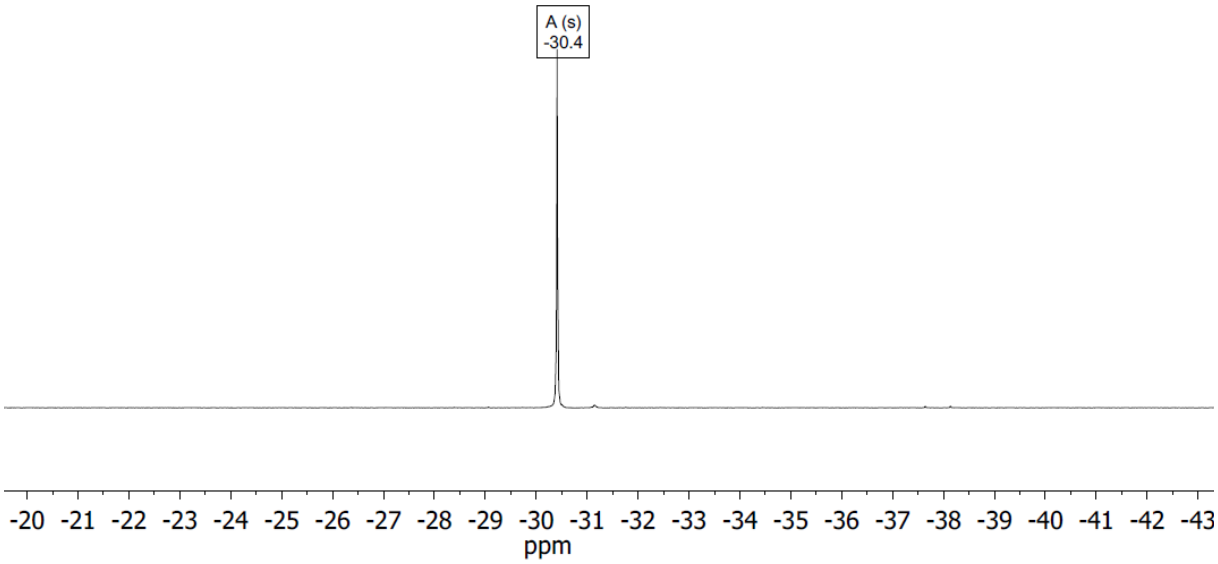

NMR spectra for compound **(*E*)-10c** and **(*Z*)-10c**

Fig. S55: ^1H NMR (CD_2Cl_2, 400 MHz)

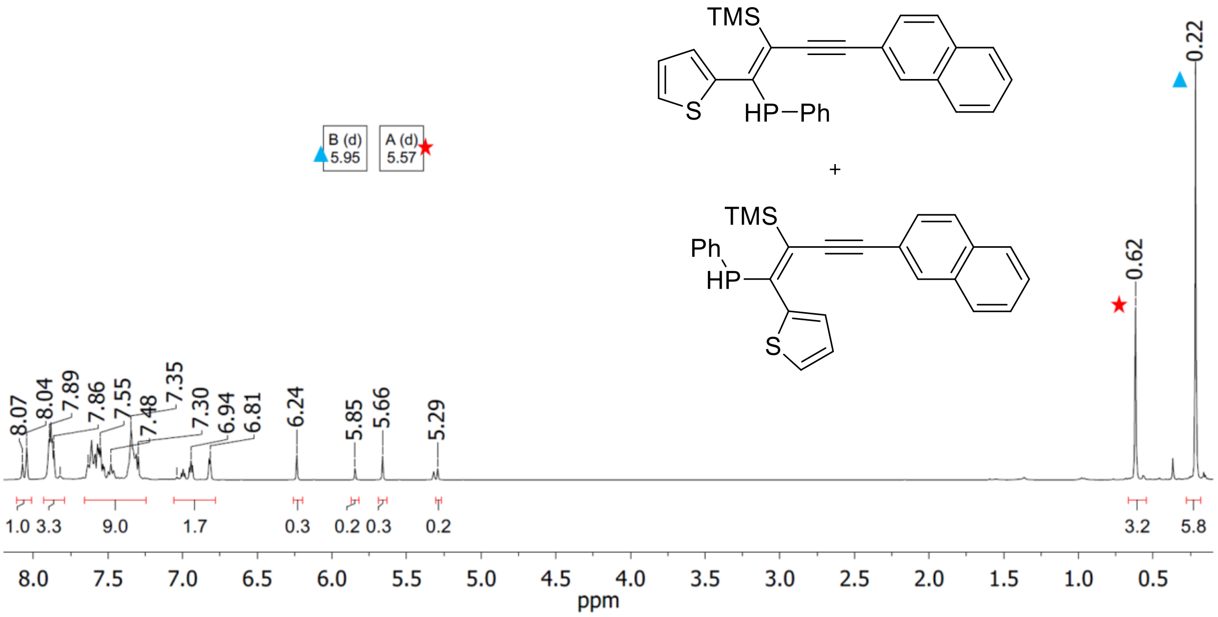

Fig. S56: ^{13}C NMR (CD_2Cl_2, 101 MHz) – Overview spectrum

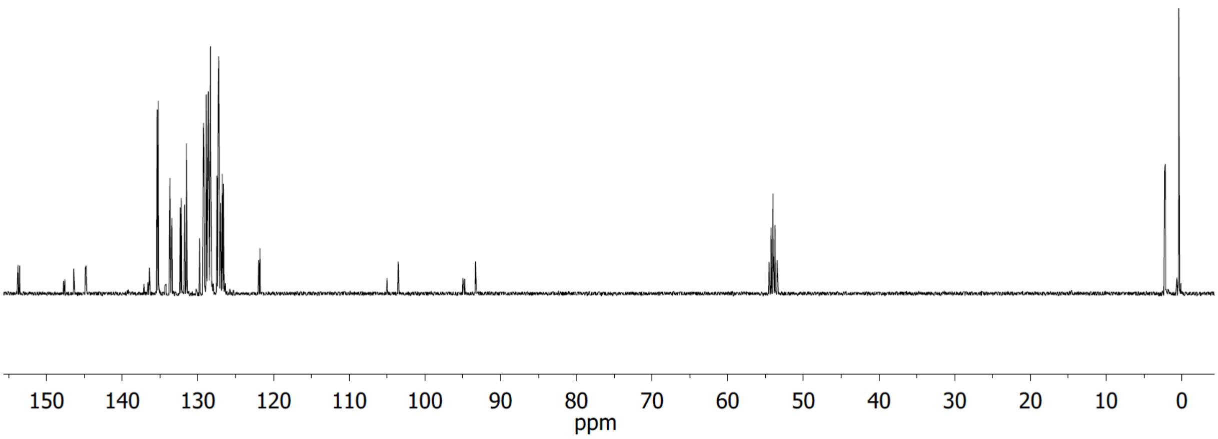

Fig. S57: ^{13}C NMR (CD_2Cl_2, 101 MHz) – 155–135ppm

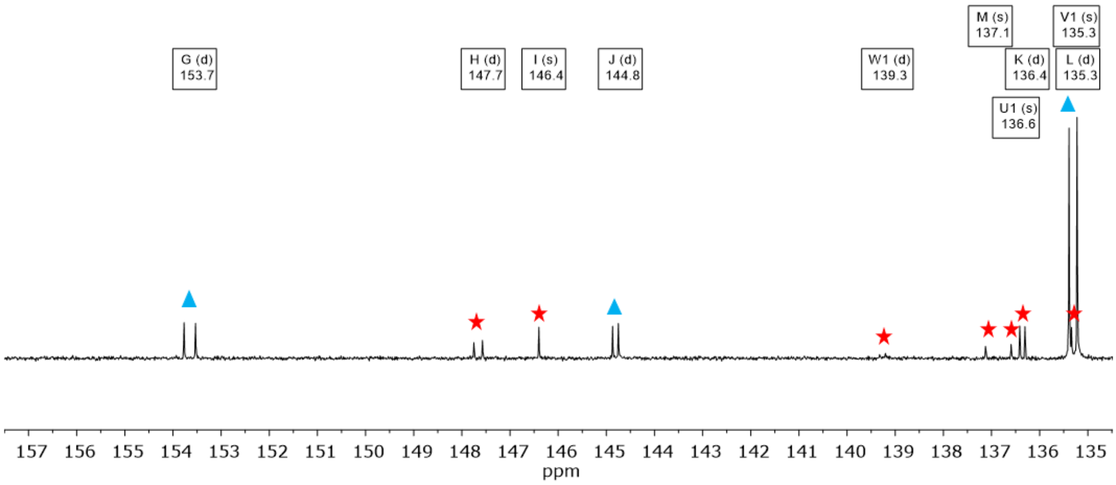

Fig. S58: ^{13}C NMR (CD_2Cl_2, 101 MHz) – 134–126 ppm

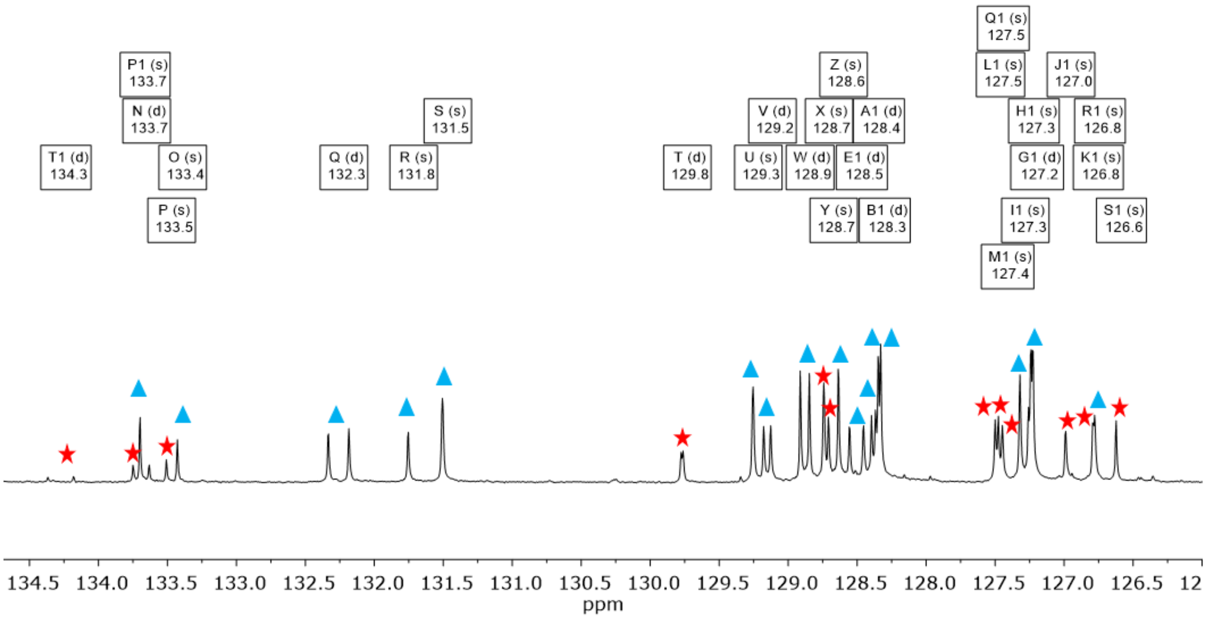

Fig. S59: ^{13}C NMR (CD_2Cl_2, 101 MHz) – 105–0 ppm

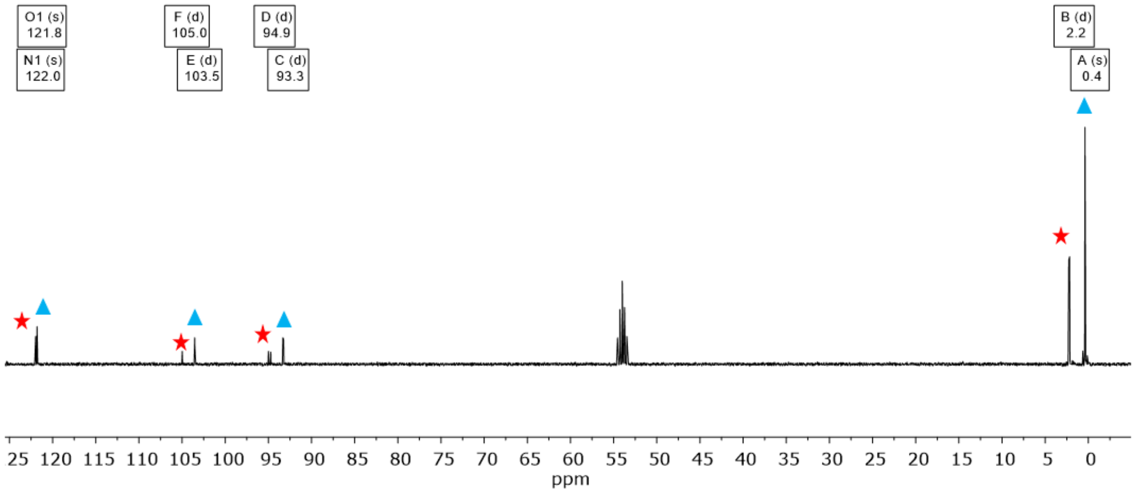

Fig. S60: $^{29}Si\{^{1}H\}$ NMR (CD_2Cl_2, 99 MHz)

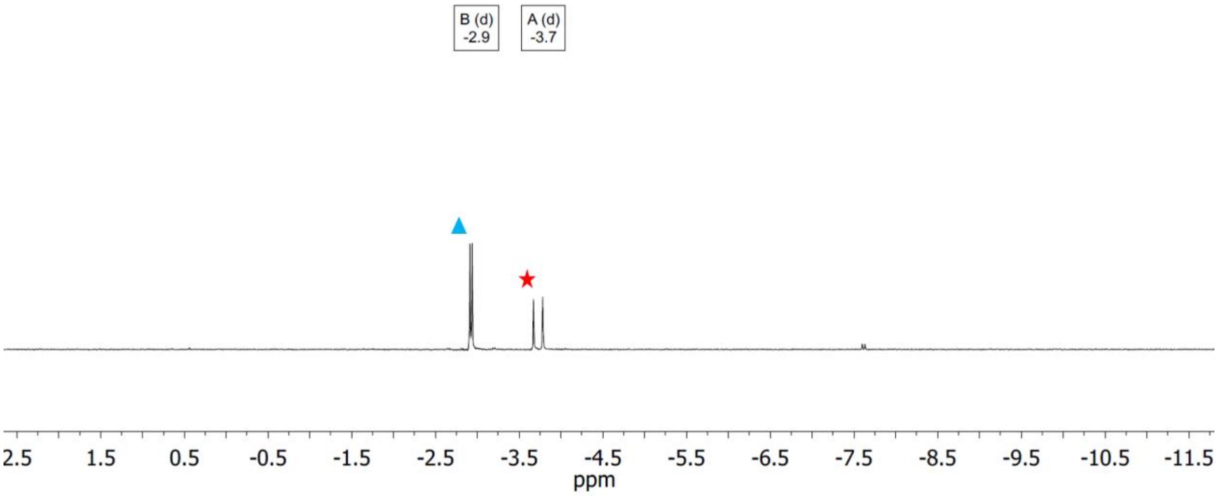

Fig. S61: ${}^{31}P\{{}^{1}H\}$ NMR (CD_2Cl_2, 202 MHz)

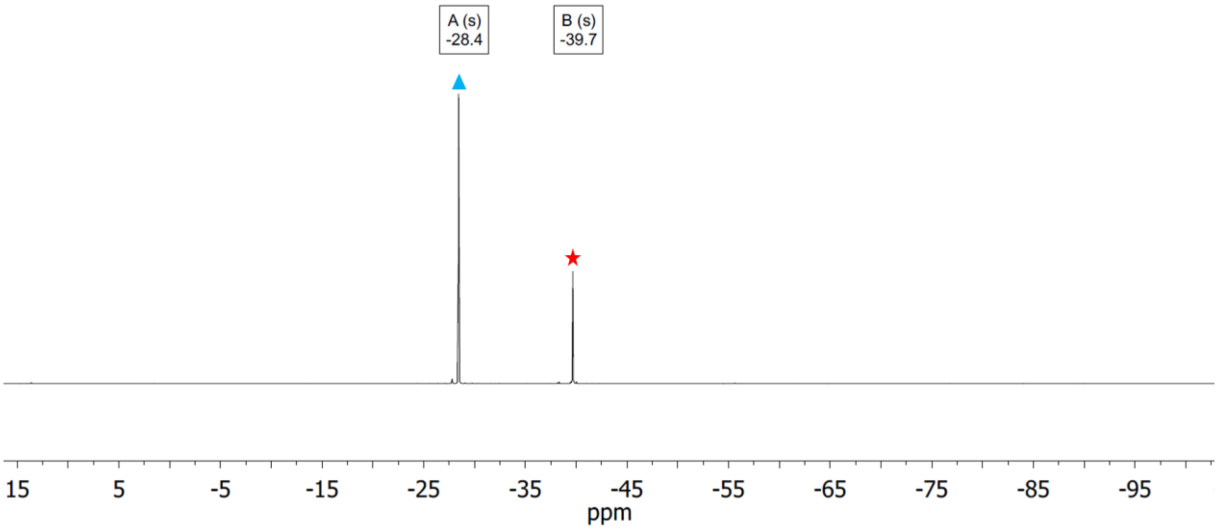

NMR spectra for compound **2d**

Fig. S62: ^{1}H NMR (CD_2Cl_2, 400 MHz)

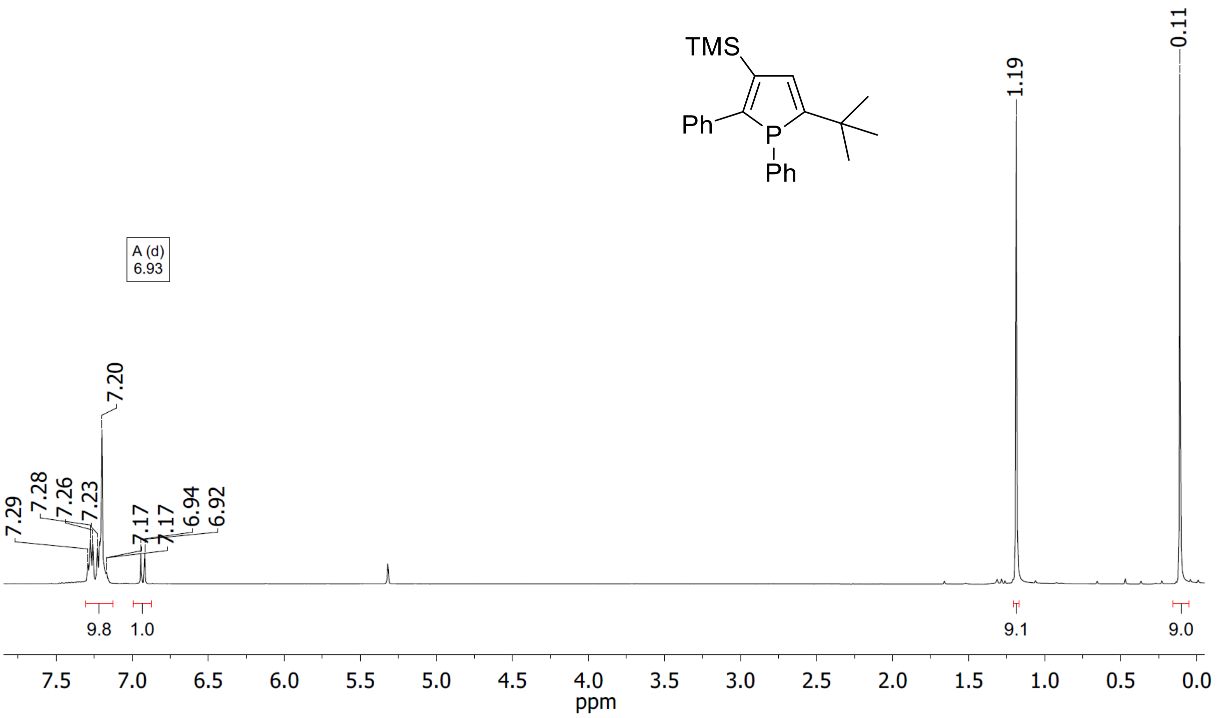

Fig. S63: ^{13}C NMR (CD_2Cl_2, 101 MHz)

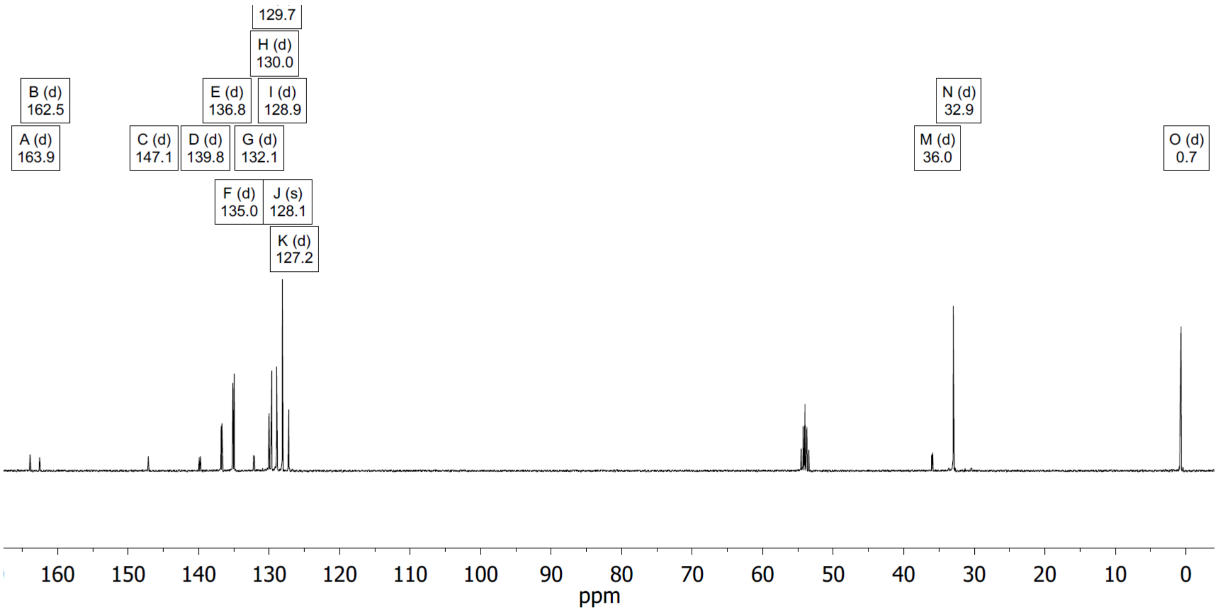

Fig. S64: $^{29}Si\{^{1}H\}$ NMR (CD_2Cl_2, 99 MHz)

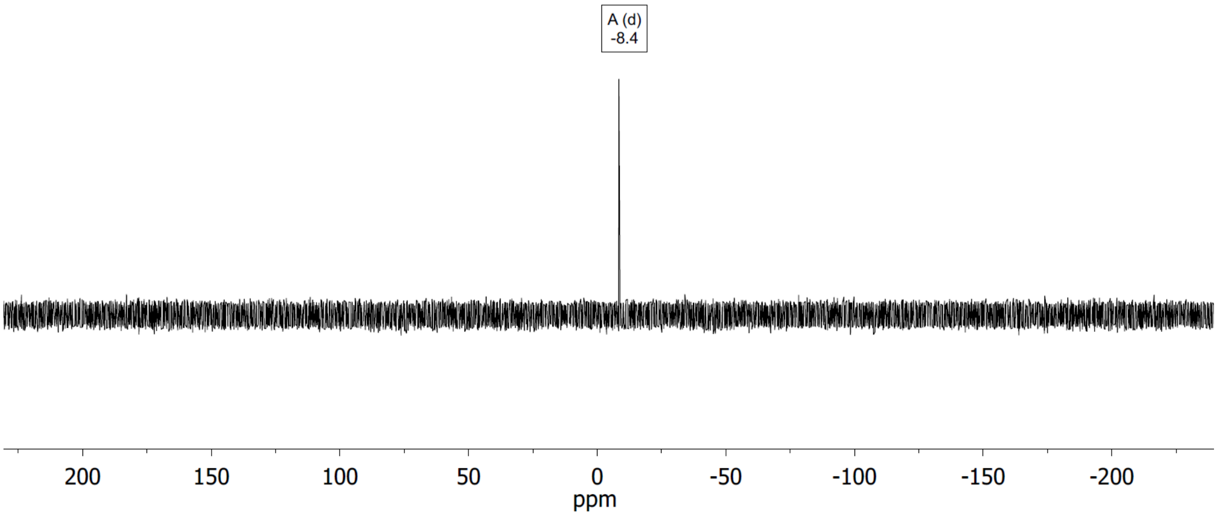

Fig. S65: $^{31}P\{^{1}H\}$ NMR (CD_2Cl_2, 202 MHz)

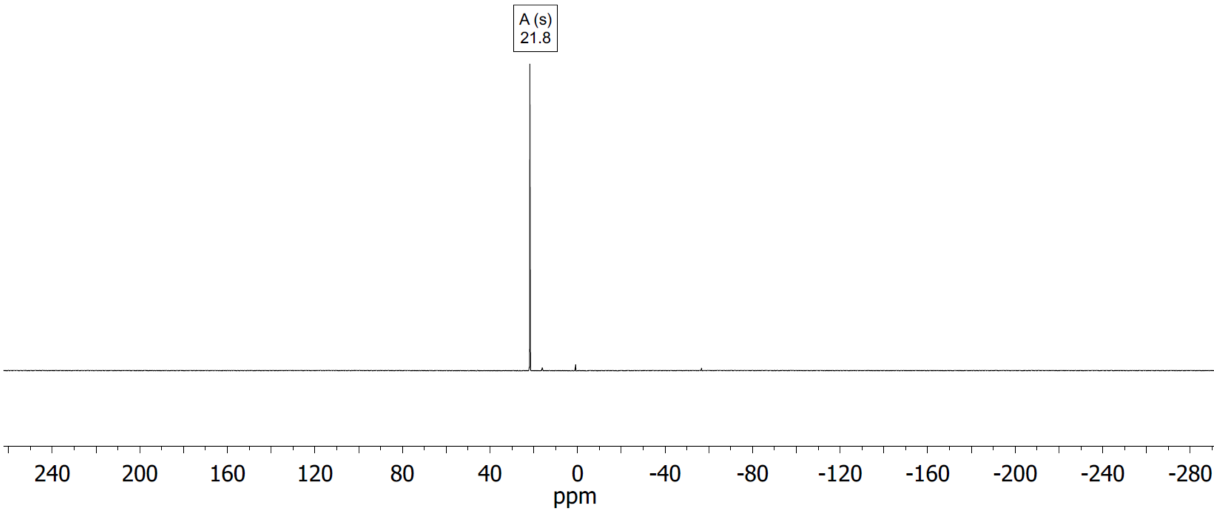

NMR spectra for compound **4d**

Fig. S66: ^{1}H NMR (CD_2Cl_2, 400 MHz)

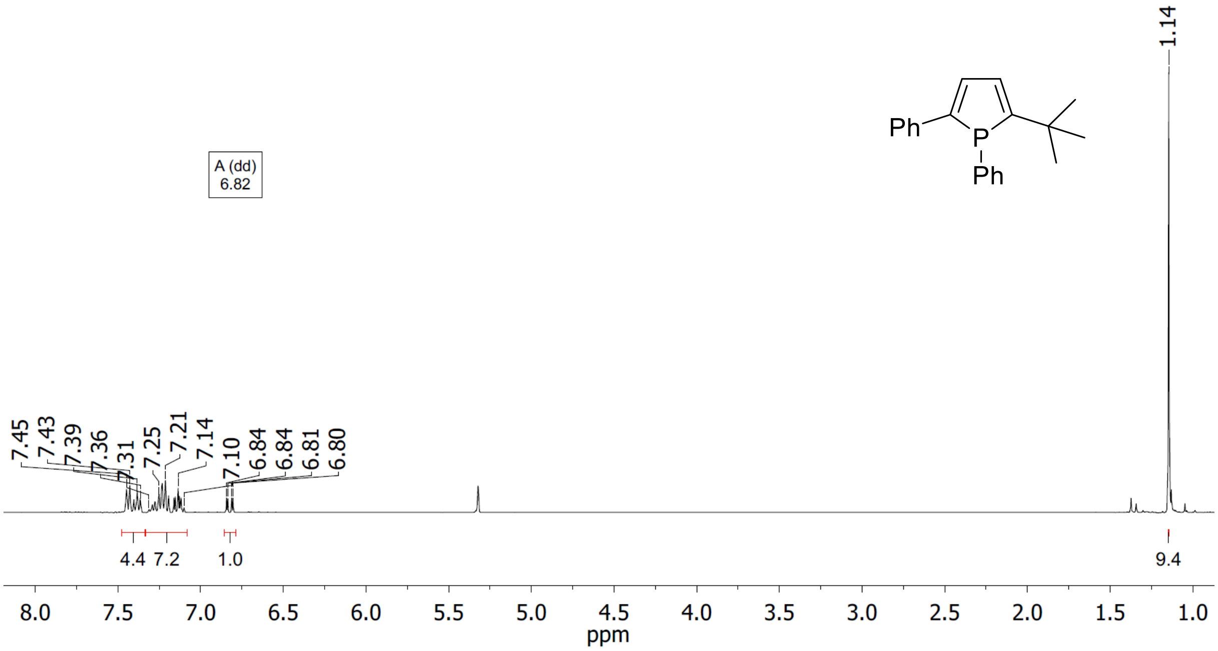

Fig. S67: ^{13}C NMR (CD_2Cl_2, 101 MHz)

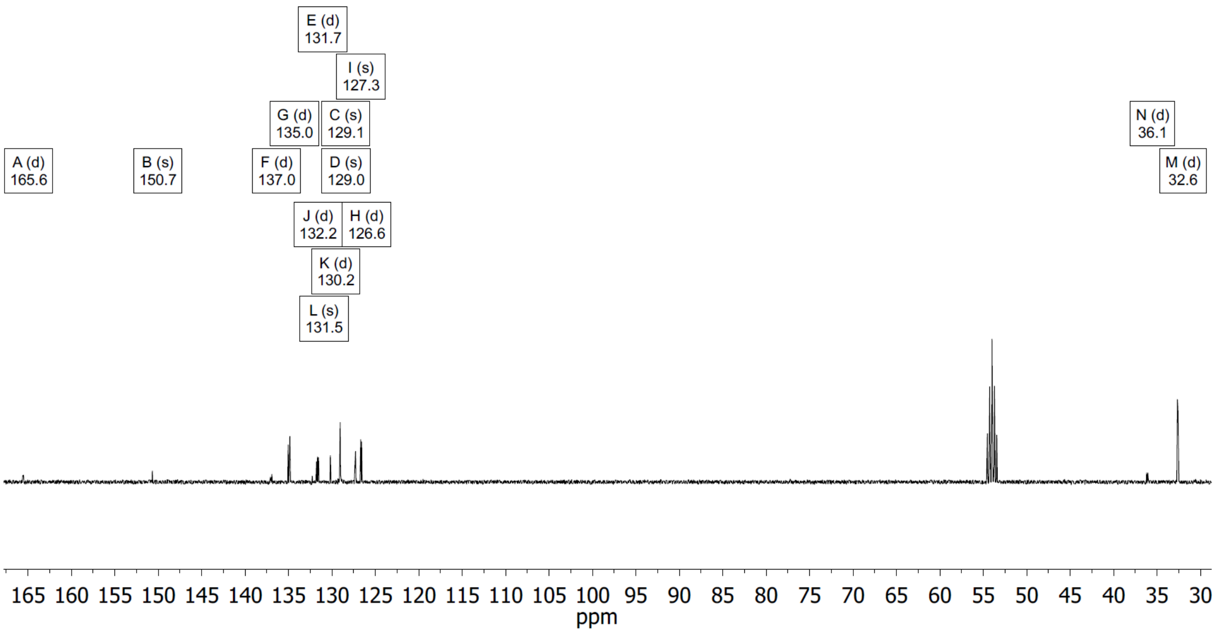

Fig. S68: ^{31}P NMR (CD_2Cl_2, 202 MHz)

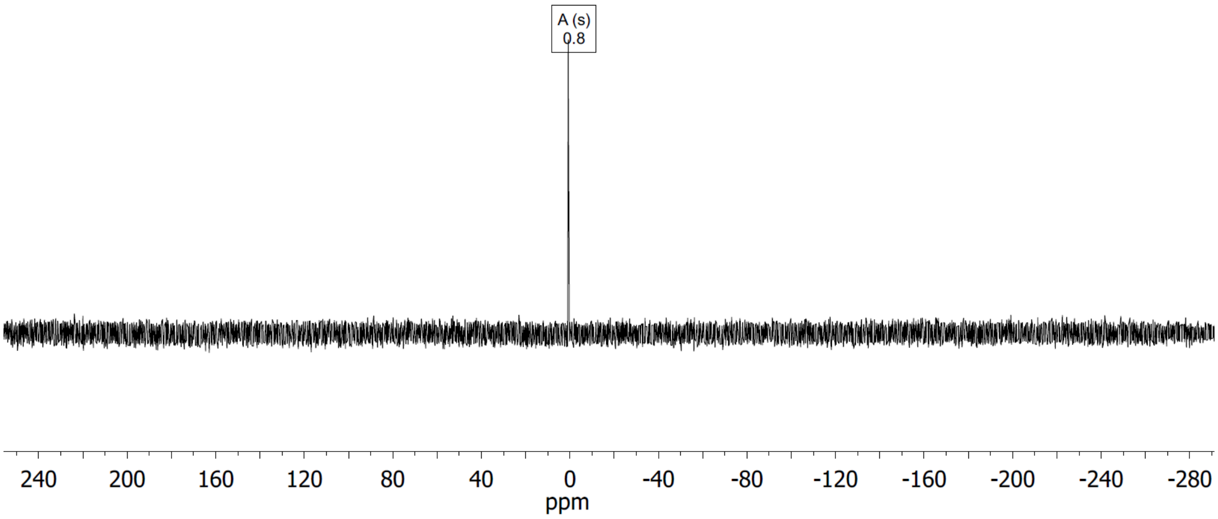

NMR spectra for compound **(*Z*)-10d'**

Fig.S69: ^{1}H NMR (CD_2Cl_2, 400 MHz)

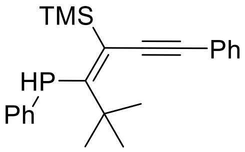

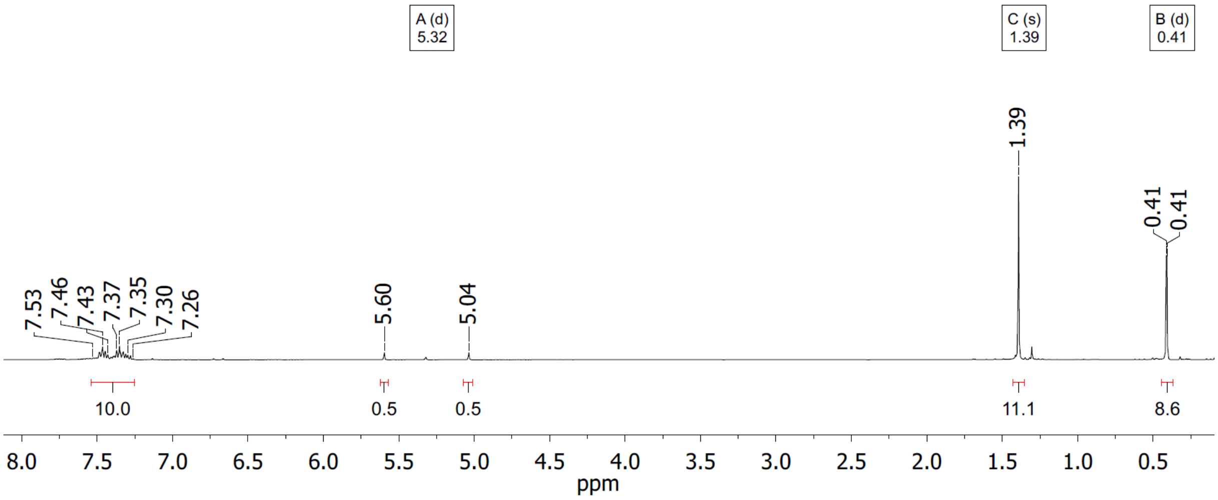

Fig. S70: ^{13}C NMR (CD_2Cl_2, 101 MHz)

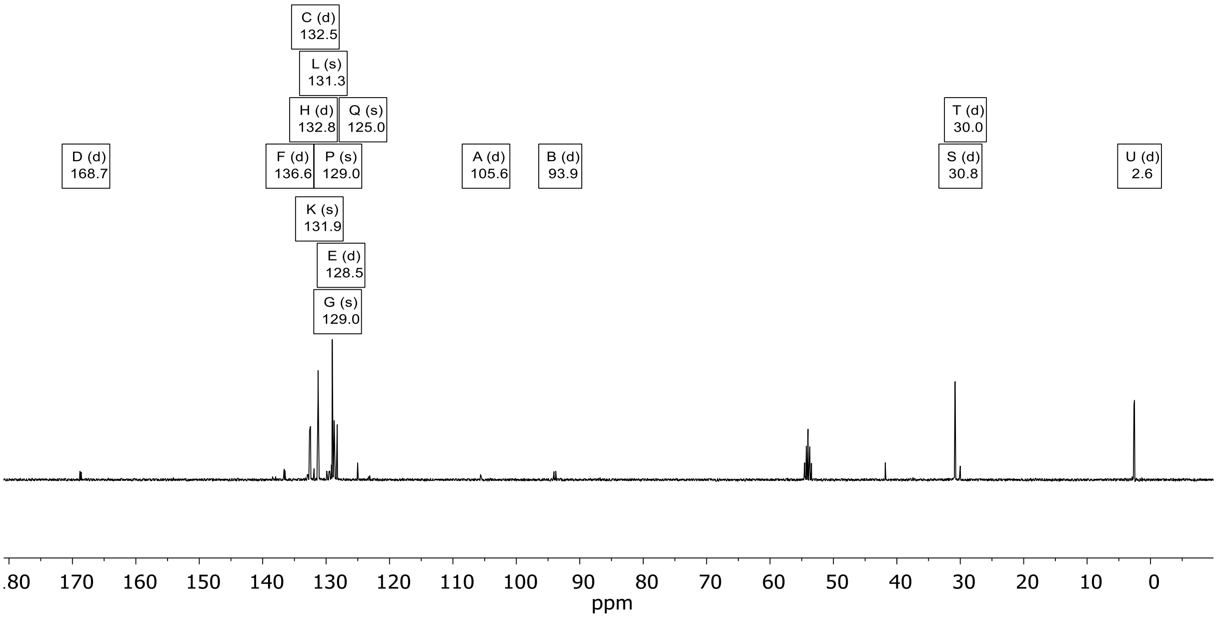

Fig. S71: $^{31}P\{^{1}H\}$ NMR (CD_2Cl_2, 202 MHz)

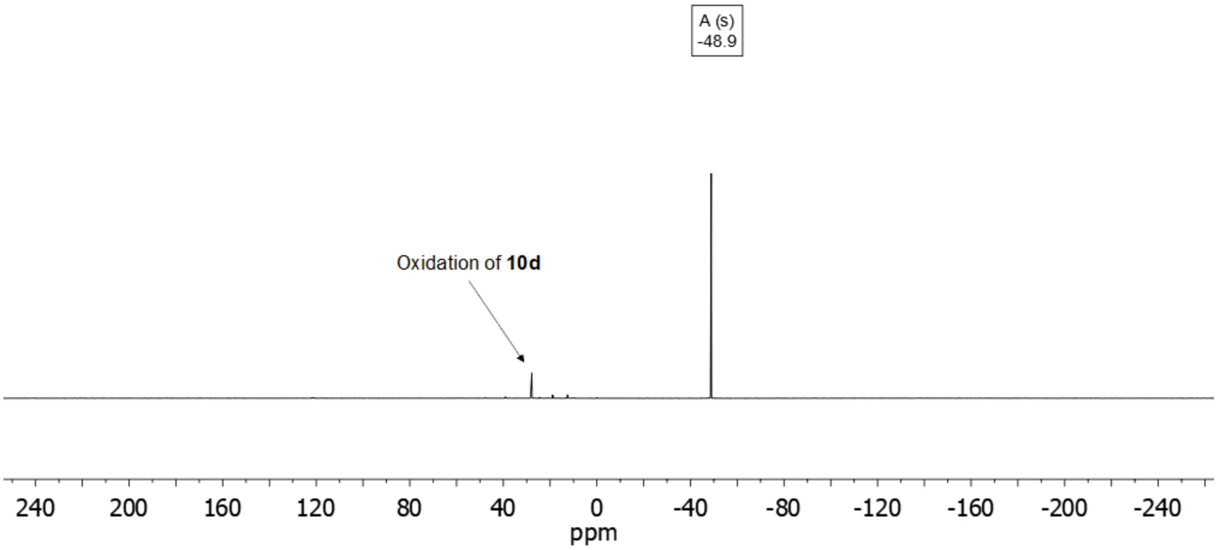

d) Crystallographic data

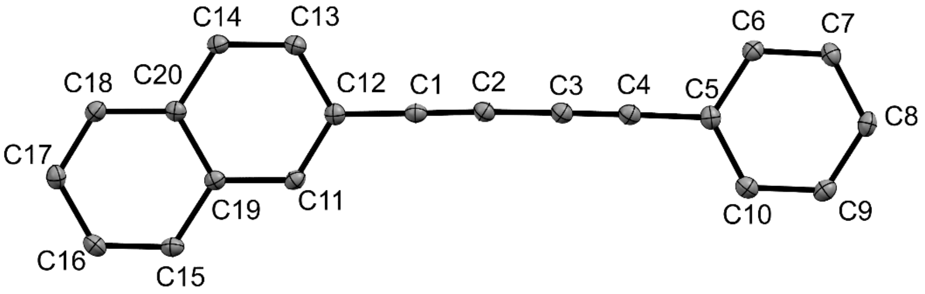

Fig. 72: Molecular structure of **1b** in the solid state. Thermal ellipsoids drawn at 30% probability level. For clarity, hydrogen atoms have been omitted. Important bond lengths and angles: (C12)–(C1) 1.428(3) Å, (C1)–(C2) 1.206(3) Å, (C2)–(C3) 1.371(3) Å, (C3)–(C4) 1.201(3) Å, (C9)–(C10) 1.434(3) Å; (C12)–(C1)–(C2) 179.3(2)°, (C3)–(C4)–(C5) 178.6(2)°.

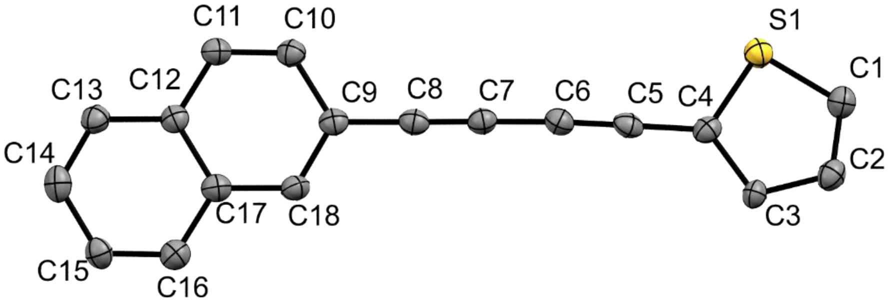

Fig. 73: Molecular structure of **1c** in the solid state. Thermal ellipsoids drawn at 30% probability level. For clarity, hydrogen atoms have been omitted. Important bond lengths and angles: (C8)–(C9) 1.426(10) Å, (C7)–(C8) 1.205(10) Å, (C6)–(C7) 1.363(10) Å, (C5)–(C6) 1.223(11) Å, (C4)–(C5) 1.400(11) Å; (C7)–(C8)–(C9) 178.2(8)°, (C4)–(C5)–(C6) 179.6(8)°.

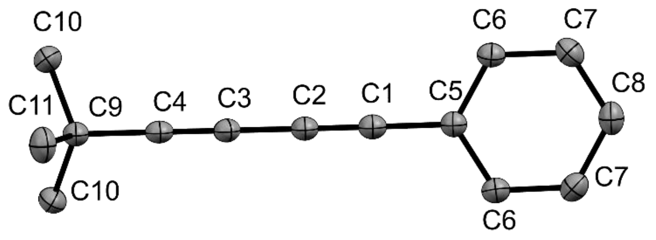

Fig. 74: Molecular structure of **1d** in the solid state. Thermal ellipsoids drawn at 30% probability level. For clarity, hydrogen atoms have been omitted. Important bond lengths and angles: (C4)–(C9) 1.478(2) Å, (C3)–(C4) 1.196(2) Å, (C2)–(C3) 1.378(2) Å, (C1)–(C2) 1.197(3) Å, (C1)–(C5) 1.432(2) Å; (C3)–(C4)–(C9) 178.8(2)°, (C8)–(C9)–(C10) 177.4(2)°.

Table S8: Structure determination and refinement of **3a–3c**.

Compound reference	**3a**	**3b'**	**3c**
Identification code	sv0757	sv0995	sv0738
Empirical formula	$C_{25}H_{25}PSi$	$C_{29}H_{27}PSi$	$C_{27}H_{25}PSSi$
Formula weight	384.51	434.56	440.59
Crystal system	monoclinic	monoclinic	monoclinic
Space group	C2/c	C2/c	C2/c
a/Å	37.703(2)	46.604(2)	41.177(2)
b/Å	5.9845(2)	6.0209(2)	6.1584(2)
c/Å	19.6736(11)	17.4856(9)	18.9885(9)
α/°	90	90	90
β/°	103.962(4)	103.130(4)	108.118(4)
γ/°	90	90	90
Volume/$Å^3$	4307.9(4)	4778.2(4)	4576.4(4)
Z	8	8	8
$\rho_{calc}g/cm^3$	1.186	1.208	1.279
μ/mm^{-1}	1.693	1.586	2.493
F(000)	1632.0	1840.0	1856.0
Crystal size/mm^3	0.22 × 0.09 × 0.02	0.59 × 0.223 × 0.04	0.14 × 0.063 × 0.01
Radiation	CuKα (λ = 1.54186)	CuKα (λ = 1.54186)	CuKα (λ = 1.54186)
2Θ range for data collection/°	4.83 to 138.984	10.39 to 141.084	4.516 to 138.946
Index ranges	-45 ≤ h ≤ 32, -3 ≤ k ≤ 7, -22 ≤ l ≤ 23	-55 ≤ h ≤ 51, -3 ≤ k ≤ 7, -20 ≤ l ≤ 19	-49 ≤ h ≤ 40, -3 ≤ k ≤ 7, -22 ≤ l ≤ 22
Reflections collected	8352	9821	8927
Independent reflections	3947 [R_{int} = 0.0826, R_{sigma} = 0.0660]	4340 [R_{int} = 0.0200, R_{sigma} = 0.0215]	4213 [R_{int} = 0.0928, R_{sigma} = 0.0908]
Data/restraints/parameters	3947/0/247	4340/102/352	4213/27/311
Goodness-of-fit on F^2	1.119	1.045	1.079
Final R indexes [I>=2σ (I)]	R_1 = 0.0969, wR_2 = 0.2520	R_1 = 0.0552, wR_2 = 0.1285	R_1 = 0.1041, wR_2 = 0.2687
Final R indexes [all data]	R_1 = 0.1192, wR_2 = 0.2842	R_1 = 0.0688, wR_2 = 0.1382	R_1 = 0.1416, wR_2 = 0.3123
Largest diff. peak/hole / e $Å^{-3}$	1.15/-0.54	0.86/-0.40	0.71/-0.49
CCDC number	2067623	2067629	2067628

Table S9: Structure determination and refinement of **51**, **5b** and **11**.

Compound reference	**5a**	**5b**	**11**
Identification code	sv0944	sv1014	i3156
Empirical formula	$C_{22}H_{17}P$	$C_{26}H_{19}P$	$C_{46}H_{56}P_2Si_2$
Formula weight	312.32	362.38	727.02
Crystal system	monoclinic	monoclinic	triclinic
Space group	$P2_1$	$P2_1$	P-1
a/Å	11.9909(10)	12.6335(5)	9.2860(9)
b/Å	5.8023(3)	5.8648(2)	10.8844(10)
c/Å	12.9427(11)	12.8666(6)	11.4139(12)
α/°	90	90	96.940(8)
β/°	114.451(6)	103.084(3)	109.564(7)
γ/°	90	90	102.036(7)
Volume/$Å^3$	819.73(11)	928.58(7)	1040.13(18)
Z	2	2	1
ρ_{calc}g/cm^3	1.265	1.296	1.161
μ/mm^{-1}	1.432	1.342	0.193
F(000)	328.0	380.0	390.0
Crystal size/mm^3	0.33 × 0.127 × 0.02	0.17 × 0.083 × 0.03	0.15 × 0.097 × 0.05
Radiation	CuKα (λ = 1.54186)	CuKα (λ = 1.54186)	MoKα (λ = 0.71073)
2Θ range for data collection/°	7.504 to 142.22	7.054 to 141.98	3.87 to 51.476
Index ranges	-14 ≤ h ≤ 14, -3 ≤ k ≤ 6, -15 ≤ l ≤ 15	-15 ≤ h ≤ 14, -5 ≤ k ≤ 7, -15 ≤ l ≤ 15	-11 ≤ h ≤ 10, -13 ≤ k ≤ 13, -13 ≤ l ≤ 13
Reflections collected	5841	9196	8112
Independent reflections	1971 [R_{int} = 0.0365, R_{sigma} = 0.0299]	2746 [R_{int} = 0.0160, R_{sigma} = 0.0154]	3916 [R_{int} = 0.0691, R_{sigma} = 0.0705]
Data/restraints/parameters	1971/607/271	2746/28/245	3916/0/232
Goodness-of-fit on F^2	1.079	1.074	1.051
Final R indexes [I>=2σ (I)]	R_1 = 0.0583, wR_2 = 0.1462	R_1 = 0.0405, wR_2 = 0.0909	R_1 = 0.0597, wR_2 = 0.1593
Final R indexes [all data]	R_1 = 0.0658, wR_2 = 0.1527	R_1 = 0.0444, wR_2 = 0.0938	R_1 = 0.0872, wR_2 = 0.1740
Largest diff. peak/hole / e Å^{-3}	0.52/-0.25	0.82/-0.45	1.40/-0.41
Flack parameter	-0.06(6)	0.31(4)	
CCDC number	2067625	2067624	2067633

Table S10: Structure determination and refinement of **4b–4d**.

Compound reference	**4b**	**4c**	**4d**
Identification code	sv1007	sv1042	sv0946
Empirical formula	$C_{26}H_{19}P$	$C_{24}H_{17}PS$	$C_{20}H_{21}P$
Formula weight	362.38	368.40	292.34
Crystal system	monoclinic	monoclinic	monoclinic
Space group	$P2_1$	$P2_1/c$	$P2_1/n$
a/Å	12.4782(15)	9.5303(9)	13.7639(15)
b/Å	5.8870(5)	5.9286(4)	6.0089(5)
c/Å	12.8360(15)	32.014(3)	19.119(2)
α/°	90	90	90
β/°	104.614(9)	96.552(8)	91.875(9)
γ/°	90	90	90
Volume/$Å^3$	912.42(18)	1797.0(3)	1580.4(3)
Z	2	4	4
$\rho_{calc}g/cm^3$	1.319	1.362	1.229
μ/mm^{-1}	1.366	2.453	1.441
F(000)	380.0	768.0	624.0
Crystal size/mm^3	0.31 × 0.127 × 0.02	0.22 × 0.087 × 0.01	0.24 × 0.103 × 0.01
Radiation	CuKα (λ = 1.54186)	CuKα (λ = 1.54186)	CuKα (λ = 1.54186)
2Θ range for data collection/°	7.118 to 141.938	9.34 to 136.968	8.042 to 137.854
Index ranges	-12 ≤ h ≤ 15, -7 ≤ k ≤ 5, -15 ≤ l ≤ 14	-10 ≤ h ≤ 11, -7 ≤ k ≤ 3, -38 ≤ l ≤ 37	-15 ≤ h ≤ 16, -3 ≤ k ≤ 7, -22 ≤ l ≤ 22
Reflections collected	7026	6829	5680
Independent reflections	2788 [R_{int} = 0.0239, R_{sigma} = 0.0219]	3183 [R_{int} = 0.0743, R_{sigma} = 0.0778]	2852 [R_{int} = 0.0710, R_{sigma} = 0.0658]
Data/restraints/parameters	2788/1/244	3183/0/235	2852/67/229
Goodness-of-fit on F^2	1.021	1.091	1.105
Final R indexes [I>=2σ (I)]	R_1 = 0.0368, wR_2 = 0.0941	R_1 = 0.1089, wR_2 = 0.3047	R_1 = 0.0878, wR_2 = 0.2290
Final R indexes [all data]	R_1 = 0.0399, wR_2 = 0.0970	R_1 = 0.1386, wR_2 = 0.3300	R_1 = 0.1095, wR_2 = 0.2523
Largest diff. peak/hole / e $Å^{-3}$	0.35/-0.20	0.79/-0.53	0.81/-0.79
Flack parameter	-0.04(3)		
CCDC number	2067630	2067631	2067626

Table S11: Structure determination and refinement of **1b–1d**.

Compound reference	**1b**	**1c**	**1d**
Identification code	i2798	sv0917	i2955
Empirical formula	$C_{20}H_{12}$	$C_{18}H_{10}S$	$C_{14}H_{14}$
Formula weight	252.30	258.32	182.25
Crystal system	orthorhombic	orthorhombic	orthorhombic
Space group	$P2_12_12_1$	$P2_12_12_1$	Pnma
a/Å	6.1805(3)	3.8645(2)	17.3308(15)
b/Å	13.7518(9)	6.1293(5)	8.4288(7)
c/Å	15.9281(7)	52.956(3)	7.8582(6)
α/°	90	90	90
β/°	90	90	90
γ/°	90	90	90
Volume/Å^3	1353.78(13)	1254.35(15)	1147.91(16)
Z	4	4	4
ρ_{calc}g/cm^3	1.238	1.368	1.055
μ/mm^{-1}	0.070	2.103	0.059
F(000)	528.0	536.0	392.0
Crystal size/mm^3	0.37 × 0.163 × 0.03	0.16 × 0.08 × 0.03	0.29 × 0.197 × 0.15
Radiation	MoKα (λ = 0.71073)	CuKα (λ = 1.54186)	MoKα (λ = 0.71073)
2Θ range for data collection/°	3.912 to 51.2	6.676 to 137.924	4.7 to 53.59
Index ranges	-7 ≤ h ≤ 6, -16 ≤ k ≤ 16, -19 ≤ l ≤ 19	-4 ≤ h ≤ 1, -7 ≤ k ≤ 6, -63 ≤ l ≤ 61	-21 ≤ h ≤ 21, -10 ≤ k ≤ 10, -9 ≤ l ≤ 9
Reflections collected	7098	4054	7541
Independent reflections	2565 [R_{int} = 0.0232, R_{sigma} = 0.0199]	2164 [R_{int} = 0.0521, R_{sigma} = 0.0407]	1311 [R_{int} = 0.0617, R_{sigma} = 0.0255]
Data/restraints/parameters	2565/0/181	2164/0/172	1311/0/82
Goodness-of-fit on F^2	1.023	1.498	1.064
Final R indexes [I>=2σ (I)]	R_1 = 0.0292, wR_2 = 0.0710	R_1 = 0.1070, wR_2 = 0.2972	R_1 = 0.0648, wR_2 = 0.1875
Final R indexes [all data]	R_1 = 0.0328, wR_2 = 0.0729	R_1 = 0.1149, wR_2 = 0.3194	R_1 = 0.0732, wR_2 = 0.1986
Largest diff. peak/hole / e Å^{-3}	0.11/-0.12	0.86/-0.64	0.44/-0.21
Flack parameter	10.0(10)	-0.01(8)	
CCDC number	2075536	2067627	2067632

e) Computational details

DFT calculations were carried out with the Gaussian 09 program package[7], using the ωB97X-D hybrid functional[8]. in conjunction with the 6-311+G**. To test this level of theory energy difference between (*E*)-7a and (*Z*)-7a was calculated at different level of theory (Table S12). LCCSD(T)/aug-cc-pVTZ calculations were performed with MRCC program package (www.mrcc.hu) using the optimized (ωB97X-D/6-311+G**) geometries.[9] Geometries were fully optimized for all molecules, and harmonic vibrational frequencies were calculated at the same level of theory to establish the nature of the stationary points obtained. Transition structures were characterized by a single negative eigenvalue of the Hessian, and IRC calculations were performed to locate the corresponding minima. TD-DFT calculations were calculated at B3LYP/6-31G*//ωB97X-D level of theory.

Table S12 Energy diffference between **(E)-7a** and **(Z)-7a** at different level of theory

Level ot theory	ΔE) [kcal/mol]
ωB97X-D/6-311+G**	-2.3
ωB97X-D /6-311+G** (PCM=THF)	-1.1
B3LYP/6-311+G**	-1.3
M06-2X/6-311+G**	-2.5
LCCSD(T)/aug-cc-pVTZ// ωB97X-D/6-311+G**	-1.6

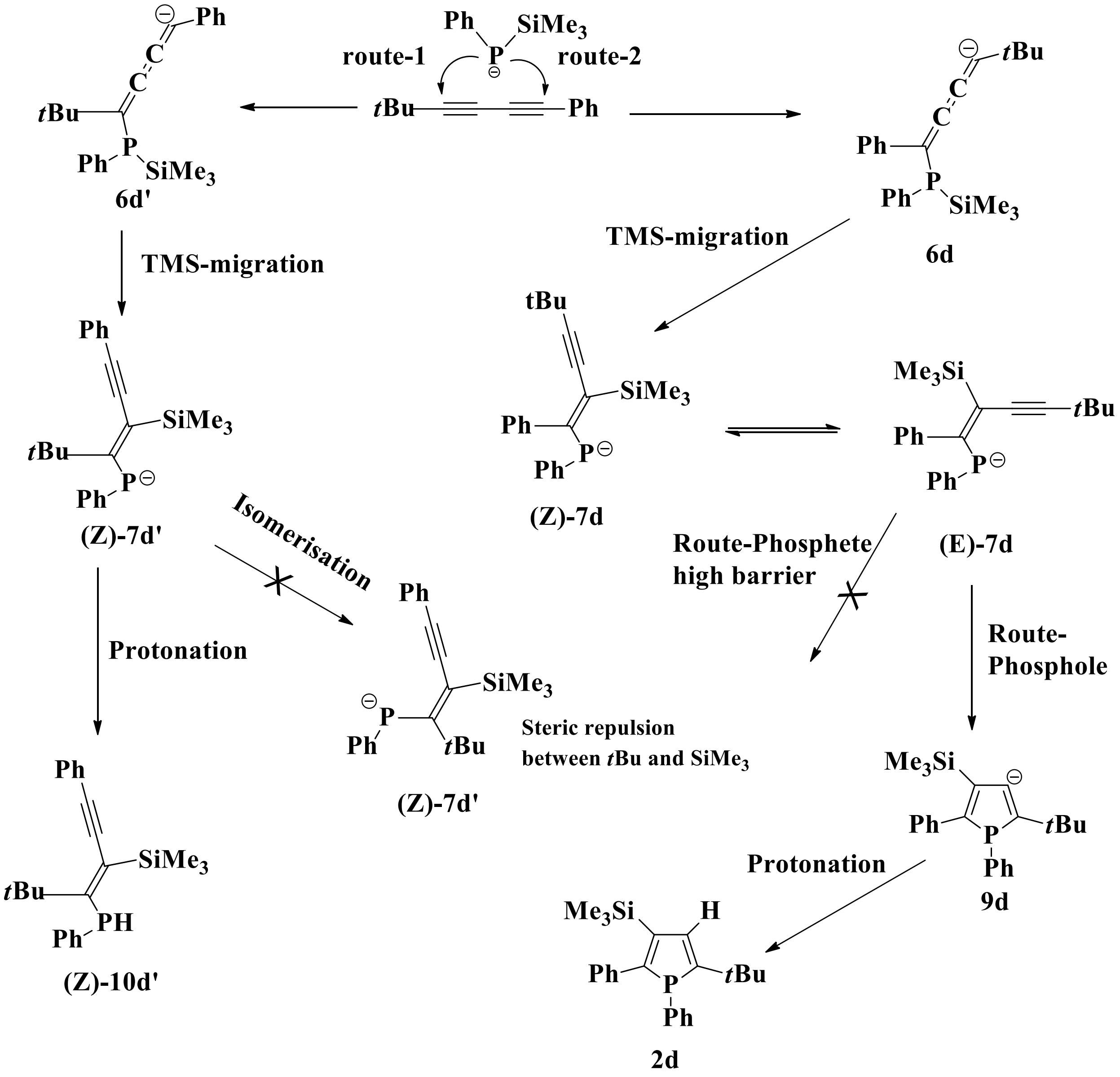

Fig. S75. Schematic representation of the reaction network starting from **1d**

XYZ coordinates and total energies of the investigated systems

Anionic species

a1 + PhTMSP$^-$

E(ωB97X-D/6-311+G**)= -1597.840677

C	5.169304	0.809399	-0.100187
C	5.065745	-0.579443	-0.258957
C	6.225177	-1.330596	-0.487946
C	7.461733	-0.704970	-0.556619
C	7.558749	0.673616	-0.397713
C	6.410213	1.425420	-0.169794
C	3.785790	-1.203661	-0.189720
C	2.671985	-1.668014	-0.126869
C	1.389618	-2.130247	-0.045106
C	0.230294	-2.462454	0.031355
C	-1.160755	-2.757702	0.142951
C	-1.599018	-4.004483	0.606985
C	-2.957030	-4.251019	0.741616
C	-3.881896	-3.260665	0.418367
C	-3.449853	-2.022512	-0.045177
C	-2.092815	-1.766211	-0.187440
P	-1.235995	1.824442	-1.202181
Si	-0.319588	2.056219	0.797326
C	1.555636	2.105262	0.501993
C	-3.003481	1.547341	-0.859109
C	-3.671860	1.654719	0.382141
C	-5.026251	1.381163	0.524646
C	-5.799438	0.991897	-0.565917
C	-5.173703	0.890188	-1.809793
C	-3.821502	1.156600	-1.948343
C	-0.728173	3.659326	1.742692
C	-0.594716	0.641562	2.038412
H	8.353760	-1.295115	-0.736796
H	8.526046	1.160865	-0.453807
H	-3.357732	1.052439	-2.925389
H	-5.748461	0.589086	-2.681945
H	-6.857578	0.780132	-0.452667
H	-5.484057	1.474651	1.506061
H	-3.120055	1.961902	1.263069
H	4.269046	1.388172	0.073719
H	6.479941	2.500461	-0.046263
H	6.142016	-2.403787	-0.614277
H	-0.154370	0.886032	3.013027
H	-1.654523	0.418162	2.190805
H	-0.116598	-0.270721	1.670766
H	-0.189356	3.712010	2.697338
H	-0.445180	4.525585	1.136082
H	-1.798728	3.747691	1.952682
H	2.096434	2.229476	1.447943
H	1.880544	1.169673	0.037029
H	1.827263	2.929545	-0.165447
H	-1.743731	-0.799135	-0.546481
H	-4.163426	-1.244270	-0.293532

H	-4.943601	-3.453356	0.532970
H	-3.295810	-5.215472	1.105581
H	-0.870207	-4.764820	0.864877

TS-1a

E(ωB97X-D/6-311+G**)= -1597.826979

C	5.765365	-1.060369	-1.307747
C	4.995229	-0.686275	-0.191750
C	5.641683	-0.020492	0.865781
C	6.999398	0.253068	0.806171
C	7.751508	-0.124840	-0.302885
C	7.122595	-0.781669	-1.357202
C	3.610167	-0.966944	-0.130864
C	2.411256	-1.181453	-0.074972
C	1.088454	-1.436289	0.030028
C	-0.143605	-1.325067	-0.036170
C	-1.451690	-1.822277	0.321987
C	-1.819081	-1.891275	1.669681
C	-3.074945	-2.358837	2.035773
C	-3.982690	-2.764984	1.065501
C	-3.623269	-2.700632	-0.277592
C	-2.372980	-2.232440	-0.647069
P	-0.985908	0.917062	-1.208419
Si	-0.255935	2.134785	0.512484
C	1.553079	2.533678	0.140944
C	-2.795438	0.859983	-0.963865
C	-3.475342	0.969907	0.263619
C	-4.847793	0.787943	0.362515
C	-5.607407	0.480573	-0.761867
C	-4.962561	0.366089	-1.990862
C	-3.591401	0.553665	-2.086353
C	-1.191792	3.774421	0.687735
C	-0.243279	1.340812	2.239352
H	7.475538	0.768166	1.634324
H	8.813374	0.091264	-0.346681
H	-3.105206	0.457655	-3.053147
H	-5.533452	0.126916	-2.884020
H	-6.678839	0.328763	-0.680767
H	-5.326347	0.869142	1.334204
H	-2.916244	1.171796	1.169656
H	5.278521	-1.567821	-2.132450
H	7.695663	-1.078430	-2.229835
H	5.057988	0.279042	1.728523
H	0.164294	2.048651	2.971646
H	-1.237822	1.039998	2.581169
H	0.392076	0.450467	2.232382
H	-0.791883	4.367964	1.518632
H	-1.100703	4.359943	-0.231876
H	-2.257792	3.607038	0.867950
H	1.988662	3.148933	0.936920
H	2.142256	1.614989	0.059640
H	1.648135	3.074809	-0.805056
H	-2.106441	-2.138790	-1.692995
H	-4.334767	-2.987258	-1.044204
H	-4.970294	-3.112412	1.349770
H	-3.346396	-2.396870	3.086018

H	-1.111361	-1.564642	2.422364

6a

E(ωB97X-D/6-311+G**)= -1597.859808

C	-0.375754	3.018962	-0.388560
C	0.682060	2.153100	-0.708178
C	1.928458	2.732186	-0.978198
C	2.109450	4.110532	-0.936169
C	1.051259	4.952019	-0.619106
C	-0.195398	4.391729	-0.346167
C	0.464015	0.681322	-0.725318
P	1.885705	-0.404914	-1.192835
C	3.000015	-0.305275	0.285495
C	4.313607	-0.772426	0.176809
C	5.180036	-0.737811	1.263389
C	4.754498	-0.210446	2.477792
C	3.456448	0.275556	2.593839
C	2.586430	0.223703	1.511292
C	-0.729861	0.146676	-0.494520
C	-1.884715	-0.366876	-0.269760
C	-2.968720	-1.021413	-0.051774
C	-4.295894	-0.597218	0.268251
C	-5.292277	-0.470394	-0.723863
C	-6.589603	-0.105184	-0.399212
C	-6.959700	0.135381	0.922775
C	-5.990868	0.005408	1.915813
C	-4.689089	-0.353860	1.602825
Si	1.061526	-2.490282	-0.778702
C	-0.243505	-2.948252	-2.046931
C	2.555485	-3.627797	-1.017299
C	0.431757	-2.728846	0.972613
H	-6.252373	0.190800	2.954082
H	-7.978086	0.413423	1.172039
H	4.659401	-1.168072	-0.773848
H	6.192323	-1.115377	1.157559
H	5.431741	-0.173103	3.324816
H	3.114784	0.694866	3.534711
H	1.571463	0.594265	1.610813
H	-5.025256	-0.664120	-1.757385
H	-7.326785	-0.009197	-1.191712
H	-3.947542	-0.452382	2.388579
H	0.294780	-3.798112	1.170731
H	1.146761	-2.336356	1.701882
H	-0.530033	-2.229305	1.108432
H	2.243586	-4.672434	-0.906773
H	3.000611	-3.510655	-2.010079
H	3.329542	-3.428814	-0.270318
H	-0.432665	-4.027010	-2.009007
H	-1.182200	-2.430118	-1.833868
H	0.080965	-2.695538	-3.060887
H	2.772634	2.099148	-1.228271
H	3.089503	4.526348	-1.148852
H	1.193483	6.027124	-0.581398
H	-1.035343	5.032620	-0.095877
H	-1.347036	2.585000	-0.176035

TS-2a

E(ωB97X-D/6-311+G**)= -1597.858403

C	-5.360928	-0.382507	-0.723253
C	-4.385456	-0.337215	0.296455
C	-4.842602	-0.367369	1.632263
C	-6.195287	-0.439617	1.923016
C	-7.145832	-0.483954	0.905449
C	-6.710645	-0.454090	-0.417848
C	-3.013753	-0.266374	-0.002851
C	-1.811524	-0.187156	-0.258845
C	-0.483629	-0.310011	-0.527994
C	0.540336	0.538102	-0.731355
C	0.501983	2.022553	-0.704826
C	-0.673678	2.720603	-0.385224
C	-0.699423	4.107393	-0.349116
C	0.446665	4.847267	-0.627885
C	1.620995	4.174057	-0.942016
C	1.647216	2.785199	-0.977395
P	2.084031	-0.340401	-1.217713
Si	1.222267	-2.408431	-0.746258
C	0.008054	-3.061270	-2.034683
C	3.195917	-0.154630	0.249721
C	4.524248	-0.585604	0.159379
C	5.387212	-0.489944	1.244306
C	4.945440	0.064846	2.441329
C	3.632696	0.513181	2.540664
C	2.765632	0.398787	1.459929
C	2.772682	-3.509934	-0.967927
C	0.709464	-2.729643	1.040349
H	-7.435818	-0.487416	-1.225484
H	-8.203521	-0.542207	1.137780
H	4.882866	-1.003812	-0.776750
H	6.410291	-0.841408	1.151500
H	5.620117	0.149749	3.287101
H	3.276131	0.950091	3.468174
H	1.737677	0.733954	1.551292
H	-4.111598	-0.334813	2.432153
H	-6.513065	-0.462862	2.961231
H	-5.034370	-0.360126	-1.756831
H	0.919030	-3.771897	1.309558
H	1.286709	-2.087699	1.713514
H	-0.349605	-2.519346	1.194436
H	2.511288	-4.564927	-0.821111
H	3.205720	-3.408446	-1.969194
H	3.550060	-3.257676	-0.238928
H	0.131289	-4.142407	-2.169840
H	-1.019014	-2.843977	-1.737495
H	0.184645	-2.578092	-3.000748
H	2.575674	2.280921	-1.223218
H	2.527680	4.730574	-1.158811
H	0.423563	5.931898	-0.596889
H	-1.626466	4.615360	-0.101341
H	-1.576180	2.162595	-0.166660

(Z)-7a

E(ωB97X-D/6-311+G**)= -1597.895240

C	-0.124353	-1.435490	1.083409
C	-0.678881	-0.847234	-0.055568
C	-1.076511	-1.677022	-1.103756
C	-0.927099	-3.055475	-1.015997
C	-0.384172	-3.630996	0.127135
C	0.016481	-2.812889	1.179056
C	-0.836288	0.636022	-0.136586
P	-2.418045	1.364459	-0.436516
C	-3.618540	0.008133	-0.105293
C	-4.681804	-0.169192	-1.002287
C	-5.667347	-1.126694	-0.789038
C	-5.615613	-1.948050	0.331858
C	-4.574988	-1.782181	1.242081
C	-3.604873	-0.811045	1.034487
C	0.334345	1.408866	-0.046051
Si	0.392468	3.270402	0.128523
C	-0.547536	3.852131	1.657142
C	1.582433	0.763194	-0.088338
C	2.667081	0.211510	-0.119961
C	3.858352	-0.550227	-0.127661
C	3.796826	-1.953248	-0.241305
C	4.954761	-2.715087	-0.247594
C	6.205708	-2.110548	-0.140861
C	6.279861	-0.724858	-0.026611
C	5.127134	0.047265	-0.019881
C	2.197691	3.783469	0.342374
C	-0.265566	4.194501	-1.379903
H	7.247333	-0.239649	0.059149
H	7.109561	-2.710420	-0.146912
H	-4.721124	0.449500	-1.894481
H	-6.471107	-1.240639	-1.510801
H	-6.375150	-2.705802	0.495859
H	-4.522088	-2.410548	2.126221
H	-2.813996	-0.688099	1.765636
H	2.822576	-2.423494	-0.322125
H	4.880119	-3.794338	-0.338526
H	5.190449	1.126009	0.070835
H	-1.514476	-1.225944	-1.986880
H	-1.243378	-3.683601	-1.842593
H	-0.272817	-4.708151	0.199379
H	0.446566	-3.249226	2.074977
H	0.200513	-0.793987	1.895196
H	-0.523384	4.944761	1.738832
H	-1.590386	3.528693	1.611934
H	-0.097241	3.430988	2.561647
H	2.272826	4.867618	0.482997
H	2.646105	3.289894	1.209632
H	2.790987	3.506473	-0.534056
H	-0.135755	5.275992	-1.255500
H	0.274068	3.886299	-2.281101
H	-1.326688	3.986037	-1.536010

TS-3a

E(ωB97X-D/6-311+G**)= -1597.864608

C	-2.534614	0.301559	1.492752
C	-1.241059	-0.011741	1.068326

C	-0.396666	-0.697183	1.949438
C	-0.854016	-1.097751	3.197228
C	-2.149509	-0.790349	3.605010
C	-2.983197	-0.077609	2.751441
C	-0.726078	0.402331	-0.266357
P	-1.473174	0.151007	-1.756749
C	-3.011674	-0.822545	-1.390019
C	-2.988270	-2.044124	-0.708071
C	-4.155245	-2.765315	-0.494870
C	-5.373229	-2.284442	-0.966998
C	-5.411109	-1.082481	-1.663673
C	-4.237998	-0.366011	-1.879460
C	0.576268	1.127260	-0.293793
Si	0.591541	2.951894	-0.142298
C	0.102361	3.849376	-1.739604
C	1.743451	0.408135	-0.289188
C	2.794665	-0.236338	-0.259967
C	3.990190	-0.961197	-0.292307
C	5.252011	-0.322533	-0.198234
C	6.430726	-1.048664	-0.225787
C	6.420460	-2.438433	-0.345388
C	5.188413	-3.085482	-0.438682
C	4.001275	-2.373506	-0.413971
C	-0.649678	3.539113	1.164712
C	2.319355	3.538035	0.334474
H	-0.625914	4.628716	1.282234
H	3.051994	-2.891951	-0.494345
H	7.378044	-0.521572	-0.150917
H	7.347684	-3.000753	-0.366676
H	-2.046983	-2.419948	-0.320762
H	-4.115275	-3.704543	0.047250
H	-6.286334	-2.845098	-0.795384
H	-6.354622	-0.700694	-2.041076
H	-4.277227	0.572026	-2.426700
H	5.152933	-4.167137	-0.536838
H	5.278656	0.757407	-0.100381
H	0.622195	-0.906404	1.642030
H	-0.190553	-1.643931	3.859952
H	-2.501934	-1.095064	4.585230
H	-3.990095	0.181192	3.062723
H	-3.190513	0.850173	0.826507
H	2.364077	4.629877	0.413335
H	2.620787	3.104472	1.292419
H	3.048837	3.214016	-0.413723
H	0.031287	4.934397	-1.595710
H	0.836061	3.650753	-2.527158
H	-0.867554	3.487990	-2.097356
H	-1.669163	3.255400	0.881621
H	-0.441354	3.078426	2.135063

(E)-7a

E(ωB97X-D/6-311+G**)= -1597.894521

C	2.809505	0.718340	-1.053164
C	1.896021	0.745107	0.002382
C	2.261611	1.398190	1.180364
C	3.498926	2.019794	1.299395

C	4.394502	1.997992	0.236499
C	4.043628	1.341772	-0.939810
C	0.562575	0.092141	-0.107221
P	0.426708	-1.652687	-0.332370
C	2.160659	-2.249930	-0.090981
C	2.719889	-3.103384	-1.052121
C	3.986186	-3.657384	-0.892567
C	4.740807	-3.365623	0.238110
C	4.201715	-2.528936	1.211861
C	2.929315	-1.996405	1.055573
C	-0.563481	0.934463	-0.063080
Si	-0.585326	2.798626	-0.177454
C	-2.238358	3.317414	-0.930903
C	-1.840835	0.350037	-0.019944
C	-2.955827	-0.133916	0.034886
C	-4.223443	-0.762266	0.088112
C	-5.415968	-0.023730	-0.013838
C	-6.650669	-0.653575	0.044128
C	-6.735120	-2.034080	0.202429
C	-5.560809	-2.777851	0.300774
C	-4.322630	-2.157420	0.246051
C	-0.487031	3.664019	1.504909
C	0.773584	3.536191	-1.262837
H	-7.557209	-0.061446	-0.035923
H	-7.701808	-2.524043	0.249182
H	2.149213	-3.323390	-1.950030
H	4.388272	-4.311596	-1.660962
H	5.732788	-3.788222	0.362785
H	4.773644	-2.296667	2.105448
H	2.519929	-1.363895	1.835236
H	-3.407046	-2.732754	0.323397
H	-5.611973	-3.855231	0.423834
H	-5.353635	1.051325	-0.141329
H	2.535397	0.196827	-1.963144
H	4.739265	1.313752	-1.772203
H	5.361419	2.482298	0.325799
H	3.764635	2.519253	2.225836
H	1.561402	1.402017	2.008690
H	0.557254	4.595080	-1.447685
H	1.760524	3.466101	-0.798856
H	0.822691	3.024113	-2.228269
H	-2.286449	4.405795	-1.048649
H	-2.380688	2.854949	-1.912147
H	-3.070921	2.999740	-0.297057
H	-0.684318	4.737411	1.400883
H	-1.228314	3.246524	2.193703
H	0.501339	3.545839	1.957307

TS-5a

E(ωB97X-D/6-311+G**)= -1597.861445

C	-3.583616	-0.014621	0.422979
C	-3.181506	-1.336911	0.141382
C	-4.169780	-2.234751	-0.304585
C	-5.483698	-1.827885	-0.480325
C	-5.865595	-0.517192	-0.196317
C	-4.903886	0.381239	0.257803

C	-1.815079	-1.731215	0.306552
C	-0.720963	-1.219827	-0.098283
C	0.723906	-1.259515	-0.072761
Si	1.616819	-2.875889	0.295411
C	0.596858	-4.293912	-0.397438
C	1.165386	-0.037361	-0.498197
C	2.524340	0.509400	-0.421647
C	3.330301	0.268106	0.698597
C	4.607866	0.802670	0.795310
C	5.106291	1.609970	-0.221596
C	4.308224	1.880680	-1.329313
C	3.032654	1.341703	-1.426446
P	-0.308717	0.702211	-1.265715
C	-0.577410	2.229857	-0.287711
C	0.046809	2.502179	0.939092
C	-0.237208	3.660674	1.652091
C	-1.171175	4.574641	1.174521
C	-1.814745	4.314086	-0.033449
C	-1.516275	3.165974	-0.752902
C	3.323862	-2.942470	-0.505459
C	1.782024	-3.115030	2.159043
H	-5.180296	1.408510	0.477161
H	-6.896501	-0.203870	-0.326376
H	-2.021463	2.978639	-1.696508
H	-2.550289	5.014651	-0.418007
H	-1.398845	5.474839	1.736239
H	0.266163	3.843707	2.596921
H	0.756043	1.787768	1.342888
H	1.080301	-5.259017	-0.208208
H	-0.395681	-4.287548	0.060051
H	0.460830	-4.179566	-1.476998
H	2.179618	-4.107780	2.396597
H	2.447101	-2.369896	2.606259
H	0.798596	-3.015654	2.627785
H	3.758877	-3.939581	-0.372347
H	3.243236	-2.749360	-1.579619
H	4.016498	-2.207498	-0.090011
H	-3.882252	-3.259904	-0.514475
H	-6.221542	-2.541354	-0.836488
H	-2.838756	0.694491	0.765944
H	2.930120	-0.329888	1.510483
H	5.210556	0.600161	1.675083
H	6.100646	2.037330	-0.144631
H	4.681445	2.521021	-2.122251
H	2.405376	1.556143	-2.285175

8a

E(ωB97X-D/6-311+G**)= -1597.868752

C	2.999518	1.414205	-1.746906
C	2.626837	0.670358	-0.618480
C	3.542648	0.584566	0.438202
C	4.795582	1.176321	0.353086
C	5.159321	1.890076	-0.784088
C	4.249284	2.011114	-1.830509
C	1.306230	0.056881	-0.529754
P	-0.319921	0.602164	-1.196825

C	-0.801996	1.975158	-0.055766
C	-1.811344	2.856155	-0.452370
C	-2.255250	3.866206	0.393545
C	-1.681288	4.025448	1.650041
C	-0.666906	3.161805	2.053898
C	-0.236020	2.145393	1.211057
C	-0.596939	-1.001057	-0.177821
C	0.862222	-1.119173	0.002722
Si	1.651590	-2.711862	0.620559
C	1.216032	-2.939224	2.435389
C	-1.630063	-1.731086	0.226243
C	-2.980263	-1.325091	-0.081995
C	-3.781954	-2.064257	-0.978172
C	-5.081476	-1.682988	-1.274029
C	-5.652909	-0.562533	-0.671101
C	-4.884378	0.173026	0.228438
C	-3.577597	-0.194465	0.517440
C	0.938357	-4.131209	-0.382606
C	3.530899	-2.748491	0.410198
H	-5.304154	1.055982	0.703244
H	-6.673977	-0.271971	-0.896466
H	-2.268305	2.731906	-1.429389
H	-3.050501	4.530755	0.070540
H	-2.023138	4.814195	2.312644
H	-0.215497	3.275894	3.034633
H	0.536364	1.457513	1.540209
H	1.347586	-5.094272	-0.056977
H	-0.147460	-4.132153	-0.257084
H	1.156956	-4.004403	-1.447521
H	1.590897	-3.891353	2.826913
H	1.628445	-2.130885	3.047985
H	0.126337	-2.917453	2.530008
H	3.883577	-3.782656	0.497101
H	3.827718	-2.369934	-0.572456
H	4.054423	-2.153468	1.162604
H	-3.354782	-2.948117	-1.441847
H	-5.662175	-2.270656	-1.980451
H	-2.988236	0.399617	1.208158
H	3.249689	0.058108	1.339762
H	5.488586	1.090416	1.184193
H	6.135829	2.358196	-0.849541
H	4.516172	2.575088	-2.718717
H	2.290920	1.510435	-2.562970

TS-4a

E(ωB97X-D/6-311+G**)= -1597.852762

C	3.496397	0.429405	-1.585522
C	2.816065	1.181467	-0.622167
C	3.571077	1.971765	0.259361
C	4.954596	2.003268	0.175793
C	5.620808	1.244512	-0.783532
C	4.882349	0.456829	-1.659432
C	1.381526	1.162089	-0.513575
C	0.328102	1.573334	0.034146
C	-1.002945	1.036483	0.004554
Si	-2.405151	2.158223	0.567551

C	-2.766299	1.924196	2.406639
C	-1.104551	-0.233733	-0.514571
P	0.316703	-1.081494	-1.288016
C	1.274049	-1.690916	0.142568
C	1.000624	-1.427005	1.494886
C	1.799429	-1.937927	2.508000
C	2.908992	-2.727048	2.215306
C	3.199088	-2.998901	0.880968
C	2.393850	-2.497381	-0.130953
C	-2.414316	-0.925260	-0.577115
C	-3.056693	-1.172376	-1.796482
C	-4.309412	-1.764001	-1.834403
C	-4.948353	-2.142930	-0.654724
C	-4.309669	-1.930273	0.560197
C	-3.055459	-1.329643	0.597261
C	-1.849736	3.939668	0.303790
C	-4.013103	1.928759	-0.392391
H	5.389262	-0.143424	-2.408361
H	6.704090	1.266185	-0.844605
H	2.631548	-2.731937	-1.165228
H	4.061398	-3.608291	0.625114
H	3.537301	-3.116810	3.009594
H	1.559536	-1.706425	3.542236
H	0.161615	-0.787761	1.748841
H	3.045788	2.551310	1.009797
H	5.519890	2.618030	0.869448
H	2.909406	-0.180660	-2.262293
H	-2.620768	4.644312	0.635008
H	-0.925885	4.142090	0.851374
H	-1.647279	4.124287	-0.755772
H	-3.462179	2.687940	2.771419
H	-3.212125	0.943236	2.594106
H	-1.842380	1.995573	2.988502
H	-4.722569	2.716765	-0.113981
H	-3.831094	2.002987	-1.468832
H	-4.481882	0.960378	-0.202349
H	-2.555399	-1.169873	1.546209
H	-4.788791	-2.230918	1.486760
H	-5.929101	-2.605817	-0.686946
H	-4.795795	-1.929089	-2.790745
H	-2.561509	-0.881098	-2.716392

9a

E(ωB97X-D/6-311+G**)= -1597.888568

C	2.777430	0.929938	-1.855579
C	2.232477	0.489122	-0.643984
C	2.935292	0.769007	0.532717
C	4.154372	1.433354	0.499642
C	4.695052	1.844746	-0.713353
C	3.997538	1.592594	-1.890892
C	0.933685	-0.200235	-0.578326
C	0.636688	-1.423516	-0.060191
C	-0.803092	-1.794406	0.034149
C	-1.569724	-0.770650	-0.449872
C	-3.041838	-0.745831	-0.458998
C	-3.770702	-1.814732	0.091405

C	-5.155313	-1.801374	0.123831
C	-5.871177	-0.718786	-0.389038
C	-5.168517	0.347918	-0.935241
C	-3.777769	0.333869	-0.968834
P	-0.588788	0.591211	-1.187280
C	-0.695499	1.963982	0.053909
C	-0.995940	1.711181	1.395380
C	-1.028035	2.746147	2.319739
C	-0.759841	4.053623	1.920535
C	-0.461974	4.316806	0.589893
C	-0.435232	3.277351	-0.336751
Si	1.802247	-2.776007	0.515727
C	3.636732	-2.469663	0.160392
C	1.584727	-3.017597	2.373184
C	1.333769	-4.371046	-0.369172
H	-5.703402	1.202822	-1.338740
H	-6.956308	-0.708932	-0.359840
H	-0.204544	3.485563	-1.377029
H	-0.251404	5.332431	0.269463
H	-0.785393	4.861297	2.645199
H	-1.262365	2.533638	3.358098
H	-1.201394	0.688661	1.698476
H	-3.199791	-2.646608	0.489109
H	-5.687265	-2.644686	0.555821
H	-3.254163	1.181064	-1.402048
H	1.906039	-5.225599	0.009573
H	0.266107	-4.553556	-0.224978
H	1.517593	-4.283315	-1.444902
H	2.143367	-3.885985	2.739488
H	1.923945	-2.134556	2.924703
H	0.522372	-3.164390	2.587339
H	4.196332	-3.402201	0.298821
H	3.789312	-2.132251	-0.869619
H	4.070464	-1.711490	0.816761
H	2.501235	0.463038	1.478547
H	4.680575	1.636585	1.426966
H	5.647476	2.363841	-0.741576
H	4.407571	1.914520	-2.843071
H	2.235360	0.735219	-2.774808

1b + PhTMSP$^-$

E(ωB97X-D/6-311+G**)= -1751.463708

C	-4.608906	1.497616	-1.912875
C	-3.729731	1.766515	-0.834410
C	-4.359216	1.949493	0.418277
C	-5.735604	1.853389	0.580490
C	-6.569887	1.578251	-0.499667
C	-5.981968	1.408635	-1.754614
P	-1.946482	1.806285	-1.205455
Si	-0.978429	1.922883	0.778535
C	-1.169985	3.563719	1.727764
C	-1.408354	0.551690	2.024891
C	0.884065	1.734917	0.453688
C	-1.066678	-2.646753	0.018528
C	0.126837	-2.467674	-0.041903

C	1.458914	-2.175527	-0.101651
C	2.624009	-1.857362	-0.140885
C	3.976898	-1.413038	-0.180660
C	5.038928	-2.351027	-0.321263
C	6.336927	-1.930420	-0.358009
C	6.660760	-0.552383	-0.257542
C	5.605334	0.388031	-0.118381
C	4.266951	-0.069029	-0.082300
C	5.922674	1.767887	-0.017929
C	7.224099	2.189748	-0.053430
C	8.274078	1.253095	-0.192073
C	7.997964	-0.084285	-0.291868
C	-2.484965	-2.762249	0.111203
C	-3.275851	-1.644339	-0.181935
C	-4.656621	-1.728898	-0.062565
C	-5.251001	-2.919856	0.340521
C	-4.466735	-4.035162	0.627039
C	-3.086463	-3.960697	0.515418
H	-4.177377	1.344673	-2.898257
H	-6.604168	1.192934	-2.619446
H	-7.644374	1.501274	-0.370146
H	-6.162420	1.994998	1.570105
H	-3.759058	2.173888	1.292644
H	4.796716	-3.404315	-0.400890
H	7.140473	-2.652295	-0.465667
H	3.458601	0.647099	0.023549
H	8.801676	-0.806377	-0.398679
H	9.301304	1.600243	-0.220313
H	5.112309	2.481873	0.086233
H	7.454012	3.246536	0.025175
H	-0.953767	0.753643	3.002723
H	-2.486583	0.437206	2.168777
H	-1.022773	-0.406684	1.665125
H	-0.610275	3.550160	2.671632
H	-0.797659	4.390226	1.114225
H	-2.218137	3.778341	1.958819
H	1.451769	1.783538	1.390834
H	1.077224	0.767312	-0.019335
H	1.246874	2.521054	-0.216596
H	-2.465748	-4.820141	0.743427
H	-4.932333	-4.963026	0.942427
H	-6.330292	-2.979757	0.435798
H	-5.259695	-0.854628	-0.282768
H	-2.799911	-0.718147	-0.500234

TS-1b

E(ωB97X-D/6-311+G**)= -1751.451129

C	-4.554879	0.416256	-2.166730
C	-3.764442	0.807966	-1.066823
C	-4.459448	1.076368	0.127542
C	-5.839605	0.960132	0.217550
C	-6.593500	0.565336	-0.883101
C	-5.934136	0.295660	-2.079818
P	-1.951069	0.764909	-1.278341
Si	-1.198287	2.131541	0.315753
C	-2.065845	3.817107	0.284370

C	-1.256333	1.538571	2.120455
C	0.633810	2.409679	-0.054002
C	-1.225153	-1.394864	0.183898
C	0.000718	-1.545481	0.251883
C	1.328363	-1.359979	0.086684
C	2.530696	-1.201013	-0.037829
C	3.913712	-0.992775	-0.234624
C	4.568784	-1.615500	-1.343274
C	5.900520	-1.432543	-1.568021
C	6.685563	-0.617071	-0.709846
C	6.047242	0.008912	0.395849
C	4.665429	-0.194698	0.610498
C	6.829614	0.828424	1.252858
C	8.167003	1.013638	1.024525
C	8.798218	0.389210	-0.075084
C	8.068911	-0.406910	-0.919827
C	-2.563663	-1.768244	0.570428
C	-3.486060	-2.250969	-0.364228
C	-4.767007	-2.603006	0.027536
C	-5.156812	-2.475241	1.357611
C	-4.248333	-1.996177	2.293096
C	-2.961068	-1.645633	1.905785
H	-4.058158	0.200586	-3.108566
H	-6.500521	-0.013281	-2.954233
H	-7.671310	0.464023	-0.808055
H	-6.329188	1.160551	1.166227
H	-3.906680	1.350029	1.018419
H	3.976278	-2.235839	-2.005542
H	6.378927	-1.910980	-2.417480
H	4.182795	0.289947	1.452154
H	8.545845	-0.888015	-1.768766
H	9.857699	0.544160	-0.247648
H	6.344972	1.309581	2.096647
H	8.748675	1.644350	1.688497
H	-0.816936	2.299514	2.777020
H	-2.272554	1.339097	2.472396
H	-0.677517	0.616910	2.228952
H	-1.668152	4.482918	1.059802
H	-1.923679	4.295667	-0.689055
H	-3.142816	3.709323	0.444534
H	1.084684	3.077261	0.689584
H	1.181439	1.462255	-0.038200
H	0.768839	2.855996	-1.043609
H	-2.252576	-1.264233	2.631333
H	-4.543471	-1.882817	3.331374
H	-6.168449	-2.728909	1.655840
H	-5.477049	-2.948663	-0.715591
H	-3.192846	-2.307572	-1.405630

6b

E(ωB97X-D/6-311+G**)= -1751.485785

C	3.016260	2.720588	-0.958001
C	1.744378	2.153151	-0.813665
C	0.665723	3.028205	-0.612383
C	0.850670	4.400257	-0.564871
C	2.122670	4.949430	-0.714953

C	3.201937	4.098113	-0.911899
C	1.517653	0.681979	-0.842200
P	2.986572	-0.417106	-1.084297
Si	2.108579	-2.498536	-0.772510
C	3.619887	-3.636433	-0.815944
C	0.300515	0.159393	-0.761410
C	-0.872020	-0.357858	-0.678058
C	-1.974017	-1.005995	-0.587754
C	-3.344742	-0.661592	-0.458486
C	-3.969572	-0.466448	0.770691
C	-5.350034	-0.196683	0.872264
C	-6.146972	-0.114867	-0.305793
C	-5.501634	-0.313247	-1.556916
C	-4.168622	-0.583969	-1.632707
C	-7.529150	0.155359	-0.195135
C	-8.122156	0.343583	1.028833
C	-7.336814	0.266868	2.201335
C	-5.993941	0.004744	2.124984
C	3.861960	-0.291623	0.545043
C	5.180840	-0.745590	0.645684
C	5.867254	-0.693984	1.853631
C	5.253116	-0.162697	2.982372
C	3.948082	0.310066	2.890914
C	3.257873	0.241326	1.687154
C	1.258162	-2.730064	0.883580
C	0.979245	-2.960319	-2.198683
H	5.673551	-1.143790	-0.236647
H	6.887136	-1.061409	1.910840
H	5.788480	-0.112052	3.924782
H	3.460933	0.731556	3.764241
H	2.235719	0.600001	1.624886
H	-3.697052	-0.743626	-2.596543
H	-6.093381	-0.248952	-2.466495
H	-3.379752	-0.529254	1.679702
H	-8.120726	0.213495	-1.105206
H	-9.184673	0.550473	1.100384
H	-5.396177	-0.052929	3.029974
H	-7.804056	0.416680	3.169798
H	1.093456	-3.798421	1.064359
H	1.875661	-2.338646	1.697373
H	0.288471	-2.227799	0.897612
H	3.298849	-4.678911	-0.711510
H	4.165515	-3.543415	-1.759889
H	4.311769	-3.416012	0.002212
H	0.811443	-4.043246	-2.201947
H	0.010288	-2.465229	-2.096978
H	1.421263	-2.682762	-3.160264
H	-0.325198	2.602679	-0.494251
H	-0.005563	5.049031	-0.407926
H	2.268066	6.023937	-0.674068
H	4.201335	4.505762	-1.028471
H	3.876646	2.078786	-1.112135

TS-2b

E(ωB97X-D/6-311+G**)= -1751.483262

C	-6.123209	-0.246483	2.140691

C	-5.443781	-0.207296	0.892559
C	-6.224523	-0.186085	-0.296807
C	-7.635142	-0.204198	-0.201667
C	-8.262480	-0.241843	1.017757
C	-7.491507	-0.263230	2.201896
C	-5.542992	-0.147766	-1.543193
C	-4.182052	-0.131332	-1.605439
C	-3.379184	-0.150424	-0.417260
C	-4.034814	-0.188818	0.806560
C	-1.978321	-0.130861	-0.526229
C	-0.754871	-0.070801	-0.637247
C	0.595585	-0.227262	-0.752783
C	1.656688	0.598274	-0.798507
P	3.193052	-0.356635	-1.135944
Si	2.030531	-2.324635	-0.851745
C	1.306209	-2.673724	0.858531
C	1.662953	2.078380	-0.711350
C	2.861181	2.800795	-0.803012
C	2.881552	4.186671	-0.706379
C	1.701707	4.894590	-0.510436
C	0.502620	4.193942	-0.412061
C	0.482117	2.809925	-0.508978
C	4.115203	-0.306729	0.464878
C	3.587519	0.262186	1.628743
C	4.314573	0.269626	2.813604
C	5.580575	-0.303360	2.867094
C	6.117763	-0.875868	1.718169
C	5.397358	-0.865196	0.530298
C	0.952739	-2.855006	-2.312517
C	3.491724	-3.573997	-0.939427
H	-6.127684	-0.132234	-2.458649
H	5.832026	-1.298916	-0.365676
H	7.105952	-1.324743	1.744979
H	6.144743	-0.301947	3.794050
H	3.884294	0.720203	3.702508
H	2.592959	0.694823	1.602207
H	-3.450124	-0.205757	1.719984
H	-3.675262	-0.102997	-2.563319
H	1.520952	-3.703196	1.168130
H	1.756762	-2.001104	1.595345
H	0.227294	-2.507041	0.870125
H	3.116237	-4.601407	-0.852383
H	4.036452	-3.497822	-1.887613
H	4.209427	-3.416038	-0.126672
H	1.071846	-3.923900	-2.524838
H	-0.098426	-2.638008	-2.111717
H	1.239071	-2.297919	-3.210303
H	3.792409	2.265165	-0.954099
H	3.827285	4.714239	-0.781851
H	1.715063	5.976884	-0.432416
H	-0.428297	4.730551	-0.257075
H	-0.459990	2.281000	-0.428934
H	-5.533473	-0.263322	3.052141
H	-7.989763	-0.293405	3.165494
H	-9.345412	-0.255527	1.077164
H	-8.218694	-0.187820	-1.117935

(Z)-7b

E(ωB97X-D/6-311+G**)= -1751.519850

C	0.941887	-1.351984	1.171555
C	1.522820	-0.844717	0.007042
C	1.794509	-1.727118	-1.038175
C	1.500702	-3.079923	-0.921376
C	0.935985	-3.576729	0.247854
C	0.657138	-2.704644	1.295828
C	1.840628	0.610129	-0.101968
P	3.483121	1.158434	-0.428396
C	4.543527	-0.319983	-0.146814
C	5.564896	-0.574538	-1.073177
C	6.466928	-1.618163	-0.899983
C	6.369341	-2.450995	0.209142
C	5.367675	-2.212061	1.146316
C	4.481183	-1.156803	0.978221
C	0.760971	1.509706	-0.006449
Si	0.907963	3.370589	0.127356
C	-0.825922	4.084566	0.349191
C	-0.546778	1.004547	-0.026486
C	-1.689618	0.581816	-0.044772
C	-2.959654	-0.029122	-0.070804
C	-4.142910	0.688415	-0.001297
C	-5.400753	0.046770	-0.040658
C	-5.463806	-1.370168	-0.153180
C	-4.242110	-2.094731	-0.216788
C	-3.038774	-1.456281	-0.177065
C	-6.620448	0.772681	0.025120
C	-7.829706	0.131009	-0.021643
C	-7.888346	-1.276183	-0.136887
C	-6.727182	-2.004120	-0.199959
C	1.929378	3.874702	1.630916
C	1.645155	4.179459	-1.410261
H	3.719989	-0.979670	1.729409
H	5.280285	-2.849164	2.021322
H	7.065354	-3.272725	0.344198
H	7.241687	-1.788413	-1.641928
H	5.639799	0.056279	-1.954481
H	1.642887	5.271124	-1.309359
H	2.672738	3.846478	-1.574608
H	1.060219	3.915954	-2.297285
H	-0.776816	5.172442	0.470519
H	-1.456923	3.860878	-0.515950
H	-1.315983	3.659638	1.230087
H	2.049043	4.963019	1.676752
H	1.437481	3.545007	2.551529
H	2.921091	3.417543	1.590817
H	-2.108990	-2.013386	-0.225603
H	-4.280519	-3.177050	-0.301751
H	-4.102216	1.769451	0.082709
H	-6.763581	-3.086195	-0.289792
H	-8.850559	-1.775403	-0.175775
H	-6.575693	1.853966	0.113741
H	-8.750319	0.703360	0.029108
H	2.250090	-1.338819	-1.941999

H	1.722853	-3.750870	-1.744883
H	0.715896	-4.635107	0.343707
H	0.211439	-3.079576	2.211677
H	0.712162	-0.666745	1.980129

TS-3b

E(ωB97X-D/6-311+G**)= -1751.491105

C	7.386873	-0.539550	-0.135862
C	6.014592	-0.210183	-0.139018
C	5.040049	-1.250589	-0.173892
C	5.507684	-2.595151	-0.205463
C	6.847282	-2.883901	-0.202255
C	7.807075	-1.847654	-0.166608
C	5.547289	1.134059	-0.110012
C	4.217478	1.423625	-0.114576
C	3.214717	0.390464	-0.151996
C	3.668706	-0.931586	-0.179949
C	1.865811	0.738287	-0.148959
C	0.679889	1.073430	-0.236049
C	-0.630911	1.452193	-0.307582
Si	-1.091544	3.227146	-0.235446
C	-1.826532	3.887392	-1.851805
C	-1.699850	0.413088	-0.266318
P	-2.333618	-0.061455	-1.754332
C	-3.580257	-1.383556	-1.377653
C	-3.268182	-2.533028	-0.643313
C	-4.216401	-3.525417	-0.435067
C	-5.497301	-3.394388	-0.963837
C	-5.818004	-2.267436	-1.711102
C	-4.862705	-1.277769	-1.921609
C	-2.112013	-0.078573	1.077576
C	-1.139337	-0.508173	1.987483
C	-1.503575	-0.967909	3.245490
C	-2.840751	-0.977212	3.633314
C	-3.810642	-0.520637	2.748971
C	-3.449347	-0.081756	1.481851
C	-2.411827	3.531624	1.089053
C	0.437505	4.255199	0.163163
H	-2.665357	4.594914	1.170360
H	2.941019	-1.735843	-0.212080
H	6.277450	1.938889	-0.083755
H	-2.277722	-2.638844	-0.213411
H	-3.956180	-4.403665	0.146796
H	-6.238881	-4.168912	-0.797564
H	-6.811824	-2.157855	-2.133611
H	-5.121086	-0.401948	-2.510800
H	3.880188	2.454380	-0.089548
H	-0.095338	-0.471722	1.696374
H	-0.736464	-1.309984	3.932590
H	-3.121435	-1.327708	4.621255
H	-4.855098	-0.507610	3.043087
H	-4.210012	0.268979	0.794008
H	0.876125	3.939790	1.114216
H	1.199684	4.117803	-0.609701
H	0.195522	5.321832	0.225098
H	-2.140471	4.933759	-1.755066

H	-1.092040	3.819227	-2.660144
H	-2.698603	3.296111	-2.149542
H	-3.330269	2.985387	0.848876
H	-2.068966	3.178575	2.066361
H	4.775052	-3.396391	-0.233639
H	7.176363	-3.918335	-0.228295
H	8.114894	0.267254	-0.109729
H	8.865455	-2.085631	-0.164758

(E)-7b

E(ωB97X-D/6-311+G**)= -1751.519403

C	-3.336751	1.211210	1.217147
C	-2.942285	0.617988	0.017057
C	-3.880265	0.511967	-1.011592
C	-5.167605	1.003683	-0.851535
C	-5.547397	1.603031	0.345942
C	-4.626812	1.700535	1.382908
C	-1.550625	0.112567	-0.142129
P	-1.233501	-1.599931	-0.407909
C	-2.887779	-2.378666	-0.125812
C	-3.409402	-3.246055	-1.094737
C	-4.616628	-3.912076	-0.905957
C	-5.344790	-3.724953	0.263375
C	-4.838386	-2.879231	1.246599
C	-3.623995	-2.233514	1.059583
C	-0.520857	1.073643	-0.105337
Si	-0.707011	2.931586	-0.183142
C	0.861974	3.642463	-0.958524
C	0.809405	0.631407	-0.089135
C	1.970958	0.267415	-0.051124
C	3.280833	-0.256667	0.003616
C	4.411651	0.540043	-0.060818
C	5.710559	-0.012270	0.013223
C	5.867963	-1.418967	0.153585
C	4.698148	-2.225381	0.208211
C	3.455066	-1.671760	0.137068
C	6.878470	0.794766	-0.043452
C	8.127910	0.238623	0.037518
C	8.280265	-1.158772	0.179495
C	7.170718	-1.963816	0.234714
C	-2.161722	3.528765	-1.229415
C	-0.865286	3.748492	1.518676
H	-3.237546	-1.591801	1.843560
H	-5.389320	-2.727815	2.170047
H	-6.292877	-4.232790	0.409163
H	-4.994303	-4.571985	-1.681730
H	-2.861555	-3.384782	-2.022530
H	-0.742701	4.834949	1.439239
H	-1.842423	3.551495	1.968164
H	-0.095609	3.368618	2.197945
H	0.783898	4.730944	-1.057040
H	1.739824	3.410405	-0.349169
H	1.032098	3.215338	-1.951303
H	-2.065847	4.607190	-1.402019
H	-2.173780	3.027516	-2.201644
H	-3.125164	3.344201	-0.747598

H	2.563773	-2.287714	0.180807
H	4.809571	-3.300795	0.312080
H	4.298645	1.613737	-0.168353
H	7.279595	-3.039017	0.344509
H	9.273068	-1.590906	0.244934
H	6.761607	1.868669	-0.153081
H	9.007506	0.872776	-0.006356
H	-3.582135	0.035391	-1.938446
H	-5.881696	0.917245	-1.663992
H	-6.554767	1.986493	0.471016
H	-4.913827	2.156890	2.325031
H	-2.615391	1.275674	2.024498

TS-5b

E(ωB97X-D/6-311+G**)= -1751.489185

C	-4.552806	0.947386	0.540102
C	-4.008623	-0.341236	0.288177
C	-4.873612	-1.358919	-0.203134
C	-6.240422	-1.061626	-0.412064
C	-6.739473	0.191592	-0.157884
C	-5.880404	1.207005	0.320313
C	-4.316171	-2.640069	-0.468393
C	-2.990792	-2.889875	-0.260086
C	-2.102994	-1.875056	0.214223
C	-2.639812	-0.623547	0.488971
C	-0.706368	-2.115033	0.384972
C	0.307624	-1.467916	-0.025016
C	1.727684	-1.223348	-0.024590
Si	2.924663	-2.644201	0.270244
C	3.152837	-2.930902	2.119598
C	1.914162	0.069829	-0.438837
P	0.336766	0.502078	-1.223453
C	-0.235438	1.998193	-0.334529
C	-1.384888	2.642461	-0.824208
C	-1.939751	3.734178	-0.173863
C	-1.354134	4.232328	0.988224
C	-0.213106	3.611611	1.487358
C	0.332710	2.506902	0.843572
C	3.144392	0.866474	-0.370449
C	3.998355	0.781493	0.736754
C	5.153493	1.548453	0.813249
C	5.473644	2.434528	-0.209952
C	4.621175	2.549315	-1.304298
C	3.469462	1.779279	-1.381315
C	2.179501	-4.195957	-0.485287
C	4.601023	-2.341924	-0.539425
H	-1.858389	2.259862	-1.724169
H	-2.835954	4.198612	-0.574417
H	-1.784422	5.087567	1.498999
H	0.251108	3.983033	2.396299
H	1.205733	2.021705	1.267078
H	2.822821	-5.067402	-0.320561
H	1.197256	-4.383403	-0.043709
H	2.036823	-4.068891	-1.562603
H	3.747807	-3.830396	2.311754
H	3.652259	-2.084816	2.601321

H	2.174487	-3.058767	2.592350
H	5.213352	-3.248652	-0.477099
H	4.469987	-2.094176	-1.597197
H	5.152218	-1.521499	-0.075102
H	-2.578712	-3.872847	-0.463898
H	-4.968035	-3.424617	-0.843887
H	-1.989659	0.160264	0.859686
H	-6.894382	-1.845745	-0.784696
H	-7.789843	0.406159	-0.326144
H	-3.886560	1.726456	0.898124
H	-6.277877	2.198785	0.512083
H	3.731783	0.116242	1.551426
H	5.799712	1.463704	1.681271
H	6.373227	3.038205	-0.149800
H	4.855471	3.247003	-2.101906
H	2.799410	1.867534	-2.229931

8b

E(ωB97X-D/6-311+G**)= -1751.497620

C	-3.636270	-1.618509	-1.802754
C	-3.302480	-0.872457	-0.664247
C	-4.169199	-0.932517	0.434288
C	-5.344600	-1.669437	0.382562
C	-5.674957	-2.382996	-0.764620
C	-4.809164	-2.358543	-1.854336
C	-2.063295	-0.103129	-0.604715
P	-0.387712	-0.457311	-1.280670
C	0.253615	-1.764182	-0.140565
C	1.371652	-2.507218	-0.529279
C	1.947837	-3.436995	0.328551
C	1.401259	-3.655387	1.588599
C	0.278457	-2.932862	1.982597
C	-0.286288	-1.995447	1.127713
C	-0.295057	1.160481	-0.249540
C	-1.757832	1.110310	-0.061985
Si	-2.738008	2.545844	0.657233
C	-2.396049	2.627423	2.505557
C	0.662240	1.971385	0.184515
C	2.043731	1.666277	-0.070478
C	2.836912	2.508876	-0.916865
C	4.147835	2.239115	-1.181374
C	4.799696	1.114029	-0.605161
C	4.038121	0.276476	0.259075
C	2.682849	0.574293	0.508701
C	6.153353	0.798608	-0.860663
C	6.743425	-0.308312	-0.300910
C	5.986864	-1.151123	0.544973
C	4.674109	-0.866233	0.817776
C	-2.154594	4.131491	-0.161495
C	-4.598614	2.390554	0.359900
H	1.813664	-2.331201	-1.505108
H	2.830818	-3.984036	0.014231
H	1.848119	-4.380768	2.260946
H	-0.153194	-3.093356	2.965654
H	-1.143173	-1.413635	1.451883
H	-2.660106	5.005834	0.263891

H	-1.075622	4.224582	-0.013634
H	-2.349108	4.109948	-1.238316
H	-2.885664	3.489662	2.971228
H	-2.741429	1.721573	3.014298
H	-1.315674	2.711530	2.655013
H	-5.076171	3.360947	0.537344
H	-4.807405	2.095069	-0.672764
H	-5.075509	1.656650	1.013454
H	2.358126	3.377917	-1.357354
H	4.716721	2.888966	-1.841952
H	2.116554	-0.081384	1.161744
H	6.725038	1.449262	-1.517719
H	7.783338	-0.539246	-0.507852
H	4.088060	-1.517984	1.459005
H	6.451430	-2.031211	0.979435
H	-3.898710	-0.404118	1.342105
H	-6.001240	-1.696014	1.246472
H	-6.591478	-2.962113	-0.804985
H	-5.049957	-2.920382	-2.751220
H	-2.960954	-1.602483	-2.651922

TS-4b

E(ωB97X-D/6-311+G**)= -1751.478115

C	0.100409	-1.145137	1.817749
C	0.516659	-1.532941	0.534641
C	1.733286	-2.232232	0.447860
C	2.501877	-2.505650	1.570203
C	2.072911	-2.107556	2.833305
C	0.861523	-1.429644	2.942752
P	-0.368992	-1.194578	-1.029182
C	-1.865573	-0.304712	-0.489946
C	-1.834304	1.022551	-0.119648
C	-0.521836	1.593331	-0.042675
C	0.577089	1.150528	-0.467352
C	2.011016	1.129517	-0.369808
C	2.645592	1.820158	0.705499
C	3.995195	1.750655	0.888883
C	4.812775	0.985999	0.015165
C	4.193057	0.308242	-1.068428
C	2.792638	0.406227	-1.240724
C	5.002410	-0.468549	-1.936985
C	6.354720	-0.567844	-1.741648
C	6.969436	0.108133	-0.663530
C	6.212520	0.866708	0.191454
C	-3.153677	-1.034691	-0.581707
C	-3.700515	-1.410204	-1.814330
C	-4.934345	-2.038667	-1.884729
C	-5.649358	-2.324454	-0.723042
C	-5.105370	-1.982181	0.508630
C	-3.870165	-1.347100	0.577324
Si	-3.298406	2.153959	0.218902
C	-3.771764	2.106613	2.046541
C	-2.769918	3.912978	-0.201955
C	-4.833815	1.768787	-0.807075
H	2.089252	-2.552971	-0.527706
H	3.447509	-3.027289	1.455047

H	2.671980	-2.316867	3.713522
H	0.509352	-1.103967	3.917844
H	-0.822236	-0.584711	1.926150
H	2.016696	2.382755	1.385457
H	4.459931	2.264176	1.725654
H	2.307187	-0.132348	-2.047671
H	6.678887	1.383081	1.025631
H	8.040255	0.019533	-0.512847
H	4.524960	-0.990607	-2.760497
H	6.958842	-1.171021	-2.411398
H	-3.567332	4.627901	0.029707
H	-1.872704	4.189753	0.357448
H	-2.530690	3.999043	-1.266421
H	-4.519506	2.872105	2.281864
H	-4.189149	1.132016	2.315267
H	-2.891436	2.283672	2.671805
H	-5.563502	2.579392	-0.695710
H	-4.579449	1.685590	-1.867912
H	-5.312737	0.832892	-0.509480
H	-3.148291	-1.188202	-2.721161
H	-5.347379	-2.303737	-2.852992
H	-6.615878	-2.813942	-0.780731
H	-5.643645	-2.208670	1.423781
H	-3.443201	-1.087528	1.539883

9b

E(ωB97X-D/6-311+G**)= -1751.515566

C	3.731677	0.900388	0.458303
C	3.000766	0.585819	-0.691767
C	3.480115	1.045704	-1.923340
C	4.668844	1.759563	-2.004393
C	5.396705	2.046107	-0.853381
C	4.918567	1.617128	0.379603
C	1.739369	-0.165595	-0.577266
C	1.531366	-1.414865	-0.080104
C	0.117987	-1.860095	0.074372
C	-0.721203	-0.859681	-0.336943
C	-2.185246	-0.911279	-0.258726
C	-2.807511	-2.068853	0.305209
C	-4.160028	-2.161205	0.439401
C	-5.015965	-1.103188	0.024703
C	-4.417577	0.056669	-0.537663
C	-3.008031	0.121046	-0.664748
C	-5.263673	1.118627	-0.950699
C	-6.625810	1.035399	-0.814386
C	-7.216679	-0.118735	-0.255115
C	-6.422865	-1.161520	0.153515
P	0.150178	0.568147	-1.083776
C	0.045910	1.903502	0.196160
C	-0.249380	1.616646	1.531636
C	-0.281466	2.628893	2.481251
C	-0.015972	3.945816	2.114295
C	0.278582	4.242431	0.789587
C	0.302351	3.227213	-0.162702
Si	2.788910	-2.721040	0.402129
C	2.635383	-3.056289	2.250857

C	2.384244	-4.298736	-0.543704
C	4.591664	-2.291578	0.017385
H	0.528267	3.461944	-1.198382
H	0.487250	5.265954	0.494330
H	-0.041464	4.735404	2.858462
H	-0.513517	2.390490	3.514419
H	-0.448417	0.586659	1.811696
H	-2.141349	-2.864564	0.620519
H	-4.606671	-3.053400	0.871183
H	-2.576444	1.020045	-1.096841
H	-6.869063	-2.052969	0.586100
H	-8.294961	-0.177880	-0.148707
H	-4.808761	2.007170	-1.378500
H	-7.255209	1.859248	-1.135897
H	3.006411	-5.137646	-0.211681
H	1.330584	-4.543403	-0.388641
H	2.543618	-4.157199	-1.617575
H	3.251903	-3.906330	2.563409
H	2.939602	-2.180384	2.833335
H	1.588962	-3.271630	2.484374
H	5.208270	-3.194649	0.095080
H	4.699440	-1.892826	-0.996213
H	4.996601	-1.543384	0.703223
H	2.913919	0.823727	-2.821664
H	5.030297	2.094846	-2.971519
H	6.325109	2.604009	-0.917634
H	5.468920	1.846345	1.286564
H	3.346556	0.576802	1.419353

1b' + PhTMSP$^-$

E(ωB97X-D/6-311+G**)= -1751.461884

C	-3.038274	1.871378	-1.965868
C	-2.167314	1.799153	-0.851966
C	-2.805678	1.616607	0.395697
C	-4.184331	1.517566	0.518289
C	-5.011915	1.594037	-0.598122
C	-4.415480	1.773650	-1.845486
P	-0.380401	1.874301	-1.179435
Si	0.533949	2.106081	0.821818
C	0.373419	0.714249	2.117727
C	2.404720	2.249525	0.525260
C	0.001735	3.680812	1.746953
C	0.555154	-1.721125	-0.458003
C	1.762777	-1.686331	-0.440994
C	3.115117	-1.508379	-0.386611
C	4.302332	-1.292180	-0.318913
C	5.680404	-0.938148	-0.221510
C	6.035304	0.393554	0.034943
C	7.372258	0.750693	0.134010
C	8.369918	-0.206892	-0.019445
C	8.024276	-1.529870	-0.274656
C	6.689392	-1.896314	-0.375720
C	-0.869404	-1.746068	-0.453275
C	-1.553576	-2.030183	0.705940
C	-2.968698	-2.042127	0.727118
C	-3.678768	-1.766895	-0.470568

C	-2.947304	-1.469602	-1.649703
C	-1.583995	-1.452675	-1.647087
C	-5.093733	-1.774590	-0.444248
C	-5.774212	-2.044035	0.713957
C	-5.067996	-2.309010	1.907458
C	-3.697656	-2.307665	1.912911
H	7.634618	1.784180	0.331484
H	9.413835	0.076375	0.060396
H	-2.599538	2.013055	-2.950314
H	-5.033642	1.838199	-2.738093
H	-6.088591	1.503699	-0.499195
H	-4.618651	1.357276	1.501592
H	-2.208785	1.535335	1.296370
H	6.414755	-2.925776	-0.574137
H	8.797684	-2.280730	-0.395154
H	5.251800	1.133860	0.152800
H	0.848841	1.023299	3.057215
H	-0.666370	0.454924	2.336966
H	0.874033	-0.191714	1.766482
H	0.501354	3.769218	2.719978
H	0.243802	4.563891	1.147786
H	-1.079828	3.687662	1.916062
H	2.940495	2.380660	1.473132
H	2.776280	1.340306	0.042187
H	2.635247	3.098213	-0.126020
H	-1.026044	-1.172549	-2.530625
H	-3.492193	-1.212358	-2.550794
H	-1.005755	-2.225770	1.621172
H	-5.629423	-1.539478	-1.357824
H	-6.859085	-2.035383	0.722285
H	-5.614364	-2.507931	2.823563
H	-3.150574	-2.502916	2.830203

TS-1b’

E(ωB97X-D/6-311+G**)= -1751.453910

C	-2.657155	2.013051	-2.042006
C	-1.900276	1.738973	-0.885962
C	-2.630879	1.519943	0.296118
C	-4.018816	1.556648	0.320274
C	-4.740136	1.819016	-0.838998
C	-4.043849	2.053574	-2.022122
P	-0.092710	1.532619	-1.087426
Si	0.709326	2.037317	0.937535
C	0.514379	0.756044	2.326416
C	2.570329	2.282566	0.729639
C	-0.020689	3.654427	1.603574
C	0.328578	-1.029351	-0.675083
C	1.518121	-1.370423	-0.580523
C	2.863843	-1.253853	-0.513865
C	4.075910	-1.169309	-0.413605
C	5.472374	-0.994596	-0.267590
C	6.006677	-0.496523	0.934822
C	7.373515	-0.319724	1.082719
C	8.245547	-0.628874	0.042288
C	7.728514	-1.118675	-1.153905
C	6.363161	-1.301886	-1.311376

C	-1.073079	-1.355811	-0.583469
C	-1.644001	-1.648034	0.633431
C	-3.034928	-1.883260	0.754026
C	-3.850123	-1.844002	-0.407269
C	-3.234270	-1.574458	-1.658132
C	-1.896922	-1.330335	-1.743804
C	-5.244650	-2.040644	-0.276134
C	-5.813550	-2.262984	0.951267
C	-5.003998	-2.301604	2.108411
C	-3.649309	-2.119046	2.009795
H	7.762621	0.070012	2.017847
H	9.313943	-0.486214	0.161557
H	-2.130782	2.188267	-2.975914
H	-4.586404	2.263169	-2.939894
H	-5.825030	1.833542	-0.821677
H	-4.540675	1.348618	1.249650
H	-2.106245	1.278850	1.213044
H	5.963736	-1.684137	-2.243648
H	8.397280	-1.361587	-1.973510
H	5.327938	-0.244180	1.741279
H	0.992779	1.126480	3.241240
H	-0.533282	0.544516	2.559391
H	0.994929	-0.184669	2.043658
H	0.424112	3.911620	2.572357
H	0.173665	4.473812	0.905385
H	-1.104247	3.579171	1.733130
H	3.041551	2.524222	1.689541
H	3.040595	1.375542	0.338261
H	2.779485	3.095693	0.028284
H	-1.439359	-1.069466	-2.690727
H	-3.855144	-1.524540	-2.547017
H	-1.024148	-1.660801	1.522982
H	-5.861822	-1.996921	-1.168607
H	-6.885579	-2.404192	1.039833
H	-5.460689	-2.472675	3.077761
H	-3.025042	-2.140949	2.898072

6b'

E(ωB97X-D/6-311+G**)= -1751.483770

C	-1.384665	5.782749	0.177012
C	-1.498865	4.421246	0.096947
C	-0.386862	3.613714	-0.268129
C	0.850483	4.252694	-0.554482
C	0.938521	5.664453	-0.461173
C	-0.149930	6.415079	-0.103604
C	-0.479759	2.207467	-0.345666
C	0.598302	1.419409	-0.697508
C	1.827427	2.077841	-0.987550
C	1.949303	3.438322	-0.919707
C	0.476174	-0.062198	-0.738178
C	-0.684030	-0.672561	-0.522366
C	-1.807549	-1.252399	-0.305438
C	-2.855775	-1.965091	-0.089040
C	-4.199443	-1.591307	0.232208
C	-5.199875	-1.500888	-0.759011
C	-6.508884	-1.180284	-0.433422

C	-6.885442	-0.951213	0.888631
C	-5.912161	-1.046902	1.881010
C	-4.598949	-1.361262	1.566788
P	1.970736	-1.051797	-1.194350
Si	1.312136	-3.183534	-0.727212
C	0.690709	-3.422160	1.027067
C	3.078003	-0.831257	0.277088
C	4.435848	-1.142624	0.158159
C	5.302041	-1.001061	1.236368
C	4.827770	-0.522362	2.452752
C	3.481952	-0.194974	2.580206
C	2.614970	-0.351959	1.505645
C	0.054378	-3.779664	-1.985153
C	2.895737	-4.199540	-0.928305
H	-6.178936	-0.869664	2.919304
H	-7.912509	-0.707954	1.138478
H	4.817267	-1.498474	-0.794660
H	6.351738	-1.252456	1.121360
H	5.503542	-0.399843	3.292825
H	3.101471	0.184305	3.523129
H	1.563947	-0.104649	1.613504
H	-4.927159	-1.685806	-1.792635
H	-7.249666	-1.110293	-1.225166
H	-3.853633	-1.433322	2.351825
H	0.594656	-4.493450	1.237804
H	1.389141	-2.993604	1.751878
H	-0.290156	-2.960084	1.157577
H	2.674748	-5.262433	-0.779907
H	3.328882	-4.080220	-1.926116
H	3.650642	-3.908183	-0.192124
H	-0.059653	-4.866668	-1.906041
H	-0.920668	-3.321833	-1.800063
H	0.369809	-3.544197	-3.006039
H	2.690579	1.485992	-1.270844
H	2.902138	3.910041	-1.142290
H	-1.426097	1.724408	-0.121711
H	1.889395	6.143246	-0.677402
H	-0.069301	7.494780	-0.033021
H	-2.243245	6.383854	0.458073
H	-2.444453	3.933316	0.311595

TS-2b’

E(ωB97X-D/6-311+G**)=-1751.483135

C	-4.334582	-2.117925	1.628703
C	-3.912183	-1.945761	0.292969
C	-4.879508	-2.112480	-0.720697
C	-6.192193	-2.429695	-0.409236
C	-6.594598	-2.594309	0.914313
C	-5.650366	-2.434641	1.925954
C	-2.578088	-1.617981	-0.011640
C	-1.422240	-1.280891	-0.267358
C	-0.098099	-1.105347	-0.535748
C	0.693589	-0.031132	-0.716439
P	2.401638	-0.509934	-1.210575
Si	2.040136	-2.730011	-0.774653
C	3.806912	-3.439154	-0.988991

C	0.297362	1.397678	-0.655720
C	1.260676	2.427498	-0.870896
C	0.932286	3.752440	-0.801007
C	-0.392586	4.160059	-0.508342
C	-1.368071	3.149989	-0.291844
C	-0.996398	1.788264	-0.368605
C	-0.776343	5.521925	-0.422439
C	-2.070041	5.871485	-0.137747
C	-3.044617	4.868134	0.074916
C	-2.701886	3.544797	0.000162
C	3.438861	-0.089141	0.263209
C	4.830879	-0.205156	0.172650
C	5.649042	0.086894	1.256869
C	5.092918	0.528079	2.453596
C	3.712655	0.663652	2.553624
C	2.894374	0.351742	1.473547
C	1.593987	-3.188158	0.999689
C	1.037283	-3.631059	-2.094747
H	-6.913764	-2.548933	-1.211885
H	-7.623688	-2.840496	1.151672
H	5.275352	-0.529429	-0.763835
H	6.725239	-0.021088	1.163295
H	5.730330	0.768509	3.298300
H	3.266260	1.010241	3.480320
H	1.817539	0.445808	1.567250
H	-3.607014	-1.998021	2.423409
H	-5.942674	-2.560828	2.964107
H	-4.578571	-1.984287	-1.754296
H	2.099363	-4.116595	1.291256
H	1.922613	-2.398915	1.683027
H	0.515969	-3.307078	1.116337
H	3.790633	-4.527723	-0.854571
H	4.214096	-3.232093	-1.984824
H	4.500999	-3.026503	-0.249076
H	1.382213	-4.666538	-2.200539
H	-0.024740	-3.622538	-1.845801
H	1.159343	-3.136235	-3.063597
H	2.284343	2.147735	-1.095313
H	1.691696	4.511236	-0.967342
H	-1.757433	1.035772	-0.196797
H	-0.023244	6.287466	-0.586835
H	-2.351168	6.917576	-0.073475
H	-4.068015	5.151756	0.297683
H	-3.446294	2.771521	0.162818

(Z)-7b'

E(ωB97X-D/6-311+G**)= -1751.520471

C	-1.057541	-5.156477	-0.192620
C	-0.571911	-4.313715	0.772172
C	-0.571213	-2.909703	0.579902
C	-1.079254	-2.379573	-0.635609
C	-1.580001	-3.277283	-1.613149
C	-1.570440	-4.630050	-1.400097
C	-0.076614	-2.006723	1.555597
C	-0.081322	-0.661104	1.326263
C	-0.592136	-0.118817	0.116020

C	-1.076786	-0.977507	-0.836251
C	-0.582473	1.360460	-0.086331
C	0.677275	1.985261	-0.081932
C	1.823446	1.170385	-0.090765
C	2.800520	0.444380	-0.093498
C	3.831837	-0.523475	-0.059911
C	3.504896	-1.893940	-0.049969
C	4.498391	-2.859242	-0.012197
C	5.843428	-2.495607	0.015492
C	6.179412	-1.144614	0.005214
C	5.192540	-0.170036	-0.031974
P	-2.082182	2.239064	-0.400229
C	-3.406658	1.032821	0.029434
C	-4.478676	0.874944	-0.859804
C	-5.540291	0.020794	-0.580560
C	-5.557895	-0.712445	0.600460
C	-4.510442	-0.560548	1.505101
C	-3.464509	0.310022	1.230918
Si	0.973275	3.830643	-0.072424
C	0.364529	4.695462	-1.635180
C	0.175944	4.650735	1.427793
C	2.834905	4.127105	0.039195
H	7.223267	-0.846543	0.026478
H	6.617835	-3.254577	0.043412
H	-4.463900	1.420838	-1.798915
H	-6.348392	-0.084369	-1.298686
H	-6.374897	-1.393788	0.814873
H	-4.510629	-1.120792	2.435247
H	-2.668517	0.426000	1.958053
H	2.458009	-2.177568	-0.070261
H	4.220022	-3.908488	-0.006160
H	5.458786	0.881186	-0.039668
H	-1.478518	-0.571777	-1.758792
H	0.319271	-2.403391	2.485816
H	0.312863	0.024954	2.067565
H	0.333543	5.735095	1.409439
H	-0.899059	4.456026	1.448403
H	0.612924	4.256575	2.350793
H	3.048921	5.201371	0.068554
H	3.253498	3.666159	0.938693
H	3.353082	3.694035	-0.821410
H	0.588600	5.767923	-1.598551
H	0.852877	4.272449	-2.518878
H	-0.714203	4.568740	-1.755620
H	-1.977010	-2.867589	-2.536655
H	-1.957089	-5.303787	-2.157640
H	-1.051139	-6.229738	-0.033001
H	-0.177029	-4.712965	1.701994

TS-3b'

E(ωB97X-D/6-311+G**)= -1751.490104

C	2.269357	0.189400	-2.562245
C	2.090001	-1.161387	-2.243571
C	3.151080	-2.038803	-2.479217
C	4.366715	-1.580480	-2.978921
C	4.535773	-0.232107	-3.267381

C	3.479110	0.650980	-3.062112
P	0.482835	-1.846127	-1.614351
C	0.083035	-0.917960	-0.265348
C	0.885722	0.183285	0.328694
C	2.212408	0.038069	0.653638
C	2.957722	1.100080	1.220411
C	2.320120	2.346584	1.455339
C	0.941890	2.471041	1.136429
C	0.243537	1.423174	0.608910
C	3.075937	3.412074	2.003004
C	4.404391	3.252986	2.300906
C	5.040374	2.013435	2.065171
C	4.332847	0.964633	1.539490
C	-1.208723	-1.240248	0.405918
Si	-1.235137	-2.455804	1.775491
C	-0.935477	-4.236888	1.200227
C	-2.329248	-0.523299	0.070230
C	-3.327307	0.140207	-0.218642
C	-4.471213	0.858718	-0.583582
C	-4.438056	1.832227	-1.612684
C	-5.570550	2.546326	-1.965443
C	-6.788423	2.332008	-1.320362
C	-6.841683	1.376706	-0.305648
C	-5.717181	0.655619	0.059649
C	0.126610	-2.106190	3.047626
C	-2.910481	-2.400153	2.638853
H	-2.970281	-3.150798	3.434545
H	-3.499382	2.007998	-2.126899
H	-7.779710	1.190780	0.210351
H	-7.672952	2.893030	-1.601805
H	1.458039	0.889537	-2.392386
H	3.602117	1.705851	-3.284373
H	5.482438	0.130736	-3.654015
H	5.181314	-2.279254	-3.141784
H	3.027450	-3.095639	-2.258557
H	-5.504043	3.284674	-2.759810
H	-5.775254	-0.083478	0.851326
H	-0.814791	1.516829	0.392169
H	0.442713	3.416320	1.327795
H	2.711507	-0.906486	0.465571
H	-0.922475	-4.940222	2.041662
H	-1.715356	-4.548695	0.498708
H	0.023955	-4.314703	0.677978
H	0.135905	-2.856571	3.846695
H	1.111330	-2.114335	2.568207
H	-0.006705	-1.118388	3.499232
H	-3.087593	-1.413029	3.075430
H	-3.713987	-2.590127	1.921179
H	4.817789	0.011816	1.351240
H	6.093230	1.895837	2.299259
H	2.582902	4.363044	2.181973
H	4.971514	4.078224	2.718654

(E)-7b’

E(ωB97X-D/6-311+G**)= -1751.518861

C	-6.140807	-1.576552	-0.216331

C	-5.215860	-1.697071	0.785876
C	-3.887536	-1.232169	0.611485
C	-3.521554	-0.637118	-0.625828
C	-4.504007	-0.523956	-1.644037
C	-5.779257	-0.980080	-1.447106
C	-2.906078	-1.331844	1.628530
C	-1.635019	-0.873733	1.418410
C	-1.254911	-0.290019	0.181990
C	-2.197682	-0.175691	-0.808831
C	0.142664	0.187928	0.000879
C	1.151297	-0.792335	0.011599
C	2.496181	-0.385554	-0.008629
C	3.671330	-0.070328	-0.011467
C	5.026638	0.339400	-0.029614
C	5.368143	1.699887	0.083772
C	6.695192	2.101072	0.066725
C	7.719992	1.166056	-0.061495
C	7.395546	-0.182914	-0.175694
C	6.070568	-0.593856	-0.161421
P	0.478865	1.902537	-0.241377
C	-1.175454	2.683881	0.043343
C	-1.720738	3.517681	-0.941476
C	-2.939274	4.164983	-0.758914
C	-3.654847	3.992014	0.420130
C	-3.123420	3.183040	1.420674
C	-1.897288	2.557870	1.239629
Si	0.926643	-2.645440	-0.062152
C	0.802048	-3.457503	1.645407
C	-0.571939	-3.219673	-1.059705
C	2.455439	-3.390736	-0.884209
H	8.182776	-0.923623	-0.277445
H	8.756926	1.484685	-0.072491
H	-1.184341	3.641397	-1.878017
H	-3.337116	4.796147	-1.548402
H	-4.613358	4.481496	0.559564
H	-3.665002	3.042733	2.351339
H	-1.490976	1.945016	2.036954
H	4.570009	2.426581	0.183672
H	6.932570	3.156534	0.156646
H	5.822537	-1.645263	-0.255338
H	-1.920350	0.286475	-1.750289
H	-3.179614	-1.773936	2.582232
H	-0.887564	-0.939546	2.201339
H	-0.490993	-4.297350	-1.244226
H	-1.516178	-3.034746	-0.541798
H	-0.617040	-2.710293	-2.026615
H	2.346237	-4.476020	-0.988125
H	2.610687	-2.960177	-1.877927
H	3.354338	-3.186254	-0.296193
H	0.900347	-4.546417	1.565536
H	1.597705	-3.091399	2.301883
H	-0.158381	-3.240390	2.121241
H	-4.223541	-0.060315	-2.584498
H	-6.519947	-0.883972	-2.234065
H	-7.153763	-1.936759	-0.069931
H	-5.488153	-2.152571	1.733584

TS-5b'

E(ωB97X-D/6-311+G**)= -1751.485427

C	-4.786714	-1.291226	-1.691419
C	-3.978186	-0.544591	-0.794251
C	-4.591661	0.044623	0.344100
C	-5.983416	-0.128700	0.545492
C	-6.737747	-0.852947	-0.339285
C	-6.129565	-1.442270	-1.471754
C	-3.775608	0.773268	1.246447
C	-2.436385	0.913234	1.020501
C	-1.809606	0.352086	-0.129264
C	-2.588966	-0.376301	-1.001236
C	-0.362094	0.481070	-0.315335
C	0.444044	1.541446	-0.002247
Si	0.109167	3.372423	0.280572
C	0.355332	3.779430	2.103932
P	0.776135	-0.693791	-1.107287
C	0.643240	-2.190340	-0.054762
C	0.027557	-2.220706	1.205569
C	-0.010464	-3.385295	1.962862
C	0.586254	-4.552503	1.497085
C	1.220655	-4.537880	0.256918
C	1.240811	-3.379122	-0.506099
C	1.808617	1.079591	-0.105989
C	3.032951	1.274523	0.189301
C	4.208197	0.490010	-0.046686
C	5.372379	1.041116	-0.613955
C	6.491550	0.260146	-0.859335
C	6.499640	-1.093763	-0.526013
C	5.361539	-1.652688	0.048748
C	4.232157	-0.880601	0.284436
C	1.342997	4.363664	-0.732924
C	-1.626313	3.880059	-0.261496
H	5.346990	-2.707121	0.309167
H	7.380364	-1.700526	-0.710166
H	1.733024	-3.384589	-1.474809
H	1.696252	-5.438321	-0.120613
H	0.562707	-5.459324	2.092686
H	-0.501626	-3.376939	2.931411
H	-0.418614	-1.314761	1.601502
H	1.217631	5.439224	-0.565053
H	2.359552	4.069998	-0.459124
H	1.214684	4.164138	-1.801144
H	0.249136	4.853077	2.293149
H	-0.365446	3.250455	2.735384
H	1.359707	3.469792	2.407753
H	-1.684853	4.971719	-0.336569
H	-1.857302	3.460365	-1.245296
H	-2.403489	3.544274	0.428148
H	5.377020	2.097417	-0.862854
H	7.371170	0.711614	-1.309500
H	3.346143	-1.326012	0.722539
H	-1.816318	1.441373	1.737047
H	-4.229927	1.206953	2.132617
H	-2.119250	-0.830822	-1.868144

H	-6.444231	0.323329	1.418983
H	-7.802313	-0.979803	-0.173572
H	-6.732822	-2.019113	-2.164827
H	-4.315732	-1.745446	-2.557448

8b’

E(ωB97X-D/6-311+G**)= -1751.492534

C	-6.170962	-1.756932	-1.688107
C	-4.829858	-1.543569	-1.863415
C	-4.080025	-0.787527	-0.923578
C	-4.752513	-0.255620	0.210105
C	-6.139996	-0.494479	0.365714
C	-6.837038	-1.225569	-0.559764
C	-2.695344	-0.547740	-1.086048
C	-1.976460	0.201504	-0.177891
C	-2.661599	0.698892	0.968609
C	-3.997098	0.487400	1.152989
C	-0.546253	0.430034	-0.331466
C	0.260782	1.484124	-0.008470
Si	-0.011429	3.276780	0.499752
C	-1.801578	3.837699	0.255375
P	0.773972	-0.618241	-1.068405
C	1.597141	0.939087	-0.305074
C	2.829856	1.372024	-0.062614
C	3.977811	0.566338	-0.402138
C	4.865144	0.947976	-1.431306
C	5.976184	0.183342	-1.752002
C	6.270206	-0.982641	-1.045386
C	5.416152	-1.369370	-0.014932
C	4.293475	-0.617264	0.300904
C	1.012980	-1.933149	0.211702
C	1.575358	-3.153124	-0.168531
C	1.844028	-4.142619	0.771647
C	1.539638	-3.931214	2.110911
C	0.967787	-2.722897	2.502329
C	0.711309	-1.734668	1.562195
C	0.470039	3.472005	2.308568
C	1.080855	4.366982	-0.567851
H	5.618304	-2.279944	0.542841
H	7.145680	-1.574407	-1.292650
H	1.820825	-3.319862	-1.212895
H	2.294782	-5.078541	0.456823
H	1.748366	-4.700402	2.847573
H	0.733477	-2.547388	3.547650
H	0.292164	-0.783145	1.874496
H	0.972047	5.424007	-0.299595
H	2.119530	4.053091	-0.434803
H	0.828501	4.250903	-1.626452
H	0.374587	4.510712	2.643283
H	-0.152543	2.846320	2.956515
H	1.510458	3.156300	2.428158
H	-1.840431	4.932905	0.245227
H	-2.197180	3.477800	-0.699369
H	-2.474594	3.486267	1.040848
H	4.658530	1.863981	-1.976208
H	6.628484	0.503087	-2.560595

H	3.631298	-0.941936	1.096742
H	-2.088552	1.239863	1.713289
H	-4.495795	0.876430	2.035980
H	-2.187979	-0.956539	-1.954720
H	-6.644450	-0.084704	1.235924
H	-7.899708	-1.400255	-0.429574
H	-6.728638	-2.337421	-2.415557
H	-4.316846	-1.952589	-2.728258

TS-4b’

E(ωB97X-D/6-311+G**)= -1751.477753

C	2.786768	-2.692562	-0.112511
C	1.789767	-1.742121	0.182284
C	1.581882	-1.451638	1.542392
C	2.316750	-2.078148	2.539774
C	3.298401	-3.013817	2.225249
C	3.527967	-3.309844	0.883421
P	0.895914	-1.003994	-1.228298
C	-0.349893	0.039181	-0.383246
C	-0.051910	1.285376	0.109487
C	1.325479	1.690978	-0.036305
C	2.269163	1.116705	-0.632374
C	3.676322	0.969569	-0.893164
C	4.596464	1.746751	-0.170867
C	5.959233	1.618289	-0.390603
C	6.439318	0.708938	-1.329877
C	5.536326	-0.065197	-2.049760
C	4.170217	0.065533	-1.839271
C	-1.739405	-0.471491	-0.349882
C	-2.055418	-1.626383	0.421348
C	-3.340996	-2.068046	0.535040
C	-4.403941	-1.407081	-0.139475
C	-4.094937	-0.282518	-0.949609
C	-2.749884	0.158414	-1.035221
C	-5.149133	0.377500	-1.630040
C	-6.445072	-0.052999	-1.511000
C	-6.751134	-1.171827	-0.705445
C	-5.750775	-1.830971	-0.037772
Si	-1.210767	2.420050	1.078405
C	-2.396111	1.472941	2.200677
C	-0.141506	3.507365	2.184502
C	-2.215506	3.565686	-0.039217
H	5.896946	-0.780601	-2.781933
H	7.506614	0.602487	-1.494438
H	2.976067	-2.949648	-1.151401
H	4.291688	-4.032347	0.609576
H	3.874731	-3.497545	3.007028
H	2.126356	-1.823608	3.578909
H	0.844026	-0.704790	1.816716
H	4.215302	2.443982	0.566296
H	6.652930	2.225266	0.182868
H	3.456384	-0.533240	-2.392785
H	-2.664303	4.377201	0.544239
H	-1.576357	4.013174	-0.806541
H	-3.023410	3.024728	-0.539384
H	-0.761276	4.161413	2.808566

H	0.484734	2.893045	2.838232
H	0.529795	4.124426	1.581511
H	-2.844963	2.159837	2.927170
H	-3.202963	0.986997	1.647184
H	-1.861629	0.696190	2.756277
H	-1.245417	-2.140402	0.927740
H	-3.568964	-2.939455	1.141879
H	-2.514047	1.017163	-1.655042
H	-5.980837	-2.692399	0.582588
H	-7.779221	-1.506683	-0.615848
H	-7.241208	0.464965	-2.035597
H	-4.909561	1.237555	-2.248204

9b'

E(ωB97X-D/6-311+G**)= -1751.512913

C	-5.844185	-1.309526	-1.900097
C	-4.520796	-1.014555	-2.090676
C	-3.724135	-0.507701	-1.030225
C	-4.328538	-0.311133	0.240688
C	-5.699654	-0.627254	0.408296
C	-6.443184	-1.113911	-0.634376
C	-2.354805	-0.195022	-1.203869
C	-1.590252	0.308604	-0.174180
C	-2.207976	0.482438	1.096803
C	-3.526145	0.188895	1.297601
C	-0.155289	0.583953	-0.320167
P	0.979918	-0.669808	-0.991542
C	0.805597	-1.958654	0.324178
C	1.597547	-1.960848	1.475058
C	1.417121	-2.930446	2.454694
C	0.436306	-3.906113	2.307214
C	-0.360747	-3.910362	1.168018
C	-0.167812	-2.950575	0.180606
C	0.553916	1.697314	0.019460
Si	-0.080333	3.401098	0.486463
C	0.800754	4.669687	-0.589169
C	2.037400	1.615061	-0.068202
C	2.401631	0.367374	-0.499947
C	3.788483	-0.094630	-0.675345
C	4.862377	0.729349	-0.295289
C	6.175099	0.307948	-0.427471
C	6.471136	-0.955507	-0.940639
C	5.423114	-1.786399	-1.316264
C	4.104600	-1.362936	-1.184163
C	-1.937882	3.659359	0.220342
C	0.321567	3.745526	2.296813
H	5.628596	-2.774397	-1.717858
H	7.500653	-1.283600	-1.044300
H	-0.787972	-2.962598	-0.710636
H	-1.131414	-4.664689	1.044589
H	0.295378	-4.659915	3.075355
H	2.043773	-2.919809	3.341020
H	2.349975	-1.188820	1.596373
H	4.615804	1.707614	0.102606
H	6.981473	0.971355	-0.126914
H	3.304637	-2.031605	-1.488810

H	0.531596	5.694005	-0.306742
H	1.879559	4.531492	-0.487196
H	0.544701	4.524512	-1.643660
H	0.103174	4.784257	2.568111
H	-0.253969	3.091262	2.959866
H	1.384446	3.553492	2.468692
H	-2.164714	4.731490	0.244578
H	-2.251712	3.269486	-0.753229
H	-2.550087	3.167295	0.979609
H	-1.592636	0.841136	1.914739
H	-3.974629	0.325472	2.277264
H	-1.898705	-0.350083	-2.176655
H	-6.154186	-0.475657	1.382978
H	-7.492722	-1.349196	-0.492838
H	-6.438915	-1.695679	-2.721330
H	-4.057711	-1.164679	-3.061101

1c +PhTMSP$^-$

E(ωB97X-D/6-311+G**)= -2072.235949

C	-4.964076	1.169452	0.729784
C	-4.657434	1.390753	-0.632609
C	-5.664674	0.990630	-1.546211
C	-6.860551	0.422621	-1.138976
C	-7.128649	0.212931	0.215581
C	-6.162370	0.598674	1.140105
P	-3.125731	2.081679	-1.334831
Si	-1.859242	2.519869	0.426106
C	-0.233490	3.215798	-0.261308
C	-2.508687	3.845690	1.628397
C	-1.349709	1.041329	1.510371
C	-0.548756	-2.299639	-0.099661
C	-1.939206	-2.510044	-0.084891
C	-2.924126	-1.607554	-0.396282
C	-4.226920	-2.150137	-0.244175
C	-4.213873	-3.446608	0.174194
S	-2.614928	-4.045449	0.399305
C	0.642685	-2.087069	-0.102231
C	1.975966	-1.801518	-0.092381
C	3.152298	-1.521586	-0.077402
C	4.528278	-1.156347	-0.056790
C	5.532369	-2.157139	0.083924
C	6.853953	-1.817720	0.107272
C	7.263318	-0.463567	-0.007627
C	6.267746	0.539779	-0.148247
C	4.903807	0.165205	-0.169396
C	6.671233	1.895857	-0.262673
C	7.996737	2.235122	-0.238956
C	8.986874	1.235888	-0.099005
C	8.627432	-0.080611	0.013642
H	-5.481207	1.136356	-2.607153
H	-7.594491	0.133585	-1.886826
H	-8.063124	-0.234421	0.537790
H	-6.338107	0.446034	2.201698
H	-4.245454	1.444692	1.492792
H	-2.724609	-0.587945	-0.722981
H	-5.130366	-1.583231	-0.428753

H	-5.059914	-4.088208	0.373083
H	5.225545	-3.192569	0.173209
H	7.612055	-2.587068	0.214666
H	4.142227	0.930056	-0.274395
H	9.385291	-0.850461	0.120771
H	10.034151	1.517016	-0.081167
H	5.908062	2.659747	-0.369168
H	8.292245	3.274582	-0.328564
H	-0.773850	1.374027	2.382897
H	-2.207955	0.466936	1.871411
H	-0.721094	0.356076	0.933682
H	-1.787857	4.035685	2.433762
H	-2.677068	4.782221	1.087529
H	-3.460049	3.558214	2.085970
H	0.467441	3.441277	0.551137
H	0.241390	2.494107	-0.933174
H	-0.410121	4.135994	-0.826801

TS-1c

E(ωB97X-D/6-311+G**)= -2072.223267

C	-4.460181	1.599498	0.105889
C	-3.855362	0.814572	-0.894682
C	-4.716589	-0.051740	-1.600995
C	-6.070918	-0.135768	-1.322745
C	-6.641409	0.654575	-0.326749
C	-5.820734	1.526799	0.379162
P	-2.082769	0.720774	-1.309010
Si	-1.128286	2.293238	-0.044078
C	0.701029	2.310452	-0.508704
C	-1.775525	4.047554	-0.374129
C	-1.200061	2.028885	1.835520
C	-1.255280	-1.253485	0.323005
C	-2.563883	-1.669603	0.706846
C	-3.411791	-1.114288	1.626634
C	-4.652904	-1.796933	1.727576
C	-4.739763	-2.864857	0.887870
S	-3.306569	-3.051020	-0.050175
C	-0.026489	-1.412518	0.360104
C	1.300813	-1.273093	0.173069
C	2.507768	-1.155869	0.037157
C	3.896494	-0.984182	-0.145010
C	4.577998	-1.724130	-1.161843
C	5.918542	-1.577311	-1.360603
C	6.686593	-0.684244	-0.566542
C	6.021233	0.059850	0.446138
C	4.631518	-0.107756	0.635815
C	6.785758	0.956947	1.239269
C	8.132305	1.103653	1.038673
C	8.790695	0.361408	0.032196
C	8.078849	-0.511102	-0.749925
H	-4.293220	-0.680093	-2.378846
H	-6.689278	-0.829903	-1.884718
H	-7.701935	0.589745	-0.106355
H	-6.239916	2.153340	1.161776
H	-3.856711	2.280484	0.694015
H	-3.152919	-0.223982	2.181557

H	-5.458747	-1.483950	2.378283
H	-5.574965	-3.533723	0.744130
H	4.000375	-2.405547	-1.775515
H	6.417685	-2.146953	-2.138864
H	4.128598	0.466049	1.406460
H	8.577335	-1.083729	-1.526609
H	9.857447	0.485892	-0.118948
H	6.279821	1.528607	2.010953
H	8.699866	1.794255	1.653459
H	-0.628124	2.808415	2.353181
H	-2.222697	2.052583	2.224305
H	-0.765371	1.057934	2.089987
H	-1.241123	4.779396	0.243983
H	-1.617888	4.307231	-1.425355
H	-2.845259	4.145464	-0.169591
H	1.234680	3.079563	0.061877
H	1.176032	1.346666	-0.308348
H	0.826720	2.527747	-1.573751

6c

E(ωB97X-D/6-311+G**)= -2072.258930

C	-7.414418	0.280113	2.156103
C	-6.067627	0.034359	2.095753
C	-5.406128	-0.156685	0.850683
C	-6.189459	-0.082324	-0.336857
C	-7.576126	0.170776	-0.242716
C	-8.186193	0.349634	0.974236
C	-4.021137	-0.408476	0.765694
C	-3.378974	-0.592587	-0.456189
C	-4.189160	-0.523336	-1.640362
C	-5.526363	-0.269787	-1.580347
C	-2.002854	-0.917998	-0.569175
C	-0.901886	-0.267078	-0.634894
C	0.268470	0.257893	-0.697173
C	1.495073	0.758333	-0.766000
C	1.719571	2.213865	-0.706147
S	3.276441	2.931622	-1.025769
C	2.676963	4.524073	-0.735975
C	1.352862	4.502988	-0.414979
C	0.808989	3.190751	-0.393349
P	2.982902	-0.315317	-0.983716
C	3.710948	-0.355763	0.718505
C	5.003739	-0.861971	0.884309
C	5.582367	-0.950926	2.145005
C	4.885037	-0.510445	3.264650
C	3.606180	0.014168	3.109812
C	3.021552	0.085113	1.851173
Si	2.086479	-2.411830	-0.967565
C	1.164968	-2.840283	0.608335
C	1.009499	-2.654230	-2.485605
C	3.591703	-3.550361	-1.093773
H	5.562457	-1.187729	0.011648
H	6.583446	-1.356777	2.251900
H	5.337269	-0.571013	4.249059
H	3.054922	0.364906	3.976315
H	2.017932	0.481642	1.738703

H	-0.220578	2.951540	-0.161264
H	0.780552	5.396377	-0.196349
H	3.335270	5.376017	-0.819329
H	-3.704139	-0.675374	-2.598706
H	-6.107630	-0.211332	-2.497028
H	-3.441770	-0.465524	1.681728
H	-8.157077	0.223600	-1.159903
H	-9.251858	0.543933	1.033042
H	-5.480433	-0.017936	3.007950
H	-7.894986	0.422701	3.119101
H	0.998682	-3.922672	0.653640
H	1.742873	-2.548880	1.490396
H	0.192858	-2.342684	0.638925
H	3.262583	-4.593126	-1.164263
H	4.195513	-3.325230	-1.977995
H	4.231501	-3.460571	-0.210824
H	0.810113	-3.720901	-2.636690
H	0.052593	-2.142487	-2.354827
H	1.497054	-2.267947	-3.385593

TS-2c

E(ωB97X-D/6-311+G**)= -2072.258918

C	-7.566968	-0.308487	-0.130933
C	-6.158059	-0.340954	-0.250238
C	-5.357202	-0.286734	0.924345
C	-6.013525	-0.201422	2.182161
C	-7.380232	-0.171185	2.266936
C	-8.171854	-0.224700	1.097206
C	-3.949930	-0.313910	0.814215
C	-3.316811	-0.391269	-0.418272
C	-4.138839	-0.450031	-1.591041
C	-5.498870	-0.424997	-1.505991
C	-1.915933	-0.409649	-0.545293
C	-0.697136	-0.318851	-0.665007
C	0.664644	-0.430035	-0.768073
C	1.633896	0.498988	-0.870495
P	3.288990	-0.263965	-1.106405
Si	2.396951	-2.351853	-0.714138
C	4.012067	-3.394316	-0.706498
C	1.495800	1.956311	-0.894142
S	-0.053173	2.744221	-0.727753
C	0.663943	4.310710	-0.821986
C	2.016333	4.236820	-0.970117
C	2.489546	2.897146	-1.008162
C	4.132801	0.014506	0.515344
C	5.473559	-0.362138	0.656252
C	6.142675	-0.201019	1.863331
C	5.490875	0.366316	2.953652
C	4.163731	0.761341	2.823332
C	3.490358	0.582005	1.620776
C	1.681248	-2.689307	1.000869
C	1.426176	-3.097886	-2.153961
H	-6.099159	-0.467518	-2.410492
H	5.995472	-0.789767	-0.194963
H	7.179531	-0.510952	1.950967
H	6.012766	0.501663	3.895400

H	3.644922	1.207015	3.666237
H	2.450324	0.877872	1.534246
H	-3.349046	-0.268852	1.716077
H	-3.648501	-0.512731	-2.555729
H	1.999812	-3.672659	1.366536
H	2.042244	-1.936074	1.708380
H	0.591102	-2.638572	0.987300
H	3.778186	-4.454588	-0.548522
H	4.552944	-3.310243	-1.656076
H	4.691657	-3.084508	0.095067
H	1.664417	-4.160289	-2.282764
H	0.353295	-2.979808	-1.991580
H	1.677965	-2.579843	-3.084836
H	-5.407550	-0.159413	3.082115
H	-7.861469	-0.103725	3.237203
H	-9.253431	-0.198684	1.175610
H	-8.166439	-0.349653	-1.036127
H	0.046553	5.195147	-0.763372
H	2.657462	5.106511	-1.047087
H	3.531788	2.624046	-1.114794

(Z)-7c

E(ωB97X-D/6-311+G**)= -2072.292990

C	-6.788247	-1.975625	-0.043051
C	-5.523702	-1.342280	-0.069241
C	-5.451382	0.071275	0.073648
C	-6.662331	0.795069	0.241730
C	-7.872610	0.154208	0.264518
C	-7.940700	-1.249833	0.119568
C	-4.192118	0.711820	0.045523
C	-3.018427	-0.004848	-0.115501
C	-3.106208	-1.428175	-0.255017
C	-4.310727	-2.065354	-0.232704
C	-1.743769	0.599998	-0.143700
C	-0.589488	0.987192	-0.132673
C	0.733449	1.456777	-0.115670
C	1.794511	0.539914	-0.218800
P	3.432419	1.059312	-0.638648
C	4.500213	-0.312127	-0.040284
C	5.613059	-0.683969	-0.808598
C	6.505993	-1.657030	-0.375637
C	6.308455	-2.299076	0.842465
C	5.215206	-1.938632	1.625305
C	4.337030	-0.951845	1.198307
Si	0.937888	3.309426	0.082226
C	1.994524	3.718919	1.589200
C	1.662721	4.167417	-1.433480
C	-0.774176	4.054864	0.367284
C	1.459559	-0.894317	-0.066520
S	1.861263	-2.050929	-1.294299
C	1.144556	-3.340024	-0.407797
C	0.627757	-2.903775	0.777098
C	0.801006	-1.505945	0.964006
H	3.506383	-0.669844	1.834972
H	5.050676	-2.423409	2.583048
H	6.998344	-3.066324	1.178919

H	7.353543	-1.925136	-0.999868
H	5.766593	-0.207742	-1.772857
H	0.445342	-0.954343	1.824083
H	0.137945	-3.554286	1.491195
H	1.149034	-4.344617	-0.804454
H	1.653008	5.255685	-1.300165
H	2.691960	3.845391	-1.608666
H	1.077692	3.926819	-2.326873
H	-0.695581	5.136240	0.525441
H	-1.429158	3.877883	-0.490818
H	-1.255933	3.610952	1.243439
H	2.134078	4.801406	1.685927
H	1.512674	3.352728	2.501171
H	2.976808	3.247926	1.506320
H	-2.181828	-1.982958	-0.376759
H	-4.356893	-3.145082	-0.342358
H	-4.143636	1.790188	0.155254
H	-6.832321	-3.055188	-0.155828
H	-8.903776	-1.748531	0.138032
H	-6.609975	1.873772	0.353894
H	-8.786843	0.724115	0.394412

TS-3c

E(ωB97X-D/6-311+G**)= -2072.265709

C	6.805000	-2.901302	0.082554
C	5.464490	-2.618085	0.070682
C	4.991352	-1.283171	-0.075169
C	5.960553	-0.246182	-0.209047
C	7.334622	-0.569715	-0.193374
C	7.760013	-1.868313	-0.050952
C	3.618434	-0.970473	-0.089471
C	3.159046	0.340747	-0.233485
C	4.155525	1.370584	-0.366902
C	5.487144	1.087690	-0.354691
C	1.806053	0.678444	-0.251146
C	0.612414	0.985847	-0.275121
C	-0.706942	1.356862	-0.286324
C	-1.763618	0.305756	-0.152786
C	-2.172474	-0.022599	1.212926
C	-3.181605	-0.795876	1.744674
C	-3.196345	-0.806711	3.160808
C	-2.199159	-0.043622	3.699890
S	-1.236917	0.707820	2.494748
Si	-1.170634	3.110174	-0.573712
C	0.159406	4.249029	0.128955
C	-1.373221	3.552938	-2.404599
C	-2.828001	3.486617	0.257187
P	-2.302224	-0.293149	-1.641199
C	-3.578021	-1.589626	-1.269151
C	-3.198004	-2.899319	-0.959347
C	-4.150648	-3.882522	-0.721319
C	-5.505973	-3.576866	-0.800325
C	-5.899439	-2.284034	-1.128789
C	-4.942099	-1.303504	-1.368169
H	-3.601980	2.805359	-0.111901
H	2.893752	-1.771301	0.013082

H	6.213255	1.890048	-0.457499
H	-2.142383	-3.139927	-0.879611
H	-3.835404	-4.889989	-0.469286
H	-6.250756	-4.342917	-0.611386
H	-6.954463	-2.038622	-1.198046
H	-5.257431	-0.294133	-1.615796
H	3.813970	2.393973	-0.479310
H	-0.069920	5.303837	-0.058451
H	0.262876	4.099362	1.207726
H	1.127231	4.021132	-0.328014
H	-1.661604	4.601704	-2.545235
H	-0.433241	3.379297	-2.938057
H	-2.136068	2.919482	-2.868420
H	-2.761373	3.353558	1.341059
H	-3.154299	4.512292	0.052679
H	-3.898889	-1.333622	1.145169
H	-3.918346	-1.358783	3.750045
H	-1.985499	0.115774	4.747181
H	8.059210	0.234024	-0.296396
H	8.819320	-2.101969	-0.039930
H	4.735087	-3.416228	0.173141
H	7.138849	-3.928221	0.196192

(E)-7c

E(ωB97X-D/6-311+G**)= -2072.292717

C	-3.373215	-3.263030	-1.010579
C	-2.850697	-2.347233	-0.087031
C	-3.542192	-2.198948	1.124867
C	-4.718989	-2.890440	1.378788
C	-5.228031	-3.784236	0.440511
C	-4.540669	-3.973695	-0.753381
P	-1.240989	-1.524305	-0.459134
C	-1.554091	0.174221	-0.096319
C	-2.928443	0.685327	0.094774
C	-3.452165	1.286201	1.205492
C	-4.814974	1.666487	1.057334
C	-5.312282	1.352921	-0.172711
S	-4.127798	0.576340	-1.151655
C	-0.518001	1.125673	-0.019381
Si	-0.686445	2.988064	-0.041606
C	0.919821	3.703921	-0.732026
C	0.807440	0.667585	-0.011964
C	1.965320	0.292530	0.017604
C	3.269097	-0.249845	0.037993
C	4.408475	0.533524	-0.032729
C	5.701053	-0.037355	0.000402
C	5.841752	-1.448749	0.107164
C	4.662705	-2.240766	0.170427
C	3.425946	-1.669507	0.138160
C	6.878237	0.755234	-0.066582
C	8.121306	0.180855	-0.027018
C	8.257317	-1.221281	0.082012
C	7.138512	-2.012525	0.146429
C	-2.090492	3.635272	-1.124304
C	-0.894705	3.753641	1.678096

H	-3.150546	-1.522254	1.875798
H	-5.237500	-2.738197	2.320791
H	-6.146715	-4.326986	0.639363
H	-4.921457	-4.669515	-1.495457
H	-2.859626	-3.402481	-1.957517
H	-2.869856	1.425317	2.106563
H	-5.398509	2.146876	1.832903
H	-6.307989	1.525817	-0.553425
H	-0.752262	4.839751	1.634318
H	-1.889439	3.561250	2.088659
H	-0.154444	3.342723	2.371850
H	0.851870	4.795068	-0.806540
H	1.769248	3.451900	-0.091111
H	1.130298	3.300462	-1.726884
H	-1.971443	4.715877	-1.265817
H	-2.076441	3.159405	-2.108819
H	-3.072453	3.456433	-0.678765
H	2.527128	-2.274043	0.186701
H	4.761829	-3.319568	0.248422
H	4.307373	1.610577	-0.116048
H	7.235120	-3.091193	0.230781
H	9.245334	-1.667861	0.114174
H	6.773539	1.832637	-0.151387
H	9.008562	0.803584	-0.078772

TS-5c

E(ωB97X-D/6-311+G**)= -2072.261908

C	1.214838	2.530148	1.014193
C	0.541184	2.305890	-0.196095
C	-0.333539	3.310499	-0.642305
C	-0.530070	4.476798	0.082474
C	0.156583	4.686413	1.276220
C	1.032541	3.706113	1.732043
P	0.638258	0.757501	-1.171505
C	2.020453	-0.144643	-0.414823
C	1.450809	-1.302756	0.037756
C	0.021851	-1.089893	0.051222
C	-1.114827	-1.448512	0.496277
C	-2.427296	-0.901344	0.375735
C	-3.528154	-1.645737	-0.021003
C	-4.807195	-1.066029	-0.164363
C	-4.989917	0.316696	0.124308
C	-3.860794	1.068266	0.543832
C	-2.632276	0.488032	0.667094
C	-6.276154	0.889191	-0.017409
C	-7.348011	0.137113	-0.426179
C	-7.172144	-1.236764	-0.711011
C	-5.938732	-1.818693	-0.583709
C	3.424722	0.220452	-0.359428
C	4.326981	-0.010545	0.647983
C	5.617672	0.524444	0.389404
C	5.684795	1.170448	-0.809532
S	4.172054	1.134233	-1.634718
Si	2.179666	-2.998390	0.410605
C	0.975043	-4.307379	-0.194214
C	3.826220	-3.262953	-0.469152

C	2.410151	-3.204517	2.270271
H	1.881114	1.766841	1.401702
H	1.568727	3.851215	2.665304
H	0.007530	5.599380	1.843475
H	-1.220946	5.229395	-0.285873
H	-0.872623	3.161838	-1.573906
H	4.049703	-0.525941	1.558488
H	6.454023	0.445039	1.072820
H	6.534432	1.672724	-1.247934
H	-1.776063	1.077104	0.975549
H	-3.984432	2.126503	0.757384
H	-3.400659	-2.702795	-0.233415
H	-6.401026	1.946167	0.201636
H	-8.327318	0.591457	-0.534177
H	-5.805008	-2.873903	-0.803638
H	-8.021441	-1.830794	-1.033935
H	1.351808	-5.316081	0.008725
H	0.007842	-4.175167	0.297299
H	0.811175	-4.209395	-1.271555
H	2.739267	-4.219281	2.519127
H	3.150076	-2.501511	2.664594
H	1.460410	-3.015999	2.779424
H	4.607391	-2.591705	-0.106567
H	4.163405	-4.296245	-0.329450
H	3.712147	-3.086053	-1.543027

8c

E(ωB97X-D/6-311+G**)= -2072.272180

C	4.669921	-0.819626	0.791346
C	4.024830	0.315562	0.228223
C	4.770889	1.141637	-0.659918
C	6.120229	0.822677	-0.934186
C	6.719497	-0.276857	-0.369925
C	5.977730	-1.108508	0.499780
C	4.109123	2.259114	-1.239548
C	2.802727	2.532458	-0.956316
C	2.024725	1.700230	-0.086401
C	2.673584	0.616523	0.496529
C	0.645556	2.004537	0.186998
C	-0.309355	1.169883	-0.207868
C	-1.767873	1.117775	-0.002279
Si	-2.747632	2.557827	0.705832
C	-2.234293	4.114573	-0.210074
P	-0.400932	-0.473653	-1.198932
C	0.249829	-1.752773	-0.033821
C	1.364693	-2.503633	-0.416461
C	1.947953	-3.415125	0.455956
C	1.411788	-3.606789	1.724828
C	0.292302	-2.876045	2.112980
C	-0.280017	-1.956913	1.243295
C	-2.072062	-0.108762	-0.516759
C	-3.297982	-0.877582	-0.516819
S	-3.764493	-1.829202	-1.898894
C	-5.181004	-2.410988	-1.104748
C	-5.286135	-1.907809	0.156848
C	-4.208607	-1.043303	0.494387

C	-4.614200	2.340318	0.510776
C	-2.313727	2.726217	2.527841
H	1.798106	-2.348483	-1.399618
H	2.827813	-3.969430	0.145626
H	1.864000	-4.318266	2.408354
H	-0.131494	-3.016598	3.102495
H	-1.135487	-1.369832	1.561406
H	-5.847718	-3.094767	-1.608690
H	-6.095534	-2.156788	0.831789
H	-4.081695	-0.573668	1.460702
H	-2.741164	4.999456	0.190847
H	-1.152509	4.234580	-0.105976
H	-2.466301	4.035836	-1.276637
H	-2.801775	3.595040	2.982810
H	-2.607585	1.833937	3.090066
H	-1.229480	2.842386	2.614977
H	-5.113088	3.302428	0.673674
H	-4.867628	1.990532	-0.494336
H	-5.028814	1.621527	1.221534
H	2.316321	3.395651	-1.399878
H	4.666555	2.900462	-1.917905
H	2.118696	-0.030319	1.167839
H	6.680741	1.464575	-1.609180
H	7.755767	-0.510695	-0.591347
H	4.094633	-1.462805	1.450654
H	6.449622	-1.983056	0.937433

TS-4c

E(ωB97X-D/6-311+G**)= -2072.259755

C	-5.359623	1.370187	0.032718
C	-4.637580	0.147197	0.027539
C	-5.365714	-1.067315	-0.099745
C	-6.775113	-1.019561	-0.219810
C	-7.444578	0.177255	-0.214460
C	-6.724501	1.386496	-0.085674
C	-4.635974	-2.285896	-0.101088
C	-3.276621	-2.293217	0.009012
C	-2.531113	-1.078199	0.129036
C	-3.229597	0.110346	0.151237
C	-1.099599	-1.164988	0.251255
C	-0.100331	-1.954888	0.197387
C	1.287502	-1.644305	0.130088
Si	2.560317	-3.006027	0.264464
C	3.915353	-2.872289	-1.043328
C	1.510478	-0.291723	-0.145681
P	0.073317	0.705290	-0.447961
C	0.613261	2.387500	0.100831
C	1.303159	2.630634	1.296725
C	1.619696	3.921880	1.697915
C	1.232702	5.014100	0.925952
C	0.528556	4.796033	-0.253308
C	0.227077	3.500209	-0.657682
C	2.872303	0.251400	-0.236163
S	3.476470	0.963232	-1.700709
C	5.021111	1.266208	-1.000418
C	5.066154	0.856077	0.298428

C	3.839365	0.279784	0.731258
C	3.400471	-3.097523	1.960494
C	1.679297	-4.655206	0.022774
H	5.802564	1.740268	-1.575875
H	5.939702	0.966585	0.928944
H	3.656716	-0.090870	1.730907
H	-0.307225	3.338619	-1.589613
H	0.220874	5.637997	-0.866470
H	1.474563	6.023765	1.242442
H	2.162709	4.078623	2.625228
H	1.590869	1.789635	1.917940
H	-2.720671	-3.223770	0.001810
H	-5.178160	-3.222357	-0.199299
H	-2.680557	1.039400	0.259726
H	-7.320680	-1.953824	-0.319643
H	-8.525051	0.200500	-0.310962
H	-4.805525	2.298634	0.130595
H	-7.257878	2.331567	-0.081893
H	2.380652	-5.493032	0.110051
H	0.890490	-4.777827	0.770742
H	1.201665	-4.698759	-0.960020
H	3.923759	-4.053331	2.078791
H	4.132728	-2.296602	2.092115
H	2.658734	-3.016822	2.761236
H	4.559408	-3.758927	-1.018543
H	3.474304	-2.798124	-2.041898
H	4.541471	-1.989181	-0.889788

9c

E(ωB97X-D/6-311+G**)= -2072.290325

C	5.258024	-1.073389	-0.971751
C	4.407695	-0.017713	-0.551574
C	5.002597	1.155771	-0.014683
C	6.411199	1.233218	0.083194
C	7.209106	0.196104	-0.331424
C	6.621480	-0.971499	-0.865734
C	4.142377	2.207502	0.406697
C	2.788524	2.096921	0.301819
C	2.170198	0.925511	-0.236332
C	2.996723	-0.101150	-0.647848
C	0.704895	0.854653	-0.285742
C	-0.138306	1.854196	0.119069
C	-1.548064	1.396995	-0.016751
Si	-2.816070	2.698995	0.454243
C	-2.419103	4.267908	-0.508702
C	-1.747180	0.135267	-0.490403
C	-2.995704	-0.619212	-0.546210
C	-3.804973	-0.970258	0.498667
C	-4.942601	-1.732613	0.111186
C	-4.985238	-1.969741	-1.228992
S	-3.632125	-1.263439	-2.033945
P	-0.157386	-0.593922	-1.002746
C	-0.027789	-1.910618	0.292372
C	0.281960	-1.607103	1.620791
C	0.333326	-2.609178	2.580382
C	0.071807	-3.931497	2.230607

C	-0.237749	-4.244181	0.913000
C	-0.280078	-3.239520	-0.049645
C	-4.615911	2.256836	0.076424
C	-2.656223	3.051560	2.298993
H	-5.726026	-2.523619	-1.786490
H	-5.690970	-2.095809	0.804466
H	-3.567757	-0.694253	1.517757
H	-0.517465	-3.486376	-1.079910
H	-0.443539	-5.272142	0.631582
H	0.111986	-4.713000	2.982622
H	0.576727	-2.358341	3.607985
H	0.477430	-0.572998	1.887645
H	2.119006	2.888035	0.621479
H	4.586713	3.109778	0.819376
H	2.567576	-1.009811	-1.061735
H	6.855231	2.134688	0.496726
H	8.288548	0.269976	-0.248871
H	4.805363	-1.971809	-1.380840
H	7.254515	-1.790549	-1.192195
H	-3.046741	5.106476	-0.186333
H	-1.367060	4.520550	-0.355937
H	-2.577067	4.113666	-1.580995
H	-3.275175	3.901976	2.605570
H	-2.954140	2.180022	2.891082
H	-1.609925	3.274106	2.526306
H	-5.238350	3.155938	0.154300
H	-4.722928	1.857153	-0.936854
H	-5.012139	1.505641	0.763719

1c' + PhTSMSP^{-}

E(ωB97X-D/6-311+G**)= -2072.235340

C	-3.742957	-2.239598	1.941866
C	-2.998547	-2.009807	0.758286
C	-3.692553	-1.807129	-0.463006
C	-5.106857	-1.852609	-0.461955
C	-5.802241	-2.088438	0.694796
C	-5.112500	-2.278233	1.911763
C	-2.947099	-1.541392	-1.641263
C	-1.585175	-1.492025	-1.615929
C	-0.885873	-1.717963	-0.398634
C	-1.583841	-1.964799	0.761058
C	0.537329	-1.666563	-0.385038
C	1.744671	-1.614417	-0.360369
C	3.087757	-1.390215	-0.302906
C	4.264646	-1.118558	-0.237814
C	5.604138	-0.691815	-0.149758
S	6.940754	-1.793352	-0.334463
C	8.081007	-0.525663	-0.104057
C	7.469583	0.675247	0.103087
C	6.053042	0.584130	0.077987
P	-0.414599	1.866552	-1.198065
Si	0.482964	2.179571	0.800460
C	-0.104289	3.760564	1.680114
C	-2.201472	1.763458	-0.877055
C	-3.068453	1.801678	-1.995782
C	-4.444032	1.680718	-1.879462

C	-5.042537	1.510329	-0.631771
C	-4.219071	1.466617	0.489166
C	-2.841719	1.588267	0.370528
C	0.355975	0.818392	2.132201
C	2.349949	2.378517	0.510572
H	-2.627942	1.936279	-2.980387
H	-5.059446	1.719571	-2.775446
H	-6.117722	1.401431	-0.535997
H	-4.654768	1.312617	1.472875
H	-2.247196	1.530408	1.274604
H	0.827758	1.161506	3.061592
H	-0.678213	0.545807	2.362114
H	0.871836	-0.087346	1.803463
H	0.384894	3.888437	2.654045
H	0.116461	4.634776	1.060143
H	-1.186753	3.739085	1.841364
H	2.874422	2.548995	1.458520
H	2.759160	1.474574	0.048749
H	2.553345	3.221473	-0.157033
H	-1.017173	-1.235257	-2.500282
H	-3.481384	-1.336281	-2.561911
H	-1.047919	-2.107302	1.692960
H	-5.630924	-1.675345	-1.395153
H	-6.886856	-2.110966	0.682644
H	-5.670731	-2.449950	2.826190
H	-3.208820	-2.378287	2.876862
H	5.370825	1.412756	0.217016
H	8.009669	1.598389	0.266855
H	9.139608	-0.736163	-0.137588

TS-1c’

E(ωB97X-D/6-311+G**)= -2072.227831

C	-3.650879	-2.097375	2.079502
C	-3.005193	-1.889473	0.834578
C	-3.780486	-1.941605	-0.353041
C	-5.168734	-2.195757	-0.258027
C	-5.768876	-2.388084	0.959480
C	-4.998553	-2.338246	2.142647
C	-3.134437	-1.701660	-1.594563
C	-1.807296	-1.399365	-1.648222
C	-1.024296	-1.333713	-0.461672
C	-1.623026	-1.594958	0.748720
C	0.364750	-0.952171	-0.523723
C	1.562997	-1.237388	-0.377351
C	2.902418	-1.094048	-0.279322
C	4.110956	-0.987489	-0.153942
C	5.494087	-0.829241	-0.001421
S	6.607871	-1.390754	-1.226713
C	7.974426	-0.854977	-0.317248
C	7.595645	-0.284038	0.860457
C	6.187636	-0.264759	1.045441
P	-0.151854	1.573976	-1.083632
Si	0.527505	2.210449	0.948235
C	-0.295809	3.828413	1.490401
C	-1.974566	1.695755	-0.978848
C	-2.682631	1.867649	-2.184559

C	-4.068331	1.829621	-2.236800
C	-4.812555	1.615840	-1.079085
C	-4.140876	1.456723	0.127851
C	-2.753659	1.499206	0.176090
C	0.314247	0.997440	2.394487
C	2.386253	2.515515	0.811721
H	-2.117819	2.023988	-3.099165
H	-4.572253	1.960701	-3.190428
H	-5.895812	1.565860	-1.118141
H	-4.699109	1.265731	1.039618
H	-2.265542	1.336447	1.129767
H	0.745200	1.430004	3.305587
H	-0.735127	0.765853	2.598772
H	0.833576	0.058658	2.182854
H	0.096548	4.160759	2.458979
H	-0.109359	4.614738	0.753224
H	-1.379488	3.710216	1.581705
H	2.798104	2.850311	1.771000
H	2.910416	1.601602	0.516360
H	2.600185	3.280338	0.059432
H	-1.329672	-1.161228	-2.591163
H	-3.725420	-1.720319	-2.504701
H	-1.035574	-1.538013	1.658203
H	-5.755903	-2.219877	-1.171203
H	-6.836275	-2.572821	1.019830
H	-5.479626	-2.487548	3.103744
H	-3.057554	-2.051181	2.987782
H	5.685443	0.154193	1.906438
H	8.300538	0.119673	1.576404
H	8.972042	-0.982841	-0.709617

6c’

E(ωB97X-D/6-311+G**)= -2072.259827

C	-4.397437	-0.276695	1.049702
C	-4.085855	-1.425417	0.352654
S	-5.563153	-2.299058	0.000152
C	-6.548521	-1.093187	0.738374
C	-5.787114	-0.091015	1.269185
C	-2.820539	-2.002579	-0.013700
C	-1.767509	-1.266336	-0.227598
C	-0.663744	-0.672898	-0.463756
C	0.490405	-0.050050	-0.703985
P	1.981177	-1.028670	-1.181061
C	3.112037	-0.807013	0.272620
C	4.469926	-1.106031	0.126426
C	5.355168	-0.961395	1.188634
C	4.899757	-0.492157	2.415955
C	3.553738	-0.177442	2.570602
C	2.667992	-0.337363	1.511798
C	0.589052	1.429469	-0.682402
C	-0.508950	2.210685	-0.374953
C	-0.435215	3.618208	-0.311866
C	0.802152	4.269422	-0.570194
C	1.920356	3.463818	-0.894732
C	1.817529	2.101355	-0.947993
C	0.870358	5.682860	-0.492185

C	-0.236691	6.424891	-0.175096
C	-1.471306	5.780735	0.077324
C	-1.566761	4.416991	0.010542
Si	1.348523	-3.169459	-0.713058
C	2.938463	-4.168305	-0.947964
C	0.761551	-3.422038	1.050970
C	0.072781	-3.768713	-1.950448
H	4.836011	-1.454176	-0.835242
H	6.404720	-1.202921	1.052958
H	5.590459	-0.367259	3.243469
H	3.187944	0.193778	3.522540
H	1.616790	-0.100190	1.639659
H	0.676294	-4.495230	1.256676
H	1.469573	-2.993169	1.766224
H	-0.220391	-2.968223	1.201446
H	2.729677	-5.233993	-0.801849
H	3.352951	-4.039283	-1.952460
H	3.703184	-3.874166	-0.223083
H	-0.026188	-4.857740	-1.878704
H	-0.904400	-3.325783	-1.741072
H	0.363761	-3.520582	-2.975599
H	2.696193	1.517843	-1.198676
H	2.873351	3.944298	-1.097036
H	-1.457200	1.719878	-0.177904
H	1.821381	6.170429	-0.687238
H	-0.170935	7.506267	-0.115772
H	-2.345081	6.374312	0.325883
H	-2.512956	3.920667	0.202831
H	-3.626536	0.404301	1.388229
H	-6.209181	0.763527	1.785042
H	-7.625695	-1.173988	0.728474

TS-2c’

E(ωB97X-D/6-311+G**)= -2072.256956

C	-2.587275	3.600281	0.157051
C	-1.289990	3.171897	-0.234106
C	-0.326765	4.155154	-0.585665
C	-0.686423	5.525112	-0.533315
C	-1.945177	5.907614	-0.151339
C	-2.907296	4.930705	0.197765
C	-0.942033	1.802424	-0.278553
C	0.315807	1.378870	-0.661158
C	1.268309	2.382257	-1.008193
C	0.961981	3.713753	-0.974219
C	0.689111	-0.057595	-0.689721
P	2.374197	-0.579084	-1.217604
C	3.458031	-0.103907	0.203955
C	4.846601	-0.220659	0.072217
C	5.698262	0.102259	1.121435
C	5.179225	0.575103	2.322709
C	3.802465	0.711836	2.462854
C	2.951121	0.370032	1.418303
Si	1.990066	-2.771899	-0.671223
C	3.744306	-3.506509	-0.905210
C	1.595958	-3.145900	1.135027
C	0.941100	-3.722418	-1.918656

C	-0.106383	-1.117912	-0.449980
C	-1.426853	-1.251647	-0.131229
C	-2.577637	-1.550917	0.183919
C	-3.896344	-1.836035	0.541980
S	-5.081847	-2.268506	-0.675093
C	-6.334217	-2.444018	0.509467
C	-5.863526	-2.190719	1.761504
C	-4.485536	-1.844615	1.790127
H	5.261189	-0.571625	-0.868367
H	6.771353	-0.006187	0.997578
H	5.842318	0.837687	3.140533
H	3.385219	1.081192	3.394310
H	1.877331	0.462628	1.543426
H	2.093379	-4.071292	1.448948
H	1.963288	-2.335659	1.772699
H	0.520430	-3.237161	1.292949
H	3.727484	-4.587358	-0.719088
H	4.119318	-3.349088	-1.922643
H	4.464039	-3.062445	-0.209259
H	1.292383	-4.757062	-2.010542
H	-0.108455	-3.717291	-1.621037
H	1.010921	-3.254391	-2.905692
H	2.263972	2.075880	-1.310045
H	1.711749	4.451934	-1.244308
H	-1.693563	1.071254	-0.004128
H	0.056624	6.270280	-0.803000
H	-2.209037	6.959496	-0.115632
H	-3.902702	5.241157	0.497873
H	-3.322277	2.846800	0.422558
H	-3.931028	-1.606733	2.687350
H	-6.484470	-2.244359	2.647627
H	-7.334717	-2.715386	0.208734

(Z)-7c’

E(ωB97X-D/6-311+G**)= -2072.293861

S	3.585964	-2.452831	-0.031580
C	3.951815	-0.741855	-0.067683
C	5.315544	-0.550525	-0.037595
C	6.053009	-1.764035	0.013405
C	5.261510	-2.873551	0.021793
C	2.915083	0.198597	-0.115446
C	1.962057	0.957425	-0.122186
C	0.883462	1.856236	-0.117122
Si	1.345736	3.668486	-0.049813
C	3.223747	3.789603	0.108004
C	-0.429385	1.353632	-0.142935
P	-1.820264	2.385347	-0.493608
C	-3.272805	1.370698	0.007466
C	-4.392155	1.343681	-0.835979
C	-5.546115	0.645459	-0.496599
C	-5.613442	-0.058274	0.700561
C	-4.517781	-0.035022	1.559443
C	-3.376698	0.681079	1.225519
C	-0.600905	-0.114789	0.061747
C	-0.108277	-0.720392	1.249652
C	-0.276973	-2.053716	1.489356

C	-0.943525	-2.880917	0.550136
C	-1.431024	-2.289398	-0.644887
C	-1.241206	-0.903051	-0.860118
C	-2.108313	-3.108636	-1.584144
C	-2.288384	-4.446302	-1.352812
C	-1.795486	-5.035255	-0.165982
C	-1.140101	-4.268306	0.760779
C	0.860842	4.635680	-1.595457
C	0.593572	4.507329	1.462865
H	-4.343720	1.867385	-1.786621
H	-6.390391	0.638801	-1.179961
H	-6.506321	-0.616952	0.961924
H	-4.554577	-0.574579	2.500974
H	-2.541991	0.698773	1.917514
H	-1.630081	-0.448883	-1.765419
H	0.106454	-2.498662	2.402695
H	0.410449	-0.094885	1.967445
H	0.816091	5.580330	1.466770
H	-0.491364	4.377956	1.474167
H	1.001732	4.069668	2.379435
H	3.531746	4.838113	0.187945
H	3.579929	3.256311	0.994300
H	3.720963	3.348216	-0.760729
H	1.224516	5.668068	-1.531891
H	1.297006	4.173481	-2.486734
H	-0.224168	4.654520	-1.722840
H	-2.489479	-2.650210	-2.491276
H	-2.812357	-5.059000	-2.079031
H	-1.940182	-6.096485	0.007836
H	-0.759142	-4.715872	1.674157
H	5.562533	-3.909865	0.056296
H	7.134960	-1.808914	0.042620
H	5.757253	0.436608	-0.053479

TS-3c'

E(ωB97X-D/6-311+G**)= -2072.264652

C	3.264718	-2.686979	-1.643667
C	2.200808	-1.791970	-1.778820
C	2.413265	-0.621421	-2.515428
C	3.656700	-0.339010	-3.063809
C	4.714787	-1.228318	-2.897642
C	4.514102	-2.406748	-2.189322
P	0.549758	-2.232149	-1.054030
C	0.125887	-0.931606	-0.066844
C	-1.197195	-1.015277	0.614636
Si	-1.305538	-1.771079	2.280757
C	0.193689	-1.287968	3.332388
C	0.936854	0.283223	0.211546
C	0.313579	1.559973	0.112680
C	1.017035	2.704736	0.354791
C	2.380899	2.657440	0.747254
C	2.999783	1.387399	0.886406
C	2.250161	0.218965	0.609239
C	4.360033	1.328972	1.283003
C	5.071430	2.473510	1.529344
C	4.453920	3.737241	1.393332

C	3.140241	3.823528	1.011631
C	-2.288647	-0.423107	0.035191
C	-3.263926	0.133029	-0.474552
C	-4.379243	0.728839	-1.046657
S	-5.916042	0.757830	-0.188055
C	-6.706310	1.617720	-1.476011
C	-5.837325	1.864914	-2.495349
C	-4.526045	1.370275	-2.267107
C	-2.883374	-1.203528	3.141040
C	-1.319072	-3.665919	2.250901
H	-1.328381	-4.090898	3.261994
H	1.599537	0.085968	-2.636893
H	3.803950	0.582996	-3.616527
H	5.688245	-1.003745	-3.320856
H	5.330981	-3.109590	-2.059092
H	3.117579	-3.610756	-1.090636
H	-0.734901	1.602554	-0.160070
H	0.531625	3.671527	0.260039
H	2.734804	-0.745906	0.710921
H	0.147614	-1.742020	4.328815
H	1.121158	-1.621584	2.854648
H	0.258385	-0.201743	3.446989
H	-2.991173	-1.672577	4.125245
H	-2.883340	-0.117347	3.270044
H	-3.760112	-1.460054	2.538957
H	-2.199820	-4.031349	1.713703
H	-0.435031	-4.046135	1.728387
H	-7.750859	1.878793	-1.400671
H	-6.126328	2.389539	-3.399014
H	-3.704946	1.466481	-2.964312
H	4.830459	0.355517	1.380896
H	6.112499	2.413694	1.828787
H	2.661352	4.792549	0.906116
H	5.024037	4.638652	1.592210

(E)-7c’

E(ωB97X-D/6-311+G**)= -2072.292606

S	5.545549	1.812872	0.176288
C	5.032326	0.158192	-0.059268
C	6.130035	-0.653025	-0.246505
C	7.364383	0.048701	-0.202400
C	7.211974	1.385261	0.016539
C	3.672942	-0.183116	-0.039494
C	2.487219	-0.460637	-0.031090
C	1.138018	-0.845313	-0.007047
Si	0.890158	-2.696317	-0.077550
C	2.403700	-3.462067	-0.908415
C	0.139766	0.146990	-0.020191
P	0.495714	1.853195	-0.279777
C	-1.145678	2.657541	0.014729
C	-1.693594	3.485453	-0.973379
C	-2.904536	4.145144	-0.785010
C	-3.608683	3.991420	0.403526
C	-3.073276	3.189586	1.407628
C	-1.854668	2.551665	1.220415
C	-1.262272	-0.311493	0.175319

C	-1.640637	-0.883561	1.417644
C	-2.918583	-1.314710	1.642909
C	-3.908816	-1.198125	0.636184
C	-3.543443	-0.617328	-0.608001
C	-2.212327	-0.184619	-0.806998
C	-4.534199	-0.486464	-1.615955
C	-5.817530	-0.911181	-1.402310
C	-6.178951	-1.492772	-0.164376
C	-5.245719	-1.630590	0.827878
C	0.763964	-3.503504	1.631712
C	-0.621864	-3.248608	-1.066534
H	-1.165924	3.594636	-1.916637
H	-3.305511	4.770948	-1.577139
H	-4.561727	4.490166	0.547458
H	-3.606002	3.064428	2.345505
H	-1.444922	1.943998	2.019969
H	-1.935728	0.268349	-1.753103
H	-3.191139	-1.746787	2.601466
H	-0.886870	-0.960602	2.193489
H	-0.554564	-4.326583	-1.254755
H	-1.560230	-3.054561	-0.541458
H	-0.667575	-2.735834	-2.031570
H	2.278917	-4.545910	-1.009718
H	2.557054	-3.035594	-1.904198
H	3.310045	-3.269414	-0.327929
H	0.847807	-4.593708	1.552983
H	1.567679	-3.146823	2.283469
H	-0.191190	-3.273290	2.111953
H	-4.253645	-0.033469	-2.561554
H	-6.564644	-0.801148	-2.181331
H	-7.198820	-1.826685	-0.004372
H	-5.518050	-2.074694	1.780963
H	7.978442	2.142307	0.088901
H	8.329794	-0.424959	-0.332245
H	6.032791	-1.717249	-0.413466

TS-5c'

E(ωB97X-D/6-311+G**)= -2072.259399

C	4.777152	1.109590	-1.740857
C	3.962042	0.393006	-0.825393
C	4.583067	-0.248802	0.279915
C	5.988462	-0.153307	0.433008
C	6.748581	0.545169	-0.467445
C	6.133189	1.184658	-1.568304
C	2.558624	0.308172	-0.981118
C	1.772449	-0.389173	-0.090586
C	2.408900	-1.009329	1.022714
C	3.761381	-0.948417	1.200131
C	0.312052	-0.424231	-0.216346
P	-0.771254	0.847975	-0.924233
C	-0.472695	2.309304	0.140448
C	-0.977686	3.551290	-0.277891
C	-0.817991	4.696396	0.489780
C	-0.131142	4.640799	1.699932
C	0.375786	3.419416	2.133103
C	0.196683	2.270463	1.372869

C	-0.536520	-1.455019	0.085912
Si	-0.264651	-3.308400	0.280598
C	-1.619943	-4.219048	-0.649268
C	-1.883834	-0.941121	0.052203
C	-3.111097	-1.126734	0.333127
C	-4.311953	-0.429050	0.039422
C	-5.474192	-0.355810	0.771368
C	-6.475189	0.468172	0.192159
C	-6.091045	1.009211	-1.000330
S	-4.481840	0.555728	-1.418605
C	1.395981	-3.863974	-0.424136
C	-0.369088	-3.772954	2.104741
H	-1.503557	3.611828	-1.226818
H	-1.225714	5.639928	0.138960
H	0.002610	5.536322	2.298146
H	0.905226	3.357180	3.079330
H	0.574418	1.323796	1.743895
H	-1.516646	-5.303471	-0.529454
H	-2.597730	-3.904885	-0.275458
H	-1.582354	-3.984823	-1.717414
H	-0.326207	-4.858483	2.244916
H	0.446505	-3.325551	2.681453
H	-1.313767	-3.407233	2.517931
H	1.415254	-4.956394	-0.509097
H	1.544009	-3.444146	-1.423801
H	2.244669	-3.554485	0.189557
H	1.786759	-1.516664	1.752426
H	4.222919	-1.422838	2.061343
H	2.082561	0.805718	-1.820489
H	6.455063	-0.642972	1.282836
H	7.823443	0.613710	-0.337481
H	6.741481	1.740196	-2.274287
H	4.300559	1.602777	-2.582145
H	-6.673556	1.638129	-1.658230
H	-7.448748	0.637649	0.636515
H	-5.585672	-0.891018	1.705208

8c'

E(ωB97X-D/6-311+G**)= -2072.269898

S	4.185912	-0.301296	-1.984679
C	4.048981	0.453095	-0.386088
C	5.179745	0.139870	0.342032
C	6.111135	-0.685130	-0.343086
C	5.731955	-0.984372	-1.619207
C	2.911002	1.222958	0.017463
C	1.665152	0.804267	-0.205958
P	0.767245	-0.714862	-0.951006
C	0.911347	-2.016435	0.347607
C	2.132546	-2.275068	0.976904
C	2.237905	-3.281975	1.930327
C	1.128266	-4.044104	2.278382
C	-0.092416	-3.793950	1.658842
C	-0.196880	-2.796456	0.697198
C	-0.504890	0.403297	-0.220544
C	0.357525	1.408513	0.104230
Si	0.199353	3.209867	0.625723

C	1.189315	4.253277	-0.582007
C	-1.949514	0.255540	-0.105332
C	-2.693298	-0.374319	-1.081804
C	-4.090822	-0.552083	-0.954943
C	-4.750747	-0.081047	0.212379
C	-3.971749	0.544016	1.219681
C	-2.623978	0.698893	1.068598
C	-6.150195	-0.263746	0.335437
C	-6.869444	-0.882232	-0.653091
C	-6.215739	-1.350363	-1.816122
C	-4.863875	-1.189282	-1.961576
C	-1.586873	3.830737	0.593506
C	0.907039	3.396799	2.356998
H	3.002521	-1.674505	0.733447
H	3.194852	-3.461897	2.409961
H	1.211274	-4.823917	3.028687
H	-0.968657	-4.377200	1.925203
H	-1.156509	-2.613059	0.222564
H	1.165884	5.313484	-0.305546
H	2.222908	3.897252	-0.581546
H	0.794375	4.153017	-1.597848
H	0.915441	4.442592	2.683027
H	0.335675	2.812750	3.085519
H	1.932777	3.016731	2.343302
H	-1.588164	4.926413	0.620088
H	-2.099622	3.514748	-0.320073
H	-2.177499	3.476230	1.441692
H	-2.032481	1.144214	1.860170
H	-4.462915	0.886145	2.125946
H	-2.194566	-0.739325	-1.974826
H	-6.645752	0.097536	1.231811
H	-7.940537	-1.016384	-0.546229
H	-6.791005	-1.840005	-2.594876
H	-4.360057	-1.549660	-2.853088
H	6.289226	-1.526470	-2.369362
H	7.048966	-1.017842	0.087508
H	5.320691	0.520486	1.345358

TS-4c’

E(ωB97X-D/6-311+G**)= -2072.252205

C	2.847507	-2.581847	-0.283931
C	1.820908	-1.701651	0.111127
C	1.616233	-1.553911	1.495333
C	2.390446	-2.239521	2.420946
C	3.405243	-3.100638	2.008057
C	3.624172	-3.262557	0.641721
P	0.939102	-0.812441	-1.209104
C	-0.343870	0.098559	-0.269263
C	-0.076023	1.291240	0.348480
C	1.301547	1.745774	0.275438
C	2.246962	1.210000	-0.359402
C	3.625295	0.980060	-0.632212
C	4.521680	0.269175	0.123300
C	5.808850	0.176475	-0.471485
C	5.884508	0.815768	-1.673181
S	4.378290	1.533673	-2.104954

C	-1.720927	-0.444384	-0.326390
C	-2.025242	-1.675337	0.321151
C	-3.297187	-2.167826	0.337712
C	-4.355258	-1.481600	-0.318129
C	-4.057098	-0.272811	-1.001197
C	-2.726841	0.218496	-0.986620
C	-5.686904	-1.962008	-0.320705
C	-6.683683	-1.276893	-0.966766
C	-6.389415	-0.072778	-1.643485
C	-5.108177	0.414188	-1.659675
Si	-1.268365	2.328216	1.382386
C	-0.261215	3.194318	2.717417
C	-2.107622	3.659693	0.338621
C	-2.596168	1.316664	2.265282
H	3.039270	-2.720772	-1.344254
H	4.411472	-3.924943	0.292900
H	4.011569	-3.632419	2.733941
H	2.207036	-2.089073	3.481296
H	0.859162	-0.859363	1.844736
H	-2.652994	4.366463	0.973683
H	-1.363240	4.220558	-0.234660
H	-2.820268	3.221608	-0.366058
H	-0.902362	3.814889	3.354010
H	0.248771	2.461449	3.350242
H	0.510761	3.822876	2.266717
H	-3.060713	1.928004	3.047336
H	-3.381453	0.968697	1.590875
H	-2.157283	0.435273	2.742878
H	-1.217576	-2.209792	0.810253
H	-3.516275	-3.100646	0.848963
H	-2.499421	1.143300	-1.506877
H	-5.907111	-2.889223	0.200372
H	-7.699673	-1.657493	-0.960205
H	-7.182833	0.465794	-2.151156
H	-4.878816	1.340149	-2.178344
H	4.241759	-0.188118	1.062966
H	6.642533	-0.348519	-0.022416
H	6.739308	0.904250	-2.327655

9c’

E(ωB97X-D/6-311+G**)= -2072.286336

C	4.895225	0.744589	-0.174808
C	3.779693	0.080954	-0.623189
S	4.256968	-1.412505	-1.392719
C	5.935061	-1.119731	-1.098360
C	6.112886	0.065203	-0.447465
C	2.381587	0.478161	-0.489822
C	1.978377	1.717285	-0.069080
C	0.492393	1.741333	0.006701
Si	-0.206001	3.428690	0.445039
C	0.173533	3.807064	2.253462
C	-0.172658	0.595987	-0.318075
P	1.008372	-0.634353	-0.955916
C	0.914406	-1.866943	0.421618
C	1.480138	-1.619127	1.675310
C	1.361782	-2.550337	2.698378

C	0.673242	-3.742650	2.488835
C	0.104207	-3.997001	1.247605
C	0.231651	-3.066450	0.220190
C	-1.597820	0.272991	-0.173315
C	-2.340534	-0.272732	-1.197499
C	-3.702248	-0.618614	-1.026657
C	-4.320762	-0.413741	0.236084
C	-3.539275	0.126158	1.288912
C	-2.228189	0.451741	1.090943
C	-5.684710	-0.761347	0.400104
C	-6.408212	-1.285488	-0.638427
C	-5.795275	-1.488996	-1.896216
C	-4.478223	-1.164725	-2.082916
C	0.641508	4.710883	-0.641017
C	-2.068314	3.620479	0.161590
H	-0.208345	-3.270382	-0.751020
H	-0.434447	-4.923280	1.074425
H	0.584516	-4.470300	3.289294
H	1.811252	-2.346542	3.665150
H	2.011581	-0.685626	1.834090
H	0.341347	5.729653	-0.370063
H	1.723437	4.604783	-0.533670
H	0.393597	4.547651	-1.694818
H	-0.070136	4.844023	2.509454
H	-0.391102	3.148684	2.921802
H	1.239601	3.641957	2.433518
H	-2.330577	4.684754	0.168692
H	-2.360391	3.207399	-0.809112
H	-2.670207	3.118524	0.922500
H	-1.628768	0.842152	1.906197
H	-3.997718	0.268146	2.263181
H	-1.874593	-0.432432	-2.164785
H	-6.149816	-0.603641	1.368822
H	-7.452565	-1.544377	-0.499681
H	-6.373825	-1.905003	-2.714406
H	-4.004538	-1.321126	-3.047178
H	4.802888	1.696610	0.329607
H	7.089023	0.444374	-0.167347
H	6.679921	-1.831953	-1.420284

1d + PhTMSP^{-}

E(ωB97X-D/6-311+G**)= -1524.066955

C	-3.378932	1.115856	-1.922432
C	-2.562989	1.514815	-0.834491
C	-3.229005	1.606601	0.409425
C	-4.579979	1.317520	0.554118
C	-5.351686	0.924132	-0.535932
C	-4.727268	0.832324	-1.781309
P	-0.801442	1.822074	-1.182566
Si	0.110403	2.087530	0.814967
C	-0.108407	0.658298	2.050042
C	1.982755	2.219501	0.525883
C	-0.361086	3.671539	1.764389
C	0.684288	-2.448338	-0.002417
C	1.821305	-2.057562	-0.115861
C	3.069468	-1.508003	-0.234389

C	4.133029	-0.945867	-0.327625
C	5.375290	-0.170853	-0.425083
C	5.209996	0.892151	-1.526888
C	-0.696943	-2.777799	0.143194
C	-1.104224	-4.037695	0.598126
C	-2.455004	-4.308814	0.761268
C	-3.403814	-3.329897	0.475890
C	-3.002674	-2.077697	0.021515
C	-1.653803	-1.797678	-0.148682
C	5.631287	0.523474	0.925300
C	6.548038	-1.105547	-0.767020
H	-2.916484	1.020235	-2.900987
H	-5.300260	0.526066	-2.652848
H	-6.407130	0.700082	-0.420940
H	-5.035953	1.401196	1.537306
H	-2.678337	1.913515	1.291101
H	0.338950	0.905666	3.020840
H	-1.160271	0.407113	2.214738
H	0.386185	-0.238720	1.666620
H	0.169384	3.737063	2.722987
H	-0.101237	4.549355	1.163965
H	-1.435232	3.724841	1.967093
H	2.508028	2.403549	1.470974
H	2.362564	1.287982	0.096161
H	2.218059	3.036547	-0.163898
H	-1.327659	-0.821002	-0.501671
H	-3.734116	-1.307392	-0.198347
H	-4.459261	-3.542330	0.611893
H	-2.768759	-5.284620	1.117695
H	-0.357678	-4.790482	0.825882
H	7.476386	-0.528911	-0.835702
H	6.672263	-1.872006	0.002517
H	6.379484	-1.605238	-1.724316
H	6.552917	1.112701	0.870292
H	4.803083	1.190330	1.174682
H	5.735491	-0.213048	1.726214
H	6.126453	1.486658	-1.606068
H	5.014127	0.422179	-2.493613
H	4.377133	1.559361	-1.295748

TS-1d

E(ωB97X-D/6-311+G**)= -1524.047820

C	-2.995032	0.978425	-2.016098
C	-2.199012	1.008106	-0.855262
C	-2.889153	1.006610	0.370345
C	-4.275366	0.978669	0.432714
C	-5.037315	0.943694	-0.730925
C	-4.381536	0.943591	-1.958961
P	-0.383792	0.869774	-1.064971
Si	0.399065	1.913014	0.747635
C	0.382733	0.954251	2.385205
C	2.216848	2.304454	0.406545
C	-0.485369	3.559176	1.067285
C	0.168096	-1.430657	-0.151737
C	1.381032	-1.723006	-0.051680
C	2.692473	-1.362615	-0.196346

C	3.867225	-1.064413	-0.281445
C	5.241777	-0.563461	-0.407757
C	5.319718	0.435195	-1.578529
C	-1.173422	-1.888214	0.163904
C	-1.540308	-2.090623	1.497858
C	-2.828242	-2.497782	1.824561
C	-3.771205	-2.710780	0.826257
C	-3.412817	-2.517303	-0.504824
C	-2.129119	-2.110267	-0.832254
C	5.633652	0.154054	0.897891
C	6.216789	-1.727455	-0.662680
H	-2.499167	0.975995	-2.982605
H	-4.955001	0.915474	-2.881529
H	-6.120925	0.909172	-0.681125
H	-4.764606	0.960287	1.402148
H	-2.329830	0.986135	1.298621
H	0.831135	1.556861	3.184590
H	-0.628797	0.677899	2.697210
H	0.961218	0.032242	2.278604
H	-0.061504	4.064911	1.943213
H	-0.378535	4.219589	0.201560
H	-1.555127	3.411864	1.240555
H	2.689080	2.771998	1.278624
H	2.754414	1.380804	0.175393
H	2.315302	2.980839	-0.447951
H	-1.859147	-1.921022	-1.864536
H	-4.147158	-2.657556	-1.290804
H	-4.782799	-3.009019	1.081341
H	-3.096600	-2.640484	2.866844
H	-0.802884	-1.913162	2.272233
H	7.242654	-1.352077	-0.757439
H	6.181738	-2.446582	0.159812
H	5.953886	-2.253564	-1.584218
H	6.652025	0.551703	0.818102
H	4.950144	0.981642	1.100946
H	5.593234	-0.537676	1.743435
H	6.338623	0.828024	-1.679409
H	5.038777	-0.051380	-2.515998
H	4.635131	1.270400	-1.414348

6d

E(ωB97X-D/6-311+G**)= -1524.075499

C	2.283365	0.211241	1.432442
C	2.607843	-0.331245	0.185914
C	3.902014	-0.828349	-0.000149
C	4.834262	-0.809575	1.030671
C	4.496991	-0.267822	2.266545
C	3.219552	0.247780	2.458982
P	1.402965	-0.399771	-1.222715
Si	0.595230	-2.489481	-0.796760
C	0.075399	-2.763255	0.984847
C	0.020845	0.685265	-0.657572
C	-1.161307	0.152100	-0.347795
C	-2.283276	-0.390951	-0.054164
C	-3.344603	-1.081436	0.235505
C	-4.639805	-0.410888	0.656791

C	-5.061564	0.658286	-0.366001
C	0.239375	2.153393	-0.647941
C	-0.797046	3.022428	-0.266665
C	-0.613924	4.394665	-0.227996
C	0.615885	4.956790	-0.567369
C	1.653286	4.114229	-0.945368
C	1.469776	2.736190	-0.982966
C	-5.762194	-1.453864	0.779773
C	-4.430444	0.254989	2.028401
C	-0.788722	-2.915784	-1.990022
C	2.067236	-3.627534	-1.151618
H	4.179364	-1.234992	-0.968510
H	5.829317	-1.211060	0.865263
H	5.226024	-0.242068	3.069895
H	2.946836	0.678951	3.416987
H	1.284772	0.605274	1.590694
H	-0.019390	-3.838135	1.178353
H	0.818435	-2.356627	1.677563
H	-0.892622	-2.294017	1.176854
H	1.754899	-4.673096	-1.050312
H	2.456391	-3.486460	-2.164639
H	2.883011	-3.451935	-0.444168
H	-0.954434	-3.999187	-1.993621
H	-1.717845	-2.429725	-1.679354
H	-0.545773	-2.605496	-3.010860
H	2.299303	2.104100	-1.280170
H	2.620968	4.529672	-1.210082
H	0.761080	6.031641	-0.532974
H	-1.438468	5.035099	0.071243
H	-1.754853	2.587154	-0.001512
H	-6.707136	-0.993009	1.098724
H	-5.923020	-1.951014	-0.181551
H	-5.482025	-2.222883	1.505326
H	-6.014824	1.123211	-0.081728
H	-4.298736	1.437888	-0.442718
H	-5.181453	0.210565	-1.357577
H	-5.366467	0.695585	2.396041
H	-4.085675	-0.480781	2.761696
H	-3.673045	1.040720	1.961036

TS-2d

E(ωB97X-D/6-311+G**)= -1524.072906

C	2.411765	0.226501	1.388598
C	2.723669	-0.327392	0.142910
C	4.001783	-0.868727	-0.034477
C	4.928785	-0.879936	1.001066
C	4.604077	-0.324966	2.234897
C	3.343306	0.232719	2.420338
P	1.514361	-0.364359	-1.260798
Si	0.533743	-2.369831	-0.778788
C	0.074802	-2.683651	1.022308
C	0.105748	0.665190	-0.672808
C	-1.002696	-0.050025	-0.403421
C	-2.283613	0.192448	-0.038306
C	-3.470870	0.322765	0.306041
C	-4.732388	-0.372218	0.661580

C	-5.953755	0.453804	0.222580
C	0.244471	2.141754	-0.631499
C	1.459567	2.775771	-0.933852
C	1.592516	4.158044	-0.879896
C	0.513664	4.955130	-0.516310
C	-0.699516	4.343804	-0.209014
C	-0.834727	2.964021	-0.263702
C	-4.791106	-1.759508	-0.006519
C	-4.771435	-0.547468	2.190638
C	-0.811886	-2.881554	-1.996374
C	1.959973	-3.597484	-1.110832
H	4.269544	-1.286665	-1.000719
H	5.911036	-1.314133	0.841125
H	5.328893	-0.322961	3.042636
H	3.079261	0.671944	3.377298
H	1.424259	0.648861	1.543578
H	0.107419	-3.759364	1.234251
H	0.794165	-2.189830	1.683408
H	-0.918207	-2.294469	1.248470
H	1.609582	-4.627340	-0.971418
H	2.341478	-3.509010	-2.133777
H	2.797363	-3.435933	-0.424272
H	-0.900784	-3.974327	-2.026989
H	-1.769788	-2.443080	-1.714594
H	-0.563673	-2.535863	-3.005010
H	2.317152	2.175356	-1.218452
H	2.548929	4.612402	-1.120821
H	0.615560	6.034789	-0.470606
H	-1.554211	4.949450	0.077513
H	-1.787258	2.507795	-0.021606
H	-5.717192	-2.288004	0.255383
H	-4.745778	-1.659180	-1.094541
H	-3.938028	-2.366574	0.307498
H	-5.697599	-1.051344	2.493326
H	-3.921147	-1.145444	2.528740
H	-4.722439	0.423968	2.689994
H	-6.887745	-0.055867	0.491222
H	-5.938784	1.437239	0.700090
H	-5.942639	0.606479	-0.860388

(Z)-7d

E(ωB97X-D/6-311+G**)= -1524.113685

C	-3.280738	-0.225751	1.034293
C	-3.220664	0.537886	-0.144166
C	-4.394003	0.596066	-0.915077
C	-5.550700	-0.078909	-0.545021
C	-5.574751	-0.853975	0.610764
C	-4.426511	-0.921292	1.395391
P	-1.777863	1.540578	-0.676007
C	-0.368805	0.528001	-0.253011
C	0.904374	1.070974	-0.100960
C	2.037072	0.210138	-0.122372
C	3.001056	-0.522498	-0.138990
C	4.068128	-1.529292	-0.088802
C	3.834792	-2.569587	-1.200257
C	-0.501813	-0.959548	-0.187556

C	-0.069225	-1.661076	0.941006
C	-0.206343	-3.039533	1.024018
C	-0.769899	-3.750876	-0.031333
C	-1.194785	-3.065597	-1.163609
C	-1.066451	-1.683767	-1.237597
Si	1.266888	2.887803	0.160737
C	0.884978	3.979985	-1.330711
C	0.356504	3.561231	1.669270
C	3.119034	3.068582	0.500823
C	5.439599	-0.861657	-0.284226
C	4.032028	-2.240460	1.277866
H	-4.384914	1.174682	-1.834988
H	-6.435619	-0.011629	-1.171999
H	-6.472238	-1.393544	0.896151
H	-4.425893	-1.513715	2.306008
H	-2.409375	-0.275153	1.677101
H	-1.415289	-1.142558	-2.109891
H	-1.638975	-3.607615	-1.992538
H	-0.881827	-4.828649	0.032363
H	0.125357	-3.561532	1.916549
H	0.378244	-1.105907	1.758385
H	0.543218	4.634057	1.793014
H	-0.719449	3.403749	1.564220
H	0.694560	3.048179	2.575375
H	3.371013	4.118374	0.689538
H	3.417404	2.478608	1.372784
H	3.712557	2.717116	-0.348380
H	1.192535	5.015628	-1.142858
H	1.418833	3.618833	-2.215743
H	-0.184605	3.966335	-1.553747
H	6.239479	-1.610068	-0.238217
H	5.487744	-0.360155	-1.254495
H	5.617108	-0.112442	0.492163
H	4.810235	-3.011545	1.332949
H	4.192412	-1.522477	2.086659
H	3.058168	-2.713048	1.429527
H	4.606428	-3.348160	-1.165532
H	2.854346	-3.037608	-1.080121
H	3.862793	-2.091928	-2.182946

TS-3d

E(ωB97X-D/6-311+G**)= -1524.076069

C	-0.269546	-0.430558	2.135784
C	-0.910738	0.205570	1.064852
C	-2.106010	0.886592	1.313384
C	-2.661928	0.913264	2.586338
C	-2.036692	0.249891	3.635187
C	-0.837695	-0.419060	3.401902
C	-0.276550	0.206110	-0.282469
C	1.149792	0.630723	-0.359016
Si	1.561337	2.413021	-0.436279
C	1.329501	3.290858	1.232475
P	-1.010284	-0.183477	-1.750169
C	-2.749535	-0.682660	-1.324837
C	-3.816880	-0.078441	-1.994371
C	-5.133143	-0.449247	-1.735815

C	-5.404397	-1.449587	-0.809809
C	-4.350897	-2.078840	-0.152559
C	-3.039900	-1.702416	-0.411392
C	2.131361	-0.261287	-0.001188
C	3.016091	-1.038955	0.408099
C	3.989298	-2.037487	-0.122815
C	4.262862	-1.799837	-1.620008
C	5.318723	-1.953316	0.646995
C	3.400766	-3.447042	0.061172
C	3.367431	2.631422	-0.937997
C	0.456953	3.341840	-1.662065
H	1.537922	4.366384	1.176188
H	-2.226485	-2.187615	0.117950
H	-4.552721	-2.861252	0.571961
H	-6.429603	-1.739506	-0.603449
H	-5.947400	0.044814	-2.256950
H	-3.614133	0.703729	-2.721096
H	0.683459	-0.918909	1.961071
H	-0.332116	-0.925970	4.217757
H	-2.473640	0.262979	4.628619
H	-3.589791	1.450295	2.755504
H	-2.600111	1.400220	0.496432
H	3.683375	3.678721	-0.873818
H	4.002761	2.031954	-0.278650
H	3.528029	2.278699	-1.961337
H	0.703477	4.409523	-1.691555
H	0.566055	2.928943	-2.669567
H	-0.597340	3.245256	-1.382197
H	0.302648	3.157725	1.590474
H	1.994402	2.848256	1.981328
H	4.097659	-4.211329	-0.306897
H	3.198205	-3.639750	1.118837
H	2.458280	-3.539492	-0.485403
H	6.038398	-2.695452	0.276090
H	5.761014	-0.958629	0.535964
H	5.150316	-2.126943	1.713609
H	4.976103	-2.533714	-2.020605
H	3.330512	-1.866812	-2.186988
H	4.673077	-0.797480	-1.773935

(E)-7d

E(ωB97X-D/6-311+G**)= -1524.113499

C	2.205492	0.995501	-1.129655
C	1.362554	0.893781	-0.021065
C	1.668187	1.644417	1.114949
C	2.776281	2.483973	1.140943
C	3.600423	2.585905	0.026449
C	3.309351	1.835052	-1.109260
C	0.169470	0.003637	-0.038926
C	-1.083490	0.617394	-0.001971
Si	-1.460792	2.436675	-0.147168
C	-3.191853	2.620640	-0.885478
P	0.367287	-1.759254	-0.166174
C	2.197882	-1.977856	0.001842
C	2.941963	-1.550013	1.113149
C	4.306115	-1.786358	1.215431

C	4.975241	-2.496058	0.221516
C	4.254973	-2.961655	-0.872848
C	2.892256	-2.700620	-0.978885
C	-2.234748	-0.211389	0.057489
C	-3.220104	-0.913529	0.108235
C	-4.354901	-1.844668	0.127129
C	-4.486867	-2.521628	-1.250537
C	-4.109269	-2.922584	1.199081
C	-5.652777	-1.083276	0.449012
C	-1.516996	3.331082	1.523553
C	-0.290326	3.415971	-1.262140
H	2.346939	-3.048370	-1.851713
H	4.759081	-3.518473	-1.657991
H	6.041192	-2.684686	0.302656
H	4.850042	-1.420695	2.081616
H	2.435721	-1.015926	1.909759
H	1.980692	0.398659	-2.006350
H	3.950403	1.901771	-1.982445
H	4.466204	3.240079	0.043387
H	2.998683	3.055546	2.036801
H	1.027357	1.552189	1.985288
H	-0.715577	4.409728	-1.447114
H	0.700020	3.545303	-0.818715
H	-0.160864	2.914889	-2.225920
H	-3.445455	3.679038	-1.014521
H	-3.251870	2.127567	-1.860508
H	-3.943007	2.157498	-0.239550
H	-1.947299	4.333920	1.416700
H	-2.134226	2.768887	2.231647
H	-0.517318	3.435736	1.954660
H	-6.505646	-1.772239	0.466225
H	-5.579134	-0.595145	1.424557
H	-5.842873	-0.312468	-0.303074
H	-5.316208	-3.239797	-1.249398
H	-4.670774	-1.774527	-2.027253
H	-3.562942	-3.048141	-1.500570
H	-4.929658	-3.650683	1.206947
H	-3.170603	-3.444830	1.000463
H	-4.034231	-2.465775	2.189295

TS-5d

E(ωB97X-D/6-311+G**)= -1524.076065

C	-1.746763	3.256084	-0.684408
C	-0.791315	2.312901	-0.268652
C	-0.194713	2.515546	0.986325
C	-0.527254	3.609369	1.774449
C	-1.481538	4.529079	1.347304
C	-2.094108	4.339144	0.111180
P	-0.474145	0.869079	-1.342455
C	0.926507	0.022427	-0.528193
C	0.352978	-1.136167	-0.104033
C	-1.089152	-0.980579	-0.250992
C	-2.184068	-1.550304	0.095450
C	-3.629088	-1.229808	-0.146708
C	-4.183441	-2.257283	-1.149653
C	2.326646	0.446590	-0.432307

C	3.052972	0.250285	0.749012
C	4.373124	0.666428	0.860463
C	4.997879	1.304339	-0.205445
C	4.281880	1.529839	-1.377934
C	2.961976	1.114331	-1.487133
Si	1.038569	-2.817097	0.398741
C	0.695271	-3.107990	2.226913
C	0.165493	-4.139416	-0.614024
C	2.891064	-2.987786	0.065472
C	-4.401011	-1.368151	1.177002
C	-3.854714	0.186894	-0.703160
H	-2.224657	3.128265	-1.651947
H	-2.842564	5.044882	-0.237596
H	-1.746004	5.378311	1.969151
H	-0.044268	3.739393	2.738715
H	0.529993	1.794810	1.349571
H	0.511744	-5.143065	-0.341651
H	-0.910832	-4.062663	-0.441381
H	0.346769	-3.989945	-1.683083
H	0.959173	-4.126921	2.530856
H	1.256138	-2.407634	2.854134
H	-0.372164	-2.950855	2.407395
H	3.140046	-2.622944	-0.935776
H	3.506064	-2.431455	0.775927
H	3.174229	-4.045190	0.122740
H	2.558595	-0.221412	1.592181
H	4.912010	0.502215	1.788403
H	6.027691	1.635201	-0.118741
H	4.755241	2.036749	-2.212996
H	2.399301	1.299546	-2.395911
H	-5.255937	-2.094124	-1.317491
H	-3.664848	-2.171214	-2.108893
H	-4.037703	-3.274853	-0.775001
H	-4.923867	0.370798	-0.872027
H	-3.478482	0.941347	-0.007106
H	-3.326910	0.323158	-1.650822
H	-5.472813	-1.180923	1.027437
H	-4.269887	-2.372545	1.590326
H	-4.025590	-0.650387	1.912462

8d

E(ωB97X-D/6-311+G**)= -1524.079092

C	-0.801106	1.885845	1.216139
C	-0.943145	2.073477	-0.163020
C	-1.514000	3.266035	-0.610052
C	-1.934162	4.245005	0.288149
C	-1.779393	4.046504	1.653507
C	-1.209175	2.860719	2.114321
P	-0.461137	0.767508	-1.388377
C	-0.980769	-0.878449	-0.499314
C	-2.053074	-1.572827	-0.120803
C	-3.476650	-1.122476	-0.399886
C	-3.662633	-0.077256	-1.516998
C	1.036637	0.042769	-0.609950
C	0.441843	-1.112596	-0.193238
Si	1.047954	-2.755309	0.498246

C	2.930689	-2.931135	0.404417
C	2.398757	0.551907	-0.498701
C	3.005858	1.247290	-1.554450
C	4.295381	1.746966	-1.436303
C	5.013357	1.574715	-0.256001
C	4.417564	0.906734	0.808611
C	3.126007	0.411375	0.690814
C	0.497893	-2.902777	2.291286
C	0.303763	-4.151996	-0.513674
C	-4.310125	-2.363661	-0.763140
C	-4.026588	-0.530950	0.911205
H	-1.634968	3.425748	-1.677520
H	-2.380835	5.162617	-0.082367
H	-2.104316	4.805759	2.357845
H	-1.092498	2.693814	3.180617
H	-0.383746	0.950725	1.577381
H	0.609900	-5.131164	-0.127360
H	-0.784231	-4.056384	-0.468970
H	0.611700	-4.081274	-1.561779
H	0.764197	-3.873901	2.723316
H	0.943938	-2.117713	2.911177
H	-0.589367	-2.783457	2.312015
H	3.204470	-3.980197	0.566696
H	3.306214	-2.631756	-0.579021
H	3.453089	-2.327582	1.150396
H	2.652523	-0.078142	1.534847
H	4.956664	0.781931	1.742583
H	6.019716	1.969475	-0.162654
H	4.744934	2.274012	-2.272189
H	2.449224	1.384613	-2.475692
H	-5.369865	-2.108604	-0.905820
H	-3.937736	-2.814288	-1.689440
H	-4.225245	-3.114578	0.027297
H	-4.729146	0.116339	-1.694968
H	-3.200330	0.877863	-1.254955
H	-3.216490	-0.422948	-2.454489
H	-5.088422	-0.269723	0.805242
H	-3.921564	-1.254021	1.725442
H	-3.473686	0.373232	1.185065

TS-4d

E(ωB97X-D/6-311+G**)= -1524.080363

C	0.653361	-2.464590	0.753128
C	0.534717	-1.621800	-0.355851
C	1.126614	-2.031137	-1.555015
C	1.780505	-3.251980	-1.649486
C	1.869441	-4.090899	-0.541965
C	1.307784	-3.687188	0.663442
C	-0.209771	-0.345909	-0.250972
C	-1.560500	-0.309986	0.063457
Si	-2.728935	-1.758582	0.244977
C	-4.483517	-1.157393	-0.095221
P	0.555070	1.225692	-0.609071
C	2.287727	1.009213	-0.016758
C	3.328900	1.630486	-0.723053
C	4.645368	1.578742	-0.279394

C	4.966101	0.889411	0.885890
C	3.947634	0.270104	1.606098
C	2.631737	0.343262	1.170108
C	-2.092250	1.027448	0.144280
C	-1.448450	2.121713	0.120037
C	-1.557221	3.601588	-0.059479
C	-2.900700	4.045454	0.547368
C	-0.417725	4.336728	0.659387
C	-1.538311	3.967101	-1.554231
C	-2.369143	-3.165713	-0.965125
C	-2.754607	-2.496151	1.991487
H	3.094406	2.152707	-1.646655
H	5.427330	2.068001	-0.853340
H	5.994071	0.837044	1.230411
H	4.179760	-0.264445	2.522735
H	1.847024	-0.116116	1.760473
H	-4.561972	-0.749572	-1.107224
H	-5.212221	-1.969280	0.014311
H	-4.744051	-0.350696	0.595779
H	-3.589214	-3.197786	2.104633
H	-1.830043	-3.036602	2.212662
H	-2.871544	-1.704727	2.738703
H	-3.174086	-3.909194	-0.931258
H	-2.306081	-2.777923	-1.986650
H	-1.425663	-3.671919	-0.745698
H	0.225026	-2.142080	1.695548
H	1.382239	-4.324189	1.539393
H	2.379775	-5.045625	-0.617371
H	2.224226	-3.552263	-2.593547
H	1.055704	-1.374546	-2.414968
H	-1.649284	5.051029	-1.687183
H	-0.599312	3.646464	-2.010382
H	-2.356898	3.462122	-2.074615
H	-3.030233	5.128050	0.423582
H	-3.727757	3.526595	0.057758
H	-2.939462	3.805232	1.613052
H	-0.534964	5.421799	0.548214
H	-0.412805	4.090252	1.724831
H	0.548902	4.041409	0.244384

9d

E(ωB97X-D/6-311+G**)= -1524.105980

C	2.335067	1.422724	0.510220
C	1.700918	0.955828	-0.646827
C	2.080185	1.518564	-1.872155
C	3.078094	2.481953	-1.939837
C	3.714802	2.918105	-0.781487
C	3.333974	2.385517	0.445076
C	0.635124	-0.053567	-0.551680
C	0.672302	-1.293208	0.012931
Si	2.150483	-2.278359	0.612141
C	1.991721	-2.548961	2.471828
C	-0.612248	-2.041175	0.118832
C	-1.626677	-1.286821	-0.379619
C	-3.081292	-1.739355	-0.520468
C	-3.242811	-2.503762	-1.847282

P	-1.043265	0.268594	-1.171440
C	-1.525758	1.607776	0.018341
C	-1.972874	2.842365	-0.446681
C	-2.332525	3.854538	0.442168
C	-2.245494	3.640095	1.810762
C	-1.796592	2.408627	2.287167
C	-1.442984	1.403389	1.399431
C	-3.445118	-2.675813	0.638026
C	-4.053796	-0.549798	-0.513766
C	2.134523	-3.952125	-0.251911
C	3.845552	-1.508095	0.263393
H	-2.038729	3.012842	-1.516894
H	-2.678860	4.810564	0.061641
H	-2.524923	4.425476	2.506031
H	-1.726683	2.233683	3.356393
H	-1.106972	0.434751	1.760130
H	2.908309	-4.618617	0.146521
H	1.151024	-4.408131	-0.115401
H	2.304453	-3.832528	-1.327051
H	2.756084	-3.236305	2.851223
H	2.083698	-1.602087	3.014468
H	1.002838	-2.966361	2.681545
H	4.627857	-2.263183	0.404961
H	3.908629	-1.142704	-0.766526
H	4.066446	-0.662731	0.919350
H	2.022851	1.020007	1.467922
H	3.811817	2.727297	1.357911
H	4.493519	3.671963	-0.834578
H	3.361826	2.895105	-2.902882
H	1.585394	1.180032	-2.776149
H	-4.275260	-2.855596	-1.980680
H	-2.985476	-1.861633	-2.696208
H	-2.569006	-3.364618	-1.857961
H	-4.465597	-3.062535	0.513875
H	-2.734984	-3.502889	0.684507
H	-3.385355	-2.142244	1.591925
H	-5.088960	-0.900464	-0.608689
H	-3.969120	0.023438	0.414173
H	-3.857167	0.134769	-1.346691

1d' + PhTMSP$^-$

E(ωB97X-D/6-311+G**)= -1524.063561

C	0.345287	-1.293690	-1.375407
C	-0.891516	-0.763909	-1.802798
C	-0.805853	0.229838	-2.805296
C	0.403146	0.658531	-3.330174
C	1.607056	0.115529	-2.882723
C	1.557892	-0.866245	-1.898049
P	-2.554625	-1.256122	-1.229988
Si	-2.161799	-2.318830	0.665382
C	-1.412284	-4.068979	0.554388
C	-3.855434	-2.575761	1.488578
C	-1.097159	-1.394550	1.940421
C	-1.359956	2.239139	0.720573
C	-2.767538	2.635784	0.613851
C	-3.169256	2.637269	-0.871921

C	-0.202164	1.909360	0.792515
C	1.100656	1.498558	0.848851
C	2.246096	1.118685	0.881604
C	3.586097	0.638219	0.830214
C	4.190152	0.050287	1.948223
C	5.489707	-0.431350	1.870290
C	6.204711	-0.334432	0.681140
C	5.610864	0.248695	-0.434013
C	4.312918	0.732240	-0.364141
C	-3.635688	1.619050	1.375647
C	-2.937805	4.041819	1.216757
H	6.158207	0.319754	-1.367740
H	7.217267	-0.718726	0.621195
H	-1.727044	0.673767	-3.173339
H	0.407767	1.428613	-4.097462
H	2.554700	0.446224	-3.297156
H	2.479022	-1.304710	-1.522735
H	0.362886	-2.068449	-0.617557
H	3.626456	-0.031819	2.870172
H	5.944090	-0.890510	2.741689
H	3.832814	1.168360	-1.231808
H	-0.962589	-1.994929	2.849116
H	-0.107624	-1.150767	1.542671
H	-1.571663	-0.448898	2.217627
H	-1.320305	-4.527928	1.547245
H	-2.053530	-4.705660	-0.063736
H	-0.420291	-4.062691	0.092348
H	-3.751105	-3.118224	2.435750
H	-4.340436	-1.616835	1.696356
H	-4.520354	-3.151747	0.836913
H	-4.688313	1.914138	1.306327
H	-3.518458	0.629757	0.925251
H	-3.353630	1.573048	2.431633
H	-4.212727	2.957576	-0.966991
H	-2.537503	3.320439	-1.446470
H	-3.073256	1.627592	-1.280469
H	-3.985453	4.349677	1.135873
H	-2.653826	4.052450	2.272828
H	-2.320122	4.771413	0.685780

TS-1d'

E(ωB97X-D/6-311+G**)= -1524.052151

C	0.360449	-1.627115	-0.423984
C	-0.915817	-1.666047	-1.007715
C	-0.986359	-2.168059	-2.322949
C	0.139669	-2.601561	-3.004515
C	1.395260	-2.556506	-2.397361
C	1.493170	-2.065968	-1.102080
P	-2.446895	-1.018378	-0.262146
Si	-2.053266	-1.068533	1.928940
C	-1.417762	-2.751662	2.526347
C	-3.726106	-0.780242	2.775026
C	-0.893779	0.241005	2.661437
C	-1.545573	1.499540	-0.611950
C	-2.799565	2.226006	-0.978100
C	-3.352025	1.702050	-2.311601

C	-0.322359	1.597894	-0.459210
C	0.998325	1.447788	-0.215409
C	2.192654	1.345901	0.008740
C	3.557832	1.060428	0.235785
C	4.171971	1.336303	1.471229
C	5.509031	1.034671	1.681166
C	6.273772	0.455419	0.671910
C	5.677272	0.177006	-0.555591
C	4.340622	0.470911	-0.775504
C	-3.866470	2.087691	0.115737
C	-2.433815	3.715590	-1.132512
H	6.259061	-0.280918	-1.349280
H	7.318913	0.220185	0.840975
H	-1.956717	-2.214005	-2.809460
H	0.040340	-2.979963	-4.018252
H	2.279098	-2.893843	-2.929560
H	2.461390	-1.999469	-0.614423
H	0.485191	-1.223181	0.572610
H	3.579796	1.785422	2.260346
H	5.959395	1.252730	2.644404
H	3.872762	0.239597	-1.725503
H	-0.844606	0.132178	3.752136
H	0.123898	0.176961	2.266817
H	-1.260450	1.244599	2.427610
H	-1.245829	-2.746231	3.609511
H	-2.146419	-3.534095	2.294293
H	-0.479101	-3.018302	2.032427
H	-3.622099	-0.845846	3.864356
H	-4.124681	0.210402	2.535169
H	-4.460508	-1.524766	2.453837
H	-4.736413	2.705348	-0.134809
H	-4.187954	1.049002	0.206439
H	-3.475048	2.415513	1.083631
H	-4.252722	2.262776	-2.587687
H	-2.609907	1.819120	-3.106506
H	-3.601803	0.643210	-2.224214
H	-3.320753	4.293182	-1.418534
H	-2.041871	4.114684	-0.193463
H	-1.666202	3.844578	-1.899219

TS-2d’

E(ωB97X-D/6-311+G**)= -1524.078013

C	-2.754046	-0.858733	1.388048
C	-3.226585	-0.303940	0.194430
C	-4.600749	-0.072744	0.076962
C	-5.473005	-0.376485	1.116205
C	-4.988858	-0.937231	2.293517
C	-3.624712	-1.178815	2.422920
P	-2.101089	0.143710	-1.208983
C	-0.515805	-0.700517	-0.823372
C	-0.303410	-2.198935	-1.143537
C	0.378297	-2.894140	0.045265
Si	-1.401172	2.178176	-0.435453
C	-0.937520	2.323402	1.386890
C	-0.217803	3.083181	-1.592728
C	-3.025593	3.187387	-0.551330

C	0.440356	0.157271	-0.437608
C	1.779330	0.022236	-0.197117
C	2.982366	0.077733	0.058389
C	4.356730	0.101141	0.351136
C	5.320230	0.362407	-0.648333
C	6.673536	0.379868	-0.351683
C	7.127207	0.140269	0.944009
C	6.189879	-0.117893	1.942241
C	4.833801	-0.138300	1.659293
C	-1.620742	-2.931498	-1.438620
C	0.604454	-2.308028	-2.380464
H	7.387443	0.584265	-1.144262
H	8.187796	0.155394	1.170191
H	-4.988438	0.348728	-0.845933
H	-6.535593	-0.185221	1.001475
H	-5.669186	-1.185824	3.101700
H	-3.233854	-1.611446	3.338761
H	-1.687010	-1.025329	1.498318
H	4.113963	-0.340029	2.444585
H	6.521393	-0.305878	2.959307
H	4.979962	0.549924	-1.660578
H	-1.167657	3.332602	1.750353
H	-1.522396	1.614823	1.981797
H	0.120225	2.110414	1.543010
H	-2.846999	4.230984	-0.264752
H	-3.436212	3.185117	-1.567029
H	-3.792322	2.785530	0.120155
H	-0.343907	4.167870	-1.490722
H	0.816315	2.813887	-1.374059
H	-0.425220	2.817688	-2.634372
H	0.575542	-3.947615	-0.189742
H	1.326652	-2.412721	0.291858
H	-0.263397	-2.860818	0.931633
H	0.794068	-3.359550	-2.632523
H	0.133080	-1.826671	-3.243185
H	1.563235	-1.816908	-2.196709
H	-1.420173	-3.986534	-1.660239
H	-2.303823	-2.892590	-0.584921
H	-2.134892	-2.496013	-2.300100

6d’

E(ωB97X-D/6-311+G**)= -1524.080007

C	2.165660	0.209098	1.835251
C	2.871716	0.037489	0.641458
C	4.213388	-0.349005	0.716904
C	4.829457	-0.569223	1.944127
C	4.116667	-0.389401	3.124212
C	2.783128	0.003516	3.062785
P	2.102953	0.272256	-1.029367
Si	1.354930	-1.860630	-1.294764
C	2.908676	-2.830607	-1.775083
C	0.553162	1.224130	-0.676060
C	0.653611	2.758445	-0.826872
C	0.854244	3.112390	-2.310646
C	-0.576968	0.598644	-0.399702
C	-1.654015	-0.056885	-0.137459

C	-2.651848	-0.823846	0.111559
C	-4.028139	-0.566047	0.387086
C	-4.495371	-0.376234	1.707620
C	-5.840008	-0.174983	1.975827
C	-6.784988	-0.162225	0.951135
C	-6.343512	-0.355564	-0.357015
C	-5.001560	-0.559072	-0.637547
C	0.641110	-2.653410	0.246656
C	0.132554	-1.915293	-2.719529
C	-0.630944	3.425531	-0.328035
C	1.839562	3.306554	-0.015734
H	-6.156947	-0.023678	3.004360
H	-7.836987	-0.007277	1.165405
H	4.780433	-0.473154	-0.201368
H	5.871642	-0.871484	1.978355
H	4.595969	-0.552714	4.083941
H	2.216136	0.141434	3.977993
H	1.119673	0.494568	1.789356
H	-4.678059	-0.716099	-1.661308
H	-7.060106	-0.348171	-1.174082
H	-3.774302	-0.388526	2.518250
H	0.530102	-3.731861	0.083878
H	1.305007	-2.505859	1.103654
H	-0.343530	-2.240230	0.482261
H	2.660030	-3.882521	-1.955224
H	3.369266	-2.427405	-2.681926
H	3.653318	-2.797529	-0.973263
H	-0.074232	-2.954654	-2.997512
H	-0.808515	-1.447939	-2.419873
H	0.521841	-1.395721	-3.600317
H	0.940405	4.198048	-2.443291
H	1.761898	2.646947	-2.705537
H	0.004577	2.757397	-2.901387
H	1.910225	4.394602	-0.131337
H	1.721949	3.080441	1.048010
H	2.787691	2.872768	-0.347621
H	-0.559054	4.513224	-0.446687
H	-1.501564	3.066487	-0.881085
H	-0.803654	3.199876	0.727907

(Z)-7d’

E(ωB97X-D/6-311+G**)= -1524.103296

C	-3.527318	-0.391280	1.174589
C	-3.664571	-0.177459	-0.209703
C	-4.954201	-0.336733	-0.746296
C	-6.034238	-0.708420	0.043517
C	-5.866754	-0.943810	1.406175
C	-4.601028	-0.781087	1.963791
P	-2.301606	0.444922	-1.257471
C	-0.820300	-0.306769	-0.555133
C	-0.611129	-1.842752	-0.438560
C	-0.014603	-2.215796	0.931354
C	0.235471	0.576218	-0.347013
Si	-0.032265	2.425913	-0.070515
C	1.605983	3.130822	0.585433
C	1.588565	0.188647	-0.205608

C	2.772962	-0.044093	-0.066187
C	4.144895	-0.359000	0.107140
C	5.076175	0.632255	0.461235
C	6.416621	0.318688	0.630237
C	6.866425	-0.986923	0.451988
C	5.953999	-1.978253	0.101796
C	4.611791	-1.673079	-0.069057
C	-0.396401	3.465757	-1.603529
C	-1.291319	2.758921	1.294661
C	0.333642	-2.282286	-1.579855
C	-1.891836	-2.674633	-0.615399
H	7.118008	1.100009	0.905090
H	7.915464	-1.228030	0.585541
H	-5.100628	-0.177634	-1.811220
H	-7.014203	-0.828732	-0.410239
H	-6.708084	-1.242775	2.023110
H	-4.451243	-0.947891	3.026867
H	-2.553018	-0.242894	1.630688
H	3.901528	-2.445432	-0.342072
H	6.291734	-2.999927	-0.040282
H	4.724796	1.648212	0.602420
H	-1.396859	3.838832	1.451035
H	-2.271337	2.344886	1.053158
H	-0.945895	2.315171	2.234290
H	1.476462	4.190351	0.835381
H	1.943063	2.603610	1.483162
H	2.401429	3.052551	-0.162180
H	-0.264039	4.531493	-1.379937
H	0.297718	3.200945	-2.408298
H	-1.411962	3.294932	-1.963169
H	0.503597	-3.364460	-1.520906
H	-0.131658	-2.059604	-2.544612
H	1.299181	-1.779930	-1.539158
H	-0.701295	-1.921779	1.731092
H	0.127586	-3.301471	0.993091
H	0.947038	-1.736106	1.113207
H	-1.615993	-3.734840	-0.658685
H	-2.592543	-2.545464	0.209157
H	-2.410038	-2.414235	-1.540698

(E)-7d’

E(ωB97X-D/6-311+G**)= -1524.085946

C	2.904408	-2.538061	0.599926
C	2.720493	-1.606737	-0.432310
C	3.761381	-1.471913	-1.358553
C	4.947618	-2.189706	-1.236844
C	5.118179	-3.083987	-0.186764
C	4.085325	-3.258987	0.730206
P	1.064445	-0.804524	-0.733040
C	0.939073	0.703581	0.150114
C	2.105168	1.492241	0.799425
C	2.775416	0.617625	1.879309
C	-0.401097	1.216822	0.187360
Si	-1.026920	2.911835	-0.297283
C	-2.644618	2.699380	-1.253460
C	-1.466423	0.305878	0.178357

C	-2.456228	-0.407369	0.246333
C	-3.515450	-1.335307	0.184509
C	-4.838738	-0.937710	-0.096640
C	-5.866075	-1.866551	-0.160902
C	-5.617832	-3.220548	0.052986
C	-4.314502	-3.629354	0.330614
C	-3.280328	-2.709680	0.398761
C	-1.479730	4.096564	1.113808
C	0.124090	3.807652	-1.512644
C	1.640072	2.756524	1.536461
C	3.153669	1.904495	-0.246745
H	-6.874956	-1.529808	-0.381093
H	-6.424041	-3.944645	0.003326
H	3.634582	-0.786560	-2.191362
H	5.738639	-2.052796	-1.968261
H	6.041859	-3.645263	-0.087563
H	4.198723	-3.965473	1.547171
H	2.103808	-2.684286	1.318538
H	-2.266370	-3.030580	0.608924
H	-4.102333	-4.681565	0.495504
H	-5.040342	0.113810	-0.269529
H	-0.417708	4.633210	-1.987968
H	1.030458	4.221964	-1.065087
H	0.431077	3.108373	-2.297156
H	-2.964176	3.659575	-1.675233
H	-2.526657	1.979166	-2.067867
H	-3.437077	2.324409	-0.600833
H	-2.002006	4.973995	0.714786
H	-2.160260	3.587177	1.804098
H	-0.619452	4.445735	1.688937
H	2.487148	3.182567	2.085468
H	1.285090	3.529724	0.858128
H	0.843187	2.528623	2.247466
H	3.296808	-0.236066	1.455857
H	3.506720	1.217313	2.434331
H	2.027620	0.248120	2.587286
H	3.941569	2.505529	0.224274
H	3.620780	1.032342	-0.703364
H	2.694284	2.498477	-1.041068

TS-5d’

E(ωB97X-D/6-311+G**)= -1524.077226

C	-1.985263	2.949419	-0.809284
C	-0.927104	2.158918	-0.326672
C	-0.628971	2.265193	1.043263
C	-1.330138	3.128583	1.874837
C	-2.372807	3.903849	1.374251
C	-2.698696	3.799928	0.023220
P	-0.118896	0.957453	-1.444131
C	1.470076	0.614762	-0.594066
C	2.675656	1.550348	-0.521516
C	2.279247	3.009126	-0.799514
C	1.397965	-0.692004	-0.206021
Si	2.542902	-2.039702	0.466824
C	2.019126	-3.702961	-0.239173
C	0.012531	-1.112312	-0.285012

C	-0.918910	-1.873880	0.083359
C	-2.336993	-1.973495	-0.024234
C	-2.981641	-3.160048	-0.421378
C	-4.362609	-3.224469	-0.528756
C	-5.150706	-2.115045	-0.225554
C	-4.529177	-0.934893	0.174450
C	-3.147035	-0.857011	0.271670
C	4.394280	-1.924010	0.077140
C	2.329789	-2.073598	2.338563
C	3.316021	1.505604	0.875853
C	3.693795	1.136223	-1.599995
H	-5.126094	-0.057341	0.405053
H	-6.231700	-2.171016	-0.303555
H	-2.246604	2.885815	-1.862027
H	-3.512553	4.392683	-0.384859
H	-2.928517	4.569823	2.026286
H	-1.073481	3.183736	2.929054
H	0.151610	1.633961	1.457850
H	2.664532	-4.502518	0.142561
H	0.981457	-3.911955	0.028070
H	2.087551	-3.698870	-1.331618
H	2.860267	-2.918534	2.790975
H	2.694641	-1.150656	2.799534
H	1.264321	-2.169222	2.567682
H	4.882629	-2.777029	0.563408
H	4.574629	-2.016670	-0.998239
H	4.887802	-1.015481	0.427027
H	-2.374667	-4.030549	-0.648265
H	-4.832119	-4.150945	-0.847097
H	-2.668144	0.069536	0.567594
H	4.201565	2.151470	0.912973
H	2.604132	1.859866	1.626915
H	3.622362	0.499573	1.161393
H	4.565377	1.802603	-1.582188
H	4.039279	0.112980	-1.456489
H	3.230152	1.201514	-2.588424
H	3.173966	3.642714	-0.778287
H	1.804271	3.106141	-1.778574
H	1.575530	3.383033	-0.052929

8d’

E(ωB97X-D/6-311+G**)= -1524.086465

C	-3.121172	-0.957391	0.602254
C	-2.250282	-1.803467	-0.121534
C	-2.847606	-2.639483	-1.091489
C	-4.213664	-2.620215	-1.326593
C	-5.055565	-1.782019	-0.595835
C	-4.488643	-0.951801	0.369323
C	-0.836520	-1.848246	0.148025
C	-0.017616	-0.904317	-0.297481
C	1.445087	-0.643278	-0.136693
Si	2.432011	-1.990062	0.748546
C	2.112767	-3.617238	-0.137126
P	-0.199971	0.694765	-1.299663
C	-0.948822	1.862872	-0.063715
C	-1.870442	2.815961	-0.494100

C	-2.467396	3.696506	0.405863
C	-2.146501	3.633890	1.755011
C	-1.228297	2.683132	2.199250
C	-0.640182	1.807220	1.299118
C	1.555410	0.584849	-0.716094
C	2.667097	1.569170	-0.991264
C	2.181477	2.989909	-0.654059
C	3.925821	1.275050	-0.175230
C	3.018873	1.511922	-2.490054
C	4.317304	-1.839006	0.875197
C	1.775601	-2.069612	2.509382
H	-5.121148	-0.280051	0.943967
H	-6.125556	-1.773899	-0.777113
H	-2.128080	2.863159	-1.547961
H	-3.187459	4.427340	0.050916
H	-2.611106	4.315678	2.460383
H	-0.982384	2.618498	3.254773
H	0.057099	1.051106	1.647976
H	2.618656	-4.447357	0.369464
H	1.034649	-3.795867	-0.148242
H	2.468827	-3.577600	-1.171919
H	2.237017	-2.887834	3.073670
H	1.966226	-1.132800	3.043945
H	0.694932	-2.229713	2.457059
H	4.674368	-2.740093	1.388363
H	4.806404	-1.806283	-0.102961
H	4.646859	-0.973838	1.455013
H	-2.207841	-3.306663	-1.661054
H	-4.630880	-3.271654	-2.090545
H	-2.698197	-0.292758	1.348587
H	4.669633	2.065870	-0.326450
H	3.696941	1.219095	0.893005
H	4.376126	0.329910	-0.477035
H	3.797628	2.245662	-2.731871
H	3.382545	0.516058	-2.759585
H	2.138620	1.726769	-3.101898
H	2.964405	3.722173	-0.883628
H	1.289291	3.251958	-1.229564
H	1.923474	3.076069	0.405074

TS-4d’

E(ωB97X-D/6-311+G**)= -1524.059969

C	2.762828	2.445185	0.477199
C	2.219147	1.553321	-0.461292
C	3.068941	1.009976	-1.430298
C	4.417618	1.338463	-1.453492
C	4.946921	2.223364	-0.520514
C	4.108643	2.776037	0.444350
C	0.823323	1.224580	-0.396452
C	-0.316060	1.325718	0.100126
C	-1.528460	0.557253	0.012217
Si	-3.007173	1.645977	0.468272
C	-3.684396	1.377249	2.213624
C	-1.365116	-0.710852	-0.511375
C	-2.489369	-1.765137	-0.616798
C	-3.122462	-1.679399	-2.017032

P	0.228920	-1.144356	-1.355268
C	1.301507	-1.681076	0.007667
C	0.996281	-1.590248	1.379115
C	1.888321	-2.016413	2.352650
C	3.127531	-2.550398	2.006631
C	3.451361	-2.650023	0.654839
C	2.557874	-2.231479	-0.318706
C	-1.926170	-3.187839	-0.416618
C	-3.572633	-1.598267	0.457481
C	-2.423921	3.439529	0.425712
C	-4.429159	1.622643	-0.789194
H	5.061420	0.896403	-2.207296
H	6.002066	2.476185	-0.540979
H	2.828376	-2.333902	-1.366640
H	4.411639	-3.061732	0.355903
H	3.825773	-2.876927	2.770339
H	1.616412	-1.918455	3.400476
H	0.049126	-1.152073	1.677713
H	2.106052	2.862012	1.232120
H	4.508673	3.464324	1.182577
H	2.645325	0.320802	-2.151427
H	-3.274317	4.114558	0.581774
H	-1.674034	3.631438	1.196203
H	-1.963013	3.676091	-0.537485
H	-2.860869	1.431136	2.932809
H	-4.399276	2.169542	2.462948
H	-4.181839	0.414573	2.345894
H	-5.120044	2.440988	-0.556388
H	-4.024032	1.805536	-1.789857
H	-5.009148	0.699146	-0.831612
H	-2.748556	-3.915033	-0.420849
H	-1.214106	-3.445470	-1.200064
H	-1.402933	-3.266459	0.541174
H	-3.897053	-2.447491	-2.135859
H	-3.576538	-0.699101	-2.182751
H	-2.359024	-1.837864	-2.783821
H	-4.274208	-2.438501	0.406881
H	-3.124244	-1.588824	1.454863
H	-4.160305	-0.692351	0.337532

9d’

E(ωB97X-D/6-311+G**)= -1524.102888

C	-2.030553	2.889745	-0.374835
C	-1.248473	1.817940	0.051603
C	-1.015835	1.664141	1.421213
C	-1.538172	2.568458	2.334539
C	-2.312578	3.641584	1.896207
C	-2.560713	3.798409	0.539233
P	-0.554876	0.608333	-1.174999
C	1.192401	0.539963	-0.642994
C	2.188254	1.660930	-0.945169
C	3.230489	1.156214	-1.958935
C	-0.875411	-1.010592	-0.398671
C	-2.216882	-1.613111	-0.383711
C	-2.423030	-2.863487	0.225629
C	-3.679097	-3.444395	0.272583

C	-4.785612	-2.799117	-0.281790
C	-4.602971	-1.560416	-0.883928
C	-3.340273	-0.978941	-0.934863
C	0.263938	-1.614959	0.047125
C	1.425012	-0.682497	-0.084890
Si	2.896129	-1.669352	0.558999
C	2.479977	-2.330049	2.274177
C	1.512100	2.898770	-1.559803
C	2.865915	2.119329	0.356525
C	3.189750	-3.092395	-0.643386
C	4.614443	-0.875773	0.806424
H	-5.449886	-1.039127	-1.321097
H	-5.769889	-3.254994	-0.240387
H	-2.218816	3.018177	-1.436746
H	-3.167804	4.627315	0.188459
H	-2.722403	4.347081	2.612099
H	-1.348743	2.433697	3.394869
H	-0.425826	0.815909	1.756802
H	-1.553867	-3.349822	0.655088
H	-3.802877	-4.412898	0.749699
H	-3.222980	-0.012843	-1.416835
H	3.932039	-3.799560	-0.255698
H	2.245042	-3.614820	-0.809869
H	3.548753	-2.715148	-1.607257
H	3.265687	-3.004319	2.634895
H	2.384179	-1.507094	2.991063
H	1.527835	-2.861250	2.233392
H	5.307666	-1.690565	1.049574
H	5.016042	-0.355912	-0.066705
H	4.633232	-0.179356	1.650393
H	2.271939	3.655054	-1.789744
H	0.986564	2.650926	-2.485948
H	0.792370	3.345676	-0.869586
H	3.992019	1.923019	-2.150348
H	3.728736	0.253714	-1.605779
H	2.740455	0.913021	-2.906512
H	3.595638	2.912822	0.153061
H	2.114026	2.509870	1.048826
H	3.382119	1.298846	0.849598

Anionic species in the presence of one Li^+

6a + Li^+

E(ωB97X-D/6-311+G**)= -1605.381987

C	2.562324	0.312708	1.510910
C	3.035859	-0.138107	0.276680
C	4.399160	-0.409690	0.138212
C	5.266752	-0.257244	1.213642
C	4.784871	0.190486	2.437970
C	3.432188	0.479779	2.581160
P	1.938812	-0.399372	-1.189544
Si	1.324105	-2.510614	-0.675867
C	2.843683	-3.600584	-0.790351
C	0.431963	0.574980	-0.751962

C	0.566177	2.056589	-0.711152
C	-0.406633	2.833689	-0.070181
C	-0.287479	4.213250	-0.003633
C	0.810258	4.852897	-0.571708
C	1.785340	4.094229	-1.205700
C	1.666585	2.711673	-1.271955
C	-0.731928	-0.025732	-0.535908
C	-1.935910	-0.402777	-0.299159
C	-2.989739	-1.134945	-0.037081
C	-4.332850	-0.620403	0.229116
C	-4.677064	0.726824	0.034512
C	-5.960547	1.181278	0.295018
C	-6.932955	0.300517	0.762449
C	-6.611854	-1.037283	0.960502
C	-5.327196	-1.491312	0.689435
C	0.611949	-2.680038	1.073837
C	0.046666	-3.114766	-1.945201
Li	-1.535835	-2.491458	-0.179065
H	-6.206199	2.225467	0.134075
H	-7.936743	0.656834	0.966284
H	4.786229	-0.736695	-0.822253
H	6.322293	-0.472694	1.088980
H	5.462518	0.322631	3.274053
H	3.050828	0.840646	3.530155
H	1.508366	0.540198	1.630993
H	-5.074245	-2.536230	0.834485
H	-7.365212	-1.729361	1.320846
H	-3.921592	1.414027	-0.333331
H	0.155444	-3.664747	1.243171
H	1.436124	-2.597138	1.787191
H	-0.095329	-1.892001	1.356755
H	2.587836	-4.645662	-0.589527
H	3.295798	-3.547478	-1.784315
H	3.595147	-3.289800	-0.059497
H	-0.415496	-4.069651	-1.660455
H	-0.734573	-2.381933	-2.182770
H	0.560093	-3.301081	-2.891999
H	2.437486	2.138818	-1.776082
H	2.647009	4.578011	-1.652371
H	0.906663	5.931244	-0.513880
H	-1.052813	4.792944	0.501141
H	-1.259490	2.341103	0.384099

(Z)-7a + Li$^+$

E(ωB97X-D/6-311+G**)= -1605.402722

C	5.083739	-0.070739	-0.323395
C	3.808624	-0.635315	-0.194933
C	3.689262	-2.030153	-0.105544
C	4.819880	-2.832752	-0.142332
C	6.083438	-2.263792	-0.269930
C	6.209562	-0.881600	-0.360273
C	2.634884	0.171196	-0.148151
C	1.582087	0.762219	-0.072580
C	0.334298	1.440950	0.001787
Si	0.457436	3.313497	0.227691
C	-0.281966	4.181405	-1.303424

C	-0.841305	0.670789	0.054295
P	-2.452611	1.423765	-0.120918
C	-3.605848	-0.006022	-0.106082
C	-4.415514	-0.276065	-1.212028
C	-5.310956	-1.340442	-1.201070
C	-5.408831	-2.157230	-0.081492
C	-4.621156	-1.889666	1.033417
C	-3.741378	-0.816362	1.026007
C	-0.689279	-0.810779	0.154844
C	-0.183131	-1.366541	1.329504
C	-0.036647	-2.741031	1.453651
C	-0.380704	-3.578490	0.397759
C	-0.876832	-3.031586	-0.779390
C	-1.036134	-1.657097	-0.896054
C	-0.438671	3.910166	1.758135
C	2.269204	3.782023	0.298294
Li	-0.819952	1.859273	-1.840327
H	7.191000	-0.431226	-0.460161
H	6.965041	-2.894278	-0.297784
H	-4.336150	0.347235	-2.097592
H	-5.925852	-1.536206	-2.073125
H	-6.100162	-2.992647	-0.074420
H	-4.695674	-2.516910	1.915067
H	-3.137629	-0.613871	1.903769
H	2.701552	-2.466975	-0.003402
H	4.713903	-3.909575	-0.069174
H	5.181961	1.006655	-0.392531
H	-1.454027	-1.239416	-1.806098
H	-1.152094	-3.676956	-1.606000
H	-0.263864	-4.652137	0.493795
H	0.353970	-3.159384	2.374718
H	0.101492	-0.710124	2.144387
H	-0.353107	4.997047	1.855434
H	-1.498047	3.646375	1.731111
H	0.002155	3.455353	2.649794
H	2.380247	4.863949	0.420414
H	2.762268	3.289959	1.140827
H	2.796845	3.480132	-0.610326
H	-0.140668	5.261615	-1.208707
H	0.234385	3.900977	-2.234091
H	-1.362375	4.028658	-1.414915

(E)-7a + Li^+

E(ωB97X-D/6-311+G**)= -1605.412403

C	-4.193110	-1.680491	0.949042
C	-4.107108	-0.495071	0.194749
C	-5.241889	-0.067604	-0.515377
C	-6.412182	-0.806153	-0.472407
C	-6.479404	-1.991375	0.258701
C	-5.367202	-2.425540	0.966598
C	-2.863799	0.198967	0.113394
C	-1.724514	0.623634	-0.005819
C	-0.424737	1.155710	-0.072570
Si	-0.342895	3.045965	-0.114392
C	1.085998	3.672472	-1.160468
C	0.638169	0.271720	-0.231405

P	0.366482	-1.388085	-0.805816
C	1.826124	-2.368116	-0.300237
C	2.356387	-3.296925	-1.201731
C	3.448668	-4.085990	-0.859015
C	4.045364	-3.948551	0.388223
C	3.529690	-3.028443	1.295425
C	2.423983	-2.258819	0.960806
C	2.022886	0.767735	-0.022956
C	2.995658	0.657552	-1.017565
C	4.275578	1.149146	-0.806974
C	4.609133	1.740583	0.407533
C	3.654416	1.832143	1.413227
C	2.370434	1.348119	1.197744
C	-1.955631	3.632047	-0.891881
C	-0.238107	3.789144	1.612384
Li	-1.963209	-1.682882	-0.530278
H	-7.281473	-0.460183	-1.020203
H	-7.398489	-2.564950	0.281134
H	1.915720	-3.391709	-2.189509
H	3.842469	-4.799283	-1.575342
H	4.905894	-4.553213	0.652615
H	3.986839	-2.914297	2.272527
H	2.024401	-1.556990	1.683850
H	-3.346022	-1.985277	1.558991
H	-5.414444	-3.336668	1.552145
H	-5.185016	0.845588	-1.095706
H	2.734058	0.192134	-1.961075
H	5.018227	1.070295	-1.593036
H	5.610564	2.123177	0.570324
H	3.909178	2.279540	2.367791
H	1.623998	1.406115	1.982209
H	0.961034	4.746526	-1.334691
H	2.054376	3.516601	-0.679924
H	1.109912	3.172911	-2.132967
H	-1.955463	4.722063	-0.992007
H	-2.092351	3.200565	-1.887975
H	-2.819305	3.349166	-0.283256
H	-0.404972	4.870640	1.570353
H	-0.997313	3.358147	2.272059
H	0.742267	3.620145	2.064716

8a + Li^+

E(ωB97X-D/6-311+G**)= -1605.390435

C	-3.523702	-0.051044	0.362736
C	-3.004797	-1.249565	-0.147852
C	-3.846039	-2.024958	-0.958898
C	-5.130898	-1.607351	-1.277248
C	-5.626499	-0.410776	-0.767508
C	-4.815940	0.360305	0.057459
C	-1.639998	-1.708833	0.167266
C	-0.606965	-0.938004	-0.198473
C	0.863832	-1.086365	-0.038070
Si	1.694103	-2.636553	0.596554
C	3.550098	-2.742600	0.383979
P	-0.344587	0.661553	-1.164049
C	-0.772662	2.018798	0.010624

C	-1.695027	2.980139	-0.404247
C	-2.102630	3.992909	0.457512
C	-1.578179	4.064811	1.741885
C	-0.647556	3.118577	2.162228
C	-0.250959	2.102294	1.303630
C	1.302304	0.091997	-0.556092
C	2.629420	0.694214	-0.657602
C	3.019788	1.362736	-1.823223
C	4.280427	1.933247	-1.923730
C	5.169462	1.861839	-0.855677
C	4.783551	1.224708	0.318108
C	3.522722	0.651860	0.417647
C	1.224981	-2.822731	2.422439
C	0.901787	-4.107074	-0.330908
Li	-1.088511	-3.453174	0.968185
H	-5.188905	1.293728	0.466728
H	-6.633332	-0.087216	-1.007341
H	-2.110563	2.923836	-1.405315
H	-2.827615	4.727220	0.123718
H	-1.890991	4.855359	2.415285
H	-0.231993	3.173892	3.162798
H	0.463298	1.357706	1.640988
H	1.606279	-4.494604	-1.071167
H	0.666107	-4.959123	0.323067
H	0.011798	-3.807917	-0.896831
H	1.431679	-3.825330	2.811200
H	1.800863	-2.114537	3.025551
H	0.174635	-2.567673	2.618285
H	3.889925	-3.768073	0.561313
H	3.835149	-2.459320	-0.633002
H	4.085910	-2.084115	1.069926
H	-3.469120	-2.960776	-1.363143
H	-5.751249	-2.219560	-1.923933
H	-2.901996	0.560873	1.006697
H	3.208239	0.187260	1.346303
H	5.462911	1.185900	1.162781
H	6.151771	2.313937	-0.933437
H	4.570085	2.439701	-2.837754
H	2.324898	1.421992	-2.654221

9a + Li^+

E(ωB97X-D/6-311+G**)= -1605.419911

C	-0.906189	3.335845	-0.540223
C	-0.699433	2.053799	-0.036598
C	-0.602152	1.875565	1.346049
C	-0.709777	2.958822	2.204893
C	-0.918202	4.238372	1.692856
C	-1.016494	4.425267	0.321694
P	-0.552427	0.632511	-1.210712
C	-1.545989	-0.683981	-0.432909
C	-3.024414	-0.733571	-0.465926
C	-3.689840	-1.917878	-0.828842
C	-5.077474	-2.028241	-0.750705
C	-5.836750	-0.943181	-0.336188
C	-5.196700	0.251998	-0.013858
C	-3.815101	0.357645	-0.075302

C	-0.785681	-1.687837	0.098994
C	0.660036	-1.364268	0.010631
Si	1.913584	-2.607626	0.671949
C	1.212373	-4.343130	0.444738
C	0.950793	-0.168778	-0.567217
C	2.270795	0.479437	-0.690664
C	2.859814	0.708240	-1.936403
C	4.113632	1.297914	-2.028110
C	4.792076	1.685290	-0.877181
C	4.203195	1.487679	0.366119
C	2.950906	0.893404	0.457445
C	3.565828	-2.537439	-0.223718
C	2.175987	-2.315526	2.516029
Li	-2.218974	-2.793638	0.883591
H	-5.783237	1.109166	0.299044
H	-6.916568	-1.018953	-0.280857
H	-0.980734	3.484452	-1.612338
H	-1.175819	5.418586	-0.083356
H	-1.000700	5.085005	2.365672
H	-0.628340	2.809733	3.276293
H	-0.444155	0.876152	1.741320
H	-3.101615	-2.734023	-1.245779
H	-5.562894	-2.952178	-1.047389
H	-3.331362	1.288209	0.200902
H	1.940666	-5.098941	0.756232
H	0.304333	-4.484151	1.038007
H	0.958105	-4.526756	-0.603325
H	2.789990	-3.105546	2.960347
H	2.679015	-1.360420	2.691949
H	1.218592	-2.292360	3.046356
H	4.202684	-3.366012	0.104017
H	3.424501	-2.629934	-1.304712
H	4.101195	-1.603637	-0.039300
H	2.483413	0.747349	1.424997
H	4.718165	1.800030	1.268118
H	5.770525	2.146642	-0.950956
H	4.565225	1.453657	-3.001768
H	2.334729	0.401272	-2.834494

6b + Li^+

E(ωB97X-D/6-311+G**)= -1759.004119

C	-0.069556	2.696323	-1.035552
C	1.194519	2.105529	-0.909715
C	2.308731	2.949016	-0.852953
C	2.165379	4.329890	-0.915645
C	0.906019	4.900339	-1.039672
C	-0.212013	4.073110	-1.099857
C	1.331757	0.625667	-0.824920
C	0.276849	-0.159545	-0.637463
C	-0.857046	-0.726478	-0.439838
C	-1.790452	-1.623355	-0.238560
C	-3.211969	-1.347131	-0.040490
C	-3.706383	-0.075569	0.173199
C	-5.083295	0.164427	0.383323
C	-5.987610	-0.931817	0.365110

C	-5.471093	-2.234151	0.143688
C	-4.132858	-2.432450	-0.044469
C	-7.368082	-0.687501	0.573191
C	-7.829979	0.583087	0.787844
C	-6.931593	1.675243	0.805697
C	-5.593628	1.470462	0.608587
P	3.018769	-0.089472	-1.081090
Si	2.667724	-2.307044	-0.813720
C	1.939690	-2.730705	0.888216
C	3.826459	0.148939	0.569949
C	3.112818	0.486807	1.721827
C	3.765572	0.631141	2.939600
C	5.138709	0.429969	3.028124
C	5.859543	0.098872	1.886581
C	5.209526	-0.028990	0.664498
C	1.532827	-2.966208	-2.183679
C	4.319482	-3.181677	-0.955248
Li	-0.145278	-2.740238	-0.356716
H	5.782540	-0.262696	-0.228032
H	6.932639	-0.047418	1.944371
H	5.645473	0.541161	3.980113
H	3.198332	0.901385	3.823627
H	2.040773	0.642408	1.660184
H	-3.741141	-3.431192	-0.204687
H	-6.158594	-3.074152	0.132009
H	-3.019339	0.765854	0.191337
H	-8.056579	-1.526570	0.559518
H	-8.888323	0.757725	0.946050
H	-4.900219	2.305376	0.622255
H	-7.310412	2.676741	0.976377
H	1.576680	-3.766245	0.952428
H	2.739813	-2.644371	1.627636
H	1.160123	-2.040368	1.230852
H	4.191368	-4.262272	-0.835753
H	4.782868	-3.000540	-1.928582
H	5.009726	-2.838705	-0.179980
H	1.261902	-4.018124	-2.024151
H	0.626153	-2.374713	-2.357880
H	2.080547	-2.934910	-3.129355
H	-0.945033	2.059124	-1.092282
H	-1.201256	4.505340	-1.205379
H	0.793802	5.977379	-1.092662
H	3.046368	4.960396	-0.866139
H	3.304011	2.531319	-0.751527

(Z)-7b + Li$^+$

E(ωB97X-D/6-311+G**)= -1759.022175

C	-6.991425	-1.558367	0.167706
C	-5.673607	-1.049917	0.058228
C	-5.444941	0.331435	0.297558
C	-6.546256	1.159245	0.638076
C	-7.808136	0.639116	0.737402
C	-8.034203	-0.735610	0.499925
C	-4.570170	-1.875892	-0.282484
C	-3.310036	-1.362268	-0.377220
C	-3.072288	0.022244	-0.133521

C	-4.129321	0.842586	0.193863
C	-1.737102	0.510567	-0.222493
C	-0.564773	0.798104	-0.284630
C	0.808500	1.168474	-0.350056
Si	1.094543	3.042483	-0.183740
C	1.690400	3.862494	-1.757046
C	1.772920	0.214657	-0.606090
P	3.463211	0.727676	-1.088272
C	4.480594	-0.319189	0.032910
C	5.876569	-0.308912	-0.178554
C	6.749615	-0.929674	0.694819
C	6.276685	-1.591932	1.830562
C	4.913188	-1.624004	2.064014
C	4.028058	-0.997092	1.184136
C	1.408315	-1.234462	-0.630905
C	0.650256	-1.813229	0.392827
C	0.334369	-3.163902	0.372014
C	0.760479	-3.963394	-0.683713
C	1.513393	-3.400943	-1.706926
C	1.844378	-2.052020	-1.674735
C	-0.535008	3.818461	0.333553
C	2.312433	3.436705	1.233131
Li	3.082176	1.188072	1.191690
H	2.962188	-1.111114	1.369354
H	4.518553	-2.149242	2.927623
H	6.965286	-2.080352	2.509933
H	7.815078	-0.903893	0.491062
H	6.269711	0.195860	-1.055915
H	1.754340	4.947272	-1.622327
H	2.670502	3.487891	-2.058018
H	0.986205	3.665855	-2.570740
H	-0.404647	4.893536	0.493371
H	-1.296911	3.679024	-0.437631
H	-0.919446	3.376365	1.256970
H	2.203008	4.496744	1.478510
H	2.076624	2.908736	2.169306
H	3.365701	3.304700	0.959320
H	-2.467760	-1.993456	-0.637799
H	-4.743652	-2.930834	-0.468935
H	-3.954095	1.897124	0.378637
H	-7.161363	-2.614436	-0.015784
H	-9.038062	-1.136596	0.583141
H	-6.370712	2.214753	0.819312
H	-8.641226	1.281622	0.999647
H	2.446003	-1.617561	-2.464645
H	1.852896	-4.016160	-2.532816
H	0.513570	-5.019115	-0.704066
H	-0.244911	-3.593099	1.182340
H	0.307509	-1.197429	1.217410

(E)-7b + Li^{+}

E(ωB97X-D/6-311+G**)= -1759.033114

C	-7.122892	1.407560	-0.646108
C	-5.819976	0.858960	-0.544861
C	-5.480061	0.089304	0.600562
C	-6.458403	-0.105437	1.610719

C	-7.707329	0.437521	1.484864
C	-8.044325	1.202500	0.344158
C	-4.842474	1.049316	-1.553752
C	-3.594970	0.504334	-1.434634
C	-3.237672	-0.258974	-0.280458
C	-4.177799	-0.451520	0.711123
C	-1.892953	-0.739863	-0.163678
C	-0.697394	-0.980955	-0.181472
C	0.679756	-1.291604	-0.156716
Si	1.072851	-3.141931	-0.201369
C	1.260101	-3.871559	1.525051
C	1.582329	-0.241218	-0.228979
P	1.068511	1.416715	-0.657948
C	2.415492	2.523244	-0.090421
C	2.885361	3.514113	-0.958166
C	3.888050	4.395121	-0.566967
C	4.456258	4.289371	0.696285
C	4.000534	3.308276	1.571308
C	2.982200	2.446660	1.187439
C	3.027677	-0.518334	-0.022868
C	3.464396	-1.053108	1.189189
C	4.810863	-1.319884	1.402503
C	5.738872	-1.054678	0.402910
C	5.312836	-0.509153	-0.804248
C	3.969433	-0.234036	-1.012490
C	-0.400717	-3.981956	-1.019637
C	2.607925	-3.522334	-1.215553
Li	-1.207082	1.360677	-0.047570
H	2.627659	1.696452	1.885183
H	4.436949	3.217243	2.560319
H	5.247282	4.966863	0.998546
H	4.236315	5.154743	-1.258876
H	2.468082	3.585631	-1.957804
H	1.261699	-4.965622	1.476187
H	2.195078	-3.557750	1.995786
H	0.432800	-3.565963	2.172816
H	-0.222418	-5.058347	-1.108032
H	-1.320271	-3.837929	-0.445410
H	-0.571477	-3.584173	-2.024402
H	2.665714	-4.602218	-1.389799
H	2.567586	-3.024965	-2.188626
H	3.527947	-3.209714	-0.717037
H	-2.862342	0.621706	-2.227170
H	-5.102371	1.623963	-2.436716
H	-3.913779	-1.026908	1.591803
H	-7.378653	1.991885	-1.523885
H	-9.038183	1.626396	0.257838
H	-6.198737	-0.692903	2.485178
H	-8.447611	0.282316	2.261400
H	3.634914	0.199444	-1.948213
H	6.033161	-0.293396	-1.585487
H	6.790479	-1.262789	0.565605
H	5.135927	-1.732768	2.351304
H	2.737208	-1.246916	1.969968

8b + Li$^+$

E(ωB97X-D/6-311+G**)= -1759.015689

C	4.616763	-0.933270	0.708743
C	4.007962	0.236676	0.184974
C	4.778885	1.098760	-0.638084
C	6.130699	0.770731	-0.905889
C	6.693011	-0.365339	-0.386064
C	5.924665	-1.227574	0.429088
C	2.652449	0.552305	0.444617
C	2.048467	1.673266	-0.085045
C	2.847196	2.542248	-0.884623
C	4.155609	2.262114	-1.160994
C	0.636124	1.994214	0.179303
C	-0.288551	1.108603	-0.217664
C	-1.766918	1.053715	-0.068330
Si	-2.806084	2.457229	0.595230
C	-4.657882	2.303256	0.384159
P	-0.322542	-0.502669	-1.199205
C	0.294562	-1.799608	-0.041378
C	1.343264	-2.614991	-0.468227
C	1.905296	-3.556341	0.387869
C	1.414457	-3.701051	1.679270
C	0.359107	-2.901594	2.111286
C	-0.194717	-1.957780	1.257416
C	-2.033716	-0.172434	-0.591666
C	-3.263074	-0.956730	-0.684719
C	-3.579519	-1.654086	-1.855737
C	-4.748828	-2.396159	-1.942557
C	-5.615133	-2.470718	-0.856221
C	-5.298057	-1.805374	0.322378
C	-4.129691	-1.060154	0.407932
C	-2.364722	2.663367	2.425984
C	-2.223676	4.041489	-0.297754
Li	-0.160996	3.634156	0.997028
H	1.737653	-2.496950	-1.472314
H	2.728996	-4.172825	0.045050
H	1.849563	-4.435189	2.348634
H	-0.028699	-3.013528	3.118215
H	-1.004754	-1.323782	1.604396
H	-2.967732	4.336665	-1.041871
H	-2.119528	4.906747	0.372442
H	-1.294606	3.882976	-0.858155
H	-2.712954	3.615332	2.839934
H	-2.832666	1.863896	3.008185
H	-1.288880	2.554777	2.619404
H	-5.141584	3.262695	0.593825
H	-4.904948	2.012271	-0.640489
H	-5.088249	1.553175	1.050131
H	2.386732	3.434607	-1.299363
H	4.737021	2.929472	-1.790376
H	2.078826	-0.114226	1.079237
H	6.715953	1.434355	-1.535346
H	7.727928	-0.608574	-0.599919
H	4.019289	-1.596131	1.327000
H	6.377584	-2.126819	0.832673
H	-3.864608	-0.570392	1.339171
H	-5.956338	-1.877885	1.181287

H	-6.525016	-3.056296	-0.923808
H	-4.984155	-2.922890	-2.860707
H	-2.902723	-1.600366	-2.701864

9b + Li$^+$

E(ωB97X-D/6-311+G**)= -1759.043524

C	0.019386	3.438728	-0.153830
C	0.212728	2.131928	0.287708
C	0.439691	1.899531	1.646705
C	0.472071	2.955949	2.544134
C	0.276641	4.260403	2.094792
C	0.050758	4.500603	0.747061
P	0.178314	0.745359	-0.926493
C	-0.769083	-0.557798	-0.080470
C	-2.235136	-0.549783	0.052798
C	-2.825931	-1.155252	1.205718
C	-4.184020	-1.274078	1.344064
C	-5.059702	-0.765048	0.354753
C	-4.492443	-0.113949	-0.774442
C	-3.086106	-0.024556	-0.898115
C	-5.366799	0.411924	-1.763214
C	-6.723263	0.297933	-1.631314
C	-7.284470	-0.346202	-0.503492
C	-6.469886	-0.863766	0.465847
C	0.003532	-1.630329	0.275893
C	1.436287	-1.360238	0.022228
Si	2.687243	-2.723851	0.382847
C	4.114329	-2.735535	-0.841731
C	1.711094	-0.138750	-0.514089
C	3.034694	0.456888	-0.770537
C	3.921341	0.671210	0.288120
C	5.181600	1.209950	0.062919
C	5.572796	1.554321	-1.225787
C	4.689017	1.369090	-2.283809
C	3.428530	0.832752	-2.057858
C	3.354164	-2.584754	2.139417
C	1.789805	-4.380676	0.243161
Li	-1.372597	-3.027986	0.485125
H	-0.153628	3.628163	-1.208072
H	-0.100493	5.514081	0.391890
H	0.301307	5.085459	2.798292
H	0.649753	2.766287	3.597260
H	0.590090	0.880738	1.991930
H	-2.164263	-1.461148	2.013975
H	-4.605645	-1.723271	2.238474
H	-2.669354	0.447014	-1.782833
H	-6.893766	-1.355101	1.336049
H	-8.361485	-0.426619	-0.410870
H	-4.937760	0.907417	-2.627956
H	-7.378323	0.704786	-2.393648
H	2.492737	-5.214759	0.336868
H	1.042449	-4.481801	1.036668
H	1.284675	-4.471931	-0.723808
H	3.919047	-3.481641	2.414246
H	4.022586	-1.725760	2.242515
H	2.537886	-2.466418	2.858681

H	4.745338	-3.615282	-0.677235
H	3.741749	-2.775451	-1.869746
H	4.741864	-1.845951	-0.751437
H	2.743233	0.682352	-2.885058
H	4.984115	1.641094	-3.291451
H	6.556204	1.974982	-1.403417
H	5.857623	1.367179	0.896387
H	3.605997	0.420098	1.294688

6b' + Li$^+$

E(ωB97X-D/6-311+G**)= -1759.004679

C	-1.874056	4.083364	0.531834
C	-0.775974	3.444124	-0.102133
C	0.224997	4.246390	-0.710653
C	0.097387	5.657544	-0.670051
C	-0.974240	6.245380	-0.054037
C	-1.971396	5.447716	0.554390
C	1.318459	3.596919	-1.334834
C	1.411181	2.233771	-1.356003
C	0.410890	1.418063	-0.759914
C	-0.651320	2.037103	-0.139090
C	0.513293	-0.065971	-0.774860
C	-0.547117	-0.834345	-0.554580
C	-1.683404	-1.381883	-0.319855
C	-2.620192	-2.256137	-0.047108
C	-4.025883	-1.936513	0.199872
C	-4.889994	-2.934763	0.664784
C	-6.229383	-2.667012	0.917264
C	-6.737777	-1.392205	0.696978
C	-5.896629	-0.387028	0.225406
C	-4.558645	-0.655429	-0.017391
P	2.156232	-0.807305	-1.176169
Si	1.844721	-2.983371	-0.657737
C	0.675880	-3.777632	-1.925187
C	3.175584	-0.380926	0.306866
C	2.622778	0.058486	1.511779
C	3.439975	0.354693	2.595987
C	4.818801	0.204694	2.497207
C	5.379227	-0.230631	1.302006
C	4.563858	-0.509847	0.211585
C	3.503324	-3.849829	-0.761259
C	1.164118	-3.239844	1.093684
Li	-0.985385	-3.394143	-0.147085
H	-6.289763	0.608060	0.046753
H	-7.784718	-1.182462	0.886403
H	5.010772	-0.825181	-0.726580
H	6.454386	-0.340164	1.211692
H	5.454377	0.436462	3.344550
H	2.996326	0.706186	3.521037
H	1.548188	0.178480	1.598526
H	-4.489323	-3.929763	0.827234
H	-6.880097	-3.454869	1.280967
H	-3.905608	0.127574	-0.390236
H	0.866148	-4.280950	1.277650
H	1.966131	-3.020574	1.803486
H	0.345892	-2.564631	1.368801

H	3.390064	-4.921055	-0.566449
H	3.951798	-3.731744	-1.751288
H	4.199612	-3.443860	-0.022475
H	0.378110	-4.795981	-1.640843
H	-0.215916	-3.182884	-2.157891
H	1.209283	-3.876204	-2.874209
H	2.257100	1.765789	-1.847587
H	2.090109	4.200257	-1.802442
H	-1.415594	1.432276	0.339102
H	0.865006	6.266155	-1.137646
H	-1.064138	7.325652	-0.029412
H	-2.816208	5.924420	1.039014
H	-2.637817	3.468730	0.997812

(Z)-7b' + Li$^+$

E(ωB97X-D/6-311+G**)= -1759.023260

C	3.046826	-0.409098	1.565564
C	3.502425	-0.388292	0.233452
C	4.733321	0.264246	0.000146
C	5.461458	0.852040	1.031094
C	4.975628	0.829241	2.332247
C	3.764210	0.191739	2.589073
P	2.588243	-1.139699	-1.180718
C	0.865173	-0.847988	-0.575099
C	-0.079250	-1.821803	-0.466406
Si	0.268260	-3.692117	-0.574868
C	-1.349787	-4.568823	-0.185312
C	0.466613	0.573146	-0.323184
C	0.097255	1.004648	0.981074
C	-0.214831	2.310556	1.231502
C	-0.197908	3.282461	0.198339
C	0.132713	2.865804	-1.117981
C	0.463490	1.502481	-1.343558
C	0.124209	3.823772	-2.164894
C	-0.182171	5.133148	-1.909060
C	-0.498431	5.550056	-0.595949
C	-0.507974	4.645556	0.431445
C	-1.425235	-1.440854	-0.193580
C	-2.561195	-1.107678	0.048514
C	-3.870546	-0.634468	0.357205
C	-4.101197	0.741977	0.485799
C	-5.368758	1.213185	0.794866
C	-6.422023	0.322815	0.978588
C	-6.201226	-1.044574	0.851156
C	-4.936004	-1.523689	0.543073
C	0.819867	-4.198737	-2.295225
C	1.538325	-4.159182	0.727243
Li	2.775089	1.272323	-1.081281
H	-7.018870	-1.742628	0.992310
H	-7.412069	0.694071	1.218338
H	5.139346	0.277423	-1.011363
H	6.410550	1.330265	0.812516
H	5.533247	1.294913	3.136396
H	3.378823	0.152824	3.602756
H	2.125632	-0.933451	1.793654
H	-3.275468	1.429819	0.340006

H	-5.536292	2.280024	0.892799
H	-4.759167	-2.588195	0.441998
H	0.685605	1.171453	-2.356503
H	-0.481792	2.620795	2.236741
H	0.059312	0.271200	1.776649
H	1.737477	-5.235418	0.696066
H	2.480458	-3.630517	0.565480
H	1.169556	-3.916441	1.728596
H	-1.206042	-5.653824	-0.209668
H	-1.723923	-4.297452	0.806041
H	-2.124960	-4.312710	-0.913195
H	0.915181	-5.287954	-2.357647
H	0.088701	-3.881567	-3.044774
H	1.784517	-3.753296	-2.546455
H	0.355535	3.500358	-3.175142
H	-0.188083	5.857223	-2.715850
H	-0.736629	6.590647	-0.407933
H	-0.755052	4.960477	1.440150

(E)-7b' + Li$^+$

E(ωB97X-D/6-311+G**)= -1759.035354

C	-1.344651	2.642716	1.122269
C	-0.799728	2.675764	-0.166799
C	-1.276651	3.641728	-1.058577
C	-2.268603	4.539533	-0.679514
C	-2.817421	4.476399	0.595262
C	-2.352271	3.521671	1.493996
P	0.517031	1.531985	-0.719175
C	0.059746	-0.081895	-0.132838
C	1.007122	-1.092310	0.002335
Si	0.686871	-2.957097	-0.018403
C	2.174642	-3.746187	-0.862140
C	-1.371277	-0.392070	0.115713
C	-1.758596	-0.918965	1.375167
C	-3.065435	-1.217559	1.641242
C	-4.070027	-1.010864	0.662803
C	-3.692365	-0.472305	-0.595681
C	-2.333125	-0.165590	-0.836579
C	-4.693563	-0.255648	-1.577627
C	-6.002588	-0.557357	-1.320205
C	-6.378003	-1.093754	-0.066486
C	-5.434788	-1.313979	0.900074
C	2.364609	-0.728863	0.024143
C	3.551150	-0.448550	0.103093
C	4.876777	0.077001	0.106094
C	5.154496	1.267442	0.804365
C	6.414034	1.852839	0.733619
C	7.422951	1.250213	-0.004882
C	7.166443	0.055497	-0.676401
C	5.910082	-0.524132	-0.632793
C	0.568646	-3.666925	1.721373
C	-0.857381	-3.404437	-0.989818
Li	2.874537	1.529093	-0.543096
H	7.955013	-0.422748	-1.246432
H	8.406898	1.701423	-0.052846
H	-0.876210	3.679459	-2.067105

H	-2.624405	5.278755	-1.389324
H	-3.601281	5.166167	0.887780
H	-2.773744	3.464207	2.491858
H	-0.982453	1.913366	1.837986
H	4.387352	1.702151	1.441040
H	6.608256	2.771921	1.274716
H	5.706276	-1.443544	-1.168508
H	-2.046159	0.250850	-1.796576
H	-3.349681	-1.614321	2.610836
H	-0.995843	-1.067252	2.131521
H	-0.873197	-4.485942	-1.162345
H	-1.775074	-3.133165	-0.463524
H	-0.869130	-2.906474	-1.963256
H	2.033219	-4.828244	-0.946767
H	2.314319	-3.343304	-1.869762
H	3.095843	-3.568603	-0.299971
H	0.605834	-4.760904	1.689670
H	1.399746	-3.320850	2.343270
H	-0.365749	-3.379304	2.209855
H	-4.401715	0.160223	-2.536450
H	-6.759686	-0.385376	-2.077078
H	-7.419246	-1.327538	0.125375
H	-5.719800	-1.725126	1.863340

8b' + Li^+

E(ωB97X-D/6-311+G**)= -1759.013039

C	0.717143	-1.755180	1.666159
C	0.964755	-1.994377	0.312469
C	1.420092	-3.254812	-0.074147
C	1.636347	-4.252794	0.871061
C	1.388931	-4.002361	2.214337
C	0.925123	-2.750342	2.610428
P	0.769032	-0.696021	-0.986001
C	1.582087	0.851955	-0.274120
C	2.822899	1.335706	-0.121695
C	3.964394	0.484978	-0.503800
C	4.896301	0.925320	-1.454950
C	5.974501	0.136026	-1.831281
C	6.171257	-1.110215	-1.243394
C	5.272178	-1.553779	-0.280661
C	4.184002	-0.769507	0.083728
C	-0.562706	0.369386	-0.282542
C	0.241871	1.420365	0.029298
Si	-0.045891	3.190474	0.555226
C	0.931572	4.289956	-0.659230
C	-1.999652	0.149095	-0.141740
C	-2.724347	-0.470688	-1.134171
C	-4.117143	-0.685955	-1.006541
C	-4.777451	-0.273384	0.181303
C	-4.010361	0.333605	1.208047
C	-2.668613	0.534109	1.052526
C	-6.171963	-0.493613	0.307275
C	-6.878969	-1.092386	-0.700223
C	-6.223246	-1.502465	-1.883808
C	-4.877616	-1.305051	-2.032664
C	-1.821407	3.778926	0.589516

C	0.748810	3.406885	2.264060
Li	2.862819	3.227913	0.521507
H	5.413688	-2.520934	0.190852
H	7.017464	-1.724614	-1.530423
H	1.618379	-3.451556	-1.122892
H	1.997702	-5.225732	0.556035
H	1.554573	-4.778898	2.953139
H	0.729927	-2.551838	3.658892
H	0.370671	-0.775255	1.980204
H	1.414963	5.149451	-0.175095
H	1.665898	3.718011	-1.238986
H	0.244681	4.711610	-1.397725
H	0.908464	4.458139	2.526160
H	0.091716	2.979789	3.027541
H	1.692803	2.855592	2.371707
H	-1.855292	4.868140	0.694010
H	-2.333415	3.508445	-0.338050
H	-2.386175	3.341763	1.414896
H	4.753833	1.897506	-1.919483
H	6.668345	0.495326	-2.584492
H	3.492035	-1.126716	0.837832
H	-2.085175	0.967017	1.858062
H	-4.504613	0.624065	2.129847
H	-2.223016	-0.791882	-2.042165
H	-6.671288	-0.179609	1.218429
H	-7.945370	-1.256115	-0.593369
H	-6.793111	-1.977134	-2.674731
H	-4.371869	-1.621039	-2.939274

9b' + Li$^+$

E(ωB97X-D/6-311+G**)= -1759.042560

C	4.369350	-1.042796	-0.416532
C	3.752095	0.160161	-0.790712
C	4.565433	1.154807	-1.361808
C	5.941102	0.978631	-1.504426
C	6.532803	-0.211075	-1.104530
C	5.735509	-1.223179	-0.573318
C	2.316990	0.417269	-0.539098
C	1.859808	1.587407	0.000606
C	0.383091	1.564531	0.146588
Si	-0.492975	3.069111	0.870585
C	-0.566567	2.922156	2.748766
C	-0.221939	0.426269	-0.284355
P	0.977803	-0.710301	-1.048406
C	1.005883	-2.046778	0.230213
C	1.219677	-1.760106	1.581188
C	1.225674	-2.776241	2.524260
C	1.015855	-4.096666	2.129750
C	0.799684	-4.390572	0.791206
C	0.795501	-3.368338	-0.155829
C	-1.648865	0.064770	-0.175275
C	-2.445844	-0.088701	-1.283416
C	-3.823023	-0.395715	-1.164534
C	-4.385643	-0.568811	0.127625
C	-3.539910	-0.443870	1.258523
C	-2.215489	-0.141306	1.111295

C	-5.767545	-0.865102	0.243033
C	-6.554168	-0.980855	-0.870868
C	-5.994863	-0.808241	-2.158029
C	-4.664179	-0.523874	-2.300458
C	0.509481	4.608664	0.446392
C	-2.229814	3.297871	0.187119
Li	3.587463	2.428047	0.453000
H	6.188134	-2.161038	-0.269869
H	7.599738	-0.359991	-1.223490
H	0.624623	-3.599152	-1.202062
H	0.632240	-5.415647	0.479257
H	1.019571	-4.890848	2.868179
H	1.392157	-2.543644	3.570677
H	1.383742	-0.729000	1.881363
H	4.086440	2.045857	-1.765050
H	6.540542	1.759861	-1.960179
H	3.766104	-1.832531	0.017995
H	-0.015347	5.513972	0.768531
H	1.484351	4.591226	0.942676
H	0.683444	4.680511	-0.631159
H	-0.953143	3.841642	3.200350
H	-1.220771	2.100614	3.053678
H	0.427430	2.733246	3.166520
H	-2.632845	4.265222	0.505136
H	-2.223602	3.278203	-0.906673
H	-2.915142	2.518120	0.527118
H	-1.570308	-0.059562	1.978861
H	-3.960534	-0.595911	2.247553
H	-2.021913	0.054728	-2.272474
H	-6.193714	-0.997941	1.232472
H	-7.610388	-1.204263	-0.770163
H	-6.627488	-0.902014	-3.033721
H	-4.231625	-0.390691	-3.286884

6c + Li^+

E(ωB97X-D/6-311+G**)= -2079.777390

C	-5.819548	1.534369	-0.126381
C	-5.259807	0.245393	0.079246
C	-6.108399	-0.809691	0.511103
C	-7.484043	-0.542739	0.723100
C	-7.994267	0.710657	0.517002
C	-7.151423	1.761785	0.086886
C	-5.544605	-2.096015	0.709796
C	-4.215264	-2.318138	0.488538
C	-3.346029	-1.272236	0.068081
C	-3.886360	-0.016416	-0.128352
C	-1.933243	-1.570061	-0.153231
C	-0.982136	-0.692515	-0.349260
C	0.173448	-0.165381	-0.534819
C	1.252011	0.590925	-0.706310
C	1.160902	2.055589	-0.622695
S	2.384321	3.099976	-1.285456
C	1.545198	4.509888	-0.764921
C	0.387904	4.196926	-0.118339
C	0.170505	2.796287	-0.031934
P	2.910448	-0.121005	-1.094272

Si	2.547381	-2.339490	-0.843247
C	1.362425	-2.958750	-2.188457
C	3.829853	0.099768	0.495749
C	3.200539	0.373431	1.711852
C	3.945229	0.493196	2.878689
C	5.326292	0.331788	2.850039
C	5.962631	0.066256	1.643313
C	5.220158	-0.037513	0.472876
C	4.186995	-3.225072	-1.034231
C	1.850356	-2.765876	0.870061
Li	-0.292057	-2.694796	-0.327886
H	5.725622	-0.219632	-0.470788
H	7.040618	-0.047567	1.608769
H	5.903877	0.423400	3.763054
H	3.443731	0.712419	3.814982
H	2.123355	0.499436	1.743435
H	-0.680771	2.336852	0.453647
H	-0.285776	4.939458	0.288940
H	1.958013	5.487106	-0.965046
H	-3.790211	-3.305455	0.633834
H	-6.190446	-2.905286	1.035966
H	-3.242891	0.793289	-0.460976
H	-8.129562	-1.350868	1.052488
H	-9.048483	0.902710	0.682517
H	-5.169311	2.337864	-0.457518
H	-7.568187	2.749820	-0.073518
H	1.459411	-3.791595	0.925226
H	2.666756	-2.712858	1.594625
H	1.095752	-2.059916	1.235905
H	4.057583	-4.304863	-0.909964
H	4.623950	-3.046709	-2.020217
H	4.899854	-2.882569	-0.279236
H	1.064265	-4.003032	-2.028298
H	0.469473	-2.338477	-2.332992
H	1.884446	-2.935953	-3.148741

(Z)-7c + Li^+

E(ωB97X-D/6-311+G**)= -2079.797049

C	-6.730187	-2.059373	-0.016572
C	-5.468146	-1.416302	-0.013348
C	-5.414116	-0.001914	-0.132838
C	-6.629002	0.721346	-0.254542
C	-7.833763	0.072315	-0.255707
C	-7.886302	-1.334873	-0.134224
C	-4.155964	0.645514	-0.125709
C	-2.985477	-0.071585	-0.006689
C	-3.047714	-1.492499	0.108436
C	-4.250110	-2.136559	0.105742
C	-1.710176	0.560123	0.010874
C	-0.582720	0.996658	0.059221
C	0.739501	1.515708	0.086891
C	1.817829	0.613066	0.128983
P	3.497792	1.161167	-0.143006
C	4.494599	-0.377157	-0.074923
C	5.292362	-0.739169	-1.163109

C	6.093746	-1.874248	-1.116538
C	6.106403	-2.671420	0.021317
C	5.328787	-2.314203	1.117975
C	4.543659	-1.170188	1.076403
Si	0.852923	3.399802	0.209989
C	1.889826	3.961968	1.662052
C	1.608373	4.083182	-1.403732
C	-0.883002	4.092092	0.330474
C	1.484363	-0.823459	0.278629
S	1.651900	-1.938311	-1.035805
C	1.022665	-3.236012	-0.096174
C	0.718432	-2.831522	1.167828
C	0.972489	-1.446995	1.377809
Li	1.858670	1.683941	-1.839651
H	3.951954	-0.895238	1.942204
H	5.339938	-2.925135	2.014176
H	6.722720	-3.562987	0.056598
H	6.700792	-2.141192	-1.975002
H	5.275876	-0.132716	-2.063416
H	0.773413	-0.922925	2.303159
H	0.324869	-3.496152	1.925684
H	0.928324	-4.221056	-0.527317
H	1.586871	5.176267	-1.377646
H	2.659852	3.807264	-1.548109
H	1.021118	3.803361	-2.291854
H	-0.852275	5.183532	0.406268
H	-1.484036	3.821746	-0.541769
H	-1.394565	3.701647	1.214068
H	1.997409	5.051217	1.660235
H	1.407808	3.671119	2.599690
H	2.886179	3.515722	1.638763
H	-2.117788	-2.043334	0.200663
H	-4.288954	-3.217428	0.197558
H	-4.116414	1.726142	-0.214041
H	-6.766940	-3.140094	0.078033
H	-8.846770	-1.837769	-0.134712
H	-6.586184	1.801842	-0.347835
H	-8.755498	0.635616	-0.349488

(E)-7c + Li^{+}

E(ωB97X-D/6-311+G**)= -2079.805708

C	2.911247	2.410116	1.234587
C	2.398214	2.499441	-0.065015
C	2.854124	3.539990	-0.881182
C	3.790397	4.457742	-0.418722
C	4.307823	4.340791	0.865552
C	3.866058	3.309962	1.688959
P	1.128469	1.357778	-0.731565
C	1.603300	-0.282486	-0.185727
C	3.021089	-0.583408	0.075378
C	3.555490	-1.116639	1.215593
C	4.966305	-1.277293	1.152740
C	5.481929	-0.862647	-0.038563
S	4.261982	-0.254893	-1.086788
C	0.683698	-1.314798	-0.082607
Si	1.046607	-3.173875	-0.069906

C	2.560002	-3.611299	-1.091911
C	-0.689837	-0.984865	-0.132104
C	-1.884688	-0.741939	-0.137116
C	-3.227484	-0.262173	-0.277683
C	-3.577658	0.463639	-1.457770
C	-4.823583	1.006944	-1.600095
C	-5.806028	0.850656	-0.590103
C	-5.473004	0.116831	0.580544
C	-4.172369	-0.422218	0.714718
C	-6.456435	-0.045316	1.591344
C	-7.703813	0.495399	1.442690
C	-8.033843	1.225392	0.277271
C	-7.107403	1.398201	-0.714560
C	1.232201	-3.852767	1.677006
C	-0.451454	-4.011477	-0.845574
Li	-1.163440	1.349968	-0.148697
H	2.564855	1.622073	1.893505
H	4.262735	3.209882	2.693827
H	5.049381	5.046842	1.222763
H	4.129777	5.254720	-1.071953
H	2.482028	3.619899	-1.897914
H	2.948111	-1.361202	2.076756
H	5.568886	-1.678144	1.957107
H	6.513272	-0.876525	-0.358017
H	1.179599	-4.946565	1.664021
H	2.188825	-3.571218	2.123539
H	0.431647	-3.487811	2.327620
H	-0.288551	-5.092226	-0.906926
H	-1.361187	-3.839814	-0.263436
H	-0.630483	-3.639374	-1.858671
H	2.591713	-4.695541	-1.244102
H	2.519249	-3.134260	-2.074926
H	3.492233	-3.309435	-0.609379
H	-2.842398	0.552665	-2.251798
H	-5.078203	1.552970	-2.502506
H	-3.913692	-0.969450	1.614635
H	-7.357807	1.955236	-1.611393
H	-9.026418	1.648276	0.173158
H	-6.202049	-0.605426	2.485043
H	-8.448128	0.365723	2.220007

8c + Li^+

E(ωB97X-D/6-311+G**)= -2079.789470

C	4.656618	-0.810078	0.751281
C	4.030014	0.329304	0.183164
C	4.778859	1.154387	-0.696034
C	6.128613	0.824195	-0.971427
C	6.709214	-0.280612	-0.406241
C	5.961686	-1.107984	0.462716
C	2.678595	0.650256	0.456424
C	2.056223	1.739853	-0.115497
C	2.831581	2.570557	-0.976916
C	4.136528	2.285328	-1.265046
C	0.650158	2.070716	0.175666
C	-0.282668	1.174908	-0.172729
C	-1.756035	1.095184	-0.002779

Si	-2.990979	2.353325	0.647434
C	-4.577980	2.321252	-0.347612
P	-0.281867	-0.458222	-1.119823
C	0.347599	-1.720324	0.070391
C	1.411881	-2.529070	-0.330145
C	1.970800	-3.452441	0.547165
C	1.460888	-3.586862	1.832455
C	0.390829	-2.793355	2.237968
C	-0.159797	-1.866809	1.363467
C	-1.992896	-0.147880	-0.511025
C	-3.185838	-0.966532	-0.561028
C	-4.135638	-1.157403	0.408923
C	-5.161740	-2.062308	0.024921
C	-4.974668	-2.560279	-1.229379
S	-3.545197	-1.936594	-1.956008
C	-3.325894	2.089457	2.479880
C	-2.287255	4.124229	0.496840
Li	0.043135	3.779778	0.950933
H	1.820696	-2.419879	-1.329474
H	2.805877	-4.064719	0.224717
H	1.891753	-4.309197	2.517304
H	-0.013826	-2.898618	3.238984
H	-0.983360	-1.239301	1.689486
H	-5.594583	-3.260993	-1.768199
H	-5.993673	-2.340062	0.658525
H	-4.075168	-0.685127	1.380209
H	-3.107402	4.825748	0.322107
H	-1.829416	4.478154	1.434307
H	-1.606503	4.231227	-0.356520
H	-3.934183	2.901875	2.890790
H	-3.858902	1.153182	2.662120
H	-2.387980	2.045635	3.042829
H	-5.285867	3.066952	0.028347
H	-4.376084	2.541767	-1.399739
H	-5.057396	1.341160	-0.303034
H	2.355851	3.435665	-1.430921
H	4.700522	2.923097	-1.939295
H	2.122732	0.014435	1.136636
H	6.698129	1.460854	-1.641799
H	7.743072	-0.524597	-0.624192
H	4.074579	-1.446805	1.410219
H	6.428526	-1.983643	0.900778

9c + Li$^+$

E(ωB97X-D/6-311+G**)= -2079.817413

C	-5.341524	0.377712	-1.782239
C	-4.474378	-0.145835	-0.786056
C	-5.049048	-0.805693	0.334232
C	-6.459591	-0.914716	0.429767
C	-7.267217	-0.398793	-0.546249
C	-6.698512	0.253706	-1.665388
C	-3.067339	-0.044618	-0.893398
C	-2.223860	-0.567214	0.065106
C	-2.822128	-1.183410	1.208184
C	-4.180653	-1.312722	1.331069
C	-0.755976	-0.561849	-0.049489

C	0.018565	-1.633165	0.305546
C	1.451034	-1.353208	0.070108
Si	2.716606	-2.702453	0.437538
C	4.130907	-2.716534	-0.800282
C	1.721528	-0.121827	-0.448508
C	3.039250	0.478904	-0.642767
C	3.986641	0.730754	0.310883
C	5.170944	1.322537	-0.210716
C	5.105532	1.524368	-1.555656
S	3.598675	0.998208	-2.202674
P	0.188881	0.760372	-0.869577
C	0.203192	2.128958	0.364215
C	-0.012060	3.438392	-0.059025
C	0.000596	4.487354	0.857308
C	0.229820	4.231532	2.201603
C	0.447718	2.924319	2.632237
C	0.434292	1.880745	1.719633
C	3.395030	-2.530715	2.186281
C	1.826699	-4.365408	0.329627
Li	-1.342233	-3.049082	0.499620
H	5.853300	1.956740	-2.203446
H	6.028133	1.594827	0.390987
H	3.819196	0.515585	1.357679
H	-0.186880	3.639890	-1.110708
H	-0.167275	5.503253	0.516925
H	0.240215	5.046689	2.916863
H	0.628405	2.722769	3.682631
H	0.602704	0.859991	2.050205
H	-2.166994	-1.488132	2.022189
H	-4.608780	-1.768801	2.218857
H	-2.644248	0.434174	-1.771196
H	-6.889334	-1.412357	1.293467
H	-8.344580	-0.486868	-0.465416
H	-4.906733	0.879591	-2.640374
H	-7.348177	0.659107	-2.433065
H	2.532894	-5.194682	0.439551
H	1.079069	-4.454014	1.124425
H	1.323051	-4.477092	-0.636027
H	3.971886	-3.417208	2.469772
H	4.054739	-1.663157	2.270378
H	2.583702	-2.410169	2.910687
H	4.785290	-3.575536	-0.618690
H	3.750523	-2.792733	-1.823233
H	4.735315	-1.808615	-0.736559

6c’ + Li^+

E(ωB97X-D/6-311+G**)= -2079.781529

C	2.634602	-0.486387	1.468202
C	3.034774	-1.054781	0.256802
C	4.350005	-1.509080	0.128926
C	5.238027	-1.422256	1.194474
C	4.828835	-0.857364	2.396872
C	3.528092	-0.383628	2.527489
P	1.903942	-1.246420	-1.193704
Si	1.088761	-3.271516	-0.612013
C	0.365420	-3.304695	1.140790

C	0.499907	-0.121839	-0.782433
C	-0.718572	-0.605715	-0.564764
C	-1.953181	-0.854729	-0.333776
C	-3.078968	-1.472621	-0.062945
C	-4.341732	-0.821204	0.164664
C	-4.701094	0.503569	0.062813
C	-6.064394	0.746463	0.359883
C	-6.741619	-0.394390	0.688628
S	-5.723302	-1.773292	0.630410
C	0.767004	1.340535	-0.764571
C	1.943608	1.883474	-1.349942
C	2.197623	3.225515	-1.315360
C	1.299579	4.124299	-0.688321
C	0.123203	3.595360	-0.094678
C	-0.110695	2.202763	-0.145055
C	1.534586	5.520844	-0.629074
C	0.643236	6.354399	-0.009174
C	-0.529194	5.829311	0.582707
C	-0.781996	4.485509	0.541601
C	-0.237332	-3.800873	-1.864017
C	2.493116	-4.510049	-0.678193
Li	-1.761082	-2.968199	-0.117456
H	4.683378	-1.929546	-0.815195
H	6.254005	-1.783987	1.079716
H	5.524401	-0.777406	3.224779
H	3.205627	0.069811	3.458343
H	1.621721	-0.113951	1.578749
H	-0.176782	-4.236686	1.350566
H	1.196363	-3.267700	1.850298
H	-0.264652	-2.443480	1.391066
H	2.129919	-5.516144	-0.445813
H	2.953497	-4.537467	-1.669370
H	3.267684	-4.252949	0.049234
H	-0.772731	-4.707455	-1.551357
H	-0.958680	-3.016482	-2.124736
H	0.258600	-4.054863	-2.804463
H	2.646822	1.222058	-1.844100
H	3.099381	3.619184	-1.773470
H	-1.005811	1.806160	0.323878
H	2.435453	5.920113	-1.084301
H	0.831827	7.421359	0.031529
H	-1.228040	6.499402	1.071000
H	-1.680374	4.079738	0.995950
H	-3.992965	1.268283	-0.230948
H	-6.528646	1.723560	0.330418
H	-7.782734	-0.491323	0.958497

(Z)-7c’ + Li$^+$

E(ωB97X-D/6-311+G**)= -2079.788038

C	-5.463475	-0.143478	0.225063
C	-4.178518	0.314099	0.073458
S	-4.142766	2.055161	0.070109
C	-5.849512	2.141051	0.272464
C	-6.416369	0.903229	0.338725
C	-2.985574	-0.423984	-0.060135

C	-1.924045	-0.998013	-0.154125
C	-0.709296	-1.725185	-0.259783
Si	-0.907511	-3.578350	-0.159138
C	-0.389681	-4.520041	-1.726393
C	0.500606	-1.070273	-0.368851
P	2.001862	-1.890024	-0.948680
C	3.349516	-1.092135	0.011488
C	4.597841	-0.926268	-0.599443
C	5.667560	-0.363964	0.087943
C	5.508130	0.066487	1.399675
C	4.272411	-0.084951	2.020154
C	3.212234	-0.668848	1.339751
C	0.566792	0.399859	-0.137045
C	1.186787	1.237491	-1.030250
C	1.277221	2.630811	-0.796206
C	0.727591	3.171186	0.395134
C	0.098968	2.289566	1.311414
C	0.016712	0.952274	1.052050
C	1.912955	3.503555	-1.715847
C	1.998367	4.845845	-1.462900
C	1.449849	5.383243	-0.276174
C	0.829843	4.564897	0.629658
C	0.163579	-4.227059	1.293462
C	-2.684089	-4.044870	0.194097
Li	1.820676	-4.165890	-0.334299
H	4.726140	-1.223345	-1.636264
H	6.623467	-0.242902	-0.410795
H	6.336495	0.521422	1.931178
H	4.132820	0.251458	3.042149
H	2.258584	-0.784211	1.843908
H	1.621820	0.824791	-1.934547
H	-0.319770	2.694827	2.227102
H	-0.466834	0.290269	1.761418
H	0.549635	-5.250391	1.162250
H	0.980429	-3.544820	1.560842
H	-0.474710	-4.281310	2.178511
H	-2.791908	-5.127653	0.310430
H	-3.036726	-3.560853	1.108712
H	-3.336808	-3.719386	-0.620213
H	-0.387618	-5.604817	-1.565916
H	-1.132403	-4.315735	-2.503384
H	0.564326	-4.209810	-2.169433
H	2.335301	3.085773	-2.624025
H	2.488851	5.503473	-2.171954
H	1.524051	6.448366	-0.086646
H	0.407284	4.974899	1.541612
H	-6.339509	3.101297	0.331670
H	-7.478658	0.741661	0.464988
H	-5.703247	-1.197811	0.253408

(E)-7c' + Li$^+$

E(ωB97X-D/6-311+G**)= -2079.808390

C	5.933309	-0.326049	-0.936205
C	4.889754	0.005827	-0.108307
S	5.387066	1.254591	1.020295
C	6.963342	1.330054	0.327419

C	7.112295	0.422455	-0.676771
C	3.556010	-0.455179	-0.096637
C	2.363515	-0.724338	-0.116534
C	1.006847	-1.086126	-0.084306
Si	0.691655	-2.952440	-0.062393
C	0.632638	-3.629563	1.693426
C	0.053476	-0.077536	-0.189889
P	0.484988	1.544785	-0.771624
C	-0.847429	2.668284	-0.213551
C	-1.352080	3.614338	-1.111178
C	-2.354163	4.500232	-0.730474
C	-2.885167	4.444384	0.552021
C	-2.392510	3.509166	1.456650
C	-1.374802	2.642765	1.082884
C	-1.368412	-0.394870	0.099193
C	-2.354201	-0.189196	-0.832822
C	-3.704801	-0.502640	-0.554512
C	-4.047497	-1.024728	0.720703
C	-3.018253	-1.208898	1.677682
C	-1.720358	-0.905103	1.375527
C	-4.730891	-0.308132	-1.515108
C	-6.030880	-0.616383	-1.221847
C	-6.371501	-1.136800	0.048408
C	-5.403774	-1.334904	0.995293
C	-0.881370	-3.420372	-0.976201
C	2.153754	-3.753588	-0.938156
Li	2.834331	1.594002	-0.536806
H	-0.965195	3.645783	-2.125171
H	-2.730992	5.224746	-1.444611
H	-3.676548	5.124847	0.846127
H	-2.799910	3.457643	2.460655
H	-0.991349	1.928728	1.802953
H	-2.094125	0.216272	-1.805079
H	-3.275703	-1.592676	2.659900
H	-0.938780	-1.036175	2.115641
H	-0.894465	-4.503710	-1.137188
H	-1.782873	-3.150608	-0.421978
H	-0.929940	-2.932830	-1.953738
H	2.009327	-4.836742	-1.001580
H	2.263728	-3.367073	-1.955798
H	3.091918	-3.569018	-0.407159
H	0.673802	-4.723792	1.681267
H	1.482748	-3.268712	2.280268
H	-0.286126	-3.336094	2.207360
H	-4.465804	0.095729	-2.486744
H	-6.807064	-0.462078	-1.963031
H	-7.405903	-1.376156	0.268407
H	-5.661997	-1.733734	1.971219
H	7.692532	2.025882	0.713723
H	8.036583	0.296432	-1.224684
H	5.840998	-1.078327	-1.707435

8c’ + Li^+

E(ωB97X-D/6-311+G**)= -2079.789304

C	5.246232	0.314837	0.137302
C	4.028918	0.393181	-0.494931

S	4.026507	-0.697271	-1.869944
C	5.632698	-1.229045	-1.544206
C	6.151037	-0.614932	-0.444941
C	2.888159	1.208807	-0.101879
C	1.633283	0.772032	-0.304094
P	0.755220	-0.752802	-0.986131
C	0.923624	-2.015691	0.343832
C	2.001144	-2.040599	1.230974
C	2.080659	-3.023391	2.211770
C	1.083238	-3.983620	2.328401
C	0.005860	-3.965614	1.448678
C	-0.066074	-2.997248	0.455758
C	-0.526732	0.357204	-0.254171
C	0.321997	1.379416	0.029035
Si	0.090959	3.162213	0.533384
C	-1.677572	3.742707	0.711585
C	-1.966887	0.183061	-0.087545
C	-2.580494	0.491518	1.157488
C	-3.923093	0.323621	1.342350
C	-4.745428	-0.167957	0.296654
C	-4.140900	-0.499192	-0.945361
C	-2.745488	-0.323791	-1.103058
C	-6.142269	-0.350860	0.453548
C	-6.904397	-0.833794	-0.575675
C	-6.304609	-1.159462	-1.814009
C	-4.958074	-0.997287	-1.993627
C	1.031565	3.400228	2.183454
C	0.967915	4.229267	-0.759462
Li	2.973730	3.014272	0.753524
H	2.780976	-1.291557	1.157421
H	2.927100	-3.034695	2.890274
H	1.145506	-4.745926	3.097224
H	-0.776244	-4.712764	1.529631
H	-0.905035	-2.999856	-0.234341
H	1.105071	5.262282	-0.422983
H	1.933071	3.813515	-1.075108
H	0.364989	4.258535	-1.671824
H	1.621911	4.326459	2.229917
H	0.316569	3.470486	3.007044
H	1.663292	2.535808	2.426798
H	-1.710045	4.829768	0.835994
H	-2.256735	3.483102	-0.178793
H	-2.174803	3.286231	1.569675
H	-1.952385	0.835391	1.972014
H	-4.376643	0.551536	2.301842
H	-2.285826	-0.581250	-2.052586
H	-6.599423	-0.102085	1.406021
H	-7.971942	-0.970155	-0.444818
H	-6.918900	-1.540507	-2.622149
H	-4.494764	-1.247404	-2.942574
H	6.110681	-1.948274	-2.192621
H	7.147884	-0.807315	-0.069318
H	5.472811	0.902886	1.019167

9c' + Li^+

E(ωB97X-D/6-311+G**)= -2079.820620

S	4.405321	-0.784250	-1.890855
C	3.743409	0.108988	-0.553206
C	4.762777	0.527076	0.279946
C	6.061463	0.132923	-0.163614
C	6.022757	-0.587437	-1.314964
C	2.321512	0.406438	-0.411635
C	1.869987	1.640468	-0.014605
C	0.394981	1.646095	0.096983
Si	-0.463171	3.253207	0.589697
C	0.593988	4.687001	-0.029408
C	-0.214632	0.474569	-0.236991
P	0.982864	-0.726645	-0.889969
C	0.973544	-2.005584	0.435611
C	1.196841	-1.668236	1.773047
C	1.164286	-2.644351	2.757248
C	0.907174	-3.970914	2.417318
C	0.683406	-4.314257	1.091412
C	0.718696	-3.334064	0.102937
C	-1.642513	0.128842	-0.121612
C	-2.265762	0.178757	1.154806
C	-3.590697	-0.117232	1.305062
C	-4.381658	-0.492397	0.189754
C	-3.762836	-0.576023	-1.085433
C	-2.386266	-0.266740	-1.207460
C	-5.762149	-0.793257	0.306505
C	-6.494187	-1.158270	-0.790817
C	-5.878673	-1.243395	-2.060798
C	-4.547769	-0.959481	-2.203771
C	-2.171599	3.422468	-0.178374
C	-0.583077	3.385213	2.465012
Li	3.585490	2.556076	-0.334962
H	0.543968	-3.602822	-0.933574
H	0.482141	-5.345476	0.823062
H	0.881592	-4.733488	3.187896
H	1.338274	-2.374757	3.793385
H	1.393715	-0.631962	2.031647
H	0.091679	5.644465	0.142341
H	1.555972	4.711074	0.490586
H	0.787147	4.593391	-1.102609
H	-0.934965	4.377469	2.765682
H	-1.279936	2.647673	2.872695
H	0.394453	3.218507	2.927771
H	-2.564871	4.428606	0.001022
H	-2.125497	3.267208	-1.260466
H	-2.885642	2.702694	0.227719
H	-1.662146	0.447505	2.014280
H	-4.053290	-0.073808	2.285971
H	-1.920539	-0.321639	-2.186512
H	-6.231154	-0.727214	1.283123
H	-7.550046	-1.383173	-0.689812
H	-6.467938	-1.535068	-2.923080
H	-4.072646	-1.024670	-3.177309
H	4.557494	0.989678	1.239652
H	6.976384	0.348696	0.372706
H	6.847651	-1.031535	-1.851561

6d + Li$^+$

E(ωB97X-D/6-311+G**)= -1531.604532

C	4.007272	-0.546997	0.003151
C	2.664131	-0.212143	0.192399
C	2.258816	0.256684	1.444104
C	3.174435	0.379299	2.481712
C	4.505699	0.026977	2.288082
C	4.920254	-0.438709	1.045780
P	1.500705	-0.413515	-1.232505
Si	0.831824	-2.506853	-0.712943
C	-0.529064	-3.051511	-1.920530
C	0.044342	0.603422	-0.722162
C	-1.117930	0.028214	-0.442520
C	-2.337183	-0.253009	-0.124367
C	-3.404710	-0.922570	0.203890
C	-4.734470	-0.329985	0.605740
C	-5.807405	-1.425400	0.662440
C	0.215475	2.083608	-0.699627
C	1.353221	2.700854	-1.227819
C	1.508004	4.080828	-1.177713
C	0.531609	4.876377	-0.593738
C	-0.604662	4.275380	-0.060117
C	-0.759209	2.898897	-0.110907
C	-4.577334	0.295772	2.003399
C	-5.165349	0.756455	-0.393442
C	2.302433	-3.650832	-0.917928
C	0.210866	-2.675249	1.071104
Li	-1.999189	-2.321747	-0.059450
H	4.342629	-0.889133	-0.971482
H	5.959307	-0.702981	0.882277
H	5.219446	0.124126	3.098539
H	2.846035	0.754676	3.444849
H	1.221651	0.533033	1.602032
H	-0.277891	-3.642111	1.250886
H	1.076764	-2.638314	1.737457
H	-0.444361	-1.862323	1.404524
H	2.017022	-4.688986	-0.720911
H	2.709171	-3.597490	-1.931339
H	3.098693	-3.380897	-0.218857
H	-1.012261	-3.989651	-1.617108
H	-1.292305	-2.287400	-2.113121
H	-0.069885	-3.248627	-2.892776
H	2.128605	2.101756	-1.692796
H	2.399500	4.533333	-1.597938
H	0.655038	5.952633	-0.549473
H	-1.374443	4.883752	0.402472
H	-1.645449	2.438776	0.312073
H	-6.775430	-1.011251	0.964762
H	-5.928898	-1.898212	-0.316559
H	-5.525630	-2.202039	1.379323
H	-6.132734	1.183030	-0.107346
H	-4.428102	1.562933	-0.431685
H	-5.261541	0.340143	-1.400277
H	-5.534280	0.701402	2.348886
H	-4.241640	-0.449933	2.730038

H	-3.843007	1.105491	1.985101

(Z)-7d + Li⁺

E(ωB97X-D/6-311+G**)= -1531.627265

C	-1.180040	-1.537745	-0.908999
C	-0.615435	-0.840736	0.156645
C	-0.288203	-1.537276	1.319387
C	-0.529538	-2.900385	1.417064
C	-1.089376	-3.589789	0.346578
C	-1.409709	-2.904463	-0.818796
C	-0.351914	0.626705	0.085529
C	0.984397	1.040179	0.008566
Si	1.626702	2.803782	0.230998
C	3.499087	2.755456	0.253575
P	-1.704336	1.800519	-0.025988
C	-3.202929	0.736133	-0.050238
C	-3.557057	-0.053185	1.049253
C	-4.700477	-0.839587	1.025086
C	-5.534606	-0.829008	-0.088177
C	-5.215219	-0.024461	-1.174779
C	-4.058695	0.748160	-1.154725
C	2.003611	0.043719	-0.099675
C	2.860076	-0.800508	-0.204678
C	3.833071	-1.896806	-0.301722
C	4.017584	-2.533208	1.087829
C	3.291489	-2.954028	-1.281096
C	5.179725	-1.353398	-0.807704
C	1.121771	3.856264	-1.280110
C	0.971070	3.616390	1.784285
Li	-0.076765	1.796020	-1.787959
H	-3.810702	1.362648	-2.014963
H	-5.863145	-0.003081	-2.044770
H	-6.430871	-1.439337	-0.105131
H	-4.945114	-1.458655	1.881469
H	-2.919187	-0.064118	1.926190
H	-1.464255	-1.003853	-1.810075
H	-1.852138	-3.431095	-1.656971
H	-1.277598	-4.654877	0.422884
H	-0.278435	-3.426899	2.331494
H	0.162080	-1.000733	2.147316
H	1.290933	4.661655	1.841363
H	-0.120136	3.585636	1.819474
H	1.354342	3.097013	2.667319
H	3.907615	3.764900	0.364016
H	3.857485	2.146859	1.087956
H	3.900208	2.316183	-0.663568
H	1.569891	4.849814	-1.191680
H	1.510523	3.444307	-2.223905
H	0.040341	4.020387	-1.361039
H	5.911325	-2.164305	-0.881173
H	5.067995	-0.898140	-1.795223
H	5.572439	-0.595397	-0.124920
H	4.737153	-3.356766	1.034054
H	4.387889	-1.796532	1.805258
H	3.066961	-2.924435	1.458854
H	3.990729	-3.793510	-1.354060

H	2.323647	-3.331647	-0.941181
H	3.159440	-2.525367	-2.277847

(E)-7d + Li+

E(ωB97X-D/6-311+G**)= -1531.637493

C	3.136383	-2.696686	-1.032509
C	2.308492	-1.954164	-0.183580
C	2.820637	-1.573579	1.062846
C	4.119523	-1.896820	1.431170
C	4.928944	-2.638381	0.576477
C	4.428237	-3.045359	-0.654161
P	0.605034	-1.571440	-0.739242
C	0.287971	0.120961	-0.248313
C	-0.997867	0.604169	-0.090882
C	-2.083414	-0.306177	-0.004886
C	-3.061962	-1.020936	0.112778
C	-4.288909	-1.834732	0.217573
C	-4.965191	-1.584182	1.576224
C	1.428121	1.063427	-0.103484
C	2.350593	1.260882	-1.131868
C	3.391893	2.164594	-0.977551
C	3.541635	2.867054	0.214369
C	2.644428	2.655876	1.254286
C	1.595947	1.759069	1.094333
Si	-1.547728	2.414528	-0.144938
C	-0.437878	3.474267	-1.228426
C	-3.281661	2.423861	-0.886072
C	-1.674324	3.173520	1.574456
C	-5.244858	-1.449090	-0.924426
C	-3.920843	-3.326387	0.097470
Li	-1.428990	-2.531873	-0.077792
H	2.766092	-2.991358	-2.009768
H	5.050028	-3.622763	-1.330247
H	5.942034	-2.894059	0.866905
H	4.499997	-1.574318	2.394720
H	2.197256	-1.009501	1.747062
H	2.234111	0.707964	-2.057165
H	4.093792	2.319962	-1.789389
H	4.358179	3.570648	0.332900
H	2.763384	3.185917	2.193045
H	0.898167	1.581168	1.905136
H	-0.916825	4.445253	-1.394325
H	0.541446	3.650277	-0.778331
H	-0.279492	3.004153	-2.203045
H	-3.639824	3.451353	-1.004886
H	-3.295138	1.943796	-1.869047
H	-3.990546	1.891333	-0.245321
H	-2.184239	4.141431	1.524974
H	-2.248203	2.526333	2.244923
H	-0.689876	3.337329	2.019411
H	-5.886603	-2.169558	1.654742
H	-4.303023	-1.862969	2.399741
H	-5.216200	-0.527314	1.687930
H	-6.169692	-2.030489	-0.857425
H	-5.496895	-0.388173	-0.865965
H	-4.784047	-1.632773	-1.897855

H	-4.812056	-3.955193	0.162402
H	-3.449956	-3.541743	-0.870570
H	-3.257606	-3.640017	0.915623

8d + Li$^+$

E(ωB97X-D/6-311+G**)= -1531.610068

C	3.098461	0.564892	0.705806
C	2.406638	0.644084	-0.507114
C	3.022075	1.293656	-1.582759
C	4.303429	1.812072	-1.457426
C	4.989331	1.706389	-0.251464
C	4.379420	1.085618	0.832361
C	1.055524	0.100158	-0.626087
C	0.502340	-1.075670	-0.228012
Si	1.227563	-2.652970	0.467847
C	0.393074	-4.115992	-0.438608
P	-0.480098	0.781325	-1.377202
C	-0.938779	-0.845927	-0.523520
C	-2.017261	-1.545379	-0.147604
C	-3.444411	-1.081919	-0.423352
C	-3.930364	-0.287371	0.802019
C	-0.997505	2.063300	-0.149908
C	-0.794866	1.907980	1.223938
C	-1.234938	2.871885	2.118322
C	-1.892550	4.008663	1.653165
C	-2.100834	4.175182	0.291117
C	-1.650526	3.208345	-0.604567
C	-3.622081	-0.213593	-1.679177
C	-4.331349	-2.329423	-0.578144
C	3.080804	-2.839111	0.285990
C	0.721778	-2.776267	2.287226
Li	-1.607610	-3.316546	0.680838
H	-1.812220	3.343572	-1.669594
H	-2.610806	5.058193	-0.078367
H	-2.240335	4.759972	2.353776
H	-1.072380	2.736845	3.182340
H	-0.303020	1.012130	1.590419
H	1.122829	-4.616473	-1.079782
H	0.024089	-4.899231	0.240487
H	-0.412583	-3.781265	-1.102271
H	0.921305	-3.765661	2.711611
H	1.280216	-2.045352	2.879670
H	-0.335812	-2.525750	2.444015
H	3.383707	-3.860801	0.537098
H	3.382573	-2.637776	-0.745722
H	3.632259	-2.150099	0.927942
H	2.609288	0.111919	1.562150
H	4.897304	1.018383	1.783020
H	5.986922	2.119480	-0.153131
H	4.768880	2.303822	-2.304570
H	2.488168	1.381022	-2.523087
H	-5.381304	-2.063918	-0.745597
H	-3.999438	-2.940780	-1.424850
H	-4.290146	-2.947314	0.329000
H	-4.685239	-0.028025	-1.870104
H	-3.144533	0.762526	-1.567879

H	-3.195093	-0.700514	-2.560512
H	-4.988805	-0.021920	0.696077
H	-3.817398	-0.874942	1.719443
H	-3.354466	0.633876	0.920868

9d + Li^{+}

E(ωB97X-D/6-311+G**)= -1531.643403

C	2.303329	1.522482	0.515398
C	1.718173	1.001171	-0.641533
C	2.183194	1.446557	-1.882594
C	3.229919	2.355135	-1.962517
C	3.821951	2.847168	-0.803407
C	3.350223	2.432709	0.436298
C	0.632172	0.009501	-0.536645
C	0.693566	-1.234689	0.004357
Si	2.246574	-2.091758	0.622427
C	2.706688	-1.596439	2.380596
C	-0.587777	-1.987190	0.046662
C	-1.619710	-1.248216	-0.440889
C	-3.069185	-1.710158	-0.553380
C	-3.219711	-3.162752	-0.077137
P	-1.039475	0.331626	-1.167584
C	-1.584326	1.641483	0.016579
C	-2.148872	2.819201	-0.466832
C	-2.568782	3.816014	0.411829
C	-2.422536	3.641625	1.780419
C	-1.854562	2.467684	2.272221
C	-1.439569	1.476361	1.396573
C	-3.977036	-0.825652	0.320977
C	-3.552857	-1.636818	-2.012083
C	1.869807	-3.958452	0.688055
C	3.717714	-1.829658	-0.514358
Li	-0.766789	-3.888162	0.504192
H	-2.261711	2.959221	-1.536912
H	-3.007619	4.728226	0.022436
H	-2.747935	4.416122	2.466403
H	-1.736316	2.328800	3.341634
H	-1.003932	0.556199	1.775157
H	2.780535	-4.542476	0.850762
H	1.205791	-4.191888	1.533065
H	1.426388	-4.307204	-0.253153
H	3.440806	-2.291192	2.802259
H	3.145174	-0.595667	2.403043
H	1.827665	-1.594496	3.032608
H	4.601238	-2.361502	-0.147034
H	3.499806	-2.185861	-1.525544
H	3.964986	-0.766808	-0.585754
H	1.915269	1.216733	1.480483
H	3.791861	2.826913	1.345207
H	4.637832	3.558536	-0.867074
H	3.587707	2.680163	-2.933553
H	1.727231	1.057868	-2.786701
H	-4.264218	-3.482776	-0.133208
H	-2.642242	-3.842398	-0.720273
H	-2.902924	-3.264951	0.969170
H	-5.018479	-1.161804	0.264339

H	-3.655914	-0.855262	1.366275
H	-3.941868	0.217610	-0.001405
H	-4.587344	-1.987446	-2.098379
H	-3.510903	-0.611281	-2.387115
H	-2.921301	-2.251072	-2.661052

6d' + Li^{+}

E(ωB97X-D/6-311+G**)= -1531.606687

C	4.372547	0.082208	0.434383
C	2.990780	0.285467	0.465062
C	2.377314	0.532223	1.695189
C	3.126335	0.566649	2.864483
C	4.499447	0.345924	2.825042
C	5.120948	0.102924	1.606288
P	2.060231	0.170659	-1.133731
Si	1.556729	-2.020335	-0.897064
C	0.962602	-2.450413	0.856369
C	0.452821	1.015582	-0.778743
C	0.398343	2.534125	-1.050908
C	-0.905654	3.129099	-0.510542
C	-0.620695	0.321559	-0.440753
C	-1.773682	-0.156722	-0.128821
C	-2.756279	-0.970590	0.163031
C	-4.140135	-0.561581	0.413962
C	-5.138005	-1.535117	0.534186
C	-6.461610	-1.184439	0.770373
C	-6.813331	0.153582	0.905014
C	-5.833490	1.137475	0.797320
C	-4.515456	0.784227	0.552185
C	1.582162	3.253524	-0.384856
C	0.459405	2.761714	-2.571876
C	0.237828	-2.534350	-2.156999
C	3.117303	-3.010656	-1.210924
Li	-1.235797	-2.217547	-0.112767
H	-6.100758	2.183285	0.905411
H	-7.844807	0.429428	1.094908
H	4.867225	-0.085969	-0.517524
H	6.192573	-0.058595	1.563458
H	5.081678	0.371452	3.739386
H	2.635500	0.763459	3.811500
H	1.305739	0.700623	1.732049
H	-4.857374	-2.578471	0.435683
H	-7.219435	-1.955895	0.854078
H	-3.752488	1.551901	0.468750
H	0.507845	-3.449418	0.917969
H	1.834339	-2.474294	1.514683
H	0.291455	-1.705999	1.301291
H	2.927701	-4.082024	-1.090645
H	3.499356	-2.843108	-2.221238
H	3.899250	-2.726787	-0.500888
H	-0.110978	-3.562503	-1.996151
H	-0.623251	-1.857305	-2.204778
H	0.680549	-2.513928	-3.156372
H	0.423847	3.832929	-2.796125
H	1.381126	2.356088	-2.997615
H	-0.388547	2.278785	-3.065592

H	1.530936	4.325720	-0.597619
H	1.569140	3.120296	0.699880
H	2.543391	2.888713	-0.758218
H	-0.923011	4.208044	-0.691672
H	-1.777442	2.686775	-0.998065
H	-1.000829	2.959753	0.565837

(Z)-7d' + Li⁺

E(ωB97X-D/6-311+G**)= -1531.610620

C	-4.473295	-0.806849	-1.427539
C	-3.815964	-0.144981	-0.387329
C	-4.485107	0.005917	0.831359
C	-5.748985	-0.536545	1.021802
C	-6.381894	-1.214300	-0.015038
C	-5.744335	-1.339736	-1.243545
P	-2.197867	0.667915	-0.661521
C	-0.828007	-0.137778	0.141133
C	-0.715099	-1.617512	0.580770
C	-0.037583	-2.407905	-0.558797
C	0.324619	0.691935	0.120863
Si	0.339671	2.585660	0.157880
C	0.240941	3.249916	-1.625204
C	1.647097	0.175554	0.083116
C	2.806454	-0.166763	-0.021542
C	4.159531	-0.606398	-0.100208
C	4.460296	-1.967499	-0.252267
C	5.778727	-2.392008	-0.331334
C	6.819281	-1.471462	-0.262052
C	6.531112	-0.119270	-0.108317
C	5.215487	0.312873	-0.026815
C	-0.993201	3.361213	1.224568
C	2.013417	3.136538	0.815148
C	-2.064313	-2.294844	0.854910
C	0.101903	-1.727969	1.882507
Li	-0.238221	0.732203	-1.970055
H	7.336440	0.604545	-0.049740
H	7.848400	-1.806124	-0.326068
H	-3.976867	-0.919783	-2.385980
H	-6.234919	-1.857670	-2.060652
H	-7.370165	-1.635612	0.131843
H	-6.245553	-0.422325	1.979327
H	-4.002682	0.545426	1.639970
H	3.649818	-2.685791	-0.303500
H	5.994618	-3.448177	-0.448898
H	4.991892	1.365815	0.100847
H	-0.735159	4.407824	1.417382
H	-1.977752	3.322506	0.759653
H	-1.051882	2.846422	2.188003
H	2.031893	4.225631	0.924924
H	2.218141	2.691137	1.792688
H	2.824352	2.845633	0.143322
H	0.423229	4.328402	-1.648060
H	1.023456	2.804765	-2.258292
H	-0.740927	3.089201	-2.084762
H	0.056433	-3.460562	-0.273767
H	-0.653312	-2.363557	-1.463899

H	0.960726	-2.031782	-0.785545
H	-0.387882	-1.165458	2.682619
H	0.151751	-2.776659	2.191505
H	1.120471	-1.358439	1.780847
H	-1.874213	-3.316975	1.196258
H	-2.625884	-1.782140	1.637764
H	-2.693655	-2.353591	-0.032257

(E)-7d' + Li$^+$

E(ωB97X-D/6-311+G**)= -1531.614977

C	-2.045488	-2.128888	1.002435
C	-2.006801	-2.081894	-0.398253
C	-2.698600	-3.073299	-1.103531
C	-3.406194	-4.067933	-0.438007
C	-3.453482	-4.085779	0.951542
C	-2.770839	-3.107913	1.668855
P	-1.014418	-0.832613	-1.305877
C	-1.190230	0.713359	-0.373148
C	-2.585438	1.340905	-0.150765
C	-2.688036	2.638039	-0.975986
C	-0.017170	1.391358	-0.065054
Si	0.471158	3.217144	0.257930
C	0.632569	4.100447	-1.397971
C	1.178008	0.625261	-0.075782
C	2.259762	0.069369	0.040752
C	3.377898	-0.814743	0.076676
C	4.619518	-0.468089	-0.483131
C	5.659687	-1.382039	-0.495628
C	5.489386	-2.660141	0.033979
C	4.268285	-3.016460	0.590434
C	3.220764	-2.103085	0.622871
C	-0.584052	4.238893	1.441606
C	2.180605	3.229999	1.051861
C	-2.794800	1.610106	1.350325
C	-3.759669	0.463340	-0.618864
Li	1.267514	-1.421523	-1.134925
H	4.130161	-4.002919	1.018187
H	6.307425	-3.370550	0.016254
H	-1.500817	-1.381725	1.571704
H	-2.797033	-3.113689	2.753778
H	-4.015572	-4.853917	1.470946
H	-3.937635	-4.822744	-1.008295
H	-2.697163	-3.051204	-2.189202
H	4.749523	0.521007	-0.906023
H	6.612860	-1.099615	-0.928206
H	2.281509	-2.363406	1.103271
H	-0.121563	5.230421	1.502230
H	-1.626806	4.375922	1.155201
H	-0.561914	3.810631	2.448050
H	2.426302	4.256827	1.342517
H	2.216905	2.607210	1.950173
H	2.957115	2.877577	0.369617
H	1.084226	5.087879	-1.256110
H	1.286893	3.524964	-2.059950
H	-0.322331	4.233237	-1.910620
H	-3.685326	3.073444	-0.858350

H	-1.968353	3.395702	-0.679660
H	-2.532132	2.420965	-2.036355
H	-3.864477	-0.446342	-0.029609
H	-4.686138	1.035443	-0.508382
H	-3.655805	0.180039	-1.668671
H	-3.727335	2.161539	1.508999
H	-2.870428	0.662224	1.890152
H	-1.978676	2.179960	1.788952

8d’ + Li^+

E(ωB97X-D/6-311+G**)= -1531.612252

C	-2.809920	-2.803793	-0.945637
C	-2.230092	-1.833671	-0.114459
C	-3.080001	-0.861971	0.433032
C	-4.441005	-0.853394	0.148568
C	-4.993242	-1.811281	-0.693627
C	-4.165836	-2.786997	-1.241959
C	-0.786207	-1.876691	0.186239
C	-0.021467	-0.862009	-0.235954
C	1.446609	-0.559209	-0.137887
Si	2.551838	-1.824307	0.698099
C	4.412502	-1.649764	0.852286
P	-0.299729	0.701106	-1.229401
C	1.491896	0.657135	-0.744829
C	2.558701	1.660398	-1.096316
C	3.878238	1.389765	-0.374196
C	-1.008865	1.879267	0.011779
C	-0.617111	1.891074	1.352789
C	-1.187774	2.780787	2.250556
C	-2.164955	3.676377	1.820183
C	-2.564850	3.672144	0.491131
C	-1.988850	2.776793	-0.407008
C	2.793123	1.595703	-2.617997
C	2.067590	3.071689	-0.728705
C	1.881027	-1.992466	2.471962
C	2.263780	-3.484989	-0.190839
Li	0.156802	-3.341025	1.163747
H	-5.072200	-0.086017	0.585198
H	-6.054095	-1.800479	-0.918016
H	-2.309463	2.770188	-1.443973
H	-3.326984	4.364445	0.150181
H	-2.611423	4.372386	2.522026
H	-0.875679	2.777609	3.289722
H	0.137050	1.186824	1.691424
H	2.392651	-4.350677	0.473162
H	1.292543	-3.533553	-0.695354
H	3.010915	-3.600240	-0.980859
H	2.100429	-2.968311	2.923968
H	2.367503	-1.246455	3.107022
H	0.808150	-1.775897	2.542329
H	4.784289	-2.526296	1.394819
H	4.910203	-1.630963	-0.120117
H	4.712204	-0.759732	1.408136
H	-2.173206	-3.570255	-1.379258
H	-4.580235	-3.539593	-1.905248
H	-2.664651	-0.106005	1.090202

H	4.597472	2.184921	-0.592516
H	3.734444	1.352115	0.709705
H	4.319647	0.447584	-0.701103
H	3.537972	2.340337	-2.917073
H	3.154877	0.606599	-2.913119
H	1.868756	1.795393	-3.165602
H	2.819600	3.813614	-1.014822
H	1.135768	3.318311	-1.244249
H	1.883513	3.161548	0.345123

9d’ + Li$^+$

E(ωB97X-D/6-311+G**)= -1531.638535

C	-1.000440	1.795146	1.404947
C	-1.244823	1.910208	0.034361
C	-2.064458	2.938848	-0.423028
C	-2.628431	3.844909	0.471919
C	-2.373563	3.728166	1.831240
C	-1.557009	2.699675	2.297034
P	-0.512586	0.716384	-1.169651
C	1.224854	0.551402	-0.615725
C	2.245989	1.652299	-0.900846
C	1.568631	3.011780	-1.146644
C	-0.916628	-0.875310	-0.408891
C	0.194526	-1.531256	0.033227
C	1.416586	-0.702274	-0.106120
Si	2.923893	-1.644088	0.539194
C	2.528783	-3.501541	0.490671
C	-2.256774	-1.477862	-0.373857
C	-3.192412	-1.322513	-1.407450
C	-4.416794	-1.973332	-1.368128
C	-4.750731	-2.801990	-0.298976
C	-3.851761	-2.948260	0.749190
C	-2.629204	-2.282887	0.718670
C	4.537713	-1.507761	-0.431903
C	3.218393	-1.219472	2.351054
C	3.199184	1.839940	0.288879
C	3.036940	1.284411	-2.170159
Li	-0.377800	-3.397832	0.243597
H	-5.119118	-1.839768	-2.184119
H	-5.709412	-3.307058	-0.275301
H	-2.256108	3.038809	-1.486545
H	-3.265598	4.641833	0.104081
H	-2.808391	4.435055	2.529443
H	-1.358624	2.602985	3.359122
H	-0.366504	0.989084	1.762875
H	-1.970224	-2.311974	1.584283
H	-4.116063	-3.551198	1.612000
H	-2.937639	-0.697271	-2.256699
H	3.429805	-4.093273	0.683729
H	1.797295	-3.761476	1.264093
H	2.139854	-3.795096	-0.490709
H	3.977651	-1.874784	2.790200
H	3.541902	-0.184906	2.487092
H	2.289049	-1.352404	2.913962
H	5.263451	-2.203576	0.003022
H	4.386262	-1.803322	-1.474841

H	4.993235	-0.516221	-0.430135
H	2.328505	3.755957	-1.404193
H	0.856747	2.967271	-1.976122
H	1.037000	3.365645	-0.260481
H	3.791318	2.049122	-2.385882
H	3.540002	0.323523	-2.061362
H	2.363529	1.216970	-3.029386
H	3.883603	2.671349	0.091816
H	2.636724	2.067679	1.198620
H	3.808011	0.958064	0.479902

Protonated compounds

(Z)-10a

E(ωB97X-D/6-311+G**)= -1598.445378

C	4.901496	0.529577	0.679313
C	3.805037	-0.247364	0.287545
C	3.969209	-1.629025	0.122303
C	5.206295	-2.215553	0.344836
C	6.291924	-1.437005	0.733820
C	6.135477	-0.064895	0.900386
C	2.526830	0.341873	0.056513
C	1.412567	0.757976	-0.149434
C	0.100300	1.264509	-0.385855
Si	-0.058205	3.154859	-0.177423
C	-0.717232	3.950061	-1.747590
C	-0.868179	0.384029	-0.740336
P	-2.604217	0.783478	-1.196265
C	-3.461409	-0.157779	0.136957
C	-4.343584	-1.175241	-0.219673
C	-4.981059	-1.931773	0.759180
C	-4.742410	-1.672504	2.101764
C	-3.862316	-0.657594	2.467495
C	-3.222031	0.091351	1.490623
C	-0.601485	-1.078898	-0.843062
C	-0.268064	-1.815520	0.294862
C	-0.034226	-3.180355	0.206663
C	-0.129790	-3.828125	-1.020732
C	-0.467220	-3.103405	-2.157025
C	-0.710898	-1.737827	-2.067629
C	-1.144048	3.560484	1.305188
C	1.659635	3.832035	0.151371
H	6.979129	0.545122	1.203311
H	7.257726	-1.898623	0.905524
H	-4.529449	-1.380723	-1.268616
H	-5.663450	-2.723152	0.470048
H	-5.239414	-2.260393	2.865481
H	-3.672093	-0.455259	3.515768
H	-2.519848	0.867044	1.779393
H	3.116928	-2.226406	-0.182442
H	5.325698	-3.284945	0.212108
H	4.773904	1.598305	0.807153

H	-0.974238	-1.175302	-2.957030
H	-0.543339	-3.600854	-3.117463
H	0.052994	-4.894693	-1.090289
H	0.220205	-3.740092	1.099965
H	-0.191968	-1.309218	1.250218
H	-1.120464	4.638668	1.494146
H	-2.189232	3.272725	1.170094
H	-0.766335	3.059151	2.201387
H	1.616706	4.919324	0.269982
H	2.087214	3.405116	1.062395
H	2.341820	3.604171	-0.672033
H	-0.661926	5.039961	-1.659055
H	-0.108403	3.654799	-2.607179
H	-1.754518	3.687211	-1.966031
H	-2.688444	2.063667	-0.603859

(E)-10a

E(ωB97X-D/6-311+G**)= -1598.448971

C	-4.444271	-1.736606	0.802559
C	-4.173580	-0.548694	0.112233
C	-5.224213	0.126212	-0.520956
C	-6.515255	-0.378401	-0.462443
C	-6.775645	-1.559740	0.223971
C	-5.737151	-2.236041	0.855680
C	-2.841471	-0.037417	0.055662
C	-1.704680	0.370131	0.018942
C	-0.390436	0.919754	-0.018984
Si	-0.343782	2.754780	-0.550351
C	1.219806	3.230583	-1.472287
C	0.671465	0.176866	0.370326
P	0.506524	-1.527133	1.063090
C	1.860955	-2.391631	0.168920
C	1.710249	-2.856231	-1.140019
C	2.768019	-3.471735	-1.797036
C	3.994090	-3.621161	-1.156758
C	4.153477	-3.164383	0.145591
C	3.089767	-2.561898	0.807179
C	2.042110	0.752461	0.458992
C	2.991360	0.529878	-0.539176
C	4.255549	1.094457	-0.444026
C	4.592963	1.876589	0.655570
C	3.661252	2.083443	1.665690
C	2.394809	1.519115	1.569883
C	-1.819286	3.031766	-1.678888
C	-0.528186	3.805525	0.995623
H	-7.322286	0.151636	-0.955622
H	-7.785075	-1.952759	0.267609
H	0.762101	-2.726212	-1.651563
H	2.636584	-3.831491	-2.811684
H	4.821939	-4.096363	-1.671245
H	5.106442	-3.279694	0.649634
H	3.220271	-2.207664	1.824557

H	-3.631589	-2.257304	1.295705
H	-5.935234	-3.157364	1.391670
H	-5.016655	1.045404	-1.056388
H	2.730803	-0.079739	-1.396866
H	4.980436	0.923424	-1.231906
H	5.580002	2.319442	0.727294
H	3.919348	2.685012	2.530222
H	1.664106	1.676396	2.356038
H	1.075272	4.219017	-1.921589
H	2.092398	3.279495	-0.817340
H	1.442647	2.525639	-2.277542
H	-1.822079	4.060608	-2.052211
H	-1.790498	2.359052	-2.541102
H	-2.760377	2.857396	-1.151457
H	-0.668218	4.859577	0.735634
H	-1.393019	3.484803	1.583541
H	0.360836	3.730149	1.627576
H	-0.563242	-1.965432	0.248114

3a

E(ωB97X-D/6-311+G**)= -1598.480360

C	-3.597367	0.342722	-0.376340
C	-3.228667	-0.988848	-0.155374
C	-4.236152	-1.960916	-0.149783
C	-5.560412	-1.623284	-0.390164
C	-5.908596	-0.298674	-0.630082
C	-4.922178	0.680848	-0.615979
C	-1.841857	-1.414100	0.074981
C	-0.713793	-0.808686	-0.309081
C	0.704979	-1.173381	-0.097268
Si	1.395927	-2.822262	0.510235
C	3.228521	-2.975510	0.137970
P	-0.235204	0.736539	-1.228631
C	-0.522419	2.100792	-0.018075
C	-0.896302	3.352745	-0.505802
C	-1.119419	4.414401	0.366707
C	-0.973298	4.228631	1.734471
C	-0.601149	2.981591	2.230884
C	-0.375402	1.926177	1.360054
C	1.301440	-0.047247	-0.573424
C	2.685140	0.421964	-0.582244
C	3.241328	0.984505	-1.735159
C	4.553533	1.435929	-1.735875
C	5.322867	1.353665	-0.579668
C	4.771018	0.820305	0.579390
C	3.461195	0.360718	0.578539
C	1.076120	-2.958933	2.357904
C	0.489124	-4.192127	-0.404162
H	-5.184829	1.719854	-0.781462
H	-6.942789	-0.031407	-0.815497
H	-1.018686	3.497386	-1.574693
H	-1.408553	5.383550	-0.024390

H	-1.149743	5.052467	2.417237
H	-0.487412	2.834659	3.299222
H	-0.088943	0.954276	1.750470
H	0.895155	-5.168831	-0.122470
H	-0.582418	-4.199635	-0.190998
H	0.610222	-4.077535	-1.485184
H	1.405628	-3.927165	2.747254
H	1.610560	-2.177632	2.906857
H	0.010514	-2.850517	2.580831
H	3.543642	-4.013206	0.288826
H	3.442237	-2.706485	-0.900406
H	3.844856	-2.339858	0.776217
H	-3.971880	-2.997601	0.035053
H	-6.322225	-2.394848	-0.388935
H	-2.854487	1.128398	-0.329344
H	3.020745	-0.036891	1.486663
H	5.359184	0.769692	1.488977
H	6.345175	1.714678	-0.579817
H	4.977117	1.858510	-2.640071
H	2.641320	1.053177	-2.636548
H	-1.738829	-2.361164	0.603853

2a

E(ωB97X-D/6-311+G**)= -1598.511925

C	-0.830645	3.257233	-0.414053
C	-0.888830	1.948134	0.059808
C	-1.107115	1.721550	1.420568
C	-1.266282	2.790219	2.290294
C	-1.201144	4.096324	1.811580
C	-0.980913	4.328867	0.461215
P	-0.612011	0.568160	-1.121727
C	-1.547321	-0.819716	-0.425881
C	-3.012524	-0.914518	-0.429561
C	-3.679306	-1.607474	0.587781
C	-5.063939	-1.698139	0.597700
C	-5.813311	-1.085682	-0.400877
C	-5.164592	-0.381451	-1.408347
C	-3.779071	-0.294805	-1.422319
C	-0.678099	-1.761724	0.009158
C	0.742861	-1.401517	-0.016216
Si	2.088662	-2.629346	0.498666
C	1.380138	-4.358678	0.277386
C	0.939752	-0.147562	-0.504110
C	2.208392	0.597406	-0.586066
C	2.740207	0.998651	-1.813972
C	3.949718	1.678432	-1.869849
C	4.637211	1.982867	-0.699744
C	4.100974	1.614404	0.528581
C	2.892689	0.932054	0.584898
C	3.615771	-2.446040	-0.575715
C	2.536313	-2.391942	2.310005
H	-5.739470	0.101806	-2.190537

H	-6.895315	-1.152542	-0.389866
H	-0.664692	3.440603	-1.470331
H	-0.929091	5.343922	0.083566
H	-1.323614	4.929949	2.494224
H	-1.440014	2.606554	3.344818
H	-1.151227	0.703538	1.793935
H	-3.104483	-2.060726	1.388269
H	-5.560990	-2.237582	1.396501
H	-3.281523	0.244728	-2.221410
H	2.152501	-5.111145	0.464637
H	0.557890	-4.554217	0.972322
H	1.004487	-4.510259	-0.738792
H	3.203541	-3.191210	2.648392
H	3.048436	-1.439946	2.471187
H	1.644618	-2.408405	2.943864
H	4.336885	-3.233446	-0.333345
H	3.361909	-2.537285	-1.635714
H	4.106639	-1.480918	-0.431249
H	2.460161	0.661160	1.541284
H	4.621235	1.863986	1.446729
H	5.582550	2.511654	-0.745673
H	4.360275	1.967977	-2.830819
H	2.211402	0.752252	-2.728425
H	-1.008806	-2.743377	0.337439

(Z)-10b

E(ωB97X-D/6-311+G**)= -1752.068112

C	-6.684543	-1.694678	0.271755
C	-5.394583	-1.123785	0.139563
C	-5.234126	0.271701	0.350652
C	-6.371810	1.050379	0.686774
C	-7.605202	0.470398	0.808142
C	-7.763964	-0.918329	0.598498
C	-4.254271	-1.900546	-0.195934
C	-3.022194	-1.326755	-0.312998
C	-2.854416	0.072604	-0.099825
C	-3.946949	0.845785	0.222702
C	-1.547283	0.628390	-0.219368
C	-0.397919	0.981996	-0.325769
C	0.963777	1.390783	-0.438872
Si	1.262417	3.254691	-0.167178
C	2.156092	4.006270	-1.639201
C	1.882650	0.436877	-0.727575
P	3.684800	0.679887	-1.008756
C	4.303560	-0.429413	0.325928
C	4.960353	-1.605441	-0.030876
C	5.380411	-2.503225	0.945164
C	5.154032	-2.226305	2.286667
C	4.503972	-1.051175	2.652877
C	4.076072	-0.161123	1.677808
C	1.496721	-0.994080	-0.892786

C	1.078219	-1.738255	0.211271
C	0.738836	-3.076054	0.070048
C	0.813041	-3.687900	-1.177320
C	1.233036	-2.954629	-2.280177
C	1.581669	-1.616246	-2.137591
C	-0.414034	4.077753	-0.000245
C	2.220210	3.530762	1.428877
H	3.547369	0.741673	1.966946
H	4.324279	-0.833655	3.699979
H	5.481465	-2.925314	3.048195
H	5.883144	-3.419114	0.655555
H	5.135437	-1.825352	-1.078734
H	2.255564	5.087779	-1.501320
H	3.157900	3.598457	-1.791264
H	1.585260	3.837685	-2.556958
H	-0.290419	5.156232	0.139449
H	-1.026599	3.916139	-0.891267
H	-0.965205	3.682637	0.857102
H	2.292461	4.602814	1.638629
H	1.701541	3.060936	2.269892
H	3.237258	3.133193	1.394261
H	-2.150207	-1.919233	-0.567698
H	-4.377148	-2.966298	-0.359390
H	-3.821827	1.911140	0.384252
H	-6.804325	-2.761061	0.109701
H	-8.745879	-1.366842	0.698725
H	-6.246306	2.116263	0.847331
H	-8.467342	1.074945	1.066596
H	1.909991	-1.045907	-3.000030
H	1.292614	-3.424715	-3.255478
H	0.549680	-4.733931	-1.288246
H	0.418803	-3.643387	0.937011
H	1.022035	-1.258773	1.181792
H	3.846468	1.905164	-0.322319

(E)-10b

E(ωB97X-D/6-311+G**)= -1752.071264

C	7.231034	-1.154789	0.662780
C	5.854318	-0.821410	0.624503
C	5.398224	0.112161	-0.343648
C	6.335358	0.680537	-1.244925
C	7.658671	0.338439	-1.184270
C	8.112309	-0.589844	-0.219412
C	4.912742	-1.388626	1.522468
C	3.591619	-1.051662	1.462670
C	3.127037	-0.116516	0.494724
C	4.024496	0.447264	-0.385324
C	1.742961	0.225971	0.435310
C	0.569275	0.505817	0.373301
C	-0.781451	0.938664	0.231905
Si	-0.922070	2.684384	-0.543847
C	-0.969732	3.944648	0.848151

C	-1.816557	0.172752	0.646923
P	-1.650945	-1.446951	1.516124
C	-2.435459	-2.531216	0.248508
C	-1.845350	-2.734953	-1.002200
C	-2.481731	-3.506392	-1.964375
C	-3.721406	-4.078423	-1.690849
C	-4.314309	-3.883825	-0.450359
C	-3.671246	-3.117005	0.516902
C	-3.226396	0.646164	0.540126
C	-3.728154	1.546820	1.480641
C	-5.026448	2.029837	1.374203
C	-5.844346	1.604269	0.333597
C	-5.360576	0.685650	-0.591116
C	-4.061932	0.204563	-0.487202
C	0.628197	2.953730	-1.571131
C	-2.418112	2.894685	-1.656458
H	-4.139565	-2.963963	1.483489
H	-5.278796	-4.328989	-0.232996
H	-4.221372	-4.676899	-2.444380
H	-2.013754	-3.658619	-2.930729
H	-0.888870	-2.272805	-1.225054
H	-0.863462	4.958867	0.450132
H	-1.916591	3.894020	1.391945
H	-0.156721	3.774057	1.559604
H	0.571209	3.918383	-2.085189
H	1.525049	2.952264	-0.946649
H	0.744578	2.172719	-2.328305
H	-2.319087	3.842490	-2.196591
H	-2.485567	2.092437	-2.396139
H	-3.356396	2.918589	-1.098390
H	2.877710	-1.487252	2.151813
H	5.259262	-2.101615	2.263426
H	3.673930	1.158515	-1.125637
H	7.576316	-1.867919	1.404318
H	9.163814	-0.850967	-0.180436
H	5.984512	1.393176	-1.984264
H	8.366393	0.777891	-1.878110
H	-3.684705	-0.502498	-1.217086
H	-5.994691	0.346265	-1.402477
H	-6.855790	1.984815	0.245550
H	-5.398394	2.739743	2.104623
H	-3.087630	1.874026	2.292513
H	-0.306443	-1.707710	1.164344

3b

E(ωB97X-D/6-311+G**)= -1752.104195

C	4.709505	-1.173940	0.144722
C	4.131217	0.110431	-0.023070
C	4.959912	1.195934	-0.407451
C	6.341173	0.963370	-0.620457
C	6.872588	-0.288012	-0.456086
C	6.047518	-1.368590	-0.068165

C	2.750184	0.335948	0.191017
C	2.189618	1.579056	0.019905
C	3.034934	2.665829	-0.339440
C	4.370820	2.479305	-0.552492
C	0.751180	1.822600	0.211069
C	-0.243890	1.031144	-0.197948
C	-1.713556	1.125492	-0.047105
Si	-2.706483	2.628314	0.519693
C	-4.530311	2.453927	0.112469
P	-0.388578	-0.555363	-1.160496
C	0.134361	-1.880841	0.011581
C	0.963037	-2.894571	-0.469450
C	1.438624	-3.884881	0.384925
C	1.084006	-3.870074	1.727617
C	0.247511	-2.868633	2.214432
C	-0.224693	-1.881627	1.361327
C	-2.066717	-0.088409	-0.551364
C	-3.338483	-0.802897	-0.631685
C	-3.703492	-1.477811	-1.800869
C	-4.911940	-2.155603	-1.875765
C	-5.764826	-2.187710	-0.777133
C	-5.398262	-1.544790	0.399708
C	-4.192506	-0.861133	0.473020
C	-2.457142	2.844537	2.370236
C	-2.034770	4.122936	-0.402102
H	1.250377	-2.899590	-1.516161
H	2.087380	-4.664623	0.001227
H	1.455726	-4.637840	2.397165
H	-0.030588	-2.856572	3.262515
H	-0.866296	-1.094340	1.745550
H	-2.588843	5.024384	-0.121740
H	-0.977094	4.300307	-0.193899
H	-2.140629	3.986395	-1.482237
H	-2.967326	3.741310	2.735180
H	-2.851680	1.986116	2.922106
H	-1.395364	2.937913	2.616484
H	-5.019022	3.427586	0.222395
H	-4.675708	2.118616	-0.918247
H	-5.043973	1.745236	0.764661
H	2.598547	3.652269	-0.460102
H	5.000943	3.315851	-0.837624
H	2.140234	-0.494181	0.524357
H	6.973915	1.794250	-0.916293
H	7.930927	-0.455194	-0.622053
H	4.067629	-1.997628	0.442503
H	6.482358	-2.353485	0.060891
H	-3.894165	-0.378542	1.397481
H	-6.050992	-1.581777	1.264861
H	-6.707131	-2.720600	-0.835397
H	-5.188276	-2.663125	-2.793147
H	-3.036915	-1.455689	-2.656770
H	0.491814	2.751090	0.719342

2b

E(ωB97X-D/6-311+G**)= -1752.134778

C	0.164252	3.273430	-0.144258
C	-0.008560	1.959675	0.289141
C	-0.230470	1.710567	1.645279
C	-0.279836	2.761405	2.549845
C	-0.097902	4.070289	2.112082
C	0.127291	4.324852	0.766131
P	0.128134	0.605971	-0.945330
C	-0.765582	-0.787777	-0.206209
C	-2.225878	-0.860382	-0.083839
C	-2.817664	-1.738036	0.869351
C	-4.172270	-1.815336	1.013328
C	-5.037817	-1.014175	0.224540
C	-4.462782	-0.123422	-0.718405
C	-3.053716	-0.070347	-0.848811
C	-5.319913	0.686395	-1.506981
C	-6.679212	0.612463	-1.363034
C	-7.250309	-0.275206	-0.423235
C	-6.447487	-1.068328	0.352221
C	0.125670	-1.754610	0.119347
C	1.543382	-1.414806	-0.025443
Si	2.914047	-2.667913	0.343344
C	4.308978	-2.515271	-0.902103
C	1.717270	-0.150915	-0.495286
C	2.987785	0.573076	-0.679778
C	3.767252	0.900699	0.431877
C	4.979285	1.561145	0.276580
C	5.423663	1.914212	-0.992265
C	4.641089	1.616472	-2.102984
C	3.427588	0.959584	-1.948067
C	3.575258	-2.430396	2.087504
C	2.151588	-4.383931	0.217376
H	0.331581	3.475640	-1.197006
H	0.268875	5.342508	0.419804
H	-0.134321	4.889102	2.822018
H	-0.458603	2.560355	3.600339
H	-0.364362	0.690401	1.988889
H	-2.176050	-2.340071	1.502800
H	-4.604276	-2.488118	1.747378
H	-2.629486	0.604620	-1.586270
H	-6.881781	-1.751341	1.075400
H	-8.328262	-0.325583	-0.318593
H	-4.879523	1.368190	-2.227339
H	-7.324383	1.236346	-1.971353
H	2.923070	-5.150802	0.337750
H	1.397997	-4.553036	0.992525
H	1.674700	-4.539213	-0.754890
H	4.258957	-3.243400	2.352338
H	4.124852	-1.490126	2.177835
H	2.764824	-2.421201	2.822369

H	5.039274	-3.316077	-0.747023
H	3.929729	-2.597833	-1.924708
H	4.830522	-1.559298	-0.814300
H	2.823808	0.717827	-2.816165
H	4.979415	1.893716	-3.095286
H	6.371835	2.425229	-1.115496
H	5.574037	1.806609	1.149515
H	3.406570	0.641492	1.420842
H	-0.183378	-2.742674	0.448329

(Z)-10b'

E(ωB97X-D/6-311+G**)= -1752.068963

C	-3.404161	0.649882	1.587016
C	-3.465296	0.077404	0.314355
C	-4.035264	-1.185357	0.165252
C	-4.535737	-1.867999	1.269392
C	-4.476719	-1.288459	2.529634
C	-3.912533	-0.025878	2.687544
P	-2.734657	0.900192	-1.163737
C	-0.970827	0.828348	-0.650055
C	-0.115655	1.868096	-0.491225
Si	-0.491180	3.728540	-0.668788
C	1.135180	4.648895	-0.514570
C	-0.525434	-0.577780	-0.431928
C	-0.211445	-1.008160	0.884580
C	0.183262	-2.292157	1.123330
C	0.294977	-3.228740	0.063048
C	-0.022165	-2.807523	-1.254597
C	-0.441827	-1.469842	-1.469357
C	0.089692	-3.738781	-2.318005
C	0.493800	-5.025220	-2.082354
C	0.807049	-5.444829	-0.769738
C	0.709559	-4.567108	0.276367
C	1.235684	1.554374	-0.159400
C	2.372025	1.261706	0.123582
C	3.675613	0.795480	0.467551
C	3.898699	-0.581777	0.599342
C	5.158155	-1.053986	0.938002
C	6.207183	-0.164262	1.148056
C	5.992328	1.203717	1.017343
C	4.735349	1.684548	0.679237
C	-1.235999	4.098148	-2.353506
C	-1.629187	4.291709	0.720127
H	6.807607	1.900009	1.178906
H	7.190754	-0.537050	1.411069
H	-4.080084	-1.642784	-0.817450
H	-4.971415	-2.852597	1.142323
H	-4.868594	-1.818393	3.390679
H	-3.861923	0.428821	3.670733
H	-2.941400	1.622793	1.718860
H	3.074709	-1.267154	0.432778
H	5.322281	-2.121010	1.037951

H	4.562861	2.749403	0.573858
H	-0.682546	-1.152726	-2.479564
H	0.415683	-2.612028	2.134055
H	-0.292836	-0.296395	1.697784
H	-1.761065	5.377623	0.676428
H	-2.621514	3.837267	0.672550
H	-1.190154	4.048742	1.692336
H	0.972876	5.723958	-0.640605
H	1.593191	4.484565	0.464381
H	1.850558	4.320667	-1.273374
H	-1.368316	5.178203	-2.474051
H	-0.566700	3.753488	-3.147135
H	-2.208574	3.625751	-2.508254
H	-0.150318	-3.413935	-3.325421
H	0.574805	-5.729262	-2.902941
H	1.124276	-6.466859	-0.595395
H	0.947212	-4.886024	1.286341
H	-3.012856	2.235795	-0.791899

(E)-10b’

E(ωB97X-D/6-311+G**)= -1752.072884

C	-0.270464	3.170576	1.032735
C	-0.724429	2.708084	-0.204458
C	-2.011818	3.047285	-0.621230
C	-2.836320	3.820249	0.188146
C	-2.374456	4.278348	1.415775
C	-1.087451	3.957202	1.834583
P	0.291444	1.594424	-1.262044
C	-0.009291	0.014939	-0.356949
C	0.954267	-0.836020	0.061764
Si	0.653386	-2.520116	0.916130
C	2.165722	-3.582556	0.583669
C	-1.458653	-0.259244	-0.168805
C	-2.045053	-0.070494	1.109025
C	-3.379716	-0.287378	1.303303
C	-4.213087	-0.696719	0.231427
C	-3.636201	-0.867532	-1.054562
C	-2.250830	-0.629085	-1.225461
C	-4.465689	-1.273621	-2.131511
C	-5.802073	-1.494146	-1.938913
C	-6.377124	-1.320652	-0.658946
C	-5.601370	-0.931779	0.399192
C	2.330967	-0.507742	-0.101421
C	3.513355	-0.285330	-0.211395
C	4.905527	-0.005202	-0.360175
C	5.336848	1.286805	-0.685444
C	6.689268	1.556736	-0.833608
C	7.628572	0.545733	-0.660493
C	7.208426	-0.740427	-0.337520
C	5.857438	-1.017309	-0.187208
C	0.499720	-2.229487	2.765941
C	-0.868944	-3.407742	0.270003

H	7.937401	-1.531642	-0.203353
H	8.684956	0.758381	-0.778910
H	-2.379097	2.690393	-1.577864
H	-3.839632	4.064407	-0.142499
H	-3.014591	4.886279	2.045435
H	-0.721092	4.314952	2.790663
H	0.724340	2.904818	1.375811
H	4.601224	2.071211	-0.820439
H	7.011650	2.560842	-1.085248
H	5.525523	-2.019014	0.060190
H	-1.810831	-0.761476	-2.209221
H	-3.820875	-0.136886	2.283251
H	-1.416557	0.266733	1.925322
H	-0.866362	-4.433934	0.652966
H	-1.798248	-2.930752	0.587761
H	-0.871203	-3.457335	-0.822212
H	2.035166	-4.572731	1.031503
H	2.333316	-3.714580	-0.489042
H	3.065401	-3.131500	1.009901
H	0.486842	-3.182779	3.304001
H	1.343446	-1.642567	3.140286
H	-0.422878	-1.695930	3.008153
H	-4.020803	-1.406264	-3.112426
H	-6.427355	-1.804083	-2.768698
H	-7.437542	-1.497997	-0.519514
H	-6.040148	-0.797754	1.382723
H	1.556057	1.903530	-0.708479

3b'

E(ωB97X-D/6-311+G**)= -1752.102976

C	1.062379	-1.739629	1.565845
C	0.874518	-2.036088	0.213696
C	0.790093	-3.370922	-0.181692
C	0.884408	-4.394146	0.757335
C	1.074775	-4.088234	2.097799
C	1.166064	-2.758112	2.501102
P	0.712543	-0.728095	-1.077797
C	1.607868	0.740795	-0.373037
C	2.873420	1.103287	-0.138932
C	4.112463	0.372581	-0.430718
C	5.315909	1.088533	-0.448114
C	6.521621	0.462794	-0.730226
C	6.552452	-0.901857	-0.993049
C	5.368090	-1.630000	-0.964212
C	4.162344	-1.003459	-0.683137
C	-0.535478	0.408177	-0.335790
C	0.332423	1.419152	-0.054467
Si	0.054978	3.223320	0.434384
C	1.221657	4.292101	-0.581220
C	-1.970808	0.234106	-0.136795
C	-2.750399	-0.369049	-1.096385
C	-4.141646	-0.548407	-0.909728

C	-4.737447	-0.117601	0.305284
C	-3.911975	0.471719	1.297017
C	-2.574304	0.640830	1.084693
C	-6.130207	-0.301204	0.491672
C	-6.895311	-0.882258	-0.483223
C	-6.303320	-1.311164	-1.693236
C	-4.960865	-1.148393	-1.900824
C	-1.704054	3.751180	0.049594
C	0.433129	3.425714	2.264874
H	5.382883	-2.698052	-1.151152
H	7.492953	-1.395963	-1.208987
H	0.650227	-3.611081	-1.231164
H	0.811418	-5.428306	0.439497
H	1.153425	-4.883169	2.831122
H	1.318951	-2.518828	3.547659
H	1.136079	-0.703660	1.882312
H	1.017514	5.351299	-0.394682
H	2.273082	4.109741	-0.347407
H	1.083525	4.107385	-1.650356
H	0.307177	4.466428	2.579241
H	-0.229182	2.807879	2.878506
H	1.463002	3.129948	2.486586
H	-1.798749	4.833741	0.183369
H	-1.959119	3.518146	-0.988264
H	-2.444395	3.266503	0.688305
H	5.299551	2.154833	-0.244231
H	7.439312	1.040399	-0.742595
H	3.262227	-1.602419	-0.633497
H	-1.946800	1.070342	1.858007
H	-4.359945	0.779780	2.236327
H	-2.297967	-0.703811	-2.025194
H	-6.581996	0.028141	1.421846
H	-7.960062	-1.017633	-0.330673
H	-6.920052	-1.770512	-2.457483
H	-4.503339	-1.477227	-2.828274
H	3.025658	2.078378	0.322010

2b'

E(ωB97X-D/6-311+G**)= -1752.134874

C	4.208283	-0.858536	-1.646702
C	3.807412	0.087770	-0.697357
C	4.793355	0.690518	0.093840
C	6.134172	0.378720	-0.079176
C	6.519508	-0.554683	-1.035349
C	5.549628	-1.174330	-1.814598
C	2.390563	0.421822	-0.502754
C	1.894113	1.616776	-0.099920
C	0.443296	1.704131	0.072924
Si	-0.396930	3.334358	0.541894
C	-0.605072	3.465408	2.406750
C	-0.173402	0.530799	-0.235484
P	1.007786	-0.662337	-0.927831

C	0.990913	-1.994513	0.334284
C	1.876021	-2.035130	1.412737
C	1.786171	-3.056533	2.350772
C	0.805967	-4.035038	2.231323
C	-0.080325	-3.997588	1.161375
C	0.020129	-2.990705	0.209675
C	-1.597418	0.186956	-0.097910
C	-2.341797	-0.248682	-1.168276
C	-3.713287	-0.572557	-1.028836
C	-4.325058	-0.458901	0.247408
C	-3.532792	-0.039119	1.346062
C	-2.212330	0.268154	1.180458
C	-5.700179	-0.775799	0.382524
C	-6.432944	-1.184342	-0.698797
C	-5.824368	-1.298776	-1.969753
C	-4.498829	-1.000067	-2.130566
C	0.733381	4.725647	-0.031240
C	-2.057588	3.502330	-0.315727
H	5.838345	-1.906372	-2.560492
H	7.566980	-0.800170	-1.168265
H	-0.668189	-2.975166	-0.629260
H	-0.846373	-4.758263	1.060974
H	0.735753	-4.826543	2.969054
H	2.483994	-3.083493	3.180436
H	2.636128	-1.270065	1.520341
H	4.500709	1.396889	0.863214
H	6.881172	0.858224	0.543887
H	3.461410	-1.337670	-2.271230
H	0.254689	5.695124	0.138455
H	1.684749	4.730825	0.508946
H	0.953476	4.643430	-1.099656
H	-0.938329	4.470943	2.683207
H	-1.346939	2.755186	2.780603
H	0.339633	3.271784	2.923441
H	-2.464720	4.504443	-0.146621
H	-1.953253	3.358398	-1.394946
H	-2.786595	2.775087	0.048627
H	-1.605844	0.562770	2.028937
H	-3.990095	0.026430	2.328111
H	-1.881821	-0.323650	-2.148831
H	-6.164416	-0.686605	1.359455
H	-7.484800	-1.420886	-0.584516
H	-6.415130	-1.623859	-2.818808
H	-4.029366	-1.086323	-3.105168
H	2.533918	2.481189	0.054947

(Z)-10c

E(ωB97X-D/6-311+G**)= -2072.841531

C	-6.703543	-1.621572	0.414132
C	-5.402671	-1.076211	0.280638
C	-5.219518	0.319767	0.468652
C	-6.344581	1.124796	0.783635

C	-7.589005	0.569239	0.905961
C	-7.770684	-0.819926	0.719048
C	-4.274858	-1.879221	-0.034750
C	-3.032409	-1.329554	-0.157698
C	-2.843306	0.070518	0.030396
C	-3.922081	0.868798	0.336905
C	-1.529427	0.604955	-0.106296
C	-0.379143	0.941940	-0.247630
C	0.985241	1.313081	-0.413517
Si	1.361108	3.181744	-0.203936
C	2.027187	3.883344	-1.809896
C	1.870558	0.327058	-0.701676
P	3.677621	0.680408	-0.866936
C	4.311778	-0.480916	0.409035
C	4.823683	-1.740941	0.097915
C	5.267669	-2.590495	1.104675
C	5.191547	-2.196274	2.434955
C	4.680443	-0.942965	2.755610
C	4.251862	-0.088226	1.748294
C	1.425039	-1.075323	-0.846829
C	1.418836	-2.065696	0.091711
C	0.889826	-3.295131	-0.396110
C	0.510850	-3.223072	-1.701330
S	0.786397	-1.653116	-2.354659
C	-0.266948	4.016204	0.209719
C	2.560070	3.459386	1.214613
H	3.857396	0.889386	2.007269
H	4.620239	-0.628393	3.791568
H	5.530131	-2.862700	3.220339
H	5.661281	-3.567871	0.848581
H	4.859301	-2.070041	-0.934903
H	1.777996	-1.913609	1.101010
H	0.801382	-4.192931	0.201321
H	0.090502	-4.001446	-2.320388
H	2.129138	4.970592	-1.731419
H	3.007165	3.467622	-2.054191
H	1.346400	3.669499	-2.638842
H	-0.113429	5.092575	0.336190
H	-1.004144	3.868258	-0.583969
H	-0.693510	3.622075	1.136190
H	2.615083	4.527405	1.449238
H	2.225818	2.937911	2.116606
H	3.567337	3.117418	0.966229
H	-2.169729	-1.941417	-0.398248
H	-4.415324	-2.945631	-0.178178
H	-3.778901	1.934500	0.479953
H	-6.840922	-2.688383	0.270127
H	-8.761588	-1.248406	0.818603
H	-6.201352	2.190958	0.926359
H	-8.442030	1.193368	1.146969
H	3.911103	-0.162805	-1.983678

(E)-10c

E(ωB97X-D/6-311+G**)= -2072.842891

C	7.179076	-1.812790	0.410216
C	5.860378	-1.297725	0.354683
C	5.638374	-0.037262	-0.261092
C	6.744900	0.667323	-0.802413
C	8.007267	0.143726	-0.734362
C	8.227153	-1.110739	-0.121348
C	4.751762	-2.000473	0.895055
C	3.491228	-1.483609	0.826305
C	3.260790	-0.221509	0.207592
C	4.322115	0.479279	-0.320506
C	1.929863	0.287723	0.141295
C	0.782296	0.663733	0.099660
C	-0.560868	1.133901	0.064283
Si	-0.735180	3.026938	-0.125153
C	-0.822142	3.771596	1.596969
C	-1.567186	0.243032	0.233688
C	-2.975011	0.674816	0.322119
C	-3.603809	1.230537	1.400402
C	-4.975870	1.524512	1.160608
C	-5.368932	1.179538	-0.096672
S	-4.082085	0.481849	-0.998337
P	-1.183353	-1.530102	0.621240
C	-2.703728	-2.395941	0.073104
C	-2.845702	-2.942381	-1.203512
C	-4.022381	-3.585193	-1.569218
C	-5.076613	-3.675889	-0.667900
C	-4.944404	-3.136995	0.606759
C	-3.760249	-2.514690	0.978870
C	0.820964	3.626997	-0.988102
C	-2.229127	3.549713	-1.129993
H	-3.660302	-2.102789	1.977913
H	-5.761287	-3.207625	1.316174
H	-5.997934	-4.169273	-0.956879
H	-4.119715	-4.007545	-2.563251
H	-2.037845	-2.855706	-1.922622
H	-3.092207	1.416884	2.335774
H	-5.636976	1.971894	1.890828
H	-6.343804	1.293858	-0.546276
H	-0.733890	4.861654	1.548743
H	-1.772841	3.531640	2.079894
H	-0.012903	3.395358	2.229237
H	0.773123	4.709727	-1.140674
H	1.713707	3.404229	-0.398112
H	0.940249	3.152491	-1.966516
H	-2.153571	4.623610	-1.332251
H	-2.273701	3.030515	-2.090727
H	-3.169239	3.371040	-0.603297
H	2.646575	-2.019835	1.242763
H	4.920620	-2.962235	1.368168
H	4.150076	1.439992	-0.793850

H	7.344893	-2.775964	0.881819
H	9.231767	-1.515538	-0.073550
H	6.573365	1.629989	-1.272919
H	8.845791	0.689341	-1.152089
H	-0.378625	-1.808262	-0.510303

3c

E(ωB97X-D/6-311+G**)= -2072.877367

C	-4.267998	-0.876760	0.492346
C	-3.338158	-0.775884	-0.508150
S	-3.808593	-1.736814	-1.876562
C	-5.258536	-2.235227	-1.097645
C	-5.370417	-1.705435	0.152634
C	-2.077364	-0.068389	-0.490417
P	-0.405133	-0.571182	-1.092543
C	0.094916	-1.857519	0.132025
C	0.820386	-2.956755	-0.327066
C	1.268338	-3.930163	0.561401
C	0.992855	-3.809902	1.917051
C	0.261675	-2.719970	2.383351
C	-0.186832	-1.752613	1.496129
C	-0.244467	1.043492	-0.181820
C	-1.710822	1.155614	-0.018747
Si	-2.690530	2.666139	0.548846
C	-2.429190	2.879002	2.397986
C	0.760688	1.838255	0.197102
C	2.195437	1.586191	-0.002534
C	3.046252	2.674940	-0.343337
C	4.380446	2.485649	-0.563428
C	4.963416	1.196990	-0.442908
C	4.129674	0.108705	-0.077824
C	2.750090	0.336601	0.141920
C	6.343396	0.961813	-0.660901
C	6.868969	-0.294701	-0.518594
C	6.039112	-1.378062	-0.149007
C	4.702084	-1.181084	0.067429
C	-2.015932	4.156682	-0.377040
C	-4.516436	2.499984	0.149800
H	1.045941	-3.045848	-1.385020
H	1.832792	-4.779625	0.192993
H	1.345049	-4.563363	2.612831
H	0.046836	-2.623705	3.441870
H	-0.746542	-0.898138	1.864425
H	-5.948826	-2.889389	-1.608748
H	-6.203993	-1.906402	0.812207
H	-4.141145	-0.392611	1.451673
H	-2.568203	5.059621	-0.097935
H	-0.957938	4.333432	-0.170074
H	-2.123014	4.018333	-1.456784
H	-2.934726	3.776162	2.768348
H	-2.821984	2.019984	2.950187
H	-1.365474	2.969408	2.637099

H	-5.000747	3.475397	0.263386
H	-4.666758	2.166905	-0.880868
H	-5.030275	1.792337	0.802741
H	2.614984	3.665871	-0.444156
H	5.014122	3.324204	-0.834359
H	2.138338	-0.497720	0.461173
H	6.979848	1.794784	-0.942511
H	7.926467	-0.463887	-0.687796
H	4.056822	-2.006912	0.351755
H	6.469585	-2.366888	-0.036623
H	0.511248	2.780119	0.684960

2c

E(ωB97X-D/6-311+G**)= -2072.908022

C	0.121025	3.283955	-0.034925
C	-0.028476	1.963161	0.385578
C	-0.260684	1.696616	1.736579
C	-0.343816	2.737875	2.649539
C	-0.184995	4.054185	2.225179
C	0.050664	4.325851	0.884370
P	0.147685	0.626273	-0.859975
C	1.737946	-0.123438	-0.397100
C	2.994701	0.607885	-0.523960
C	3.895530	0.894612	0.463778
C	5.020438	1.633534	0.002856
C	4.954278	1.909642	-1.328671
S	3.519487	1.275477	-2.038767
C	1.571816	-1.401319	0.042412
Si	2.947519	-2.662650	0.367646
C	3.655107	-2.454491	2.097270
C	0.156905	-1.753194	0.166746
C	-0.738901	-0.789115	-0.156388
C	-2.199982	-0.880072	-0.061062
C	-2.797111	-1.769049	0.877953
C	-4.153034	-1.862882	0.997863
C	-5.013997	-1.069097	0.196717
C	-4.433489	-0.168464	-0.733458
C	-3.023201	-0.097302	-0.838335
C	-6.424935	-1.140289	0.299741
C	-7.223440	-0.354200	-0.487188
C	-6.646830	0.542855	-1.414655
C	-5.286267	0.633360	-1.534816
C	2.169787	-4.371965	0.244396
C	4.302783	-2.502699	-0.918734
H	5.662513	2.455247	-1.933973
H	5.835724	1.953653	0.637981
H	3.733212	0.605212	1.493143
H	0.296405	3.498469	-1.083919
H	0.174698	5.349463	0.549055
H	-0.247339	4.865636	2.941725
H	-0.530247	2.523902	3.696104
H	-0.376916	0.670759	2.069443

H	-2.159004	-2.365738	1.519979
H	-4.589843	-2.543310	1.721918
H	-2.593959	0.585045	-1.566043
H	-6.863629	-1.830457	1.013378
H	-8.302346	-0.417598	-0.401265
H	-4.841554	1.322181	-2.245741
H	-7.288825	1.160891	-2.032202
H	2.939238	-5.144015	0.342498
H	1.431789	-4.543225	1.033962
H	1.672325	-4.517270	-0.719041
H	4.310170	-3.296067	2.344805
H	4.245307	-1.538755	2.179138
H	2.861835	-2.415502	2.849563
H	5.043765	-3.297713	-0.786586
H	3.891974	-2.589066	-1.928647
H	4.818820	-1.542545	-0.846299
H	-0.147361	-2.749547	0.473771

(Z)-10c’

E(ωB97X-D/6-311+G**)= -2072.841783

C	-0.056150	-4.613059	0.245551
C	-0.234226	-3.221953	0.042520
C	-0.477918	-2.743716	-1.271120
C	-0.533389	-3.672768	-2.340833
C	-0.356339	-5.011073	-2.114857
C	-0.115218	-5.487308	-0.806302
C	-0.662340	-1.352862	-1.475470
C	-0.589862	-0.467154	-0.431596
C	-0.347151	-0.953182	0.880493
C	-0.177492	-2.287520	1.108683
C	-0.818042	0.990797	-0.638431
P	-2.557691	1.324954	-1.138995
C	-3.386190	0.550097	0.312082
C	-3.989025	-0.696569	0.151647
C	-4.552188	-1.352449	1.240686
C	-4.528953	-0.760173	2.496644
C	-3.937229	0.487879	2.664573
C	-3.362182	1.135664	1.579495
C	0.179321	1.894024	-0.479431
Si	0.072469	3.788768	-0.659859
C	-0.932712	4.509526	0.757751
C	1.472001	1.397689	-0.144617
C	2.569179	0.982642	0.145161
C	3.806827	0.404426	0.493001
S	3.933830	-1.315965	0.723817
C	5.607634	-1.190460	1.090705
C	6.047821	0.099360	1.038246
C	5.018729	1.014403	0.695955
C	1.818166	4.465759	-0.560711
C	-0.660276	4.257738	-2.324994
H	-4.005142	-1.164796	-0.826835
H	-5.008868	-2.326373	1.105789

H	-4.970769	-1.269011	3.346154
H	-3.915967	0.953048	3.643919
H	-2.877895	2.096679	1.721010
H	-0.853292	-0.991779	-2.481567
H	0.002630	-2.650398	2.115379
H	-0.306203	-0.244209	1.699269
H	-0.854948	5.601494	0.752663
H	-1.993744	4.254575	0.702012
H	-0.546975	4.152118	1.717039
H	1.806725	5.551439	-0.699460
H	2.275028	4.250397	0.408739
H	2.458475	4.030441	-1.332701
H	-0.583162	5.340149	-2.470202
H	-0.108042	3.773333	-3.135527
H	-1.712791	3.984520	-2.428343
H	-0.716788	-3.303568	-3.344832
H	-0.399109	-5.712996	-2.940147
H	0.024932	-6.549461	-0.639875
H	0.129422	-4.975510	1.251669
H	6.177463	-2.077466	1.324250
H	7.072007	0.386235	1.235311
H	5.154534	2.082732	0.596517
H	-2.654767	2.668239	-0.707338

(E)-10c’

E(ωB97X-D/6-311+G**)= -2072.845698

C	-5.445855	-1.486625	1.001093
C	-4.075294	-1.144773	0.872195
C	-3.655003	-0.406762	-0.265329
C	-4.618264	-0.026776	-1.234989
C	-5.934117	-0.367712	-1.081615
C	-6.352377	-1.107651	0.049037
C	-2.290482	-0.058712	-0.396145
C	-1.369215	-0.422042	0.552639
C	-1.798353	-1.142545	1.696870
C	-3.109844	-1.500935	1.846956
C	0.062923	-0.037491	0.427833
P	0.461256	1.628311	1.126353
C	-0.807589	2.647928	0.271549
C	-0.635527	3.119518	-1.031804
C	-1.653923	3.817431	-1.669301
C	-2.861349	4.041679	-1.015613
C	-3.039302	3.583587	0.283944
C	-2.013278	2.901673	0.926902
C	1.004416	-0.906673	-0.005932
Si	0.698309	-2.714883	-0.548422
C	-0.967980	-2.983601	-1.368218
C	2.377919	-0.535022	-0.020808
C	3.561370	-0.287790	-0.042730
C	4.935820	0.030100	-0.056238
S	5.520921	1.499487	0.668807
C	7.141726	1.124680	0.239393

C	7.232136	-0.063337	-0.423284
C	5.971084	-0.691111	-0.594557
C	2.053826	-3.154365	-1.771552
C	0.849203	-3.797460	0.978393
H	0.294498	2.926717	-1.556549
H	-1.507658	4.180089	-2.680916
H	-3.660592	4.575318	-1.517958
H	-3.978620	3.754158	0.797559
H	-2.160016	2.540739	1.939761
H	-1.976219	0.510581	-1.264721
H	-3.427262	-2.057868	2.722705
H	-1.066295	-1.407880	2.451868
H	-0.975492	-3.978895	-1.825412
H	-1.795920	-2.930415	-0.658292
H	-1.152942	-2.250296	-2.157398
H	1.914670	-4.175640	-2.139481
H	2.043530	-2.480236	-2.633039
H	3.041812	-3.089227	-1.309180
H	0.790134	-4.857199	0.710631
H	1.804311	-3.628593	1.483948
H	0.045715	-3.585817	1.689057
H	-4.291644	0.545212	-2.097395
H	-6.663891	-0.070622	-1.826338
H	-7.398496	-1.370313	0.159562
H	-5.765323	-2.050975	1.871389
H	7.939782	1.803281	0.501043
H	8.165902	-0.477413	-0.778961
H	5.819977	-1.638344	-1.094101
H	1.554129	1.934277	0.281778

3c’

E(ωB97X-D/6-311+G**)= -2072.877473

C	-4.848201	-1.527961	-1.739355
C	-4.081677	-0.756065	-0.828237
C	-4.726724	-0.189946	0.303368
C	-6.113570	-0.412621	0.488995
C	-6.827301	-1.160722	-0.407947
C	-6.186589	-1.725365	-1.534472
C	-3.953330	0.570226	1.218021
C	-2.619470	0.769267	1.010233
C	-1.966316	0.227256	-0.131118
C	-2.695707	-0.537180	-1.012932
C	-0.537079	0.439726	-0.327660
C	0.295565	1.493055	-0.083450
Si	-0.049792	3.308987	0.316778
C	0.014898	3.579699	2.177490
P	0.747071	-0.677151	-1.033071
C	0.993679	-1.929236	0.296387
C	0.961005	-1.598789	1.652924
C	1.141991	-2.576972	2.619106
C	1.357262	-3.899795	2.240763
C	1.391351	-4.239218	0.894978

C	1.210672	-3.256116	-0.072905
C	1.588674	0.846393	-0.373806
C	2.848386	1.213414	-0.112013
C	4.072090	0.479429	-0.373324
S	4.145407	-0.998117	-1.284348
C	5.850812	-1.110294	-1.078707
C	6.342915	-0.066224	-0.356139
C	5.327674	0.842226	0.044071
C	1.276808	4.361131	-0.500386
C	-1.719663	3.811540	-0.375487
H	0.794356	-0.567876	1.950222
H	1.114908	-2.311522	3.670236
H	1.497639	-4.663935	2.997375
H	1.559726	-5.268156	0.597197
H	1.243726	-3.520195	-1.125291
H	0.994728	5.417648	-0.449132
H	2.250033	4.257750	-0.014075
H	1.401432	4.096546	-1.553937
H	-0.094741	4.642834	2.414107
H	-0.781570	3.041041	2.698484
H	0.970321	3.240875	2.589213
H	-1.878220	4.883526	-0.219604
H	-1.763386	3.618982	-1.451349
H	-2.549038	3.276061	0.090788
H	-2.033522	1.328202	1.730724
H	-4.437061	0.984381	2.096837
H	-2.207541	-0.972986	-1.879691
H	-6.601694	0.020754	1.355995
H	-7.888268	-1.324750	-0.257137
H	-6.762801	-2.317949	-2.236050
H	-4.354268	-1.960818	-2.603202
H	6.389996	-1.941058	-1.508175
H	7.391746	0.053349	-0.119741
H	5.509524	1.738261	0.624208
H	3.009548	2.169218	0.382341

2c'

E(ωB97X-D/6-311+G**)= -2072.907680

C	-4.452484	-1.104435	-2.154756
C	-3.688663	-0.659406	-1.044826
C	-4.317088	-0.556859	0.224184
C	-5.686686	-0.902679	0.344129
C	-6.398400	-1.327623	-0.744823
C	-5.773275	-1.430511	-2.008716
C	-3.546463	-0.119468	1.331245
C	-2.231588	0.217742	1.179701
C	-1.601575	0.149970	-0.092157
C	-2.323201	-0.305291	-1.169667
C	-0.186952	0.536522	-0.216833
P	1.036590	-0.635216	-0.876944
C	1.083122	-1.911029	0.442318
C	1.670024	-1.678408	1.687877

C	1.635594	-2.659121	2.669012
C	1.006879	-3.875885	2.421290
C	0.417431	-4.111300	1.186415
C	0.462825	-3.135246	0.196648
C	0.386629	1.733028	0.084375
Si	-0.513999	3.336714	0.535129
C	-2.169973	3.443492	-0.340657
C	1.841382	1.697789	-0.080657
C	2.374413	0.513148	-0.466475
C	3.792391	0.227095	-0.612861
C	4.834527	0.759691	0.100266
C	6.101086	0.243738	-0.286019
C	6.009077	-0.682838	-1.279789
S	4.376901	-0.933961	-1.764328
C	-0.745308	3.468440	2.397175
C	0.575993	4.761122	-0.034042
H	0.010566	-3.325769	-0.770924
H	-0.072473	-5.057911	0.988081
H	0.980310	-4.639540	3.190526
H	2.099778	-2.474208	3.631447
H	2.153365	-0.727667	1.885219
H	0.067601	5.716078	0.131318
H	1.523849	4.796040	0.511203
H	0.804360	4.684000	-1.101111
H	-1.117421	4.462406	2.665598
H	-1.465666	2.733918	2.766107
H	0.200264	3.310904	2.924448
H	-2.610239	4.433556	-0.183972
H	-2.049855	3.294412	-1.417513
H	-2.879159	2.696218	0.022330
H	-1.641570	0.527352	2.034480
H	-4.015707	-0.063766	2.308248
H	-1.851426	-0.370631	-2.145224
H	-6.163508	-0.822631	1.315778
H	-7.446230	-1.586338	-0.642021
H	-6.347217	-1.768968	-2.864037
H	-3.970353	-1.182255	-3.123841
H	4.689218	1.481447	0.893628
H	7.038584	0.542361	0.163968
H	6.806302	-1.232602	-1.756696
H	2.455055	2.581146	0.070431

(Z)-10d

E(ωB97X-D/6-311+G**)= -1524.673208

C	-3.775779	-0.843236	-0.454400
C	-3.104586	0.323783	-0.086797
C	-3.270377	0.817805	1.209142
C	-4.079417	0.152532	2.121751
C	-4.745695	-1.008372	1.744445
C	-4.596209	-1.501589	0.453782
P	-1.962733	1.220476	-1.216569

C	-0.382249	0.394629	-0.737164
C	0.686311	1.106762	-0.310274
C	1.850629	0.369648	0.070505
C	2.807818	-0.292383	0.383195
C	3.896782	-1.210014	0.738374
C	5.255102	-0.563550	0.421443
C	-0.335959	-1.093690	-0.794025
C	0.008697	-1.745200	-1.977561
C	0.077947	-3.132171	-2.025044
C	-0.204777	-3.884336	-0.890364
C	-0.558565	-3.241137	0.290583
C	-0.624615	-1.854784	0.338815
Si	0.824357	3.009367	-0.162764
C	-0.475299	3.681995	1.016067
C	2.524711	3.377814	0.538990
C	0.670033	3.801078	-1.856802
C	3.807213	-1.529868	2.241384
C	3.719591	-2.503516	-0.078613
H	-3.641401	-1.252781	-1.449715
H	-5.109132	-2.409068	0.155270
H	-5.380473	-1.526340	2.454724
H	-4.194358	0.544142	3.126422
H	-2.756945	1.725795	1.509950
H	0.239606	-1.157886	-2.860433
H	0.357552	-3.626109	-2.949177
H	-0.151018	-4.966715	-0.926936
H	-0.786056	-3.820671	1.178562
H	-0.901031	-1.352049	1.259079
H	-0.257104	4.728060	1.254632
H	-1.475288	3.637550	0.578547
H	-0.480027	3.119689	1.954698
H	2.665941	4.459060	0.634915
H	2.653409	2.927990	1.527301
H	3.315391	2.985488	-0.106132
H	0.859239	4.877393	-1.789817
H	1.399038	3.375263	-2.552349
H	-0.328524	3.658221	-2.276167
H	6.066068	-1.251362	0.680163
H	5.332729	-0.323819	-0.641907
H	5.389564	0.359374	0.991242
H	4.603732	-2.226417	2.520958
H	3.915108	-0.622320	2.840541
H	2.844884	-1.988398	2.481173
H	4.497899	-3.225323	0.189147
H	2.740805	-2.950736	0.113195
H	3.790732	-2.297011	-1.149258
H	-2.218186	0.427494	-2.365971

(E)-10d

E(ωB97X-D/6-311+G**)= -1524.675813

C	1.842572	-2.699953	-0.990979
C	1.941124	-2.151760	0.290335

C	3.199523	-2.045448	0.882875
C	4.339510	-2.458096	0.202656
C	4.230927	-2.999512	-1.072112
C	2.979222	-3.126483	-1.666021
P	0.473908	-1.515284	1.199425
C	0.289288	0.147536	0.415822
C	-0.898642	0.641880	0.003261
C	-2.084385	-0.152036	0.079871
C	-3.125007	-0.760590	0.130243
C	-4.382473	-1.517307	0.198153
C	-5.494099	-0.719785	-0.505368
C	1.524035	0.980544	0.436515
C	2.473613	0.887591	-0.581608
C	3.608622	1.685623	-0.559135
C	3.816137	2.578499	0.486822
C	2.886640	2.661590	1.516819
C	1.749978	1.861553	1.494474
Si	-1.226702	2.416154	-0.621843
C	0.226580	3.201002	-1.513294
C	-2.671632	2.317966	-1.818510
C	-1.705962	3.456199	0.867324
C	-4.192190	-2.874932	-0.501732
C	-4.756116	-1.743368	1.673990
H	0.871434	-2.782109	-1.468166
H	2.888537	-3.553773	-2.658702
H	5.119139	-3.326174	-1.601546
H	5.312317	-2.356722	0.670511
H	3.290737	-1.619983	1.876917
H	2.313963	0.191384	-1.397051
H	4.333861	1.611353	-1.361617
H	4.701785	3.203915	0.501291
H	3.044197	3.351762	2.338273
H	1.020975	1.922548	2.295573
H	-0.113714	4.133923	-1.975834
H	1.056646	3.436611	-0.843999
H	0.607771	2.553395	-2.307235
H	-2.898225	3.310184	-2.221249
H	-2.438623	1.658286	-2.659766
H	-3.569906	1.930770	-1.331723
H	-2.017982	4.459319	0.559769
H	-2.534951	2.995403	1.412303
H	-0.863695	3.561670	1.556823
H	-6.437145	-1.273509	-0.463125
H	-5.642443	0.248915	-0.021422
H	-5.243444	-0.543340	-1.554308
H	-5.120727	-3.452114	-0.453416
H	-3.925749	-2.736179	-1.552337
H	-3.399992	-3.452609	-0.019235
H	-5.692006	-2.306960	1.739768
H	-3.974502	-2.305134	2.190909
H	-4.889812	-0.789741	2.190269
H	-0.516823	-2.192244	0.450355

3d

E(ωB97X-D/6-311+G**)= -1524.707037

C	3.027393	0.645003	0.759023
C	2.347493	0.688401	-0.461448
C	2.936647	1.363763	-1.534605
C	4.189434	1.945723	-1.399178
C	4.865001	1.879901	-0.185042
C	4.277265	1.232914	0.896014
C	1.026533	0.076825	-0.591384
C	0.520605	-1.119659	-0.188273
Si	1.339100	-2.694595	0.453171
C	0.548951	-4.151922	-0.433033
P	-0.520311	0.695720	-1.375072
C	-0.912311	-0.902734	-0.501690
C	-1.997625	-1.565768	-0.108545
C	-3.460278	-1.256722	-0.334633
C	-4.038261	-0.716017	0.986776
C	-1.102661	1.983126	-0.184681
C	-0.989055	1.826386	1.198780
C	-1.453889	2.809007	2.060050
C	-2.037917	3.965588	1.548686
C	-2.152706	4.134049	0.175648
C	-1.685367	3.145917	-0.686719
C	-3.699398	-0.231651	-1.446291
C	-4.170219	-2.570853	-0.700445
C	3.179130	-2.697937	0.084075
C	1.032392	-2.825750	2.304399
H	-1.780742	3.277750	-1.760077
H	-2.605625	5.033055	-0.227393
H	-2.401420	4.732889	2.223326
H	-1.360950	2.675841	3.132315
H	-0.539496	0.923147	1.600277
H	1.026209	-5.088288	-0.127147
H	-0.520548	-4.234518	-0.225104
H	0.668293	-4.052333	-1.515804
H	1.451218	-3.751749	2.710446
H	1.491696	-1.988345	2.838309
H	-0.038878	-2.814164	2.526611
H	3.589640	-3.695402	0.272180
H	3.365662	-2.451025	-0.965063
H	3.734558	-1.983352	0.694305
H	2.557518	0.158645	1.607361
H	4.790219	1.192586	1.850529
H	5.840402	2.341038	-0.079019
H	4.640036	2.454494	-2.244055
H	2.410689	1.418334	-2.482023
H	-5.243973	-2.403817	-0.829469
H	-3.773726	-2.982227	-1.633322
H	-4.038856	-3.322868	0.084180
H	-4.774264	-0.117569	-1.617018
H	-3.311084	0.753475	-1.178240

H	-3.237700	-0.545665	-2.386106
H	-5.113621	-0.535973	0.886566
H	-3.889989	-1.429883	1.803097
H	-3.553863	0.225142	1.261290
H	-1.831075	-2.446225	0.515827

2d

E(ωB97X-D/6-311+G**)= -1524.737785

C	-1.483993	1.409675	1.399968
C	-1.618239	1.554848	0.017348
C	-2.166370	2.727977	-0.493967
C	-2.582238	3.743490	0.363918
C	-2.450348	3.589536	1.736474
C	-1.898948	2.419474	2.254338
P	-1.058475	0.239754	-1.147479
C	-1.639457	-1.319208	-0.398414
C	-3.062582	-1.836573	-0.510746
C	-4.049747	-0.794057	0.041514
C	-0.568767	-1.983353	0.085800
C	0.726364	-1.286962	0.042248
Si	2.315797	-2.115253	0.645909
C	3.789950	-1.594806	-0.392005
C	0.622084	-0.056395	-0.518716
C	1.672734	0.970920	-0.638488
C	2.228256	1.535530	0.511981
C	3.235280	2.487434	0.418845
C	3.694462	2.896892	-0.827535
C	3.129915	2.359602	-1.979515
C	2.120886	1.410301	-1.886320
C	2.616534	-1.702903	2.456657
C	2.074971	-3.975411	0.485619
C	-3.389015	-2.102315	-1.992446
C	-3.235742	-3.142931	0.273815
H	-2.270793	2.850306	-1.566882
H	-3.008193	4.653203	-0.044339
H	-2.774114	4.378566	2.406187
H	-1.791340	2.297642	3.326600
H	-1.053044	0.497383	1.801376
H	2.998202	-4.501686	0.747424
H	1.287616	-4.343135	1.150379
H	1.809568	-4.254809	-0.538310
H	3.462517	-2.276306	2.848953
H	2.842822	-0.641639	2.588605
H	1.739368	-1.940394	3.066241
H	4.669973	-2.182293	-0.110311
H	3.597862	-1.764029	-1.455380
H	4.030485	-0.537256	-0.260913
H	1.854621	1.226437	1.481687
H	3.658417	2.914616	1.321297
H	4.481837	3.638494	-0.901519
H	3.479799	2.679270	-2.954924
H	1.687352	0.985089	-2.785045

H	-4.405850	-2.495856	-2.091323
H	-3.319405	-1.185611	-2.583776
H	-2.693798	-2.832023	-2.416690
H	-4.273771	-3.480684	0.204785
H	-2.601757	-3.939770	-0.124802
H	-2.994827	-3.006047	1.331989
H	-5.074218	-1.171069	-0.037036
H	-3.842900	-0.571952	1.091893
H	-3.996041	0.147068	-0.512342
H	-0.640350	-2.990886	0.483394

(Z)-10d’

E(ωB97X-D/6-311+G**)= -1524.663980

C	4.490904	-1.619673	0.153915
C	3.980386	-0.324616	0.308135
C	4.831156	0.694960	0.753466
C	6.161510	0.422220	1.035444
C	6.661556	-0.866330	0.877611
C	5.822844	-1.884844	0.437018
C	2.612318	-0.047137	0.014087
C	1.447981	0.154276	-0.237647
C	0.101281	0.536746	-0.518418
Si	-0.081551	2.441437	-0.326943
C	-1.369999	2.895766	0.967856
C	-0.848949	-0.374138	-0.864058
C	-0.583131	-1.899938	-0.955436
C	-1.843692	-2.710702	-1.312607
P	-2.528077	0.157254	-1.408896
C	-3.549145	-0.214680	0.087209
C	-3.021325	-0.233951	1.379410
C	-3.833422	-0.520401	2.469183
C	-5.185870	-0.785114	2.281514
C	-5.721497	-0.766979	0.999659
C	-4.905210	-0.490263	-0.091917
C	-0.085418	-2.439478	0.399327
C	0.449617	-2.165903	-2.068468
C	1.552760	3.128540	0.297627
C	-0.402477	3.265454	-1.988258
H	6.812409	1.218292	1.379027
H	7.702549	-1.075469	1.096198
H	-5.325154	-0.495998	-1.092879
H	-6.774406	-0.975797	0.846184
H	-5.819417	-1.009586	3.132425
H	-3.408865	-0.535963	3.466993
H	-1.967423	-0.028214	1.531259
H	3.832307	-2.409166	-0.189707
H	6.209373	-2.890073	0.312305
H	4.435523	1.696792	0.874459
H	-1.394975	3.985330	1.075612
H	-2.383612	2.556599	0.748742
H	-1.083477	2.477798	1.937803
H	1.451150	4.208758	0.445625

H	1.843115	2.679681	1.251008
H	2.361761	2.956109	-0.416613
H	-0.310697	4.351001	-1.878313
H	0.352091	2.941622	-2.711579
H	-1.384631	3.054495	-2.415221
H	0.624769	-3.242827	-2.158324
H	0.071659	-1.804954	-3.029606
H	1.404087	-1.678940	-1.872210
H	-0.853575	-2.305436	1.166293
H	0.114292	-3.512054	0.310629
H	0.827598	-1.951232	0.735605
H	-1.574799	-3.769468	-1.365131
H	-2.627267	-2.612709	-0.557160
H	-2.257385	-2.429718	-2.284019
H	-2.387192	1.539298	-1.176687

(E)-10d’

E(ωB97X-D/6-311+G**)= -1524.657139

C	-3.816674	-1.729766	1.148237
C	-3.523131	-0.771465	0.170005
C	-4.488551	-0.467551	-0.797440
C	-5.719343	-1.107964	-0.782289
C	-6.003117	-2.058113	0.193093
C	-5.048702	-2.366802	1.156588
C	-2.253589	-0.118167	0.165542
C	-1.159392	0.394148	0.185307
C	0.074418	1.119276	0.188034
Si	-0.356656	2.844332	-0.564401
C	0.954766	3.785893	-1.539221
C	1.191079	0.553774	0.725841
C	2.553332	1.272010	0.855567
C	3.200543	1.390083	-0.537090
P	1.144841	-1.165693	1.427755
C	2.093253	-2.078348	0.124685
C	3.069148	-2.995011	0.513745
C	3.788152	-3.711945	-0.437818
C	3.537436	-3.516356	-1.789523
C	2.562558	-2.606522	-2.188388
C	1.845387	-1.893420	-1.237302
C	3.552277	0.513945	1.752293
C	2.370400	2.650419	1.514291
C	-1.733839	2.576451	-1.820343
C	-1.035957	3.907179	0.828530
H	-6.460220	-0.864828	-1.535499
H	-6.965691	-2.556695	0.202157
H	1.091378	-1.178687	-1.551566
H	2.362218	-2.452541	-3.243035
H	4.098904	-4.071987	-2.532452
H	4.544351	-4.421694	-0.121235
H	3.269985	-3.147323	1.569600
H	-3.070409	-1.965412	1.897989
H	-5.265512	-3.107289	1.918146

H	-4.261535	0.270733	-1.557857
H	0.480560	4.719684	-1.862241
H	1.867406	4.048157	-1.005085
H	1.234109	3.241189	-2.445825
H	-1.879063	3.500312	-2.389761
H	-1.488922	1.779442	-2.528236
H	-2.679333	2.321626	-1.338779
H	-1.459263	4.832709	0.425165
H	-1.836900	3.369414	1.344459
H	-0.279912	4.174571	1.570025
H	3.345151	3.130877	1.643375
H	1.751105	3.327628	0.933503
H	1.911420	2.540473	2.500640
H	3.810156	-0.467992	1.351606
H	4.480222	1.090821	1.806194
H	3.175467	0.388286	2.769901
H	4.119132	1.982874	-0.475864
H	3.460691	0.399075	-0.917443
H	2.536396	1.859853	-1.259199
H	-0.134061	-1.529034	0.962538

3d’

E(ωB97X-D/6-311+G**)= -1524.704732

C	-2.129051	2.602504	-0.544293
C	-1.160145	1.723652	-0.064144
C	-0.994608	1.584515	1.315772
C	-1.779800	2.314390	2.195482
C	-2.743587	3.193082	1.705937
C	-2.918690	3.336281	0.336484
P	-0.140427	0.784263	-1.279158
C	1.546915	0.721126	-0.538451
C	2.601079	1.800196	-0.506576
C	1.918824	3.177329	-0.549681
C	0.067589	-0.862543	-0.457572
C	1.501304	-0.598485	-0.191494
Si	2.767218	-1.865822	0.425360
C	2.134494	-3.595543	0.039581
C	-0.776418	-1.810178	-0.040214
C	-2.231276	-1.837616	-0.245888
C	-3.030624	-2.522266	0.676244
C	-4.412881	-2.541531	0.552306
C	-5.027088	-1.881456	-0.505695
C	-4.244548	-1.214040	-1.441674
C	-2.862541	-1.196964	-1.318161
C	4.426844	-1.703354	-0.446472
C	2.972556	-1.693656	2.285807
C	3.441958	1.716930	0.774552
C	3.498910	1.653166	-1.749248
H	-4.712882	-0.710813	-2.280376
H	-6.106487	-1.895198	-0.605963
H	-2.267949	2.713475	-1.615236
H	-3.670107	4.016645	-0.048525

H	-3.358135	3.762195	2.394739
H	-1.645387	2.198220	3.265360
H	-0.247094	0.895820	1.698378
H	2.960987	-4.309511	0.113764
H	1.358562	-3.922908	0.735888
H	1.724302	-3.658729	-0.971854
H	3.643238	-2.466540	2.674554
H	3.380870	-0.719421	2.565245
H	2.006099	-1.806564	2.786585
H	5.071012	-2.532602	-0.135637
H	4.300813	-1.772938	-1.531055
H	4.958829	-0.775808	-0.227732
H	-2.557861	-3.035748	1.507838
H	-5.012021	-3.072587	1.283759
H	-2.267420	-0.699429	-2.075625
H	4.161562	2.540810	0.804020
H	2.804390	1.788420	1.660075
H	4.006088	0.786067	0.836329
H	4.252673	2.447164	-1.764956
H	4.011539	0.689863	-1.756156
H	2.905908	1.728493	-2.664893
H	2.673221	3.969473	-0.557255
H	1.308243	3.289763	-1.451186
H	1.269989	3.328959	0.316805
H	-0.382933	-2.612245	0.579813

2d’

E(ωB97X-D/6-311+G**)= -1524.729404

C	-3.195326	-1.340394	-1.352291
C	-2.218714	-1.623720	-0.391490
C	-2.552225	-2.487949	0.658157
C	-3.810244	-3.068226	0.730473
C	-4.768919	-2.785270	-0.236530
C	-4.456715	-1.915787	-1.274956
C	-0.884570	-1.015541	-0.453850
C	0.274269	-1.570246	-0.040306
C	1.482418	-0.733677	-0.114129
Si	3.075755	-1.587531	0.484091
C	4.153066	-0.566918	1.644877
P	-0.521849	0.596498	-1.175104
C	1.219029	0.508672	-0.620829
C	2.152413	1.703666	-0.819255
C	2.387632	2.375936	0.547323
C	-1.320095	1.744746	0.026059
C	-2.143708	2.764793	-0.443886
C	-2.762948	3.636674	0.447806
C	-2.562711	3.490307	1.813327
C	-1.743106	2.469911	2.290102
C	-1.127136	1.601508	1.401664
C	3.490884	1.286620	-1.446581
C	1.529141	2.752619	-1.757883
C	2.563620	-3.089059	1.504117

C	4.072083	-2.231425	-0.975432
H	-5.198012	-1.685962	-2.032304
H	-5.753704	-3.234567	-0.176508
H	-2.300665	2.880942	-1.511308
H	-3.403727	4.426972	0.072912
H	-3.045097	4.168229	2.508721
H	-1.587690	2.350962	3.356680
H	-0.487050	0.805175	1.768939
H	-1.821015	-2.687865	1.433920
H	-4.048597	-3.735042	1.551897
H	-2.955311	-0.674865	-2.174920
H	4.914804	-2.833598	-0.620456
H	3.443714	-2.873512	-1.600166
H	4.467690	-1.436591	-1.610215
H	3.454420	-3.526948	1.965567
H	1.872003	-2.821772	2.308779
H	2.094525	-3.869523	0.898681
H	4.943973	-1.219210	2.030495
H	4.634778	0.300082	1.191406
H	3.570617	-0.218890	2.503424
H	2.220444	3.592697	-1.872304
H	1.334200	2.337014	-2.749945
H	0.594238	3.154771	-1.359394
H	4.096302	2.175677	-1.649428
H	4.077594	0.641456	-0.795857
H	3.327869	0.761893	-2.391759
H	3.090795	3.208877	0.442321
H	1.450046	2.769175	0.948746
H	2.795472	1.672173	1.273134
H	0.315726	-2.593236	0.319312

Desilylated ring systems

5a

E(ωB97X-D/6-311+G**)= -1189.793657

C	0.350296	0.670139	0.210346
C	0.078981	-0.023279	1.395068
C	1.085314	-0.834803	1.931995
C	2.331901	-0.922275	1.328619
C	2.595391	-0.208233	0.165034
C	1.596561	0.581650	-0.393631
C	-1.210427	0.064722	2.090967
C	-2.085120	1.075263	2.060206
C	-3.373147	1.258485	2.723330
H	-3.849326	0.601851	3.448619
P	-2.207846	2.777704	1.308354
C	-2.691484	2.464541	-0.446307
C	-2.148687	3.289968	-1.431398
C	-2.466222	3.095773	-2.772744
C	-3.330805	2.073043	-3.138036
C	-3.880330	1.246233	-2.161244
C	-3.563767	1.441879	-0.825153

C	-3.779205	2.451299	2.230258
C	-4.985219	3.248468	2.403091
C	-5.099443	4.486237	1.763730
C	-6.243521	5.258787	1.912509
C	-7.292632	4.803930	2.701582
C	-7.193696	3.569894	3.339309
C	-6.052561	2.797815	3.190191
H	1.785164	1.125786	-1.312502
H	3.567917	-0.275249	-0.309472
H	-1.467166	4.085907	-1.146954
H	-2.036127	3.742427	-3.529513
H	-3.577889	1.916386	-4.182179
H	-4.556857	0.447786	-2.445082
H	-3.994257	0.794647	-0.067603
H	0.886343	-1.393577	2.841177
H	3.099895	-1.549242	1.767974
H	-0.425779	1.256795	-0.265253
H	-5.985836	1.835865	3.686885
H	-8.011125	3.209405	3.953690
H	-8.186102	5.406706	2.818423
H	-6.315109	6.217028	1.410617
H	-4.283605	4.844634	1.144219
H	-1.458762	-0.783644	2.728768

4a

E(ωB97X-D/6-311+G**)= -1189.826431

C	0.083844	0.582910	0.085544
C	0.091683	0.052848	1.373576
C	1.306807	-0.308517	1.959189
C	2.494446	-0.140816	1.263980
C	2.480407	0.389891	-0.024041
C	1.276782	0.751166	-0.612256
P	-1.503765	-0.152874	2.259647
C	-1.172440	0.545011	3.905054
C	-0.988454	1.977618	4.161187
C	-0.210938	2.413567	5.240975
C	-0.034330	3.766813	5.489185
C	-0.617813	4.714874	4.655305
C	-1.377999	4.295889	3.570061
C	-1.561866	2.941925	3.325261
C	-1.207019	-0.449393	4.824616
C	-1.339623	-1.784663	4.275907
H	-1.403986	-2.660830	4.913575
C	-1.409493	-1.842178	2.924075
C	-1.487775	-3.049953	2.095213
C	-2.156205	-3.042335	0.866136
C	-2.220562	-4.187367	0.084019
C	-1.618890	-5.364095	0.513546
C	-0.943164	-5.383301	1.729131
C	-0.871561	-4.237611	2.507821
H	-1.834833	5.027091	2.912513
H	-0.476933	5.772408	4.847529
H	-0.857413	0.865401	-0.374355
H	1.261586	1.164570	-1.614503
H	3.410765	0.520052	-0.565620
H	3.434830	-0.423331	1.724049

H	1.314693	-0.721738	2.962938
H	0.273442	1.681194	5.877642
H	0.571049	4.084035	6.331182
H	-2.172347	2.625056	2.486159
H	-0.315985	-4.252922	3.439184
H	-0.459718	-6.293430	2.066793
H	-1.671925	-6.258695	-0.096415
H	-2.747443	-4.161360	-0.863317
H	-2.644957	-2.133621	0.530663
H	-1.166810	-0.272725	5.894949

5b

E(ωB97X-D/6-311+G**)= -1343.417423

C	0.501286	0.729334	0.203445
C	0.303580	0.297866	1.540470
C	1.387622	-0.288869	2.243537
C	2.639070	-0.420180	1.591841
C	2.803366	0.010524	0.302516
C	1.722053	0.590467	-0.399643
C	-0.948274	0.434035	2.186280
C	-1.135383	0.023715	3.485690
C	-0.049300	-0.589103	4.170587
C	1.170256	-0.734306	3.573509
C	-2.417975	0.190903	4.181702
C	-3.260627	1.219313	4.045794
C	-4.557127	1.510084	4.650655
H	-5.078155	0.942820	5.418925
P	-3.283571	2.856616	3.153576
C	-3.706302	2.429785	1.407705
C	-3.034604	3.109116	0.390739
C	-3.276865	2.803291	-0.945289
C	-4.194586	1.814231	-1.273632
C	-4.874398	1.135346	-0.265310
C	-4.632576	1.442250	1.065787
C	-4.899330	2.667786	4.039416
C	-6.077768	3.520457	4.105366
C	-6.110731	4.723937	3.395122
C	-7.226240	5.548931	3.448423
C	-8.328826	5.181265	4.209762
C	-8.311721	3.980783	4.915347
C	-7.199168	3.156582	4.861732
H	-2.309445	3.875080	0.647884
H	-2.747740	3.336687	-1.727287
H	-4.384106	1.571879	-2.313406
H	-5.591115	0.362774	-0.520791
H	-5.159677	0.906656	1.849087
H	-0.196751	-0.931165	5.189986
H	1.991490	-1.194591	4.113518
H	-1.774987	0.844239	1.619163
H	3.466922	-0.868717	2.131797
H	3.765209	-0.094524	-0.186937
H	-0.334136	1.173124	-0.329488
H	1.864946	0.925614	-1.420906
H	-7.196854	2.219930	5.408675
H	-9.171524	3.687822	5.507470
H	-9.200029	5.824943	4.253904

H	-7.234289	6.480783	2.894524
H	-5.252162	5.014629	2.798216
H	-2.687004	-0.591979	4.890970

4b

E(ωB97X-D/6-311+G**)= -1343.449447

C	0.292344	0.687705	0.192085
C	0.207915	0.020572	1.411711
C	1.363249	-0.522532	1.978172
C	2.582581	-0.398754	1.330539
C	2.661049	0.270780	0.111498
C	1.517422	0.813590	-0.456794
P	-1.425582	-0.110359	2.241410
C	-1.077395	0.462858	3.931877
C	-0.776679	1.858032	4.264002
C	0.008568	2.163360	5.411994
C	0.319127	3.453177	5.731374
C	-0.123967	4.533254	4.924583
C	-0.900014	4.243100	3.772536
C	-1.209317	2.895352	3.469134
C	-1.346640	5.316127	2.959233
C	-1.033151	6.610404	3.275182
C	-0.256840	6.897700	4.420711
C	0.186488	5.882292	5.225300
C	-1.203009	-0.577078	4.791405
C	-1.433744	-1.862400	4.162016
H	-1.579902	-2.766953	4.744173
C	-1.481651	-1.835992	2.808136
C	-1.632617	-2.985332	1.909232
C	-1.098772	-4.232480	2.255454
C	-1.233681	-5.323241	1.409046
C	-1.891325	-5.188527	0.190827
C	-2.412312	-3.952371	-0.172183
C	-2.284064	-2.861728	0.677084
H	-0.602237	1.110652	-0.252991
H	1.576109	1.335363	-1.405336
H	3.615979	0.367340	-0.393038
H	3.476178	-0.823142	1.774620
H	1.298519	-1.041355	2.929405
H	0.383289	1.351258	6.025007
H	0.925417	3.666622	6.605953
H	-1.819809	2.689180	2.595134
H	0.782520	6.098641	6.106183
H	-0.014394	7.926873	4.660456
H	-1.942362	5.093624	2.079801
H	-1.380539	7.422296	2.646072
H	-2.709400	-1.905260	0.391915
H	-2.925546	-3.835732	-1.120205
H	-1.992588	-6.040624	-0.471636
H	-0.812053	-6.280296	1.695737
H	-0.555635	-4.339253	3.188164
H	-1.162480	-0.469808	5.870737

5b’

E(ωB97X-D/6-311+G**)= -1343.416796

C	0.101042	-0.065842	0.054140

C	0.005143	0.133194	1.433276
C	1.140653	0.528074	2.140866
C	2.352899	0.721194	1.484808
C	2.436944	0.519360	0.113912
C	1.308651	0.124328	-0.600745
P	-1.565975	-0.088254	2.378111
C	-2.416446	-1.527252	1.551825
C	-2.335039	-2.861675	1.553653
C	-1.364553	-3.697471	2.270910
C	-1.727170	-5.000073	2.633154
C	-0.851825	-5.817714	3.333526
C	0.413710	-5.353385	3.674917
C	0.795827	-4.069966	3.300894
C	-0.080189	-3.251349	2.601839
C	-2.832747	0.594077	1.213868
C	-3.331838	-0.616093	0.870224
H	-4.197067	-0.871010	0.261943
C	-3.169013	1.958338	0.842699
C	-2.425228	3.007946	1.330377
C	-2.699773	4.345969	0.959234
C	-3.767955	4.606758	0.061706
C	-4.528486	3.511226	-0.429347
C	-4.240119	2.232094	-0.055224
C	-4.035410	5.944413	-0.317840
C	-3.276514	6.974841	0.170867
C	-2.214223	6.715950	1.065620
C	-1.933115	5.432852	1.450536
H	1.788766	-3.706591	3.541801
H	1.100977	-5.991022	4.219373
H	1.076338	0.679255	3.214206
H	3.229422	1.028067	2.044550
H	3.379819	0.668600	-0.400417
H	1.373242	-0.032356	-1.671814
H	-0.776794	-0.372394	-0.505932
H	-2.714438	-5.367805	2.371340
H	-1.156205	-6.820620	3.611759
H	0.253126	-2.272118	2.281501
H	-4.829798	1.409294	-0.444163
H	-5.345839	3.709118	-1.115330
H	-1.599895	2.815243	2.009663
H	-4.851047	6.141661	-1.006073
H	-3.487396	7.995112	-0.129099
H	-1.620394	7.539898	1.444905
H	-1.117637	5.229128	2.136831
H	-3.087695	-3.403055	0.980329

5c

E(ωB97X-D/6-311+G**)= -1664.190975

C	0.366324	0.871244	0.444961
C	0.223358	0.306502	1.737727
C	1.340477	-0.331357	2.337549
C	2.565134	-0.382596	1.628852
C	2.675117	0.174046	0.382017
C	1.562840	0.806933	-0.216618
C	1.189837	-0.891419	3.634915
C	0.000964	-0.816854	4.298354

C	-1.125857	-0.176692	3.707533
C	-0.997653	0.368310	2.450701
C	-2.382334	-0.060340	4.427650
C	-2.796672	-0.466049	5.651806
H	-2.289746	-1.074056	6.397970
P	-4.009660	0.680375	3.951734
C	-3.698438	2.491996	4.097951
C	-2.862914	3.041575	5.072688
C	-2.650654	4.411009	5.125073
C	-3.271185	5.249529	4.202707
C	-4.104644	4.713815	3.230171
C	-4.316719	3.339478	3.179307
C	-4.131540	0.112814	5.724877
C	-5.004302	0.213220	6.734261
C	-6.302082	0.858828	6.736431
S	-7.051570	1.537578	5.322775
C	-8.429692	2.040879	6.222657
C	-8.326982	1.702151	7.537735
C	-7.113440	1.026299	7.830346
H	-2.376554	2.390650	5.792293
H	-1.998478	4.827945	5.884561
H	-3.103604	6.320152	4.243837
H	-4.590492	5.364145	2.511333
H	-4.972672	2.921568	2.421866
H	-0.092154	-1.246151	5.289840
H	2.043173	-1.379067	4.095149
H	-1.845518	0.866048	1.989015
H	3.418879	-0.868197	2.090519
H	3.618576	0.132425	-0.150430
H	1.662511	1.244407	-1.203561
H	-0.490212	1.357375	-0.010553
H	-9.238621	2.554649	5.725545
H	-9.090261	1.924235	8.271394
H	-6.837443	0.674703	8.816491
H	-4.719161	-0.217130	7.693409

4c

E(ωB97X-D/6-311+G**)= -1664.222321

P	-0.102917	-0.210909	0.013226
C	-0.077379	-0.070466	1.825478
C	1.202774	0.056505	2.253172
C	2.204533	-0.099740	1.218144
C	1.712480	-0.333603	-0.022729
C	2.477430	-0.579618	-1.247955
C	3.760410	-1.193227	-1.175586
C	4.485831	-1.445764	-2.303346
C	3.981589	-1.112676	-3.587346
C	2.699988	-0.509938	-3.675169
C	1.972827	-0.256495	-2.487253
C	2.186739	-0.174446	-4.954312
C	2.911459	-0.429038	-6.087097
C	4.186395	-1.032346	-5.998403
C	4.707630	-1.366042	-4.777023
C	-0.643627	-1.942766	-0.271333
C	-1.615526	-2.191469	-1.237492
C	-2.043217	-3.492937	-1.484160

C	-1.502066	-4.548119	-0.763578
C	-0.530976	-4.304794	0.205208
C	-0.102821	-3.009286	0.449937
C	-1.266801	-0.043512	2.658106
C	-1.395997	-0.460541	3.957258
C	-2.714040	-0.311296	4.465804
C	-3.575656	0.207797	3.547141
S	-2.790101	0.530176	2.050843
H	1.464780	0.280075	3.282193
H	-4.627145	0.422741	3.663133
H	-3.010205	-0.586317	5.469360
H	-0.573111	-0.886482	4.516300
H	-2.040644	-1.365521	-1.797863
H	-2.799510	-3.679591	-2.238187
H	-1.834942	-5.562437	-0.953536
H	-0.109410	-5.129362	0.769203
H	0.654228	-2.816054	1.203381
H	4.152105	-1.483678	-0.207158
H	5.458953	-1.920815	-2.229579
H	1.002267	0.223706	-2.568543
H	5.686558	-1.829191	-4.704652
H	4.750818	-1.228791	-6.903008
H	1.207476	0.288683	-5.020433
H	2.509058	-0.167551	-7.059343
H	3.264026	-0.003080	1.432775

5c'

E(ωB97X-D/6-311+G**)= -1664.190975

C	0.366324	0.871244	0.444961
C	0.223358	0.306502	1.737727
C	1.340477	-0.331357	2.337549
C	2.565134	-0.382596	1.628852
C	2.675117	0.174046	0.382017
C	1.562840	0.806933	-0.216618
C	1.189837	-0.891419	3.634915
C	0.000964	-0.816854	4.298354
C	-1.125857	-0.176692	3.707533
C	-0.997653	0.368310	2.450701
C	-2.382334	-0.060340	4.427650
C	-2.796672	-0.466049	5.651806
H	-2.289746	-1.074056	6.397970
P	-4.009660	0.680375	3.951734
C	-3.698438	2.491996	4.097951
C	-2.862914	3.041575	5.072688
C	-2.650654	4.411009	5.125073
C	-3.271185	5.249529	4.202707
C	-4.104644	4.713815	3.230171
C	-4.316719	3.339478	3.179307
C	-4.131540	0.112814	5.724877
C	-5.004302	0.213220	6.734261
C	-6.302082	0.858828	6.736431
S	-7.051570	1.537578	5.322775
C	-8.429692	2.040879	6.222657
C	-8.326982	1.702151	7.537735
C	-7.113440	1.026299	7.830346
H	-2.376554	2.390650	5.792293

H	-1.998478	4.827945	5.884561
H	-3.103604	6.320152	4.243837
H	-4.590492	5.364145	2.511333
H	-4.972672	2.921568	2.421866
H	-0.092154	-1.246151	5.289840
H	2.043173	-1.379067	4.095149
H	-1.845518	0.866048	1.989015
H	3.418879	-0.868197	2.090519
H	3.618576	0.132425	-0.150430
H	1.662511	1.244407	-1.203561
H	-0.490212	1.357375	-0.010553
H	-9.238621	2.554649	5.725545
H	-9.090261	1.924235	8.271394
H	-6.837443	0.674703	8.816491
H	-4.719161	-0.217130	7.693409

5d

E(ωB97X-D/6-311+G**)= -1116.019399

C	0.101987	0.861292	0.169639
C	-0.030621	0.026019	1.285797
C	1.064778	-0.750555	1.673879
C	2.259992	-0.695006	0.968854
C	2.379388	0.139024	-0.135787
C	1.295605	0.918407	-0.532019
C	-1.286089	-0.063438	2.020971
C	-2.507898	0.485063	1.834971
H	-2.847266	1.204702	1.092478
P	-1.745785	-0.924446	3.589761
C	-3.293397	-0.140628	2.899914
C	-4.606700	-0.167070	3.116651
C	-5.393024	-0.928062	4.159936
C	-6.015255	-2.158879	3.473036
C	-1.914026	-2.691091	3.074130
C	-2.381976	-3.070965	1.814210
C	-2.505226	-4.412357	1.483611
C	-2.162560	-5.394712	2.409774
C	-1.694613	-5.029083	3.664427
C	-1.569485	-3.682067	3.993007
C	-4.536836	-1.390223	5.342464
C	-6.513982	-0.012634	4.678465
H	-1.205461	-3.397603	4.975639
H	-1.425961	-5.789538	4.389297
H	-2.259397	-6.442870	2.149271
H	-2.867122	-4.695957	0.501489
H	-2.652199	-2.308637	1.090087
H	-0.736975	1.471696	-0.146655
H	1.383349	1.571200	-1.393355
H	3.311232	0.182992	-0.688152
H	3.098612	-1.306865	1.281474
H	0.975883	-1.407139	2.533522
H	-7.139483	-0.545254	5.401309
H	-6.100247	0.872204	5.170646
H	-7.158695	0.323622	3.860348
H	-5.176469	-1.854894	6.099121
H	-3.800043	-2.138752	5.041818

H	-4.012874	-0.551596	5.808336
H	-6.638818	-2.716483	4.179605
H	-6.644224	-1.863601	2.627587
H	-5.232890	-2.826089	3.100213
H	-5.223768	0.395935	2.412645

4d

E(ωB97X-D/6-311+G**)= -1116.052047

C	-0.087025	-0.162306	0.072445
C	-0.077648	-0.250794	1.420724
C	1.244490	-0.216176	2.026258
C	2.279996	-0.114636	1.163938
C	3.755963	-0.030568	1.509851
C	3.975339	-0.194213	3.018747
P	1.607679	0.140194	-0.516333
C	1.975354	-1.422917	-1.416540
C	1.674039	-2.671890	-0.869885
C	1.969979	-3.831772	-1.569324
C	2.570714	-3.756623	-2.824062
C	2.869943	-2.519046	-3.375630
C	2.571046	-1.354205	-2.673506
C	4.531095	-1.140010	0.776139
C	4.308542	1.339867	1.076535
H	-0.979049	-0.323348	2.021658
C	-1.251969	-0.248377	-0.815658
C	-2.359230	-1.030374	-0.465525
C	-3.461584	-1.118169	-1.303238
C	-3.475432	-0.439916	-2.517498
C	-2.376100	0.326719	-2.885059
C	-1.276172	0.422041	-2.043380
H	2.806229	-0.386776	-3.105094
H	3.334641	-2.454979	-4.353198
H	2.800136	-4.665108	-3.369927
H	1.730702	-4.798004	-1.139070
H	1.207414	-2.728377	0.108722
H	-2.342846	-1.589176	0.463902
H	-4.309036	-1.729780	-1.013551
H	-4.334435	-0.512986	-3.174788
H	-2.375541	0.856454	-3.831229
H	-0.429567	1.036321	-2.331682
H	5.370311	1.414930	1.331558
H	4.206070	1.486464	-0.001735
H	3.774634	2.150890	1.579583
H	5.044218	-0.143306	3.244411
H	3.477696	0.598045	3.585180
H	3.603586	-1.159499	3.374552
H	5.591219	-1.093814	1.044122
H	4.149356	-2.128600	1.045477
H	4.453833	-1.039091	-0.309025
H	1.364926	-0.260363	3.103235

5d’

E(ωB97X-D/6-311+G**)= -1116.020956

C	0.087451	-0.088323	-0.024990
C	0.148153	0.081243	1.359948
C	1.387705	0.300736	1.960116

C	2.546603	0.363116	1.190752
C	2.473937	0.195498	-0.185090
C	1.241389	-0.034366	-0.792142
P	-1.348863	0.054237	2.442560
C	-2.522120	1.177079	1.541833
C	-2.620702	2.670705	1.459610
C	-2.724037	3.242387	2.883791
C	-2.554281	-1.015152	1.507408
C	-3.287915	0.174156	1.067601
H	-4.225210	0.216439	0.516546
C	-2.771113	-2.315345	1.292468
C	-1.977625	-3.454676	1.770561
C	-0.630395	-3.347834	2.134558
C	0.068186	-4.452128	2.601663
C	-0.557399	-5.689643	2.706169
C	-1.889499	-5.815694	2.328946
C	-2.587926	-4.711192	1.861692
C	-1.350236	3.216185	0.783025
C	-3.854552	3.077266	0.646281
H	1.112174	-4.346823	2.875229
H	-0.007580	-6.550469	3.069570
H	1.447245	0.422262	3.037427
H	3.503725	0.538189	1.669492
H	3.374719	0.239885	-0.787239
H	1.183378	-0.171505	-1.866357
H	-0.871345	-0.265587	-0.502178
H	-3.628391	-4.816646	1.570483
H	-2.384935	-6.777920	2.396860
H	-0.110199	-2.404330	2.026099
H	-3.921849	4.167232	0.583486
H	-3.803714	2.683824	-0.373200
H	-4.773994	2.709396	1.110880
H	-2.774551	4.334992	2.848703
H	-3.619633	2.871525	3.389583
H	-1.854815	2.962518	3.485415
H	-1.395936	4.308093	0.724419
H	-0.451814	2.942989	1.343171
H	-1.243569	2.819391	-0.230156
H	-3.658251	-2.575961	0.714840

References

[1] U. Nagel and A. Bublewitz, *Chem. Ber.* **1992**, *125*, 1061–1072.
[2] A. S. Hay, *J. Org. Chem.* **1962**, *27*, 3320–3321.
[3] W. Chodkiewicz, *Ann. Chim. Paris* **1957**, *2*, 819.
[4] G. M. Sheldrick, *Acta Crystallogr., Sect. C: Struct. Chem.* **2015**, *71*, 3–8.
[5] O. V. Dolomanov, L. J. Bourhis, R. J. Gildea, J. A. K. Howard and H. Puschmann, *J Appl Crystallogr* **2009**, *42*, 339–341.
[6] C. F. Macrae, I. Sovago, S. J. Cottrell, P. T. A. Galek, P. McCabe, E. Pidcock, M. Platings, G. P. Shields, J. S. Stevens, M. Towler and P. A. Wood, *J Appl Crystallogr* **2020**, *53*, 226–235.
[7] M. J. Frisch, G. W. Trucks, H. B. Schlegel, G. E. Scuseria, M. A. Robb, J. R. Cheeseman, G. Scalmani, V. Barone, B. Mennucci, G. A. Petersson, H. Nakatsuji and X. L. M. Caricato, H. P. Hratchian, A. F. Izmaylov, J. Bloino, G. Zheng, J. L. Sonnenberg, M. Hada, M. Ehara, K. Toyota, R. Fukuda, J. Hasegawa, M. Ishida, T. Nakajima, Y. Honda, O. Kitao, H. Nakai, T. Vreven, J. A. Montgomery, Jr., J. E. Peralta, F. Ogliaro, M. Bearpark, J. J. Heyd, E. Brothers, K. N. Kudin, V. N. Staroverov, R. Kobayashi, J. Normand, K. Raghavachari, A. Rendell, J. C. Burant, S. S. Iyengar, J. Tomasi, M. Cossi, N. Rega, J. M. Millam, M. Klene, J. E. Knox, J. B. Cross, V. Bakken, C. Adamo, J. Jaramillo, R. Gomperts, R. E. Stratmann, O. Yazyev, A. J. Austin, R. Cammi, C. Pomelli, J. W. Ochterski, R. L. Martin, K. Morokuma, V. G. Zakrzewski, G. A. Voth, P. Salvador, J. J. Dannenberg, S. Dapprich, A. D. Daniels, Ö. Farkas, J. B. Foresman, J. V. Ortiz, J. Cioslowski, and D. J. Fox, in *Gaussian 09*, *Vol.* Wallingford, CT, **2009**.
[8] J.-D. Chai and M. Head-Gordon, *Phys.Chem. Chem.Phys.* **2008**, *10*, 6615-6620.
[9] M. Kállay, P. R. Nagy, D. Mester, Z. Rolik, G. Samu, J. Csontos, J. Csóka, P. B. Szabó, L. Gyevi-Nagy, B. Hégely, I. Ladjánszki, L. Szegedy, B. Ladóczki, K. Petrov, M. Farkas, P. D. Mezei and Á. Ganyecz, *J. Chem. Phys.* **2020**, *152*, 074107.
